# Consistency of metagenomic assignment programs in simulated and real data

**DOI:** 10.1186/1471-2105-15-90

**Published:** 2014-03-28

**Authors:** Koldo Garcia-Etxebarria, Marc Garcia-Garcerà, Francesc Calafell

**Affiliations:** 1 Institut de Biologia Evolutiva (CSIC-Universitat Pompeu Fabra), Barcelona, Spain

**Keywords:** Metagenomics, Assignment, Comparison

## Abstract

**Background:**

Metagenomics is the genomic study of uncultured environmental samples, which has been greatly facilitated by the advent of shotgun-sequencing technologies. One of the main focuses of metagenomics is the discovery of previously uncultured microorganisms, which makes the assignment of sequences to a particular taxon a challenge and a crucial step. Recently, several methods have been developed to perform this task, based on different methodologies such as sequence composition or sequence similarity. The sequence composition methods have the ability to completely assign the whole dataset. However, their use in metagenomics and the study of their performance with real data is limited. In this work, we assess the consistency of three different methods (BLAST + Lowest Common Ancestor, Phymm, and Naïve Bayesian Classifier) in assigning real and simulated sequence reads.

**Results:**

Both in real and in simulated data, BLAST + Lowest Common Ancestor (BLAST + LCA), Phymm, and Naïve Bayesian Classifier consistently assign a larger number of reads in higher taxonomic levels than in lower levels. However, discrepancies increase at lower taxonomic levels. In simulated data, consistent assignments between all three methods showed greater precision than assignments based on Phymm or Bayesian Classifier alone, since the BLAST + LCA algorithm performed best. In addition, assignment consistency in real data increased with sequence read length, in agreement with previously published simulation results.

**Conclusions:**

The use and combination of different approaches is advisable to assign metagenomic reads. Although the sensitivity could be reduced, the reliability can be increased by using the reads consistently assigned to the same taxa by, at least, two methods, and by training the programs using all available information.

## Background

Metagenomics is an emerging field that enables the genomic study of environmental samples, allowing the identification and characterization of bacterial and viral genomes previously unknown given the difficulty or impossibility to culture most species. The capacity of studying environments from a genomic point of view afforded by metagenomics is unprecedented [1]. A crucial step in metagenomics is sequence assignment, both taxonomic and functional: sequence reads need to be allocated to a genomic unit, or, at least, to a particular taxonomic level, and, in a further step, they may be mapped to a gene or set of genes with known functions. Different algorithms have been devised to assign sequences to taxonomic levels; they are based on sequence similarity, composition, phylogeny, or a combination of them [2]. However, at least two issues make taxonomic assignment difficult. First, the read length obtained by next generation sequencing technologies is not long enough to allow the original methods to properly assign the reads to low taxonomic levels (such as genus or species) due to the low sequence divergence between closely related taxonomic groups [3]. And second, since reference genomes are not available for many uncultured organisms, an incorrect assignment (or even no assignment at all) may be produced when no closely related species have been previously identified. The main approaches for assignment are based on sequence similarity and sequence composition. Packages using the former approach include MG-RAST [4] and MEGAN [5], while the Naïve Bayesian Classifier (such as implemented in Fragment Classification Package, FCP) [6] and the interpolated Markov model classification (IMM-based), used by Phymm [7] are based on composition similarity. The performance of assignment programs has been assessed using simulated and well-known experimental data [2,3,6-8]. In the case of composition-based programs, these methods can classify all the reads [2], and report the associated likelihood of the read to be assigned to the different categories. However, there are few metagenomic studies where these methods were used, e.g. [9-11].

To assess the relative performance of composition- vs. sequence-similarity methods, here we compare the Markov Model based (implemented in Phymm) and Bayesian-based (implemented in FCP) approaches against the classical pair wise sequence alignment (BLAST) in simulated metagenomic data and in data newly generated in our group. Our aim is to clarify which strategy could be the best to deal with taxonomic read assignment in metagenomic data.

## Results & discussion

Once metagenomic reads are obtained, taxonomic assignment is a crucial step that may determine subsequent analyses. Similarity-based approaches are the most commonly used but, due to the lack of reference sequences in the publicly available databases, a huge percentage of sequences are not assigned correctly or not assigned at all. For instance, between 23.5% and 29.6% of the reads were not assigned in the intestinal microbiome of the dog [12]. In our simulated data, using the BLAST + LCA (Lowest Common Ancestor) method implemented in FCP, between 0% and 3.65% of reads were not assigned at a domain level. Since all the sequences used are present in databases, it is not surprising that almost all reads were assigned. However, in two mouse skin metagenome real datasets (see Methods), 31.7% and 37.1% of the reads could not be assigned to a taxonomic domain. Thus, around one third of reads were of unknown origin in real data, although they are likely to be bacterial or viral, since the host genome is well characterized. As the reference databases grow, it is expected that the proportion of unassigned fragments will decrease.

The new, composition-based algorithms such in Phymm or FCP routinely classify all the reads [2]. However, they carry the risk of incorrectly assigning reads coming from unknown taxonomic units that may be closely related to some of the references. In order to deal with those possible incorrect assignments, a similarity assignment from BLAST is added to FCP and Phymm to provide some degree of reliability to the results obtained by their core composition-based algorithms [6,7]. And still, since all the methods rely on previously trained databases or on comparing their classifiers against known data, the biases should not be ignored, and wrong assignments could occur. Indeed, the performance of each method, in terms such as sensitivity and precision, has been investigated both with simulated data and with well-known data-sets separately [2,3,6,7], but, as far as we know, the analysis of the consistency of assignments by different methods with simulated and new data is limited.

### Consistency, sensitivity and precision in simulated data

We simulated different metagenomic datasets with different features (Additional file 1: Table S1) to assess the performance of the programs in diverse scenarios. Given that different simulation algorithms may mimic different environment scenarios, three different methods were used. We constructed four metagenomic environments with Metasim and two with iMESS, which are the most popular metagenomic simulation algorithms. Four additional datasets were simulated with a custom script developed in our lab to facilitate the use of the homemade databases and the creation of different scenarios. Accordingly, both Metasim and iMESS simulations were compared with our constructed datasets to test the robustness of our algorithm. Finally a set called “Synthetic” was constructed using 3 genomes of hypothetical bacteria constructed through a phylogenetic-based approach. Bacterial genomes were constructed with a script developed in our laboratory (Garcia-Garcerà, M. Manuscript in preparation). To deal with different complexity scenarios, two different levels of complexity (regarding the number of initial species) were used in our algorithm. In the first one, only bacteria were used, limiting the number of variants to the number of already sequenced bacteria. In the second situation, we included plasmids and other viral genomes (independently of their environmental association), constructing genomic sets with a much higher complexity.

**Figure 1:**
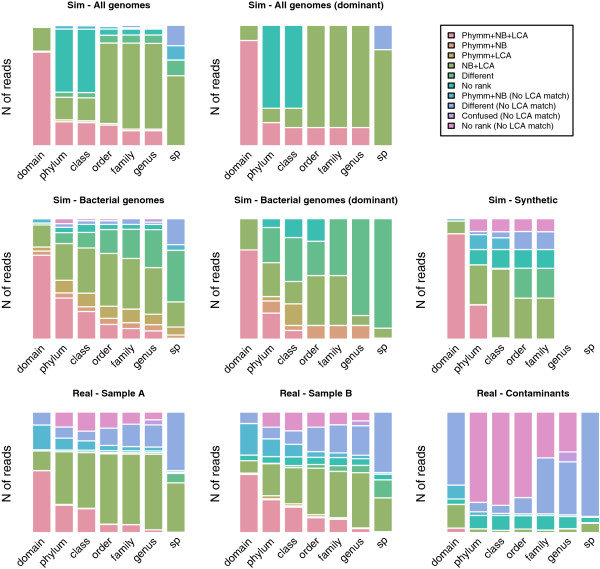
**Number of reads assigned in each taxonomic level in simulated and real data.** Sim, simulated data; Real, real data. NBC, Naïve Bayesian Classifier; LCA, Lowest Common Ancestor of BLAST results; Confused, genera that are known to be systematically confused between Phymm and Naïve Bayesian Classifier [6]. In the case of the Synthetic set, genus and species levels are not applicable since the “evolved” genomes were based on these or higher levels.

In the four simulated scenarios, a similar trend was observed (Figure 1). At domain level, the three assignment algorithms matched most of the reads to the same taxa (77.7% of reads were assigned to the same taxa for the “All genomes” set and 68.7% for the “Bacterial genomes” set). However, this consistency of assignment dropped dramatically in shallower taxonomic levels: no reads were assigned to the same species in the “All genomes” dataset and only 0.8% in the “Bacterial genomes”. The major difference between the “All genomes” and “Bacterial genomes” sets was the number of “no rank” assignments (at phylum level, 52.4% of reads were assigned as “no rank” in the “All genomes” dataset and 8.4% in the “Bacterial” dataset, respectively). These differences in the unranked assignments and the inconsistencies between the assignments by different methods may probably be due to the presence of viruses, which tend to be more variable and, then, more difficult to assign. Consistency on the assignments of dominant species datasets compared to their no-dominant counterparts was quite similar, e.g. at phylum level, reads assigned by all three methods to the same taxa was 19.2% in “All genomes” and 19.8% in “All genomes - Dominant”. Since these are simulated datasets, the consistency results could be separated between true and false positives (Additional file 2: Figure S1). The reads assigned to the same taxa by the three approaches used were defined as correctly assigned. In shallower taxonomic levels, the number of correctly assigned reads decreased, and the assignments were more discordant, especially in those that were wrongly assigned (Additional file 2: Figure S1). In the “Synthetic” set, the agreement between the three programs was higher at domain and phylum levels and in shallower levels, such as class or order, Bayesian Classifier and BLAST + LCA agreed in the taxonomic assignment of reads (Figure 1).

True-positive measures (sensitivity and precision) are shown in Table 1. Broadly, Phymm and NBC were less sensitive and precise than BLAST + LCA or than a combined strategy (where a read was assigned to a taxon if at least two methods converged). In addition, sensitivity and precision at genus level by Phymm and NBC were within the range previously described [2]. Thus, the use of a combined strategy improved the performance of assignments. However, this improvement was due to the effect of BLAST + LCA. In a more realistic scenario, where a high number of reads was not assigned by BLAST + LCA (that is, in a scenario where we find taxonomic units not previously described or without representatives in the reference database), the improvement would not be so remarkable. The results were very different between methods (Table 1) in the “Synthetic” dataset, that is, a metagenome simulation of three synthetic genomes (see Methods). The ability to assign correctly “evolved” genomes of a given taxonomic group was better in the case of BLAST + LCA (sensitivity 45.7% and precision 61.43% at class level) and poorer for PhymmBL (both sensitivity and precision 5.0% at class level). This lack of sensitivity in the artificially constructed genomes could be a result of the lack of reference sequences on the original databases used to train the programs. This result points out the importance of training to correctly assign metagenomic reads [2].

**Table 1:** Percentage sensitivity and precision in simulated datasets

	**Sensitivity**				**Precision**			
	**Phymm**	**Bayesian**	**BLAST + LCA**	**Combined**	**Phymm**	**Bayesian**	**BLAST + LCA**	**Combined**
Domain								
All genomes	79.45	98.41	98.97	99.16	79.46	98.41	98.97	99.90
All genomes (dominant)	87.36	100	100	100	87.36	100	100	100
Bact genomes	73.05	87.98	96.31	91.28	73.05	87.98	96.32	92.80
Bact genomes (dominant)	74.08	100	100	100	74.08	100	100	100
Synthetic	89.46	99.99	97.99	99.99	89.46	100	100	100
Phylum								
All genomes	22.37	39.65	47.60	41.18	39.65	78.99	93.76	95.46
All genomes (dominant)	19.20	31.16	31.16	31.16	85.31	100	100	100
Bact genomes	44.67	64.59	92.83	75.11	53.75	68.03	92.84	94.66
Bact genomes (dominant)	25.22	49.81	100	53.40	34.04	49.81	100	84.10
Synthetic	23.13	40.12	49.73	40.31	23.13	40.12	66.87	40.31
Class								
All genomes	21.45	39.30	47.51	40.46	30.40	78.31	93.58	94.91
All genomes (dominant)	15.12	31.16	31.16	31.16	68.66	100	100	100
Bact genomes	34.24	60.43	95.22	71.72	42.65	63.65	95.23	94.79
Bact genomes (dominant)	25.22	25.82	100	43.78	34.04	25.82	100	91.41
Synthetic	5.03	30.56	45.69	30.56	5.03	30.56	61.43	30.56
Order								
All genomes	26.55	38.23	47.60	40.45	39.40	78.32	93.58	94.91
All genomes (dominant)	15.12	31.16	31.16	31.16	67.41	31.16	31.16	31.16
Bact genomes	22.24	56.07	95.22	71.72	42.64	63.64	95.22	94.79
Bact genomes (dominant)	0	41.40	100	41.40	0	41.40	100	78.45
Synthetic	4.7	30.56	45.69	30.56	4.7	30.56	61.43	30.56
Family								
All genomes	25.31	38.15	47.60	39.37	32.86	39.15	48.84	45.67
All genomes (dominant)	15.12	31.16	31.16	31.16	51.82	31.16	31.16	31.16
Bact genomes	20.18	50.89	95.22	66.06	26.69	56.87	95.22	92.83
Bact genomes (dominant)	0	41.40	100	41.40	0	41.40	100	78.45
Synthetic	0	30.56	45.69	30.56	0	30.56	61.43	30.56
Genus								
All genomes	25.30	37.97	47.60	39.17	32.37	38.95	48.84	45.56
All genomes (dominant)	15.12	31.16	31.16	31.16	50.41	31.16	31.16	31.16
Bact genomes	15.56	45.56	95.22	54.23	17.44	46.18	95.23	91.36
Bact genomes (dominant)	0	8.22	100	8.22	0	8.22	100	41.97
Synthetic	NA	NA	NA	NA	NA	NA	NA	NA
Species								
All genomes	17.94	36.44	24.46	23.03	17.94	36.44	24.46	32.89
All genomes (dominant)	0	31.16	10.86	10.86	0	31.16	10.86	13.62
Bact genomes	13.12	39.75	73.71	32.96	13.12	39.75	73.72	92.45
Bact genomes (dominant)	0	8.22	100	8.22	0	8.22	100	90.18
Synthetic	NA	NA	NA	NA	NA	NA	NA	NA

*NA*, not applicable. In the case of the Synthetic set, genus and species levels are not applicable since the “evolved” genomes were based on these or higher levels. The features of each dataset is explained in Methods and their descriptive and diversity parameters in Additional file 1: Table S1.

To assess the robustness of the results obtained with our script to generate simulated metagenomic data and the use of different scenarios, MetaSim [13] and iMESS were used to simulate metagenomic datasets. However, the results obtained in those simulations were very similar between them and more consistent than the results obtained in the “Bacterial genomes” dataset (Additional file 3: Figure S2). For example, at phylum level, the three programs assigned to the same taxa 51.33% of reads in “Metasim 1” set, 51.25% in “Metasim 2” set, 49.86% in “Metasim 3” set and 51.56% in “Metasim 4”, compared to the 33.68% of convergent assignments obtained in the “Bacterial” dataset. In the case of sensitivity and precision a similar trend was observed (Additional file 1: Table S1).

### Consistency in real data

The number of consistent assignments in both samples was quite similar and it depended on the depth of taxonomic level (Figure 1). At domain level around half of the reads were consistently assigned to the same taxa by the three approaches used, at phylum it decreased to around a quarter, and at species level there were only few agreements. The main difference compared to simulated data is that BLAST + LCA does not improve the assignment, perhaps because the misassignments are due to the lack of representatives in the reference databases, since previously uncharacterized taxonomic units may be present in the environment sampled.

In real data, ~12%-15% “no rank” assignments were consistently assigned by all three methods. This percentage may reflect truly uncharacterized individuals, since skin is the least studied ecosystem on the body, given its extension and the difficulty to reach the commensal community. Moreover, 11 genera are consistently confused between Phymm and NBC [6], and we have labeled them separately as “confused”; these genera comprised 4.2% of sample A and 4.1% of sample B in our real datasets.

Regarding the “contaminant” dataset (Figure 1), with the exception of the domain level, most reads were assigned to different taxonomic group or to the “no rank” group. Only 11.5% of the read were consistently assigned by Phymm and NBC to the same bacterial or viral taxa. Those reads consistently assigned kept some degree of uncertainty since they are differently assigned by the third method, and, more importantly, were assigned as “contaminants” while they would be considered as part of the commensal community.

In general, it is a good approach to take into account the reads consistently assigned to the same taxa by, at least, two programs but being careful with those assigned by Phymm and NBC but not by BLAST + LCA. One may consider that BLAST + LCA unassignment or missassignment indicates the lack of a representative taxonomic unit in the reference databases, but still it should be taken carefully. Given the low consistency, a large fraction of the reads were assigned to different taxa by each program. The proportion of differently assigned reads correlated negatively with phylogenetic depth, from 11-13% at domain level to 57-79% at species level in real data (Figure 1). At domain level, Phymm tended to assign as viruses the reads that NBC assigns as bacteria (Table 2), at phylum the most repeated confusions were observed in Cyanobacteria (Phymm) with Firmicutes (NBC), Firmicutes with Proteobacteria and Proteobacteria with Firmicutes (Table 2). The proportion of reads that are assigned to different taxa could be used as guiding information at phylum level but it should be taken carefully.

**Table 2:** Summary of assignments of reads that were not assigned to the same taxa by phymm and NBC

	**A sample**			**B sample**		
	**Assigned by Phymm as**	** Assigned by NBC as**	** %**	**Assigned by Phymm as**	** Assigned by NBC as**	** %**
Domain	Virus	Bacteria	89.92	Virus	Bacteria	87.5
	Archaea	Bacteria	3.57	Virus	Archaea	4.6
	Virus	Archaea	2.69	Bacteria	Virus	3.72
	Bacteria	Archaea	2.08	Archaea	Bacteria	2.46
	Bacteria	Virus	1.74	Bacteria	Archaea	1.65
				Archaea	Virus	0.06
Phylum	Cyanobacteria	Firmicutes	15.36	Proteobacteria	Firmicutes	15.75
	Firmicutes	Proteobacteria	15.34	Firmicutes	Proteobacteria	14.62
	Proteobacteria	Firmicutes	13.55	Cyanobacteria	Proteobacteria	9.51
	Cyanobacteria	Proteobacteria	8.47	Proteobacteria	Bacteroidetes	5.26
	Firmicutes	Super Bacteroidetes/Chlorobi group	3.91	Proteobacteria	Cyanobacteria	4.85
	Proteobacteria	Super Bacteroidetes/Chlorobi group	3.56	Firmicutes	Cyanobacteria	4.76
	Proteobacteria	Cyanobacteria	3.05	Cyanobacteria	Firmicutes	4.26
	Firmicutes	Cyanobacteria	2.74	Proteobacteria	Super Bacteroidetes/Chlorobi group	3.14
	Firmicutes	Fusobacteria	2.56	Firmicutes	Super Bacteroidetes/Chlorobi group	2.93
	Proteobacteria	Bacteroidetes	2.16	Proteobacteria	Actinobacteria	2.27
Class	Bacilli	Gammaproteobacteria	10.75	Alphaproteobacteria	Gammaproteobacteria	6.43
	Bacilli	Clostridia	6.54	Gammaproteobacteria	Bacilli	5.59
	Gammaproteobacteria	Bacilli	6.36	Bacilli	Gammaproteobacteria	5.46
	Bacilli	Eproteobacteria	5.94	Gammaproteobacteria	Betaproteobacteria	4.97
	Gammaproteobacteria	Betaproteobacteria	4.73	Gammaproteobacteria	Alphaproteobacteria	4.38
	Gammaproteobacteria	Clostridia	4.33	Gammaproteobacteria	Actinobacteria (class)	4.37
	Negativicutes	Bacilli	2.73	Alphaproteobacteria	Betaproteobacteria	4.2
	Bacilli	Flavobacteria	2.65	Bacilli	Eproteobacteria	3.71
	Alphaproteobacteria	Clostridia	2.5	Gammaproteobacteria	Eproteobacteria	2.56
	Bacilli	Fusobacteria (class)	2.35	Gammaproteobacteria	Clostridia	2.51

Percent of reads differently assigned at this taxonomic level.

### Effect of read length in consistency in real data

**Figure 2:**
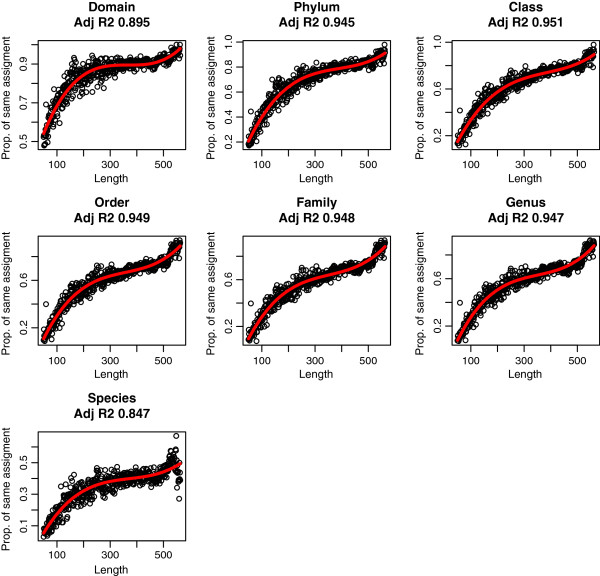
**Proportion of reads assigned to the same taxa by, at least, two methods according to length of reads at different taxonomic levels.** The red line shows the polynomial regression.

Although 88.7% and 86.1% of reads (in samples A and B, respectively) were assigned consistently at domain level and 76.9% and 71.9% at phylum, we observed a correlation between the consistency of the assignment and the read length (Figure 2). Combining both datasets and classifying them by size, we observe a tendency of improving consistency at lower taxonomic levels: to reach a 75% agreement by at least two approaches, a 83 bp length was needed at domain level, 254 bp at phylum, 355 bp at class, 430 bp at order, 440 bp at family and 448 bp at genus. Like in composition-based methods, in BLAST searches the limitation of assignment by read length has been previously suggested [14]. We extend this suggestion to all methodologies. Thus, in the design of metagenomic projects where proper assignment is crucial, these limitations should be taken into account to choose the sequencing platform in order to achieve the objectives of the study.

### Performance of scores and cut-off values of each program in real data

**Figure 3:**
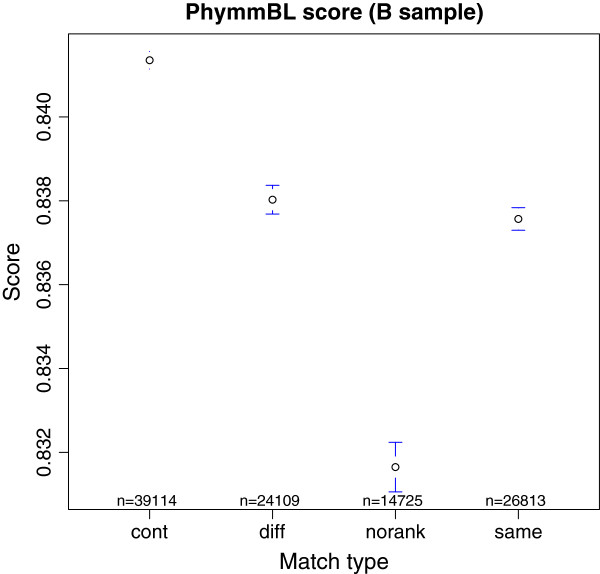
**Mean and 95% of confidence interval of PhymmBL results in real sample B by each category at phylum level.** Cont, contaminant reads (false positives); diff, reads assigned to different taxa by Phymm and FCP; norank, reads assigned to species with no rank at phylum; same, reads assigned to the same taxon.

PhymmBL provides a confidence score for assignments that, although it is not associated with a statistical significance, it is informative, and ranges from 0 (worst) to 1 (best) [7]. Since in one of the samples we kept the contaminant reads (a sort of false positives), the average value given by PhymmBL of each category of reads at phylum level could be compared (Figure 3): the average for contaminant reads was 0.8413 (±0.0211), for reads assigned to different taxa by PhymmBL and NBC it was 0.8380 (±0.02712), for reads with no rank assignment at phylum it was 0.8316 (±0.0366) and for reads assigned to the same taxa it was 0.8375 (±0.0225). Surprisingly, the highest confidence value was associated to the contaminant dataset. Differences between categories were statistically significant (ANOVA, p < 2 × 10^−16^).

**Figure 4:**
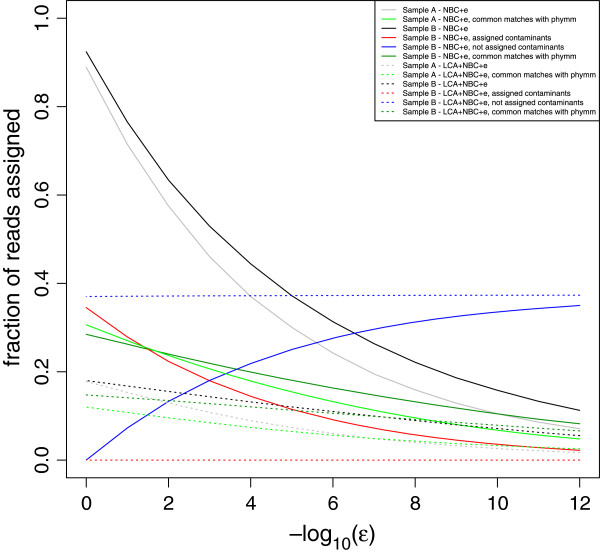
**Performance of two programs implemented in fragment classification package in different combinations and conditions at phylum level.** NBC, Naïve Bayesian Classifier; LCA + NBC, combination of results from LCA based on BLAST search and Naïve Bayesian Classifier. The optimal ϵ value suggested by the authors is 10^−5^[6].

On the other hand, one may use the “ϵ value” as a cut-off value of consistency with BLAST + LCA. The combination of the Bayesian algorithm with the BLAST + LCA is performed in order to obtain more significant results [6]. When an ϵ value of 0 is used, without BLAST + LCA combination, almost all the reads are assigned in both samples at phylum level (Figure 4) and the number of assigned reads decreased similarly in both samples with higher ϵ values. In the combined BLAST + LCA + Bayesian methods, when the ϵ value is 0, only a third of the reads are assigned. Besides, regarding contaminants, the number of contaminant reads assigned decreases with the ϵ value in the Bayesian classification. In the case of BLAST + LCA + Bayesian, contaminant reads were not classified at all. Then, it seems that the use of BLAST + LCA reduces the false-positive assignments as well as the overall assignments, and the use of ϵ value could be omitted. In the case of only Bayesian assignment, the ϵ value is necessary. Based on previous studies, the recommended ϵ value is 10^−5^[6], at which point false positives match true positives. Therefore, a higher value of ϵ should be used to increase sensitivity without increasing the false positive rate. For a conservative approach, an ϵ value of 10^−10^[6] is recommended, but it could be lower without raising the number of false positives at phylum level. In any case, the number of reads assigned to the same taxa by Phymm and NBC was almost similar regardless the ϵ value in the purely Bayesian approach and in the combined BLAST + LCA + Bayesian approach (Figure 4).

## Conclusions

After analyzing the metagenomic assignment with three different approaches, since each one has its biases and uncertainties, the use and combination of different approaches is advisable, as it was advised in the use of gene prediction methods in metagenomic samples [15]. We recommend to use the reads consistently assigned to the same taxa by, at least, two methods, whereas the reads that are assigned to different taxa should be used with caution and taking into account the lack of consistency. New approaches performing a combination of methods, including Markov models, Bayesian and similarity-based algorithms may improve the confidence in taxonomic assignment, while the expansion of the reference databases will reduce the level of uncertainty.

## Methods

### Sampling

Two mouse skin samples were collected and processed according to the protocol we previously developed [16]. Mice belonged to the C57BL/6J strain, they were unrelated and were euthanized according to a protocol approved by a local IRB board (Parc de Recerca Biomèdica de Barcelona, Animal Research committee). DNA collected was then sheared by sonication using Bioruptor™ (Diagenode). 454™ libraries were prepared according to Zheng et al [17]. Sequencing was performed using an FLX Titanium platform. These samples were collected as part of a larger project to analyze the skin microbiota in health and disease [16]. These two samples were used as validation for a new methodology produced in our laboratory. Real data has been deposited to MG-RAST database (accession numbers 4496968.3 and 4496969.3).

### Simulations

Three methods were chosen to simulate the metagenomic environments: first a customized perl script was constructed to perform simulations based on expected rarefaction curve slopes, maximum diversity H’ index, and maximum number of reads. This script generates metagenomic datasets using the existing databases of reference genomes [18] and uses the H’ index and the expected slope of the rarefaction curve to design the metagenome. The script uses the following pipeline: according to the user-selected variables, the algorithm assigns the probabilities for each taxon to exist on the environment according to the H’ index. With the expected number of reads and the maximum expected number of taxa on the environment, the script constructs the line between point zero (0,0 co-ordinates in the diversity space) and the maximum sampling point (number of expected reads, number of expected taxa). With the number of reads and the probability for each taxon to exist on the ecosystem, the algorithm calculates the maximum linear point of the rarefaction curve (the last point where the logarithm of species discovery per read is linear), and with those three points (point zero, maximum linear point, and maximum sampling point), the method fits the curve to the expected logarithmic equation. The script then randomly fragments the selected genomes with a fragment size range from 350-600bp and randomly samples the expected number of reads for each genome, according to the expected number of taxa on each point of the rarefaction curve and applies to each read the sequencing platform error rate (separating both homopolymeric region modification and point mismatch). The script allows also to simulate environments according to the maximum threshold of sequences for one species. In this case, four diversity scenarios were simulated, each one with 60,000 reads, since in real data the average number of reads was around 60,000: “All genomes” set, where bacterial and viral genomes were used and the maximum relative amount for species was 5%; “All genomes (dominant)” set, where bacterial and viral genomes were used and the maximum relative amount for a species was 80%; “Bacterial genomes” set, where only bacterial genomes were used and a maximum relative amount of 5%; and “Bacterial genomes (dominant)”, where only bacterial genomes were used with a maximum relative amount of 80%.

To test the consistency of results obtained by our script, four simulations were constructed using Metasim [13] using the default 454 generation option (Lognormal Distribution Mean: 0.23; Lognormal Distribution Std. Deviation: 0.15; Proportionality Constant for Std. Deviation: 0.15) and two simulations with iMESS using the following conditions: Method A (Species Selection) including all bacterial phyla to the selection without selection of genome size of GC content. We limited the most abundant species to 100 individuals. Given that our script used a logarithmic distribution, the same model was used for iMESS, with base e. We selected the 454 platform as sequencing method, with the Titanium read size (150-650 with mean at 350 and normal distribution) and an expected read count of 50K (equivalent to 1/16th of a plate).

The features and genomes and number of reads of the six simulated datasets are shown in Additional file 1: Tables S2, S3 and S4. The synthetic and simulated datasets are provided in Additional files 4 and 5.

Finally, a set with synthetic genomes was built and analyzed. Three synthetic genomes were built based on Burkholderiales, Desulfovibrio and Thermacea known genomes, and coding regions were simulated through a hidden-Markov model-based sequence generation, using a custom script (Garcia-Garcerà, M, manuscript in preparation).

### Informatic analyses

To assess the specificity and consistency of the different programs, simulated data were used. In real data, reads from the host were removed from the two metagenomic samples of mouse (NBCI37/mm9 genome version) skin using Deconseq [19] with the default options and retaining bacterial/viral reads. For the latter, Deconseq uses the genomes of bacteria and viruses described in Additional file 1: Table S4, to calculate the read coverage and alignment identity that is used to classify a specific read as contaminant. Moreover, to measure the effect of contaminants in metagenomic sequence assignment, the reads assigned as contaminants were used to construct an additional dataset. In total, 60,488 reads in Sample A, 65,647 in Sample B and 39,114 in the Contaminant dataset were analyzed. Overall, average read length was 374 bp.

BLAST [20], Phymm [7] and Naïve Bayesian Classifier implemented in FCP [6] were used to assign the metagenomic reads in simulated and real data. BLAST searches were carried out against a custom database constructed using the following method: reference bacterial and viral genomes and draft genomes were downloaded from the NCBI FTP repository and concatenated in a single file. The Fasta file including all genomes was formatted using the formatdb software included in BLAST. This database was used for all BLAST queries (Additional file 1: Table S4). Results from BLAST were parsed with a Lowest Common Ancestor (LCA) script implemented in FCP, using a cutoff e-value of 10^−5^. In addition, Phymm and NBC were trained with the same bacterial and viral data (Additional file 1: Table S4). The results of both programs were improved using BLAST results (PhymmBL and NB + BLAST programs, respectively). In addition, BLAST + LCA and Bayesian Classifiers were parsed with FCP using different ϵ-values (from 0 to 10^−12^).

The assignment of each read by the different programs was compared to calculate the consistency of metagenomic assignments. The read were classified according to the number of programs that produced the same taxonomic assignment. In some cases, such as phyla in viruses, a particular taxonomic level may not exist. Such situations were labeled as “no rank”. Homemade scripts written in PHP and Python were used to perform these comparisons. The sensitivity (number of correct assignments/number of sequences in the data set) and precision (number of correct assignments/number of assignments made) were calculated as in [2]. These two parameters were applied to each method used and to the combined assignment. These scripts are available at https://github.com/koldogarcia/metagenomics.

The graphical representation of results and statistical analyses were performed with the R language [21].

## Competing interests

The authors declare that they have no competing interests.

## Authors’ contributions

KGE, MGG and FC conceived the work. KGE performed the analyses. MGG provided the real metagenomic data, and wrote the simulation script. KGE wrote the manuscript, with revisions and contributions by MGG and FC. All authors read and approved the final manuscript.

## Supplementary Material

Additional file 1: Table S1 Descriptive and diversity parameters of each simulated dataset. **Table S2.** Sensitivity and precision in simulated datasets generated by MetaSim. **Table S3.** List of genomes and number of reads used in each simulated dataset. **Table S4.** List of genomes used to build BLAST database and to train Phymm and NBC. Click here for file

Additional file 2: Figure S1 Number of reads assigned in each taxonomic level in simulated data according to true positives and false positives. Click here for file

Additional file 3: Figure S2 Number of reads assigned in each taxonomic level in simulated data generated by Metasim. Click here for file

Additional file 4 Simulated and synthetic sequences generated using our scripts in FASTA format, and their keys. Click here for file

Additional file 5 Simulated sequences generated using iMESS in FASTA format, and their keys. Click here for file
