# Supplementary material for: Consistency of metagenomic assignment programs in simulated and real data
Source: BMC Bioinformatics. 2014 Mar 28;15:90. doi: 10.1186/1471-2105-15-90 (PMC3986635; doi:10.1186/1471-2105-15-90)
Supplement: Additional file 1: Table S1 — Descriptive and diversity parameters of each simulated dataset. Table S2. Sensitivity and precision in simulated datasets generated by MetaSim. Table S3. List of genomes and number of reads used in each simulated dataset. Table S4. List of genomes used to build BLAST database and to train Phymm and NBC. [file 1471-2105-15-90-S1.docx]

Additional file 1.

Supplementary Table S1: Descriptive and diversity parameters of each simulated dataset. Page 2.

Supplementary Table S2: Sensitivity (in %) and precision (in %) in simulated datasets generated by MetaSim. Page 3.

Supplementary Table S3: List of genomes and number of reads used in each simulated dataset. Page 4.

Supplementary Table S4: List of genomes used to build BLAST database and to train Phymm and NBC. Page 40.

Additional data files:

simulated_files.zip: simulated and synthetic sequences generated using our scripts in FASTA format, and their keys
simulated_metasim.zip: simulated sequences generated using metasim in FASTA format
simulated_imess.zip: simulated sequences generated using iMESS in FASTA format, and their keys

Supplementary Table S1: Descriptive and diversity parameters of each simulated dataset.

| **Simulated dataset** | **Analyzed bp** | **Number of reads** | **Genomes used** | **Number of reads from most abundant genome** | **Size of read** | | | | **H’** |
| --- | --- | --- | --- | --- | --- | --- | --- | --- | --- |
|  |  |  |  |  | **Average** | **Standard deviation** | **Maximum** | **Minimum** |  |
| All genomes | 19513675 | 60000 | 42 | 2918 | 325.2 | 100.71 | 499 | 151 | 3.52 |
| All genomes (dominant) | 19451450 | 60000 | 4 | 40523 | 324.2 | 100.57 | 499 | 151 | 0.89 |
| Bacterial genomes | 19784302 | 59170 | 42 | 2981 | 334.4 | 116.36 | 987 | 151 | 3.54 |
| Bacterial genomes (dominant) | 19532051 | 60000 | 2 | 32460 | 325.5 | 100.85 | 151 | 499 | 0.69 |
| MetaSim1 | 16052049 | 60000 | 1261 | 219 | 267.5 | 19.1 | 394 | 202 | 6.70 |
| MetaSim2 | 16055024 | 60000 | 1265 | 223 | 267.6 | 19.23 | 413 | 193 | 6.71 |
| MetaSim3 | 16354917 | 60000 | 16 | 8640 | 272.6 | 16.55 | 368 | 194 | 2.28 |
| MetaSim4 | 16282072 | 60000 | 10 | 10548 | 271.4 | 16.22 | 359 | 197 | 1.99 |
| iMESS1 | 12526912 | 49560 | 125 | 2414 | 252.8 | 2.40 | 264 | 243 | 4.61 |
| iMESS2 | 12464015 | 49308 | 135 | 2348 | 252.8 | 2.40 | 264 | 243 | 4.60 |

Supplementary Table S2: Sensitivity (in %) and precision (in %) in simulated datasets generated by MetaSim.

|  | **Sensitivity** | | | | **Precision** | | | |
| --- | --- | --- | --- | --- | --- | --- | --- | --- |
|  | **Phymm** | **Bayesian** | **LCA** | **Combined** | **Phymm** | **Bayesian** | **LCA** | **Combined** |
| *Phylum* |  |  |  |  |  |  |  |  |
| MetaSim 1 | 62.41 | 77.73 | 95.55 | 85.45 | 73.78 | 84.11 | 100 | 96.58 |
| MetaSim 2 | 62.27 | 77.88 | 95.5 | 85.56 | 73.71 | 84.14 | 100 | 96.51 |
| MetaSim 3 | 65.39 | 68.73 | 94 | 84.04 | 79.85 | 85.71 | 100 | 84.04 |
| MetaSim 4 | 65.98 | 74.09 | 99.68 | 88.27 | 80.73 | 87.55 | 100 | 88.28 |
| iMESS1 | 53.48 | 66.87 | 77.51 | 71.72 | 62.76 | 71.79 | 82.57 | 80.89 |
| iMESS2 | 56.43 | 68.05 | 79.57 | 73.03 | 66.01 | 72.95 | 84.34 | 81.84 |
| *Class* |  |  |  |  |  |  |  |  |
| MetaSim 1 | 42.63 | 57.49 | 73.41 | 62.85 | 52.84 | 63.91 | 76.69 | 77.42 |
| MetaSim 2 | 42.64 | 57.82 | 73.43 | 62.95 | 52.92 | 64.12 | 76.7 | 77.47 |
| MetaSim 3 | 62.42 | 66.94 | 93.98 | 82.22 | 79.47 | 84.15 | 100 | 82.23 |
| MetaSim 4 | 62.14 | 72.03 | 99.67 | 86 | 79.52 | 85.75 | 100 | 86 |
| iMESS1 | 37.43 | 53.09 | 65.78 | 56.46 | 45.57 | 58.49 | 69.77 | 69.7 |
| iMESS2 | 40.32 | 53.48 | 66.08 | 56.95 | 48.83 | 58.67 | 69.75 | 69.8 |
| *Order* |  |  |  |  |  |  |  |  |
| MetaSim 1 | 36.86 | 57.43 | 87.94 | 62.26 | 42.43 | 62.57 | 91.84 | 91.31 |
| MetaSim 2 | 44.63 | 66.97 | 90.58 | 72.77 | 52.76 | 71.85 | 94.24 | 92.5 |
| MetaSim 3 | 32.34 | 50.33 | 93.89 | 61.62 | 39.74 | 58.26 | 94.26 | 61.63 |
| MetaSim 4 | 24.99 | 56.08 | 99.55 | 61.27 | 30.84 | 65.83 | 100 | 61.27 |
| iMESS1 | 35.39 | 55.17 | 74.64 | 57.82 | 41.54 | 58.45 | 78.45 | 75.34 |
| iMESS2 | 37.93 | 56 | 75.72 | 59.08 | 44.47 | 59.38 | 79.58 | 76.7 |
| *Family* |  |  |  |  |  |  |  |  |
| MetaSim 1 | 33.05 | 52.52 | 86.74 | 56.9 | 36.51 | 54.08 | 90.68 | 87.97 |
| MetaSim 2 | 36.63 | 57.89 | 87.7 | 62.63 | 42.21 | 62.98 | 91.63 | 91.54 |
| MetaSim 3 | 24.91 | 40.07 | 93.6 | 49.85 | 29.49 | 46.34 | 94.24 | 49.85 |
| MetaSim 4 | 15.42 | 47.24 | 99.11 | 47.19 | 18.5 | 55.41 | 100 | 47.19 |
| iMESS1 | 31.87 | 51.59 | 72.41 | 53.8 | 36.42 | 55.7 | 76.74 | 76.71 |
| iMESS2 | 34.64 | 50.97 | 72.53 | 53.66 | 39.49 | 54.93 | 76.9 | 77.44 |
| *Genus* |  |  |  |  |  |  |  |  |
| MetaSim 1 | 33.05 | 52.52 | 86.74 | 56.9 | 36.51 | 54.08 | 90.68 | 87.97 |
| MetaSim 2 | 32.81 | 53.01 | 86.55 | 57.2 | 36.3 | 54.56 | 90.46 | 88.25 |
| MetaSim 3 | 24.14 | 40.07 | 93.59 | 49.82 | 27.5 | 45.71 | 94.24 | 49.82 |
| MetaSim 4 | 14.99 | 47.24 | 99.09 | 47.15 | 17.2 | 54.65 | 100 | 47.15 |
| iMESS1 | 27.46 | 43.68 | 72.64 | 46.42 | 30.19 | 45.19 | 77.61 | 73.15 |
| iMESS2 | 28.89 | 42.44 | 73.99 | 45.3 | 31.8 | 44.09 | 79.23 | 74.33 |
| *Species* |  |  |  |  |  |  |  |  |
| MetaSim 1 | 20.97 | 32.11 | 50.89 | 27.33 | 20.97 | 32.11 | 78.95 | 79.82 |
| MetaSim 2 | 20.65 | 32.2 | 50.51 | 27.24 | 20.65 | 32.2 | 78.45 | 79.79 |
| MetaSim 3 | 20.36 | 19.63 | 78.75 | 28.45 | 20.36 | 19.63 | 93.23 | 28.45 |
| MetaSim 4 | 10.19 | 21.67 | 80.26 | 20.36 | 10.19 | 21.67 | 100 | 20.36 |
| iMESS1 | 17.92 | 23.41 | 42.49 | 21.19 | 17.92 | 23.41 | 67.73 | 60.72 |
| iMESS2 | 19.52 | 25.29 | 45.2 | 24.26 | 19.52 | 25.29 | 69.93 | 66.59 |

Supplementary Table S3: List of genomes and number of reads used in each simulated dataset.

| **Genome** | **Number of reads** |
| --- | --- |
| *All genomes* |  |
| Elm mottle virus | 2918 |
| Agrotis segetum granulovirus | 2845 |
| Vibrio_Vibrio_cholerae_V52 | 2689 |
| Streptococcus pneumoniae G54 | 2664 |
| Mycobacterium phage Cali | 2628 |
| Simplexvirus_Human_herpesvirus_1 | 2511 |
| Nairovirus_Crimean_Congo_hemorrhagic_fever_virus | 2332 |
| Fluviicola taffensis DSM 16823 | 2281 |
| Fusobacterium_Fusobacterium_1_1_41FAA | 2268 |
| Enterovirus_Human_enterovirus_A | 2261 |
| Streptococcus mutans UA159 | 2207 |
| Mycobacterium phage ScottMcG | 2203 |
| Begomovirus_Okra_leaf_curl_virus | 2192 |
| Lactobacillus gasseri ATCC 33323 | 2049 |
| Tomato yellow leaf curl Mali virus associated DNA beta | 1919 |
| Iltovirus_Psittacid_herpesvirus_1 | 1782 |
| Staphylococcus_Staphylococcus_aureus_TCH130 | 1781 |
| Listeria_Listeria_monocytogenes_FSL_N3_165 | 1571 |
| Chlorobium chlorochromatii CaD3 | 1561 |
| Nitrosococcus halophilus Nc4 | 1544 |
| Verrucomicrobiae bacterium DG1235 | 1518 |
| Staphylococcus epidermidis RP62A | 1504 |
| Bacteroides phage B40 8 | 1408 |
| Granulicatella_Granulicatella_adiacens_ATCC_49175 | 1316 |
| Bromovirus_Broad_bean_mottle_virus | 1304 |
| Clostridium_Clostridium_leptum_DSM_753 | 1267 |
| Anaeromyxobacter dehalogenans 2CP-C | 1101 |
| Parechovirus_Human_parechovirus | 918 |
| Shewanella_Shewanella_benthica_KT99 | 864 |
| Aurantimonas_Aurantimonas_manganoxydans_SI85_9A1 | 731 |
| Tomato leaf curl Sulawesi virus | 581 |
| Aeromicrobium_Aeromicrobium_marinum_DSM_15272 | 538 |
| Candidatus Riesia pediculicola USDA | 487 |
| Bacillus_Bacillus_thuringiensis_serovar_tochigiensis_BGSC_4Y1 | 462 |
| Acidimicrobium ferrooxidans DSM 10331 | 395 |
| Haemophilus influenzae PittGG | 319 |
| Geobacter metallireducens GS-15 | 306 |
| Alicycliphilus denitrificans K601 | 292 |
| Begomovirus associated DNA II | 217 |
| Streptococcus pyogenes MGAS2096 | 163 |
| Candidatus_Zinderia_Candidatus_Pelagibacter_ubique_HTCC1002 | 86 |
| Enterococcus_Enterococcus_faecalis_X98 | 17 |
| *All genomes (dominant)* |  |
| Staphylococcus phage EW | 40523 |
| Burkholderia ambifaria MC40-6 | 12180 |
| Anaerofustis_Anaerofustis_stercorihominis_DSM_17244 | 6514 |
| Foveavirus_Peach_chlorotic_mottle_virus | 783 |
| *Bacterial genomes* |  |
| Arthrobacter arilaitensis Re117 | 2981 |
| Halorhabdus utahensis DSM 12940 | 2943 |
| Candidatus Sulcia muelleri DMIN | 2741 |
| Mycoplasma leachii PG50 | 2661 |
| Nitrosococcus oceani ATCC 19707 | 2383 |
| Azorhizobium caulinodans ORS 571 | 2348 |
| Caulobacter crescentus CB15 | 2295 |
| Mycoplasma penetrans HF-2 | 2279 |
| Methanoculleus marisnigri JR1 | 2216 |
| Methanobrevibacter smithii ATCC 35061 | 2094 |
| Granulibacter bethesdensis CGDNIH1 | 2025 |
| Calditerrivibrio nitroreducens DSM 19672 | 2000 |
| Serratia proteamaculans 568 | 1934 |
| Burkholderia pseudomallei MSHR346 | 1868 |
| Pseudomonas putida F1 | 1846 |
| Desulfarculus baarsii DSM 2075 | 1820 |
| Caldicellulosiruptor bescii DSM 6725 | 1810 |
| Salinibacter ruber M8 | 1607 |
| Akkermansia muciniphila ATCC BAA-835 | 1519 |
| Bacteroides thetaiotaomicron VPI-5482 | 1412 |
| Streptococcus pyogenes MGAS315 | 1383 |
| Thermococcus barophilus MP | 1369 |
| Robiginitalea biformata HTCC2501 | 1291 |
| Anaeromyxobacter dehalogenans 2CP-1 | 1264 |
| Xylella fastidiosa Temecula1 | 1206 |
| Macrococcus caseolyticus JCSC5402 | 1125 |
| Rhodopseudomonas palustris CGA009 | 1119 |
| Paenibacillus polymyxa SC2 | 1077 |
| Burkholderia cenocepacia HI2424 | 1074 |
| Dickeya dadantii Ech586 | 925 |
| Helicobacter pylori G27 | 703 |
| Pseudomonas aeruginosa PAO1 | 631 |
| Leptothrix cholodnii SP-6 | 625 |
| Leptotrichia buccalis C-1013-b | 558 |
| Helicobacter hepaticus ATCC 51449 | 460 |
| Streptococcus pyogenes NZ131 | 397 |
| Exiguobacterium sibiricum 255-15 | 381 |
| Pelobacter propionicus DSM 2379 | 339 |
| Colwellia psychrerythraea 34H | 231 |
| Borrelia duttonii Ly | 217 |
| Thermotoga naphthophila RKU-10 | 10 |
| Nautilia profundicola AmH | 3 |
| Bact genomes (dominant) |  |
| Riemerella anatipestifer DSM 15868 | 32460 |
| Thermoanaerobacterium thermosaccharolyticum DSM 571 | 27540 |
| *MetaSim 1* |  |
| Catenulispora acidiphila DSM 44928 | 219 |
| Solibacter usitatus Ellin6076 | 213 |
| Streptosporangium roseum DSM 43021 | 210 |
| Bradyrhizobium sp. BTAi1 | 208 |
| Haliangium ochraceum DSM 14365 | 208 |
| Streptomyces griseus subsp. griseus NBRC 13350 | 203 |
| Myxococcus xanthus DK 1622 chromosome | 191 |
| Frankia alni ACN14a | 185 |
| Nostoc punctiforme PCC 73102 | 184 |
| Chitinophaga pinensis DSM 2588 | 184 |
| Rhodococcus jostii RHA1 | 181 |
| Frankia sp. EAN1pec | 180 |
| Bacillus licheniformis ATCC 14580 | 178 |
| Actinosynnema mirum DSM 43827 | 175 |
| Saccharopolyspora erythraea NRRL 2338 chromosome | 168 |
| Conexibacter woesei DSM 14684 | 168 |
| Gluconacetobacter diazotrophicus PAl 5 | 167 |
| Hahella chejuensis KCTC 2396 chromosome | 166 |
| Bradyrhizobium japonicum USDA 110 chromosome | 162 |
| Trichodesmium erythraeum IMS101 | 159 |
| Spirosoma linguale DSM 74 | 158 |
| Kribbella flavida DSM 17836 | 155 |
| Stackebrandtia nassauensis DSM 44728 chromosome | 154 |
| Pseudomonas fluorescens SBW25 chromosome | 154 |
| Bradyrhizobium sp. ORS278 | 153 |
| Pseudomonas aeruginosa PAO1 chromosome | 151 |
| Mycobacterium smegmatis str. MC2 155 | 150 |
| Mesorhizobium loti MAFF303099 chromosome | 148 |
| Nakamurella multipartita DSM 44233 | 148 |
| Methylobacterium nodulans ORS 2060 | 147 |
| Dyadobacter fermentans DSM 18053 | 145 |
| Mycobacterium marinum M | 145 |
| Herpetosiphon aurantiacus ATCC 23779 chromosome | 142 |
| Pseudomonas aeruginosa PA7 | 141 |
| Rhodopirellula baltica SH 1 chromosome | 137 |
| Desulfitobacterium hafniense Y51 | 137 |
| Bacillus anthracis str. Sterne chromosome | 136 |
| Pseudoalteromonas atlantica T6c | 135 |
| Photorhabdus luminescens subsp. laumondii TTO1 | 134 |
| Methylobacterium populi BJ001 | 134 |
| Cyanothece sp. PCC 7424 | 134 |
| Nocardiopsis dassonvillei subsp. dassonvillei DSM 43111 chromosome | 134 |
| Acaryochloris marina MBIC11017 | 133 |
| Pseudomonas entomophila L48 | 130 |
| Mycobacterium avium 104 | 130 |
| Klebsiella pneumoniae 342 | 130 |
| Mycobacterium sp. MCS | 129 |
| Pedobacter heparinus DSM 2366 | 129 |
| Pseudomonas putida KT2440 chromosome | 129 |
| Corynebacterium glutamicum ATCC 13032 | 129 |
| Rhodococcus erythropolis PR4 | 128 |
| Rhodopseudomonas palustris CGA009 chromosome | 128 |
| Methanosarcina acetivorans C2A chromosome | 128 |
| Mycobacterium sp. KMS | 127 |
| Xanthomonas axonopodis pv. citri str. 306 chromosome | 126 |
| Methylobacterium chloromethanicum CM4 | 126 |
| Flavobacterium johnsoniae UW101 | 126 |
| Roseiflexus castenholzii DSM 13941 | 125 |
| Nocardia farcinica IFM 10152 | 125 |
| Shewanella woodyi ATCC 51908 | 124 |
| Pseudomonas syringae pv. phaseolicola 1448A | 124 |
| Methylobacterium extorquens AM1 | 123 |
| Pseudomonas putida W619 | 123 |
| Bacillus cereus B4264 | 122 |
| Salmonella enterica subsp. enterica serovar Dublin str. CT_02021853 | 122 |
| Pseudomonas syringae pv. tomato str. DC3000 chromosome | 122 |
| Cyanothece sp. PCC 7822 chromosome | 122 |
| Escherichia coli E24377A | 122 |
| Salmonella enterica subsp. enterica serovar Paratyphi B str. SPB7 chromosome | 122 |
| Bacteroides vulgatus ATCC 8482 chromosome | 122 |
| Bacillus cereus ATCC 10987 | 122 |
| Bordetella petrii DSM 12804 | 122 |
| Desulfobacterium autotrophicum HRM2 | 121 |
| Mycobacterium sp. JLS | 121 |
| Shewanella baltica OS185 | 121 |
| Shigella flexneri 2a str. 301 | 121 |
| Paenibacillus polymyxa E681 chromosome | 121 |
| Yersinia pestis Antiqua | 120 |
| Shigella sonnei Ss046 | 120 |
| Xanthobacter autotrophicus Py2 | 120 |
| Burkholderia multivorans ATCC 17616 chromosome 2 | 120 |
| Teredinibacter turnerae T7901 | 120 |
| Bacillus cereus ATCC 14579 | 118 |
| Thermomonospora curvata DSM 43183 | 118 |
| Shewanella baltica OS223 chromosome | 118 |
| Photorhabdus asymbiotica chromosome | 117 |
| Xanthomonas oryzae pv. oryzae PXO99A | 117 |
| Methylobacterium extorquens PA1 | 117 |
| Frankia sp. CcI3 | 116 |
| Clostridium beijerinckii NCIMB 8052 | 116 |
| Escherichia coli APEC O1 | 116 |
| Nostoc sp. PCC 7120 | 115 |
| Burkholderia phytofirmans PsJN chromosome 1 | 115 |
| Escherichia coli B str. REL606 chromosome | 115 |
| Anabaena variabilis ATCC 29413 | 115 |
| Rhodopseudomonas palustris BisA53 | 115 |
| Methylobacterium radiotolerans JCM 2831 | 114 |
| Bordetella bronchiseptica RB50 | 114 |
| Shewanella baltica OS195 | 114 |
| Escherichia coli 536 | 114 |
| Shewanella frigidimarina NCIMB 400 | 113 |
| Mycobacterium ulcerans Agy99 | 113 |
| Methylocella silvestris BL2 chromosome | 112 |
| Bacillus thuringiensis str. Al Hakam | 112 |
| Aeromonas hydrophila subsp. hydrophila ATCC 7966 | 112 |
| Bacillus cereus E33L | 112 |
| Colwellia psychrerythraea 34H | 112 |
| Rhodopseudomonas palustris HaA2 | 111 |
| Bacillus cereus G9842 | 111 |
| Parabacteroides distasonis ATCC 8503 | 111 |
| Candidatus Koribacter versatilis Ellin345 | 111 |
| Cellulomonas flavigena DSM 20109 chromosome | 111 |
| Klebsiella pneumoniae subsp. pneumoniae MGH 78578 | 111 |
| Xanthomonas campestris pv. campestris str. 8004 chromosome | 110 |
| Rhodoferax ferrireducens T118 | 110 |
| Xanthomonas campestris pv. campestris str. ATCC 33913 | 110 |
| Rhodospirillum rubrum ATCC 11170 | 110 |
| Escherichia coli HS | 110 |
| Yersinia pestis CO92 | 110 |
| Azotobacter vinelandii DJ chromosome | 110 |
| Polaromonas sp. JS666 | 109 |
| Shewanella piezotolerans WP3 chromosome | 109 |
| Dickeya dadantii 3937 chromosome | 109 |
| Azorhizobium caulinodans ORS 571 | 109 |
| Rhodopseudomonas palustris BisB18 | 108 |
| Xanthomonas oryzae pv. oryzae MAFF 311018 | 108 |
| Pseudomonas putida F1 | 108 |
| Bacillus cereus AH820 | 108 |
| Bacillus weihenstephanensis KBAB4 | 108 |
| Mesorhizobium sp. BNC1 | 108 |
| Pseudomonas syringae pv. syringae B728a | 108 |
| Bacillus anthracis str. Ames | 107 |
| Clostridium phytofermentans ISDg | 107 |
| Planctomyces limnophilus DSM 3776 chromosome | 107 |
| Yersinia pseudotuberculosis YPIII chromosome | 107 |
| Cyanothece sp. PCC 7425 | 107 |
| Chromobacterium violaceum ATCC 12472 | 106 |
| Escherichia coli CFT073 | 106 |
| Cyanothece sp. PCC 8802 | 106 |
| Cellvibrio japonicus Ueda107 | 106 |
| Marinomonas sp. MWYL1 chromosome | 105 |
| Bacillus cereus Q1 chromosome | 105 |
| Serratia proteamaculans 568 chromosome | 105 |
| Psychromonas ingrahamii 37 | 104 |
| Shigella flexneri 5 str. 8401 | 104 |
| Desulfovibrio salexigens DSM 2638 | 104 |
| Bacteroides fragilis YCH46 chromosome | 104 |
| Anaeromyxobacter sp. K | 103 |
| Polaromonas naphthalenivorans CJ2 | 103 |
| Bordetella parapertussis 12822 | 103 |
| Geobacter uraniireducens Rf4 chromosome | 103 |
| Caulobacter sp. K31 chromosome | 103 |
| Geodermatophilus obscurus DSM 43160 | 103 |
| Sphingomonas wittichii RW1 | 103 |
| Escherichia coli UTI89 | 102 |
| Beijerinckia indica subsp. indica ATCC 9039 | 102 |
| Phenylobacterium zucineum HLK1 | 102 |
| Yersinia pestis Nepal516 | 102 |
| Shigella flexneri 2a str. 2457T | 102 |
| Jannaschia sp. CCS1 | 102 |
| Acinetobacter baumannii AB0057 | 102 |
| Alkaliphilus metalliredigens QYMF | 102 |
| Mycobacterium tuberculosis KZN 1435 | 102 |
| Janthinobacterium sp. Marseille | 102 |
| Rhizobium leguminosarum bv. trifolii WSM1325 | 102 |
| Mycobacterium tuberculosis CDC1551 | 101 |
| Marinobacter aquaeolei VT8 | 101 |
| Shewanella baltica OS155 | 101 |
| Rhizobium leguminosarum bv. viciae 3841 | 100 |
| Caulobacter segnis ATCC 21756 chromosome | 100 |
| Xanthomonas oryzae pv. oryzae KACC10331 chromosome | 100 |
| Escherichia coli str. K-12 substr. W3110 chromosome | 100 |
| Geobacter bemidjiensis Bem | 99 |
| Sebaldella termitidis ATCC 33386 | 99 |
| Rhodopseudomonas palustris BisB5 | 99 |
| Gordonia bronchialis DSM 43247 | 99 |
| Pectobacterium atrosepticum SCRI1043 chromosome | 98 |
| Yersinia pseudotuberculosis IP 32953 | 98 |
| Salmonella enterica subsp. enterica serovar Paratyphi C strain RKS4594 | 98 |
| Mycobacterium tuberculosis H37Rv | 98 |
| Yersinia pestis KIM 10 chromosome | 98 |
| Yersinia pestis biovar Microtus str. 91001 | 97 |
| Dechloromonas aromatica RCB | 97 |
| Geobacter sp. M21 | 97 |
| Arthrobacter chlorophenolicus A6 | 96 |
| Nocardioides sp. JS614 | 96 |
| Pseudomonas mendocina ymp | 96 |
| Shewanella pealeana ATCC 700345 | 96 |
| Syntrophobacter fumaroxidans MPOB | 96 |
| Acidovorax sp. JS42 | 96 |
| Pseudomonas stutzeri A1501 | 96 |
| Aeromonas salmonicida subsp. salmonicida A449 | 95 |
| Salmonella enterica subsp. enterica serovar Newport str. SL254 | 95 |
| Shigella boydii Sb227 | 95 |
| Salmonella enterica subsp. enterica serovar Heidelberg str. SL476 | 95 |
| Thauera sp. MZ1T | 95 |
| Bordetella pertussis Tohama I | 94 |
| Escherichia coli str. K-12 substr. MG1655 chromosome | 94 |
| Nitrosococcus halophilus Nc4 chromosome | 94 |
| Beutenbergia cavernae DSM 12333 | 94 |
| Yersinia pseudotuberculosis IP 31758 | 94 |
| Desulfotomaculum acetoxidans DSM 771 | 93 |
| Methanosarcina barkeri str. fusaro chromosome | 93 |
| Clostridium botulinum A str. Hall | 93 |
| Chloroflexus aggregans DSM 9485 chromosome | 93 |
| Salmonella enterica subsp. enterica serovar Typhimurium str. LT2 chromosome | 92 |
| Bacteroides fragilis NCTC 9343 chromosome | 92 |
| Methylibium petroleiphilum PM1 chromosome | 92 |
| Mycobacterium tuberculosis F11 | 92 |
| Kineococcus radiotolerans SRS30216 | 92 |
| Rhizobium leguminosarum bv. trifolii WSM2304 | 92 |
| Salmonella enterica subsp. enterica serovar Typhi str. CT18 chromosome | 91 |
| Aromatoleum aromaticum EbN1 | 91 |
| Bacillus cereus AH187 | 91 |
| Cyanothece sp. ATCC 51142 chromosome chromosome circular | 91 |
| Sanguibacter keddieii DSM 10542 | 91 |
| Rhodospirillum centenum SW chromosome | 90 |
| Xenorhabdus nematophila ATCC 19061 | 90 |
| Burkholderia phytofirmans PsJN chromosome 2 | 90 |
| Saccharomonospora viridis DSM 43017 | 90 |
| Oligotropha carboxidovorans OM5 | 90 |
| Leptospira interrogans serovar Lai str. 56601 chromosome chromosome I | 90 |
| Rhizobium sp. NGR234 | 89 |
| Clostridium thermocellum ATCC 27405 | 89 |
| Ralstonia pickettii 12J chromosome 1 | 89 |
| Burkholderia pseudomallei 1710b chromosome I | 89 |
| Cytophaga hutchinsonii ATCC 33406 | 89 |
| Clostridium botulinum F str. Langeland | 89 |
| Burkholderia pseudomallei K96243 chromosome 1 | 89 |
| Gamma proteobacterium HdN1 | 89 |
| Desulfomicrobium baculatum DSM 4028 | 89 |
| Rhizobium etli CIAT 652 | 89 |
| Pelobacter propionicus DSM 2379 | 89 |
| Yersinia pestis Pestoides F | 89 |
| Escherichia coli ATCC 8739 | 88 |
| Burkholderia xenovorans LB400 chromosome 1 | 88 |
| Salmonella enterica subsp. enterica serovar Schwarzengrund str. CVM19633 | 88 |
| Agrobacterium radiobacter K84 chromosome 1 | 88 |
| Salmonella enterica subsp. enterica serovar Typhi str. Ty2 chromosome | 88 |
| Clostridium botulinum Ba4 str. 657 | 88 |
| Arthrobacter sp. FB24 chromosome chromosome 1 | 88 |
| Cyanothece sp. PCC 8801 | 88 |
| Clostridium botulinum A str. ATCC 19397 | 88 |
| Butyrivibrio proteoclasticus B316 chromosome 1 | 88 |
| Salmonella enterica subsp. enterica serovar Agona str. SL483 | 88 |
| Tsukamurella paurometabola DSM 20162 chromosome | 87 |
| Acinetobacter baumannii ACICU | 87 |
| Shewanella denitrificans OS217 | 87 |
| Flavobacteriales bacterium HTCC2170 chromosome | 87 |
| Stenotrophomonas maltophilia K279a chromosome | 87 |
| Thermobispora bispora DSM 43833 chromosome | 87 |
| Shigella dysenteriae Sd197 | 87 |
| Fibrobacter succinogenes subsp. succinogenes S85 | 87 |
| Halomicrobium mukohataei DSM 12286 | 86 |
| Acinetobacter baumannii ATCC 17978 | 86 |
| Ralstonia pickettii 12D chromosome 1 | 85 |
| Nitrobacter hamburgensis X14 | 85 |
| Candidatus Desulfococcus oleovorans Hxd3 | 85 |
| Rhizobium etli CFN 42 | 85 |
| Burkholderia mallei NCTC 10247 chromosome I | 85 |
| Acidobacterium capsulatum ATCC 51196 | 85 |
| Clostridium botulinum A str. ATCC 3502 | 85 |
| Bacillus subtilis subsp. subtilis str. 168 | 85 |
| Burkholderia ambifaria AMMD chromosome 1 | 84 |
| Clostridium botulinum A3 str. Loch Maree | 84 |
| Clostridium botulinum E3 str. Alaska E43 | 84 |
| Agrobacterium vitis S4 chromosome 1 | 84 |
| Salmonella enterica subsp. enterica serovar Paratyphi A str. ATCC 9150 chromosome | 84 |
| Arthrobacter aurescens TC1 | 84 |
| Gloeobacter violaceus PCC 7421 chromosome | 84 |
| Azoarcus sp. BH72 | 84 |
| Burkholderia sp. 383 chromosome 1 | 83 |
| Photobacterium profundum SS9 chromosome 1 | 83 |
| Enterobacter sp. 638 | 83 |
| Brachybacterium faecium DSM 4810 | 83 |
| Roseobacter denitrificans OCh 114 | 83 |
| Candidatus Nitrospira defluvii | 83 |
| Bordetella avium 197N | 82 |
| Clostridium botulinum B str. Eklund 17B | 82 |
| Bacillus pseudofirmus OF4 chromosome | 82 |
| Novosphingobium aromaticivorans DSM 12444 | 82 |
| Mycobacterium bovis BCG str. Pasteur 1173P2 | 82 |
| Burkholderia pseudomallei 1710b chromosome II | 81 |
| Burkholderia pseudomallei 1106a chromosome II | 81 |
| Staphylococcus aureus subsp. aureus JH9 | 81 |
| Desulfovibrio vulgaris subsp. vulgaris DP4 | 81 |
| Legionella pneumophila str. Paris | 81 |
| Burkholderia cenocepacia HI2424 chromosome 1 | 81 |
| Yersinia pestis Angola | 81 |
| Yersinia enterocolitica subsp. enterocolitica 8081 | 81 |
| Rhodobacter sphaeroides ATCC 17025 chromosome | 80 |
| Burkholderia pseudomallei 1106a chromosome I | 80 |
| Burkholderia thailandensis E264 chromosome I | 80 |
| Oceanobacillus iheyensis HTE831 | 80 |
| Burkholderia vietnamiensis G4 chromosome 1 | 80 |
| Mycobacterium tuberculosis H37Ra | 80 |
| Methanosarcina mazei Go1 chromosome | 79 |
| Vibrio parahaemolyticus RIMD 2210633 chromosome 1 | 79 |
| Cupriavidus metallidurans CH34 chromosome chromosome 1 | 79 |
| Thermobifida fusca YX | 79 |
| Clostridium difficile 630 | 79 |
| Acinetobacter sp. ADP1 | 78 |
| Pelobacter carbinolicus DSM 2380 | 78 |
| Brachyspira murdochii DSM 12563 chromosome | 78 |
| Clostridium kluyveri DSM 555 | 78 |
| Halorhabdus utahensis DSM 12940 chromosome | 78 |
| Burkholderia sp. 383 chromosome 2 | 78 |
| Prevotella ruminicola 23 chromosome | 78 |
| Xylanimonas cellulosilytica DSM 15894 | 78 |
| Burkholderia pseudomallei 668 chromosome I | 77 |
| Clostridium acetobutylicum ATCC 824 | 77 |
| Geobacter sulfurreducens PCA chromosome | 77 |
| Gluconobacter oxydans 621H chromosome | 77 |
| Desulfovibrio desulfuricans subsp. desulfuricans str. G20 chromosome | 77 |
| Salinibacter ruber DSM 13855 | 77 |
| Bacillus selenitireducens MLS10 chromosome | 77 |
| Shewanella amazonensis SB2B | 76 |
| Dinoroseobacter shibae DFL 12 | 76 |
| Meiothermus silvanus DSM 9946 chromosome | 76 |
| Burkholderia cenocepacia J2315 chromosome chromosome 1 | 76 |
| Maricaulis maris MCS10 | 75 |
| Bdellovibrio bacteriovorus HD100 | 75 |
| Clavibacter michiganensis subsp. michiganensis NCPPB 382 | 75 |
| Aliivibrio salmonicida LFI1238 chromosome 1 | 75 |
| Burkholderia multivorans ATCC 17616 chromosome 1 | 75 |
| Geobacter lovleyi SZ | 74 |
| Sinorhizobium medicae WSM419 | 74 |
| Robiginitalea biformata HTCC2501 | 74 |
| Rhodobacter sphaeroides 2.4.1 chromosome 1 | 74 |
| Acidithiobacillus ferrooxidans ATCC 53993 | 73 |
| Burkholderia xenovorans LB400 chromosome 2 | 73 |
| Acinetobacter sp. DR1 chromosome | 73 |
| Eubacterium rectale ATCC 33656 | 73 |
| Gramella forsetii KT0803 | 73 |
| Cupriavidus taiwanensis str. LMG19424 chromosome I | 73 |
| Clostridium cellulolyticum H10 | 73 |
| Clostridium botulinum B1 str. Okra | 73 |
| Geobacillus kaustophilus HTA426 | 73 |
| Burkholderia cenocepacia AU 1054 chromosome 1 | 72 |
| Ralstonia solanacearum GMI1000 | 72 |
| Acinetobacter baumannii AYE | 72 |
| Escherichia coli str. K-12 substr. DH10B | 72 |
| Meiothermus ruber DSM 1279 chromosome | 72 |
| Erythrobacter litoralis HTCC2594 chromosome | 72 |
| Ralstonia eutropha H16 chromosome 1 | 72 |
| Geobacillus sp. WCH70 | 72 |
| Nitrosococcus oceani ATCC 19707 | 72 |
| Legionella pneumophila str. Lens | 71 |
| Sinorhizobium meliloti 1021 | 71 |
| Methylococcus capsulatus str. Bath | 71 |
| Pseudoalteromonas haloplanktis TAC125 chromosome I | 71 |
| Corynebacterium glutamicum R chromosome | 71 |
| Lactobacillus casei str. Zhang chromosome | 71 |
| Staphylococcus aureus subsp. aureus MW2 | 71 |
| Xanthomonas albilineans chromosome | 71 |
| Nitrosomonas eutropha C91 | 71 |
| Nitrosospira multiformis ATCC 25196 chromosome | 71 |
| Vibrio cholerae O395 chromosome 2 | 71 |
| Clostridium perfringens SM101 | 70 |
| Burkholderia pseudomallei 668 chromosome II | 70 |
| Psychrobacter cryohalolentis K5 | 70 |
| Burkholderia mallei SAVP1 chromosome I | 70 |
| Synechocystis sp. PCC 6803 chromosome | 70 |
| Acidithiobacillus ferrooxidans ATCC 23270 | 70 |
| Vibrio sp. Ex25 chromosome 1 | 70 |
| Syntrophus aciditrophicus SB chromosome | 70 |
| Desulfotalea psychrophila LSv54 | 69 |
| Chromohalobacter salexigens DSM 3043 | 69 |
| Parvularcula bermudensis HTCC2503 chromosome | 69 |
| Listeria monocytogenes str. 4b F2365 | 68 |
| Caulobacter crescentus CB15 | 68 |
| Eggerthella lenta DSM 2243 | 68 |
| Sphingopyxis alaskensis RB2256 | 68 |
| Legionella pneumophila str. Corby chromosome | 68 |
| Alcanivorax borkumensis SK2 | 68 |
| Alkaliphilus oremlandii OhILAs | 68 |
| Clavibacter michiganensis subsp. sepedonicus | 68 |
| Rhodothermus marinus DSM 4252 | 68 |
| Staphylococcus aureus subsp. aureus str. Newman | 67 |
| Bacillus amyloliquefaciens FZB42 | 67 |
| Rhodobacter capsulatus SB 1003 chromosome | 67 |
| Vibrio vulnificus CMCP6 chromosome I | 67 |
| Caldicellulosiruptor saccharolyticus DSM 8903 | 67 |
| Alicyclobacillus acidocaldarius subsp. acidocaldarius DSM 446 | 67 |
| Haloquadratum walsbyi DSM 16790 | 66 |
| Edwardsiella tarda EIB202 chromosome | 66 |
| Clostridium perfringens ATCC 13124 | 66 |
| Xylella fastidiosa M12 chromosome | 66 |
| Leptotrichia buccalis DSM 1135 | 66 |
| Burkholderia multivorans ATCC 17616 chromosome chromosome 1 | 65 |
| Burkholderia mallei NCTC 10229 chromosome I | 65 |
| Sulfolobus solfataricus P2 chromosome | 65 |
| Renibacterium salmoninarum ATCC 33209 chromosome | 65 |
| Enterococcus faecalis V583 chromosome | 65 |
| Herminiimonas arsenicoxydans | 65 |
| Haloarcula marismortui ATCC 43049 chromosome chromosome I | 65 |
| Hyphomonas neptunium ATCC 15444 | 65 |
| Propionibacterium acnes KPA171202 | 64 |
| Natronomonas pharaonis DSM 2160 | 64 |
| Bifidobacterium longum subsp. infantis ATCC 15697 | 64 |
| Desulfovibrio desulfuricans subsp. desulfuricans str. ATCC 27774 | 64 |
| Symbiobacterium thermophilum IAM 14863 | 64 |
| Clostridium perfringens str. 13 | 64 |
| Kangiella koreensis DSM 16069 | 64 |
| Methylobacillus flagellatus KT | 64 |
| Burkholderia mallei ATCC 23344 chromosome 1 | 64 |
| Lactobacillus brevis ATCC 367 | 63 |
| Halothermothrix orenii H 168 | 63 |
| Heliobacterium modesticaldum Ice1 | 63 |
| Sphaerobacter thermophilus DSM 20745 chromosome 1 | 63 |
| Vibrio vulnificus YJ016 chromosome I | 63 |
| Burkholderia cenocepacia J2315 chromosome chromosome 2 | 63 |
| Rhodobacter sphaeroides ATCC 17029 chromosome chromosome 1 | 63 |
| Clostridium tetani E88 chromosome | 63 |
| Staphylococcus aureus subsp. aureus MRSA252 chromosome | 63 |
| Legionella pneumophila subsp. pneumophila str. Philadelphia 1 chromosome | 63 |
| Acinetobacter baumannii SDF | 63 |
| Denitrovibrio acetiphilus DSM 12809 chromosome | 63 |
| Ralstonia eutropha JMP134 chromosome 1 | 62 |
| Vibrio fischeri ES114 chromosome I | 62 |
| Desulfovibrio vulgaris str. Hildenborough chromosome | 62 |
| Slackia heliotrinireducens DSM 20476 | 61 |
| Croceibacter atlanticus HTCC2559 chromosome | 61 |
| Syntrophomonas wolfei subsp. wolfei str. Goettingen | 61 |
| Chloroherpeton thalassium ATCC 35110 | 61 |
| Exiguobacterium sp. AT1b | 61 |
| Halorubrum lacusprofundi ATCC 49239 chromosome 1 | 61 |
| Lactobacillus casei ATCC 334 | 61 |
| Candidatus Puniceispirillum marinum IMCC1322 chromosome | 60 |
| Synechococcus sp. PCC 7002 chromosome | 60 |
| Rubrobacter xylanophilus DSM 9941 | 60 |
| Lactococcus lactis subsp. lactis Il1403 | 60 |
| Chlorobium phaeobacteroides DSM 266 | 60 |
| Methanocella paludicola SANAE chromosome | 60 |
| Streptococcus agalactiae NEM316 | 60 |
| Azospirillum sp. B510 chromosome | 60 |
| Sulfurospirillum deleyianum DSM 6946 | 60 |
| Rhizobium sp. NGR234 plasmid pNGR234b | 60 |
| Staphylococcus aureus subsp. aureus JH1 | 59 |
| Staphylococcus haemolyticus JCSC1435 | 59 |
| Sulfolobus tokodaii str. 7 chromosome | 59 |
| Burkholderia vietnamiensis G4 chromosome 2 | 59 |
| Cupriavidus taiwanensis str. LMG19424 chromosome 2 | 59 |
| Caldicelulosiruptor becscii DSM 6725 chromosome | 59 |
| Burkholderia cenocepacia AU 1054 chromosome 2 | 59 |
| Sulfolobus islandicus M.14.25 chromosome | 59 |
| Listeria innocua Clip11262 | 58 |
| Burkholderia cenocepacia HI2424 chromosome 2 | 58 |
| Corynebacterium diphtheriae NCTC 13129 | 58 |
| Kocuria rhizophila DC2201 | 58 |
| Ruegeria sp. TM1040 | 58 |
| Chlorobium limicola DSM 245 | 58 |
| Staphylococcus aureus subsp. aureus COL | 58 |
| Anoxybacillus flavithermus WK1 | 58 |
| Agrobacterium radiobacter K84 chromosome 2 | 58 |
| Staphylococcus aureus subsp. aureus Mu3 | 57 |
| Thiomonas intermedia K12 chromosome | 57 |
| Sulfolobus islandicus Y.N.15.51 chromosome | 57 |
| Brucella ovis ATCC 25840 chromosome I | 57 |
| Vibrio fischeri MJ11 chromosome I | 57 |
| Treponema denticola ATCC 35405 chromosome | 57 |
| Xylella fastidiosa 9a5c | 57 |
| Jonesia denitrificans DSM 20603 | 56 |
| Moorella thermoacetica ATCC 39073 | 56 |
| Vibrio cholerae O1 biovar El Tor str. N16961 chromosome I | 56 |
| Brucella canis ATCC 23365 chromosome I | 56 |
| Pyrobaculum aerophilum str. IM2 chromosome | 56 |
| Staphylococcus epidermidis ATCC 12228 | 56 |
| Neisseria meningitidis MC58 | 56 |
| Thiobacillus denitrificans ATCC 25259 | 56 |
| Staphylococcus aureus subsp. aureus Mu50 | 56 |
| Sulfolobus islandicus L.D.8.5 chromosome | 56 |
| Burkholderia phymatum STM815 chromosome 2 | 56 |
| Lactococcus lactis subsp. cremoris MG1363 | 56 |
| Streptococcus pneumoniae R6 | 56 |
| Burkholderia phymatum STM815 chromosome 1 | 56 |
| Prosthecochloris aestuarii DSM 271 | 56 |
| Cupriavidus metallidurans CH34 megaplasmid | 55 |
| Prochlorococcus marinus str. MIT 9303 | 55 |
| Thermoanaerobacter pseudethanolicus ATCC 33223 | 55 |
| Listeria welshimeri serovar 6b str. SLCC5334 | 55 |
| Leuconostoc mesenteroides subsp. mesenteroides ATCC 8293 | 55 |
| Prochlorococcus marinus str. MIT 9313 chromosome | 55 |
| Staphylococcus aureus subsp. aureus USA300_FPR3757 | 55 |
| Capnocytophaga ochracea DSM 7271 | 55 |
| Actinobacillus succinogenes 130Z | 55 |
| Granulibacter bethesdensis CGDNIH1 chromosome | 55 |
| Staphylococcus aureus subsp. aureus USA300_TCH1516 chromosome | 54 |
| Neisseria meningitidis FAM18 | 54 |
| Photobacterium profundum SS9 chromosome 2 | 54 |
| Nitrosomonas europaea ATCC 19718 | 54 |
| Staphylococcus aureus subsp. aureus NCTC 8325 | 54 |
| Methanoculleus marisnigri JR1 chromosome | 54 |
| Haloferax volcanii DS2 chromosome | 53 |
| Burkholderia pseudomallei K96243 chromosome 2 | 53 |
| Burkholderia mallei NCTC 10247 chromosome II | 53 |
| Porphyromonas gingivalis ATCC 33277 | 53 |
| Chlorobium tepidum TLS | 53 |
| Lactococcus lactis subsp. cremoris SK11 | 53 |
| Synechococcus sp. CC9311 | 53 |
| Staphylococcus aureus RF122 | 53 |
| Corynebacterium urealyticum DSM 7109 | 53 |
| Chlorobium chlorochromatii CaD3 | 53 |
| Sulfolobus acidocaldarius DSM 639 chromosome | 53 |
| Bifidobacterium longum DJO10A | 53 |
| Kytococcus sedentarius DSM 20547 | 53 |
| Clostridium novyi NT | 52 |
| Paracoccus denitrificans PD1222 chromosome 1 | 52 |
| Methanopyrus kandleri AV19 | 52 |
| Desulfohalobium retbaense DSM 5692 | 52 |
| Wolinella succinogenes DSM 1740 chromosome | 52 |
| Orientia tsutsugamushi Boryong | 52 |
| Ralstonia eutropha JMP134 chromosome 2 | 52 |
| Campylobacter concisus 13826 | 52 |
| Actinobacillus pleuropneumoniae L20 | 52 |
| Francisella tularensis subsp. mediasiatica FSC147 | 52 |
| Pasteurella multocida subsp. multocida str. Pm70 | 52 |
| Synechococcus sp. RCC307 | 51 |
| Sulfolobus islandicus L.S.2.15 chromosome | 51 |
| Synechococcus elongatus PCC 6301 | 51 |
| Staphylococcus epidermidis RP62A | 51 |
| Archaeoglobus fulgidus DSM 4304 | 51 |
| Candidatus Desulforudis audaxviator MP104C | 51 |
| Halorhodospira halophila SL1 | 51 |
| Ammonifex degensii KC4 | 51 |
| Staphylococcus aureus subsp. aureus MSSA476 chromosome | 51 |
| Streptococcus sanguinis SK36 | 51 |
| Ralstonia eutropha H16 chromosome 2 | 51 |
| Lactobacillus plantarum WCFS1 | 51 |
| Pelotomaculum thermopropionicum SI chromosome | 51 |
| Thermoanaerobacter tengcongensis MB4 | 51 |
| Corynebacterium jeikeium K411 | 51 |
| Xylella fastidiosa M23 | 51 |
| Bifidobacterium dentium Bd1 | 50 |
| Burkholderia mallei ATCC 23344 chromosome 2 | 50 |
| Anaerococcus prevotii DSM 20548 | 50 |
| Bartonella tribocorum CIP 105476 | 50 |
| Streptococcus equi subsp. zooepidemicus | 50 |
| Mycobacterium leprae TN chromosome | 50 |
| Neisseria meningitidis 053442 | 50 |
| Candidatus Methanoregula boonei 6A8 chromosome | 50 |
| Ochrobactrum anthropi ATCC 49188 chromosome 1 | 50 |
| Veillonella parvula DSM 2008 | 50 |
| Coxiella burnetii CbuG_Q212 chromosome | 50 |
| Francisella tularensis subsp. holarctica | 50 |
| Agrobacterium tumefaciens str. C58 chromosome linear | 50 |
| Dictyoglomus turgidum DSM 6724 | 50 |
| Deinococcus geothermalis DSM 11300 | 49 |
| Streptococcus agalactiae A909 | 49 |
| Kosmotoga olearia TBF 19.5.1 | 49 |
| Actinobacillus pleuropneumoniae serovar 3 str. JL03 | 49 |
| Synechococcus elongatus PCC 7942 chromosome | 49 |
| Staphylococcus saprophyticus subsp. saprophyticus ATCC 15305 | 49 |
| Mannheimia succiniciproducens MBEL55E | 49 |
| Sulfurihydrogenibium sp. YO3AOP1 | 49 |
| Idiomarina loihiensis L2TR | 49 |
| Streptococcus pneumoniae CGSP14 | 49 |
| Synechococcus sp. WH 7803 | 48 |
| Streptococcus pneumoniae D39 | 48 |
| Deinococcus radiodurans R1 chromosome 1 | 48 |
| Arcobacter butzleri RM4018 | 48 |
| Thermoanaerobacter sp. X514 | 48 |
| Thermotoga lettingae TMO | 48 |
| Thermosipho melanesiensis BI429 | 48 |
| Synechococcus sp. CC9902 chromosome | 48 |
| Agrobacterium tumefaciens str. C58 chromosome circular | 48 |
| Synechococcus sp. CC9605 | 48 |
| Lactobacillus gasseri ATCC 33323 | 48 |
| Synechococcus sp. WH 8102 | 48 |
| Chlorobaculum parvum NCIB 8327 | 48 |
| Prochlorococcus marinus str. MIT 9312 | 48 |
| Candidatus Protochlamydia amoebophila UWE25 | 48 |
| Chlorobium phaeobacteroides BS1 | 47 |
| Acidothermus cellulolyticus 11B | 47 |
| Streptococcus pyogenes MGAS10270 chromosome | 47 |
| Burkholderia mallei NCTC 10229 chromosome II | 47 |
| Haemophilus influenzae PittGG | 47 |
| Pyrobaculum arsenaticum DSM 13514 | 47 |
| Petrotoga mobilis SJ95 | 47 |
| Streptococcus pyogenes MGAS10750 chromosome | 47 |
| Streptococcus pyogenes MGAS8232 | 47 |
| Burkholderia thailandensis E264 chromosome II | 47 |
| Sulfolobus islandicus Y.G.57.14 chromosome | 47 |
| Sulfolobus islandicus M.16.27 chromosome | 47 |
| Zymomonas mobilis subsp. mobilis ZM4 chromosome | 46 |
| Lactobacillus fermentum IFO 3956 | 46 |
| Lactobacillus johnsonii NCC 533 | 46 |
| Brucella melitensis bv. 1 str. 16M chromosome chromosome I | 46 |
| Streptococcus mutans UA159 chromosome | 46 |
| Burkholderia cepacia AMMD chromosome 2 | 46 |
| Prochlorococcus marinus str. MIT 9215 | 46 |
| Pediococcus pentosaceus ATCC 25745 | 46 |
| Methanococcus maripaludis C5 | 45 |
| Haemophilus somnus 2336 | 45 |
| Staphylococcus aureus subsp. aureus N315 | 45 |
| Aquifex aeolicus VF5 | 45 |
| Streptococcus pyogenes str. Manfredo | 45 |
| Ralstonia solanacearum GMI1000 plasmid pGMI1000MP | 45 |
| Pyrobaculum calidifontis JCM 11548 chromosome | 45 |
| Streptococcus mitis B6 | 44 |
| Bifidobacterium adolescentis ATCC 15703 chromosome | 44 |
| Eubacterium eligens ATCC 27750 | 44 |
| Acidimicrobium ferrooxidans DSM 10331 | 44 |
| Pyrococcus horikoshii OT3 | 44 |
| Methanosphaera stadtmanae DSM 3091 chromosome | 44 |
| Prochlorococcus marinus str. MIT 9301 | 44 |
| Coxiella burnetii RSA 331 chromosome | 44 |
| Leifsonia xyli subsp. xyli str. CTCB07 | 44 |
| Candidatus Amoebophilus asiaticus 5a2 chromosome | 44 |
| Methanococcoides burtonii DSM 6242 | 44 |
| Streptococcus pneumoniae 70585 | 44 |
| Thermodesulfovibrio yellowstonii DSM 11347 | 43 |
| Francisella tularensis subsp. tularensis FSC198 | 43 |
| Streptococcus uberis 0140J chromosome | 43 |
| Xylella fastidiosa Temecula1 | 43 |
| Finegoldia magna ATCC 29328 | 43 |
| Brucella melitensis biovar Abortus 2308 chromosome I | 42 |
| Brucella suis ATCC 23445 chromosome I | 42 |
| Brucella abortus S19 chromosome 1 | 42 |
| Streptococcus pyogenes MGAS6180 chromosome | 42 |
| Streptococcus suis 05ZYH33 | 42 |
| Sulfurimonas denitrificans DSM 1251 | 42 |
| Fusobacterium nucleatum subsp. nucleatum ATCC 25586 chromosome | 42 |
| Micrococcus luteus NCTC 2665 | 42 |
| Burkholderia multivorans ATCC 17616 chromosome 3 | 42 |
| Sulfolobus islandicus M.16.4 chromosome | 42 |
| Methanosaeta thermophila PT chromosome | 42 |
| Methanothermobacter thermautotrophicus str. Delta H chromosome | 41 |
| Streptococcus gordonii str. Challis substr. CH1 | 41 |
| Ochrobactrum anthropi ATCC 49188 chromosome 2 | 41 |
| Thermococcus onnurineus NA1 | 41 |
| Thermococcus kodakarensis KOD1 | 41 |
| Streptococcus pneumoniae G54 chromosome | 41 |
| Candidatus Korarchaeum cryptofilum OPF8 chromosome | 41 |
| Chlorobium luteolum DSM 273 | 41 |
| Burkholderia mallei SAVP1 chromosome II | 41 |
| Francisella tularensis subsp. novicida U112 | 40 |
| Bifidobacterium longum NCC2705 chromosome | 40 |
| Porphyromonas gingivalis W83 chromosome | 40 |
| Francisella tularensis subsp. holarctica OSU18 | 40 |
| Prosthecochloris vibrioformis DSM 265 | 40 |
| Methanocorpusculum labreanum Z chromosome | 40 |
| Campylobacter curvus 525.92 | 40 |
| Streptococcus pyogenes M1 GAS chromosome | 40 |
| Lactobacillus delbrueckii subsp. bulgaricus ATCC 11842 | 40 |
| Vibrio vulnificus CMCP6 chromosome II | 40 |
| Lactobacillus acidophilus NCFM chromosome | 39 |
| Coxiella burnetii CbuK_Q154 chromosome | 39 |
| Helicobacter pylori Shi470 | 39 |
| Sinorhizobium medicae WSM419 plasmid pSMED01 | 39 |
| Prochlorococcus marinus subsp. marinus str. CCMP1375 | 39 |
| Francisella tularensis subsp. tularensis SCHU S4 | 39 |
| Thermotoga sp. RQ2 | 39 |
| Haemophilus somnus 129PT | 39 |
| Neisseria gonorrhoeae NCCP11945 chromosome | 39 |
| Neisseria gonorrhoeae FA 1090 | 39 |
| Thermoproteus neutrophilus V24Sta chromosome | 39 |
| Rickettsia rickettsii str. Iowa chromosome | 38 |
| Thermanaerovibrio acidaminovorans DSM 6589 | 38 |
| Thermosipho africanus TCF52B | 38 |
| Rickettsia felis URRWXCal2 | 38 |
| Thermomicrobium roseum DSM 5159 | 38 |
| Neisseria meningitidis Z2491 | 38 |
| Streptococcus pyogenes MGAS5005 chromosome | 38 |
| Haemophilus ducreyi 35000HP | 38 |
| Halobacterium salinarum R1 | 37 |
| Bifidobacterium animalis subsp. lactis AD011 | 37 |
| Metallosphaera sedula DSM 5348 chromosome | 37 |
| Bartonella quintana str. Toulouse | 37 |
| Pyrococcus abyssi GE5 chromosome | 37 |
| Prochlorococcus marinus str. MIT 9515 | 37 |
| Brucella suis 1330 chromosome II | 37 |
| Streptococcus thermophilus CNRZ1066 chromosome | 37 |
| Thermofilum pendens Hrk 5 chromosome | 37 |
| Lactobacillus salivarius UCC118 | 36 |
| Pyrococcus furiosus DSM 3638 | 36 |
| Streptococcus pyogenes MGAS315 | 36 |
| Leuconostoc citreum KM20 | 36 |
| Thermoplasma acidophilum DSM 1728 chromosome | 36 |
| Thermus thermophilus HB8 | 36 |
| Campylobacter jejuni subsp. doylei 269.97 | 36 |
| Streptococcus pneumoniae TIGR4 chromosome | 36 |
| Campylobacter jejuni RM1221 | 36 |
| Streptococcus suis 98HAH33 | 36 |
| Vibrio vulnificus YJ016 chromosome II | 36 |
| Brucella suis 1330 chromosome I | 35 |
| Ehrlichia chaffeensis str. Arkansas | 35 |
| Thermus thermophilus HB27 | 35 |
| Burkholderia phymatum STM815 plasmid pBPHY01 | 35 |
| Prochlorococcus marinus str. AS9601 | 35 |
| Thermoplasma volcanium GSS1 chromosome | 35 |
| Methanothermobacter marburgensis str. Marburg chromosome | 35 |
| Coxiella burnetii RSA 493 | 35 |
| Haemophilus influenzae PittEE | 35 |
| Helicobacter hepaticus ATCC 51449 chromosome | 35 |
| Streptococcus pyogenes MGAS10394 chromosome | 35 |
| Streptococcus pneumoniae JJA | 35 |
| Streptococcus pyogenes MGAS2096 chromosome | 35 |
| Lactobacillus helveticus DPC 4571 | 34 |
| Lactobacillus sakei subsp. sakei 23K | 34 |
| Streptococcus mutans NN2025 | 34 |
| Pyrobaculum islandicum DSM 4184 chromosome | 34 |
| Ehrlichia canis str. Jake | 34 |
| Methanococcus maripaludis C7 | 34 |
| Helicobacter pylori J99 | 34 |
| Francisella philomiragia subsp. philomiragia ATCC 25017 | 33 |
| Anaplasma phagocytophilum HZ | 33 |
| Macrococcus caseolyticus JCSC5402 | 33 |
| Aeropyrum pernix K1 | 33 |
| Coprothermobacter proteolyticus DSM 5265 | 33 |
| Paracoccus denitrificans PD1222 chromosome 2 | 33 |
| Ehrlichia ruminantium str. Gardel | 33 |
| Thermotoga maritima MSB8 chromosome | 33 |
| Prochlorococcus marinus subsp. pastoris str. CCMP1986 | 33 |
| Orientia tsutsugamushi str. Ikeda | 33 |
| Nitrosopumilus maritimus SCM1 chromosome | 32 |
| Ehrlichia ruminantium str. Welgevonden | 32 |
| Candidatus Pelagibacter ubique HTCC1062 chromosome | 32 |
| Methylacidiphilum infernorum V4 | 32 |
| Prochlorococcus marinus str. NATL2A | 32 |
| Prochlorococcus marinus str. MIT 9211 | 31 |
| Treponema pallidum subsp. pallidum str. Nichols chromosome | 31 |
| Campylobacter jejuni subsp. jejuni 81116 | 31 |
| Hydrogenobaculum sp. Y04AAS1 | 31 |
| Dehalococcoides sp. BAV1 | 31 |
| Streptococcus pyogenes MGAS9429 chromosome | 31 |
| Vibrio fischeri ES114 chromosome chromosome II | 31 |
| Haemophilus influenzae Rd KW20 | 31 |
| Streptococcus pyogenes NZ131 chromosome | 31 |
| Vibrio sp. Ex25 chromosome 2 | 31 |
| Ralstonia pickettii 12D chromosome 2 | 31 |
| Methanococcus maripaludis S2 | 31 |
| Methanocaldococcus jannaschii DSM 2661 chromosome | 30 |
| Campylobacter lari RM2100 | 30 |
| Prochlorococcus marinus str. NATL1A | 30 |
| Burkholderia xenovorans LB400 chromosome 3 | 30 |
| Sinorhizobium meliloti 1021 plasmid pSymA | 30 |
| Cryptobacterium curtum DSM 15641 | 30 |
| Staphylothermus marinus F1 chromosome | 30 |
| Thermotoga neapolitana DSM 4359 | 30 |
| Vibrio parahaemolyticus RIMD 2210633 chromosome 2 | 30 |
| Dichelobacter nodosus VCS1703A | 29 |
| Agrobacterium vitis S4 chromosome 2 | 29 |
| Bartonella bacilliformis KC583 | 29 |
| Streptococcus thermophilus LMG 18311 chromosome | 29 |
| Campylobacter jejuni subsp. jejuni NCTC 11168 chromosome | 29 |
| Burkholderia cepacia AMMD chromosome 3 | 29 |
| Azospirillum sp. B510 plasmid pAB510a | 29 |
| Methanococcus vannielii SB | 29 |
| Brucella canis ATCC 23365 chromosome II | 28 |
| Rickettsia massiliae MTU5 | 28 |
| Helicobacter acinonychis str. Sheeba chromosome | 28 |
| Vibrio cholerae O395 chromosome 1 | 28 |
| Aciduliprofundum boonei T469 chromosome | 28 |
| Chlamydophila pneumoniae J138 | 28 |
| Dehalococcoides ethenogenes 195 | 28 |
| Sinorhizobium meliloti 1021 plasmid pSymB | 28 |
| Helicobacter pylori HPAG1 | 28 |
| Methanococcus voltae A3 chromosome | 28 |
| Sinorhizobium medicae WSM419 plasmid pSMED02 | 27 |
| Desulfurococcus kamchatkensis 1221n chromosome | 27 |
| Atopobium parvulum DSM 20469 | 27 |
| Anaplasma marginale str. Florida | 27 |
| Rickettsia prowazekii str. Madrid E chromosome | 26 |
| Rickettsia canadensis str. McKiel | 26 |
| Rhizobium etli CIAT 652 plasmid pC | 26 |
| Methanococcus maripaludis C6 | 26 |
| Chlamydia muridarum Nigg | 26 |
| Burkholderia cenocepacia AU 1054 chromosome 3 | 26 |
| Picrophilus torridus DSM 9790 chromosome | 26 |
| Polynucleobacter necessarius subsp. necessarius STIR1 | 26 |
| Helicobacter pylori 26695 | 26 |
| Aliivibrio salmonicida LFI1238 chromosome 2 | 26 |
| Rhizobium leguminosarum bv. viciae 3841 plasmid pRL12 | 26 |
| Rickettsia conorii str. Malish 7 | 25 |
| Wolbachia endosymbiont strain TRS of Brugia malayi | 25 |
| Brucella melitensis biovar Abortus 2308 chromosome II | 25 |
| Brucella melitensis bv. 1 str. 16M chromosome chromosome II | 25 |
| Brucella ovis ATCC 25840 chromosome II | 25 |
| Streptobacillus moniliformis DSM 12112 | 24 |
| Chlamydophila pneumoniae AR39 | 24 |
| Rickettsia typhi str. Wilmington | 24 |
| Dehalococcoides sp. VS chromosome | 24 |
| Brucella abortus S19 chromosome 2 | 24 |
| Elusimicrobium minutum Pei191 | 24 |
| Hyperthermus butylicus DSM 5456 chromosome | 24 |
| Burkholderia cenocepacia J2315 chromosome chromosome 3 | 24 |
| Burkholderia sp. 383 chromosome 3 | 24 |
| Wolbachia endosymbiont of Drosophila melanogaster | 23 |
| Cyanothece sp. PCC 7822 plasmid Cy782201 | 23 |
| Methanobrevibacter smithii ATCC 35061 chromosome | 23 |
| Wolbachia sp. wRi | 23 |
| Burkholderia cenocepacia HI2424 chromosome 3 | 23 |
| Chlamydophila caviae GPIC | 23 |
| Treponema pallidum subsp. pallidum SS14 | 23 |
| Ralstonia pickettii 12J chromosome 2 | 22 |
| Ruegeria sp. TM1040 mega plasmid | 22 |
| Candidatus Phytoplasma mali | 22 |
| Mycoplasma mycoides subsp. mycoides SC str. PG1 chromosome | 22 |
| Borrelia burgdorferi ZS7 | 22 |
| Rhodobacter sphaeroides ATCC 17029 chromosome chromosome 2 | 22 |
| Mycoplasma crocodyli MP145 chromosome | 22 |
| Candidatus Vesicomyosocius okutanii HA | 22 |
| Methylobacterium extorquens AM1 megaplasmid | 22 |
| Ehrlichia ruminantium str. Welgevonden chromosome | 22 |
| Borrelia turicatae 91E135 chromosome | 22 |
| Rhodococcus jostii RHA1 plasmid pRHL1 | 21 |
| Rhizobium leguminosarum bv. trifolii WSM2304 plasmid pRLG201 | 21 |
| Candidatus Phytoplasma australiense | 21 |
| Borrelia duttonii Ly | 21 |
| Brucella suis ATCC 23445 chromosome II | 21 |
| Burkholderia phymatum STM815 plasmid pBPHY02 | 21 |
| Sphaerobacter thermophilus DSM 20745 chromosome 2 | 21 |
| Neorickettsia risticii str. Illinois | 21 |
| Rickettsia akari str. Hartford | 21 |
| Mycoplasma hyopneumoniae 7448 | 20 |
| Tropheryma whipplei str. Twist | 20 |
| Mycoplasma mobile 163K | 20 |
| Rhizobium leguminosarum bv. trifolii WSM1325 plasmid pR132501 | 20 |
| Ureaplasma parvum serovar 3 str. ATCC 27815 chromosome | 20 |
| Chlamydophila pneumoniae CWL029 | 20 |
| Borrelia hermsii DAH chromosome | 20 |
| Rhodobacter sphaeroides 2.4.1 chromosome 2 | 20 |
| Azospirillum sp. B510 plasmid pAB510c | 19 |
| Borrelia recurrentis A1 | 19 |
| Dehalococcoides sp. CBDB1 chromosome | 19 |
| Methylobacterium nodulans ORS 2060 plasmid pMNOD01 | 19 |
| Mesoplasma florum L1 | 19 |
| Burkholderia vietnamiensis G4 chromosome 3 | 18 |
| Anaplasma marginale str. St. Maries | 18 |
| Mycoplasma hyopneumoniae 232 | 18 |
| Vibrio cholerae O1 biovar eltor str. N16961 chromosome II | 18 |
| Mycoplasma pneumoniae M129 | 17 |
| NC_001318 Borrelia burgdorferi B31 chromosome | 17 |
| Borrelia garinii PBi chromosome chromosome linear | 17 |
| Thermomicrobium roseum DSM 5159 plasmid unnamed | 17 |
| Candidatus Blochmannia pennsylvanicus str. BPEN | 16 |
| Eubacterium eligens ATCC 27750 plasmid unnamed | 16 |
| Azospirillum sp. B510 plasmid pAB510b | 16 |
| Candidatus Riesia pediculicola USDA chromosome | 16 |
| Vibrio fischeri MJ11 chromosome II | 16 |
| Mycoplasma genitalium G37 | 16 |
| Borrelia afzelii PKo | 16 |
| Methylobacterium radiotolerans JCM 2831 plasmid pMRAD01 | 15 |
| Neorickettsia sennetsu str. Miyayama | 15 |
| Deinococcus geothermalis DSM 11300 plasmid pDGEO01 | 15 |
| Rhodobacter sphaeroides ATCC 17025 plasmid pRSPA01 | 15 |
| Rhizobium leguminosarum bv. viciae 3841 plasmid pRL10 | 15 |
| Rhizobium etli CFN 42 plasmid p42f | 15 |
| Ralstonia eutropha JMP134 megaplasmid | 15 |
| Mycoplasma hyopneumoniae J | 14 |
| Candidatus Blochmannia floridanus chromosome | 14 |
| Rhizobium leguminosarum bv. trifolii WSM1325 plasmid pR132502 | 14 |
| Mycoplasma capricolum subsp. capricolum ATCC 27343 | 14 |
| Mycoplasma pulmonis UAB CTIP | 14 |
| Cupriavidus necator megaplasmid pHG1 | 14 |
| Agrobacterium vitis S4 plasmid pAtS4e | 14 |
| Rhizobium sp. NGR234 plasmid pNGR234a | 14 |
| Deinococcus radiodurans R1 chromosome 2 | 14 |
| Phenylobacterium zucineum HLK1 plasmid unnamed | 13 |
| Anabaena variabilis ATCC 29413 plasmid A | 13 |
| Rhizobium leguminosarum bv. viciae 3841 plasmid pRL11 | 13 |
| Halorubrum lacusprofundi ATCC 49239 chromosome 2 | 13 |
| Arthrobacter chlorophenolicus A6 plasmid pACHL01 | 12 |
| Wigglesworthia glossinidia endosymbiont of Glossina brevipalpis chromosome | 12 |
| Rhizobium etli CFN 42 plasmid p42e | 12 |
| Herpetosiphon aurantiacus ATCC 23779 plasmid pHAU01 | 12 |
| Mycoplasma agalactiae PG2 | 12 |
| Azospirillum sp. B510 plasmid pAB510e | 11 |
| Mesorhizobium sp. BNC1 plasmid 1 | 11 |
| Methylobacterium chloromethanicum CM4 plasmid pMCHL01 | 11 |
| Rhizobium leguminosarum bv. trifolii WSM2304 plasmid pRLG202 | 11 |
| Haloferax volcanii DS2 plasmid pHV4 | 11 |
| Rhodococcus erythropolis PR4 plasmid pREL1 | 11 |
| Novosphingobium aromaticivorans DSM 12444 plasmid pNL2 | 11 |
| Methylibium petroleiphilum PM1 plasmid RPME01 | 11 |
| Azospirillum sp. B510 plasmid pAB510d | 11 |
| Mycoplasma synoviae 53 | 11 |
| Blattabacterium sp. (Blattella germanica) str. Bge | 11 |
| Rhizobium leguminosarum bv. trifolii WSM1325 plasmid pR132503 | 11 |
| Methylobacterium nodulans ORS 2060 plasmid pMNOD02 | 11 |
| Agrobacterium radiobacter K84 plasmid pAtK84c | 10 |
| Mycobacterium sp. KMS plasmid pMKMS01 | 10 |
| Agrobacterium tumefaciens str. C58 plasmid At | 10 |
| Cupriavidus taiwanensis plasmid pRALTA | 10 |
| Paracoccus denitrificans PD1222 plasmid 1 | 10 |
| Sphingomonas wittichii RW1 plasmid pSWIT01 | 10 |
| Anabaena variabilis ATCC 29413 plasmid C | 10 |
| Nocardiopsis dassonvillei subsp. dassonvillei DSM 43111 plasmid pNDAS01 | 10 |
| Rhodococcus jostii RHA1 plasmid pRHL2 | 10 |
| Haloarcula marismortui ATCC 43049 plasmid pNG700 | 9 |
| Haloarcula marismortui ATCC 43049 chromosome II | 9 |
| Nostoc punctiforme PCC 73102 plasmid pNPUN01 | 9 |
| Thermus thermophilus HB8 plasmid pTT27 | 9 |
| Ureaplasma parvum serovar 3 str. ATCC 700970 | 9 |
| Bradyrhizobium sp. BTAi1 plasmid pBBta01 | 9 |
| Bacillus cereus E33L plasmid pE33L466 | 9 |
| Xanthobacter autotrophicus Py2 plasmid pXAUT01 | 9 |
| Rhizobium leguminosarum bv. trifolii WSM2304 plasmid pRLG203 | 9 |
| Azospirillum sp. B510 plasmid pAB510f | 9 |
| Rhodobacter sphaeroides ATCC 17025 plasmid pRSPA02 | 9 |
| Haloferax volcanii DS2 plasmid pHV3 | 8 |
| Halorubrum lacusprofundi ATCC 49239 plasmid pHLAC01 | 8 |
| Cyanothece sp. PCC 7822 plasmid Cy782202 | 8 |
| Polaromonas sp. JS666 plasmid 2 | 8 |
| Arthrobacter aurescens TC1 plasmid TC1 | 8 |
| Agrobacterium vitis S4 plasmid pTiS4 | 8 |
| Rhizobium etli CIAT 652 plasmid pA | 8 |
| Acaryochloris marina MBIC11017 plasmid pREB7 | 8 |
| Novosphingobium aromaticivorans DSM 12444 plasmid pNL1 | 8 |
| Ralstonia pickettii 12D plasmid pRp12D01 | 8 |
| Bacillus pseudofirmus OF4 plasmid pBpOF4-01 | 8 |
| Rhodococcus jostii RHA1 plasmid pRHL3 | 8 |
| Bacillus cereus AH820 plasmid pAH820_272 | 8 |
| Polaromonas naphthalenivorans CJ2 plasmid pPNAP01 | 8 |
| Enterobacter sp. 638 plasmid pENTE01 | 8 |
| Clostridium botulinum Ba4 str. 657 plasmid pCLJ | 7 |
| Cyanothece sp. PCC 7425 plasmid pP742501 | 7 |
| Marinobacter aquaeolei VT8 plasmid pMAQU01 | 7 |
| Salmonella enterica subsp. enterica serovar Typhi str. CT18 plasmid pHCM1 | 7 |
| Cupriavidus metallidurans CH34 plasmid pMOL30 | 7 |
| Burkholderia vietnamiensis G4 plasmid pBVIE01 | 7 |
| Acaryochloris marina MBIC11017 plasmid pREB2 | 7 |
| Bacillus cereus ATCC 10987 plasmid pBc10987 | 7 |
| Shigella flexneri 2a str. 301 plasmid pCP301 | 7 |
| Cyanothece sp. PCC 7425 plasmid pP742502 | 7 |
| Meiothermus silvanus DSM 9946 plasmid pMESIL01 | 6 |
| Mesorhizobium loti MAFF303099 plasmid pMLa | 6 |
| Ochrobactrum anthropi ATCC 49188 plasmid pOANT01 | 6 |
| Nocardioides sp. JS614 plasmid pNOCA01 | 6 |
| Lactobacillus salivarius UCC118 plasmid pMP118 | 6 |
| Rhizobium leguminosarum bv. trifolii WSM1325 plasmid pR132505 | 6 |
| Nostoc punctiforme PCC 73102 plasmid pNPUN02 | 6 |
| Borrelia garinii pBi plasmid | 6 |
| Deinococcus geothermalis DSM 11300 plasmid pDGEO02 | 6 |
| Cyanothece sp. PCC 7424 plasmid pP742401 | 6 |
| Escherichia coli APEC O1 plasmid pAPEC-O1-R | 6 |
| Arthrobacter aurescens TC1 plasmid TC2 | 6 |
| Agrobacterium tumefaciens str. C58 plasmid Ti | 6 |
| Cyanothece sp. PCC 7822 plasmid Cy782203 | 6 |
| Pseudoalteromonas haloplanktis TAC125 chromosome II | 6 |
| Rhizobium leguminosarum bv. viciae 3841 plasmid pRL9 | 6 |
| Candidatus Sulcia muelleri GWSS | 6 |
| Nostoc sp. PCC 7120 plasmid pCC7120alpha | 6 |
| Butyrivibrio proteoclasticus B316 plasmid pCY360 | 6 |
| Nitrobacter hamburgensis X14 plasmid 1 | 6 |
| Desulfovibrio vulgaris str. Hildenborough plasmid pDV | 5 |
| Rhizobium etli CFN 42 plasmid p42b | 5 |
| Burkholderia vietnamiensis G4 plasmid pBVIE02 | 5 |
| Rhizobium etli CFN 42 symbiotic plasmid p42d | 5 |
| Candidatus Carsonella ruddii PV | 5 |
| Bacillus weihenstephanensis KBAB4 plasmid pBWB401 | 5 |
| Bacillus cereus G9842 plasmid pG9842_209 | 5 |
| Acaryochloris marina MBIC11017 plasmid pREB3 | 5 |
| Arthrobacter sp. FB24 plasmid 2 | 5 |
| Klebsiella pneumoniae 342 plasmid pKP187 | 5 |
| Azoarcus sp. EbN1 plasmid 1 | 5 |
| Mycobacterium ulcerans AGY99 plasmid pMUM001 | 5 |
| Alicyclobacillus acidocaldarius subsp. acidocaldarius DSM 446 plasmid pAACI02 | 5 |
| Spirosoma linguale DSM 74 plasmid pSLIN02 | 5 |
| Polaromonas sp. JS666 plasmid 1 | 5 |
| Rhizobium etli CIAT 652 plasmid pB | 5 |
| Borrelia duttonii Ly plasmid pl70 | 5 |
| Synechocystis sp. PCC 6803 plasmid pSYSM | 5 |
| Nostoc sp. PCC 7120 plasmid pCC7120beta | 5 |
| Acaryochloris marina MBIC11017 plasmid pREB1 | 5 |
| Pseudomonas syringae pv. phaseolicola 1448A large plasmid | 5 |
| Nitrobacter hamburgensis X14 plasmid 2 | 5 |
| Cyanothece sp. ATCC 51142 chromosome chromosome linear | 5 |
| Agrobacterium vitis S4 plasmid pAtS4c | 5 |
| Agrobacterium radiobacter K84 plasmid pAtK84b | 5 |
| Roseobacter denitrificans plasmid pTB1 | 4 |
| Yersinia pestis CO92 plasmid pMT1 | 4 |
| Dinoroseobacter shibae DFL 12 plasmid pDSHI01 | 4 |
| Escherichia coli APEC O1 plasmid pAPEC-O1-ColBM | 4 |
| Arthrobacter sp. FB24 plasmid 1 | 4 |
| Geobacter lovleyi SZ plasmid pGLOV01 | 4 |
| Escherichia coli E24377A plasmid pETEC_74 | 4 |
| Clostridium botulinum A3 str. Loch Maree plasmid pCLK | 4 |
| Meiothermus silvanus DSM 9946 plasmid pMESIL02 | 4 |
| Ralstonia pickettii 12D plasmid pRp12D02 | 4 |
| Rhizobium leguminosarum bv. viciae 3841 plasmid pRL8 | 4 |
| Halomicrobium mukohataei DSM 12286 plasmid pHmuk01 | 4 |
| Synechocystis sp. PCC 6803 plasmid pSYSX | 4 |
| Ochrobactrum anthropi ATCC 49188 plasmid pOANT03 | 4 |
| Haloferax volcanii DS2 plasmid pHV1 | 4 |
| Sebaldella termitidis ATCC 33386 plasmid pSTERM01 | 4 |
| Bacillus cereus Q1 plasmid pBc239 | 4 |
| Methanosarcina barkeri str. fusaro plasmid 1 | 4 |
| Sphingomonas wittichii RW1 plasmid pSWIT02 | 4 |
| Yersinia pseudotuberculosis IP 32953 plasmid pYV | 4 |
| Bacillus cereus AH187 plasmid pAH187_270 | 4 |
| Vibrio fischeri MJ11 plasmid pMJ100 | 4 |
| Burkholderia vietnamiensis G4 plasmid pBVIE04 | 4 |
| Burkholderia vietnamiensis G4 plasmid pBVIE03 | 4 |
| Alicyclobacillus acidocaldarius subsp. acidocaldarius DSM 446 plasmid pAACI01 | 4 |
| Rhizobium etli CFN 42 plasmid p42c | 4 |
| Borrelia afzelii PKo plasmid lp60 | 4 |
| Rhodobacter sphaeroides ATCC 17025 plasmid pRSPA04 | 4 |
| Caulobacter sp. K31 plasmid pCAUL01 | 4 |
| Rhodobacter sphaeroides ATCC 17029 plasmid pRSPH01 | 4 |
| Clostridium tetani E88 plasmid pE88 | 4 |
| Rhodococcus erythropolis PR4 plasmid pREC1 | 4 |
| Synechococcus sp. PCC 7002 plasmid pAQ7 | 4 |
| Synechococcus sp. PCC 7002 plasmid pAQ6 | 4 |
| Halobacterium salinarum R1 plasmid PHS3 | 4 |
| Thermus thermophilus HB27 plasmid pTT27 | 3 |
| Mycobacterium sp. MCS plasmid1 | 3 |
| Dinoroseobacter shibae DFL 12 plasmid pDSHI04 | 3 |
| Marinobacter aquaeolei VT8 plasmid pMAQU02 | 3 |
| Mycobacterium sp. KMS plasmid pMKMS02 | 3 |
| Acaryochloris marina MBIC11017 plasmid pREB6 | 3 |
| Leptospira interrogans serovar Lai str. 56601 chromosome chromosome II | 3 |
| Pseudomonas fluorescens SBW25 plasmid pQBR103 | 3 |
| Aeromonas salmonicida subsp. salmonicida A449 plasmid 4 | 3 |
| Macrococcus caseolyticus JCSC5402 plasmid pMCCL2 | 3 |
| Methylobacterium nodulans ORS 2060 plasmid pMNOD03 | 3 |
| Polaromonas naphthalenivorans CJ2 plasmid pPNAP04 | 3 |
| Polaromonas naphthalenivorans CJ2 plasmid pPNAP05 | 3 |
| Polaromonas naphthalenivorans CJ2 plasmid pPNAP02 | 3 |
| Nocardia farcinica IFM 10152 plasmid pNF2 | 3 |
| Beijerinckia indica subsp. indica ATCC 9039 plasmid pBIND02 | 3 |
| Bacillus cereus G9842 plasmid pG9842_140 | 3 |
| Nostoc punctiforme PCC 73102 plasmid pNPUN03 | 3 |
| Dinoroseobacter shibae DFL 12 plasmid pDSHI05 | 3 |
| Beijerinckia indica subsp. indica ATCC 9039 plasmid pBIND01 | 3 |
| Azoarcus sp. EbN1 plasmid 2 | 3 |
| Burkholderia phytofirmans PsJN plasmid pBPHYT01 | 3 |
| Salmonella enterica subsp. enterica serovar Typhi str. CT18 plasmid pHCM2 | 3 |
| Nitrosomonas eutropha C91 plasmid2 | 3 |
| Yersinia pseudotuberculosis IP 31758 plasmid_59kb | 3 |
| Shigella sonnei Ss046 plasmid pSS_046 | 3 |
| Rhizobium leguminosarum bv. trifolii WSM2304 plasmid pRLG204 | 3 |
| Kineococcus radiotolerans SRS30216 plasmid pKRAD01 | 3 |
| Burkholderia multivorans ATCC 17616 plasmid pTGL1 | 3 |
| Acaryochloris marina MBIC11017 plasmid pREB5 | 3 |
| Rhodobacter capsulatus SB 1003 plasmid pRCB133 | 3 |
| Yersinia pestis KIM plasmid pMT-1 | 3 |
| Spirosoma linguale DSM 74 plasmid pSLIN01 | 3 |
| Yersinia pestis biovar Microtus str. 91001 plasmid pMT1 | 3 |
| Yersinia pestis Angola plasmid pMT-pPCP | 3 |
| Rhizobium leguminosarum bv. trifolii WSM1325 plasmid pR132504 | 3 |
| Borrelia duttonii Ly plasmid pl40 | 3 |
| Burkholderia vietnamiensis G4 plasmid pBVIE05 | 3 |
| Yersinia pestis CO92 plasmid pCD1 | 3 |
| Bacillus pseudofirmus OF4 plasmid pBpOF4-02 | 3 |
| Desulfovibrio vulgaris subsp. vulgaris DP4 plasmid pDVUL01 | 3 |
| Ralstonia pickettii 12J plasmid pRPIC01 | 3 |
| Rhodobacter sphaeroides 2.4.1 plasmid D | 2 |
| Salinibacter ruber DSM 13855 plasmid pSR35 | 2 |
| Thiomonas intermedia K12 plasmid pTINT02 | 2 |
| Rhodobacter sphaeroides ATCC 17025 plasmid pRSPA03 | 2 |
| Cyanothece sp. PCC 8802 plasmid pP880201 | 2 |
| Xenorhabdus nematophila ATCC 19061 plasmid XNC1_p | 2 |
| Klebsiella pneumoniae subsp. pneumoniae MGH 78578 plasmid pKPN5 | 2 |
| Cyanothece sp. PCC 7424 plasmid pP742403 | 2 |
| Klebsiella pneumoniae subsp. pneumoniae MGH 78578 plasmid pKPN4 | 2 |
| Pseudomonas syringae pv. tomato str. DC3000 plasmid pDC3000A | 2 |
| Rhodothermus marinus DSM 4252 plasmid pRMAR01 | 2 |
| Salmonella enterica subsp. enterica serovar Schwarzengrund str. CVM19633 plasmid pCVM19633_110 | 2 |
| Gordonia bronchialis DSM 43247 plasmid pGBRO01 | 2 |
| Yersinia enterocolitica subsp. enterocolitica 8081 plasmid pYVe8081 | 2 |
| Escherichia coli E24377A plasmid pETEC_73 | 2 |
| Yersinia pestis Pestoides F plasmid MT | 2 |
| Yersinia pestis Pestoides F plasmid CD | 2 |
| Xylanimonas cellulosilytica DSM 15894 plasmid pXCEL01 | 2 |
| Cyanothece sp. PCC 8801 plasmid pP880102 | 2 |
| Yersinia pseudotuberculosis IP 31758 plasmid_153kb | 2 |
| Shigella boydii Sb227 plasmid pSB4_227 | 2 |
| Nitrobacter hamburgensis X14 plasmid 3 | 2 |
| Herpetosiphon aurantiacus ATCC 23779 plasmid pHAU02 | 2 |
| Escherichia coli UTI89 plasmid pUTI89 | 2 |
| Thauera sp. MZ1T plasmid pTha01 | 2 |
| Salmonella enterica subsp. enterica serovar Paratyphi C strain RKS4594 plasmid pSPCV | 2 |
| Escherichia coli E24377A plasmid pETEC_80 | 2 |
| Shigella dysenteriae Sd197 plasmid pSD1_197 | 2 |
| Cyanothece sp. PCC 7424 plasmid pP742402 | 2 |
| Shewanella baltica OS223 plasmid pS22302 | 2 |
| Borrelia recurrentis A1 plasmid pl124 | 2 |
| Borrelia recurrentis A1 plasmid pl37 | 2 |
| Sinorhizobium medicae WSM419 plasmid pSMED03 | 2 |
| Natronomonas pharaonis DSM 2160 plasmid PL131 | 2 |
| Clostridium kluyveri DSM 555 plasmid pCKL555A | 2 |
| Nostoc punctiforme PCC 73102 plasmid pNPUN04 | 2 |
| Agrobacterium vitis S4 plasmid pAtS4b | 2 |
| Shewanella baltica OS185 plasmid pS18501 | 2 |
| Nostoc sp. PCC 7120 plasmid pCC7120epsilon | 2 |
| Nostoc sp. PCC 7120 plasmid pCC7120gamma | 2 |
| Agrobacterium vitis S4 plasmid pAtS4a | 2 |
| Bacillus weihenstephanensis KBAB4 plasmid pBWB402 | 2 |
| Ralstonia eutropha JMP134 plasmid 1 | 2 |
| Borrelia burgdorferi ZS7 plasmid ZS7_lp25 | 2 |
| Shewanella baltica OS155 plasmid pSbal01 | 2 |
| Caulobacter sp. K31 plasmid pCAUL02 | 2 |
| Clostridium perfringens str. 13 plasmid pCP13 | 2 |
| Clostridium botulinum B str. Eklund 17B plasmid pCLL | 2 |
| Rhizobium leguminosarum bv. viciae 3841 plasmid pRL7 | 2 |
| Borrelia burgdorferi B31 plasmid lp38 | 2 |
| Borrelia burgdorferi B31 plasmid lp56 | 2 |
| Borrelia burgdorferi ZS7 plasmid ZS7_lp17 | 2 |
| Borrelia burgdorferi ZS7 plasmid ZS7_cp32-3+10 | 2 |
| Borrelia burgdorferi ZS7 plasmid ZS7_cp32-1 | 2 |
| Sulfolobus islandicus Y.N.15.51 plasmid pYN01 | 2 |
| Rhizobium etli CFN 42 plasmid p42a | 2 |
| Borrelia duttonii Ly plasmid pl165 | 2 |
| Aeromonas salmonicida subsp. salmonicida A449 plasmid 5 | 2 |
| Pelobacter propionicus DSM 2379 plasmid pPRO1 | 2 |
| Finegoldia magna ATCC 29328 plasmid pFMC | 2 |
| Butyrivibrio proteoclasticus B316 chromosome 2 | 2 |
| Arthrobacter sp. FB24 plasmid 3 | 2 |
| Vibrio vulnificus YJ016 plasmid pYJ016 | 2 |
| Acaryochloris marina MBIC11017 plasmid pREB8 | 2 |
| Halobacterium salinarum R1 plasmid PHS1 | 2 |
| Polaromonas naphthalenivorans CJ2 plasmid pPNAP03 | 2 |
| Butyrivibrio proteoclasticus B316 plasmid pCY186 | 2 |
| Acidovorax sp. JS42 plasmid pAOVO01 | 2 |
| Acidovorax sp. JS42 plasmid pAOVO02 | 2 |
| Desulfotalea psychrophila LSv54 plasmid large | 2 |
| Deinococcus radiodurans R1 plasmid MP1 | 2 |
| Cyanothece sp. PCC 7822 plasmid Cy782205 | 2 |
| Staphylococcus saprophyticus subsp. saprophyticus ATCC 15305 plasmid pSSP1 | 2 |
| Jannaschia sp. CCS1 plasmid1 | 1 |
| Campylobacter lari RM2100 megaplasmid pCL2100 | 1 |
| Gluconobacter oxydans 621H plasmid pGOX1 | 1 |
| Gluconobacter oxydans 621H plasmid pGOX2 | 1 |
| Rhodospirillum rubrum ATCC 11170 plasmid unnamed | 1 |
| Ruegeria sp. TM1040 plasmid unnamed | 1 |
| Sphingopyxis alaskensis RB2256 F plasmid | 1 |
| Burkholderia multivorans ATCC 17616 plasmid pBMUL01 | 1 |
| Methanocaldococcus jannaschii DSM 2661 plasmid large ECE | 1 |
| Thiomonas intermedia K12 plasmid pTINT01 | 1 |
| Halobacterium salinarum R1 plasmid PHS2 | 1 |
| Nitrosospira multiformis ATCC 25196 plasmid 3 | 1 |
| Dinoroseobacter shibae DFL 12 plasmid pDSHI02 | 1 |
| Helicobacter pylori HPAG1 plasmid pHPAG1 | 1 |
| Nitrosospira multiformis ATCC 25196 plasmid 2 | 1 |
| Legionella pneumophila str. Lens plasmid pLPL | 1 |
| Haloarcula marismortui ATCC 43049 plasmid pNG400 | 1 |
| Haloarcula marismortui ATCC 43049 plasmid pNG500 | 1 |
| Haloarcula marismortui ATCC 43049 plasmid pNG600 | 1 |
| Rhodoferax ferrireducens T118 plasmid1 | 1 |
| Haloarcula marismortui ATCC 43049 plasmid pNG300 | 1 |
| Haloarcula marismortui ATCC 43049 plasmid pNG200 | 1 |
| Sulfolobus islandicus L.D.8.5 plasmid pLD8501 | 1 |
| Burkholderia cenocepacia HI2424 plasmid 1 | 1 |
| Haloferax volcanii DS2 plasmid pHV2 | 1 |
| Desulfobacterium autotrophicum HRM2 plasmid pHRM2a | 1 |
| Gluconacetobacter diazotrophicus PAl 5 plasmid pGDIA01 | 1 |
| Dinoroseobacter shibae DFL 12 plasmid pDSHI03 | 1 |
| Shewanella baltica OS155 plasmid pSbal03 | 1 |
| Acinetobacter baumannii ATCC 17978 plasmid pAB1 | 1 |
| Shewanella baltica OS155 plasmid pSbal02 | 1 |
| Legionella pneumophila str. Paris plasmid pLPP | 1 |
| Shewanella baltica OS195 plasmid pS19501 | 1 |
| Psychrobacter cryohalolentis K5 plasmid 1 | 1 |
| Burkholderia cenocepacia J2315 plasmid pBCJ2315 | 1 |
| Shewanella baltica OS195 plasmid pS19502 | 1 |
| Haloquadratum walsbyi DSM 16790 plasmid PL47 | 1 |
| Cupriavidus metallidurans CH34 plasmid pMOL28 | 1 |
| Staphylococcus aureus subsp. aureus USA300_FPR3757 plasmid pUSA03 | 1 |
| Lactobacillus brevis ATCC 367 plasmid 2 | 1 |
| Borrelia burgdorferi plasmid cp26 | 1 |
| Borrelia burgdorferi ZS7 plasmid ZS7_lp28-3 | 1 |
| Borrelia burgdorferi ZS7 plasmid ZS7_lp28-4 | 1 |
| Borrelia burgdorferi ZS7 plasmid ZS7_lp36 | 1 |
| Borrelia burgdorferi B31 plasmid lp5 | 1 |
| Borrelia burgdorferi B31 plasmid lp36 | 1 |
| Borrelia burgdorferi B31 plasmid cp32-3 | 1 |
| Borrelia burgdorferi B31 plasmid cp32-6 | 1 |
| Borrelia burgdorferi B31 plasmid lp21 | 1 |
| Borrelia burgdorferi B31 plasmid lp28-4 | 1 |
| Lactobacillus salivarius UCC118 plasmid pSF118-44 | 1 |
| Borrelia afzelii PKo plasmid cp27 | 1 |
| Borrelia duttonii Ly plasmid pl26 | 1 |
| Borrelia duttonii Ly plasmid pl23 | 1 |
| Borrelia duttonii Ly plasmid pl28 | 1 |
| Borrelia duttonii Ly plasmid pl35 | 1 |
| Borrelia duttonii Ly plasmid pl42 | 1 |
| Borrelia duttonii Ly plasmid pl11 | 1 |
| Borrelia recurrentis A1 plasmid pl33 | 1 |
| Borrelia afzelii PKo plasmid cp30 | 1 |
| Borrelia afzelii PKo plasmid lp25 | 1 |
| Borrelia afzelii PKo plasmid lp34 | 1 |
| Borrelia recurrentis A1 plasmid pl23 | 1 |
| Lactococcus lactis subsp. cremoris SK11 plasmid 1 | 1 |
| Planctomyces limnophilus DSM 3776 plasmid pPLIM01 | 1 |
| Agrobacterium radiobacter K84 plasmid pAgK84 | 1 |
| Cyanothece sp. PCC 8802 plasmid pP880202 | 1 |
| Cyanothece sp. PCC 7822 plasmid Cy782204 | 1 |
| Cyanothece sp. PCC 7822 plasmid Cy782206 | 1 |
| Synechocystis sp. PCC 6803 plasmid pSYSA | 1 |
| Caldicelulosiruptor becscii DSM 6725 plasmid pATHE01 | 1 |
| Campylobacter concisus 13826 plasmid pCCON16 | 1 |
| Synechococcus sp. PCC 7002 plasmid pAQ4 | 1 |
| Synechococcus elongatus PCC 7942 plasmid 1 | 1 |
| Cyanothece sp. PCC 8801 plasmid pP880101 | 1 |
| Cyanothece sp. ATCC 51142 plasmid A | 1 |
| Nostoc sp. PCC 7120 plasmid pCC7120delta | 1 |
| Anabaena variabilis ATCC 29413 plasmid B | 1 |
| Leuconostoc citreum KM20 plasmid pLCK1 | 1 |
| Chlamydophila caviae GPIC plasmid pCpGP1 | 1 |
| Lactococcus lactis subsp. cremoris SK11 plasmid 3 | 1 |
| Photobacterium profundum SS9 plasmid pPBPR1 | 1 |
| Leuconostoc citreum KM20 plasmid pLCK2 | 1 |
| Leuconostoc citreum KM20 plasmid pLCK4 | 1 |
| Clostridium acetobutylicum ATCC 824 plasmid pSOL1 | 1 |
| Clostridium botulinum B1 str. Okra plasmid pCLD | 1 |
| Acaryochloris marina MBIC11017 plasmid pREB4 | 1 |
| Anaerococcus prevotii DSM 20548 plasmid pAPRE01 | 1 |
| Vibrio fischeri ES114 plasmid pES100 | 1 |
| Ochrobactrum anthropi ATCC 49188 plasmid pOANT02 | 1 |
| Mesorhizobium sp. BNC1 plasmid 2 | 1 |
| Yersinia pestis Antiqua plasmid pMT | 1 |
| Tsukamurella paurometabola DSM 20162 plasmid pTpau01 | 1 |
| Salmonella typhimurium LT2 plasmid pSLT | 1 |
| Salmonella enterica subsp. enterica serovar Dublin str. CT_02021853 plasmid pCT02021853_74 | 1 |
| Yersinia pestis Nepal516 plasmid pMT | 1 |
| Yersinia pestis Nepal516 plasmid pPCP | 1 |
| Staphylococcus aureus subsp. aureus Mu50 plasmid VRSAp | 1 |
| Bartonella tribocorum CIP 105476 plasmid pBT01 | 1 |
| Photorhabdus asymbiotica plasmid pPAU1 | 1 |
| Prosthecochloris aestuarii DSM 271 plasmid pPAES01 | 1 |
| Mesorhizobium sp. BNC1 plasmid 3 | 1 |
| Salmonella enterica subsp. enterica serovar Newport str. SL254 plasmid pSN254 | 1 |
| Rhodobacter sphaeroides 2.4.1 plasmid C | 1 |
| Rhodobacter sphaeroides 2.4.1 plasmid B | 1 |
| Spirosoma linguale DSM 74 plasmid pSLIN07 | 1 |
| Nitrosococcus halophilus Nc4 plasmid pNHAL01 | 1 |
| Spirosoma linguale DSM 74 plasmid pSLIN04 | 1 |
| Rhodobacter sphaeroides 2.4.1 plasmid A | 1 |
| Xanthomonas axonopodis pv. citri str. 306 plasmid pXAC33 | 1 |
| Mesorhizobium loti MAFF303099 plasmid pMLb | 1 |
| Streptosporangium roseum DSM 43021 plasmid pSROS01 | 1 |
| Bacteroides fragilis NCTC 9343 plasmid pBF9343 | 1 |
| Xylella fastidiosa 9a5c plasmid pXF51 | 1 |
| Corynebacterium glutamicum R plasmid pCGR1 | 1 |
| Nocardia farcinica IFM 10152 plasmid pNF1 | 1 |
| Macrococcus caseolyticus JCSC5402 plasmid pMCCL5 | 1 |
| Methylobacterium radiotolerans JCM 2831 plasmid pMRAD06 | 1 |
| Arthrobacter chlorophenolicus A6 plasmid pACHL02 | 1 |
| Staphylococcus epidermidis ATCC 12228 plasmid pSE-12228-05 | 1 |
| Methylobacterium chloromethanicum CM4 plasmid pMCHL02 | 1 |
| Methylobacterium radiotolerans JCM 2831 plasmid pMRAD04 | 1 |
| Methylobacterium extorquens AM1 plasmid p1META1 | 1 |
| Ochrobactrum anthropi ATCC 49188 plasmid pOANT04 | 1 |
| Thermus thermophilus HB8 plasmid pTT8 | 1 |
| Bacillus cereus AH187 plasmid pAH187_45 | 1 |
| Bacillus cereus Q1 plasmid pBc53 | 1 |
| Clavibacter michiganensis subsp. sepedonicus plasmid pCSL1 | 1 |
| Aeromonas salmonicida salmonicida A449 plasmid pAsa3 | 1 |
| Staphylococcus aureus subsp. aureus JH9 plasmid pSJH901 | 1 |
| Methylobacterium nodulans ORS 2060 plasmid pMNOD04 | 1 |
| Staphylococcus aureus subsp. aureus N315 plasmid pN315 | 1 |
| Klebsiella pneumoniae subsp. pneumoniae MGH 78578 plasmid pKPN3 | 1 |
| Staphylococcus aureus subsp. aureus JH1 plasmid pSJH101 | 1 |
| Methylobacterium populi BJ001 plasmid pMPOP02 | 1 |
| Clavibacter michiganensis subsp. michiganensis NCPPB 382 plasmid pCM2 | 1 |
| Staphylococcus epidermidis RP62A plasmid pSERP | 1 |
| Staphylococcus aureus subsp. aureus USA300_TCH1516 plasmid pUSA300HOUMR | 1 |
| Synechococcus sp. PCC 7002 plasmid pAQ5 | 1 |
| Spirosoma linguale DSM 74 plasmid pSLIN03 | 1 |
| *Metasim 2* |  |
| Solibacter usitatus Ellin6076 | 223 |
| Haliangium ochraceum DSM 14365 | 211 |
| Frankia sp. EAN1pec | 206 |
| Bradyrhizobium japonicum USDA 110 chromosome | 201 |
| Catenulispora acidiphila DSM 44928 | 201 |
| Bacillus licheniformis ATCC 14580 | 191 |
| Bradyrhizobium sp. BTAi1 | 186 |
| Chitinophaga pinensis DSM 2588 | 185 |
| Streptosporangium roseum DSM 43021 | 180 |
| Myxococcus xanthus DK 1622 chromosome | 180 |
| Trichodesmium erythraeum IMS101 | 179 |
| Actinosynnema mirum DSM 43827 | 179 |
| Gluconacetobacter diazotrophicus PAl 5 | 177 |
| Saccharopolyspora erythraea NRRL 2338 chromosome | 170 |
| Nostoc punctiforme PCC 73102 | 168 |
| Spirosoma linguale DSM 74 | 164 |
| Kribbella flavida DSM 17836 | 163 |
| Rhodococcus jostii RHA1 | 161 |
| Rhodopirellula baltica SH 1 chromosome | 159 |
| Frankia alni ACN14a | 157 |
| Clostridium beijerinckii NCIMB 8052 | 157 |
| Streptomyces griseus subsp. griseus NBRC 13350 | 157 |
| Bradyrhizobium sp. ORS278 | 156 |
| Methylobacterium nodulans ORS 2060 | 156 |
| Pseudomonas aeruginosa PAO1 chromosome | 151 |
| Mycobacterium marinum M | 149 |
| Mycobacterium smegmatis str. MC2 155 | 148 |
| Mycobacterium sp. JLS | 144 |
| Pseudomonas putida KT2440 chromosome | 143 |
| Acaryochloris marina MBIC11017 | 143 |
| Pseudomonas aeruginosa PA7 | 142 |
| Pseudomonas putida W619 | 142 |
| Rhodococcus erythropolis PR4 | 141 |
| Stackebrandtia nassauensis DSM 44728 chromosome | 138 |
| Mycobacterium sp. KMS | 138 |
| Cyanothece sp. PCC 7822 chromosome | 137 |
| Methylobacterium extorquens PA1 | 136 |
| Anabaena variabilis ATCC 29413 | 134 |
| Dyadobacter fermentans DSM 18053 | 134 |
| Mesorhizobium loti MAFF303099 chromosome | 134 |
| Pseudomonas syringae pv. syringae B728a | 134 |
| Hahella chejuensis KCTC 2396 chromosome | 134 |
| Pseudomonas fluorescens SBW25 chromosome | 132 |
| Nakamurella multipartita DSM 44233 | 132 |
| Alkaliphilus metalliredigens QYMF | 132 |
| Thermomonospora curvata DSM 43183 | 131 |
| Conexibacter woesei DSM 14684 | 131 |
| Nocardiopsis dassonvillei subsp. dassonvillei DSM 43111 chromosome | 131 |
| Rhodopseudomonas palustris BisB18 | 130 |
| Flavobacterium johnsoniae UW101 | 129 |
| Corynebacterium glutamicum ATCC 13032 | 129 |
| Herpetosiphon aurantiacus ATCC 23779 chromosome | 129 |
| Nocardia farcinica IFM 10152 | 129 |
| Nostoc sp. PCC 7120 | 129 |
| Serratia proteamaculans 568 chromosome | 128 |
| Methylobacterium chloromethanicum CM4 | 128 |
| Xanthomonas oryzae pv. oryzae PXO99A | 128 |
| Burkholderia multivorans ATCC 17616 chromosome 2 | 126 |
| Sphingomonas wittichii RW1 | 126 |
| Photorhabdus luminescens subsp. laumondii TTO1 | 126 |
| Methylobacterium radiotolerans JCM 2831 | 125 |
| Shewanella piezotolerans WP3 chromosome | 124 |
| Shewanella baltica OS185 | 124 |
| Candidatus Koribacter versatilis Ellin345 | 124 |
| Bordetella petrii DSM 12804 | 123 |
| Mycobacterium ulcerans Agy99 | 122 |
| Klebsiella pneumoniae subsp. pneumoniae MGH 78578 | 121 |
| Methylobacterium populi BJ001 | 120 |
| Bordetella parapertussis 12822 | 120 |
| Bacillus cereus B4264 | 120 |
| Salmonella enterica subsp. enterica serovar Typhimurium str. LT2 chromosome | 120 |
| Roseiflexus castenholzii DSM 13941 | 120 |
| Dickeya dadantii 3937 chromosome | 120 |
| Bacillus weihenstephanensis KBAB4 | 120 |
| Methylobacterium extorquens AM1 | 119 |
| Pseudomonas syringae pv. phaseolicola 1448A | 119 |
| Cyanothece sp. PCC 7424 | 119 |
| Pseudomonas entomophila L48 | 119 |
| Escherichia coli CFT073 | 118 |
| Anaeromyxobacter sp. K | 116 |
| Cytophaga hutchinsonii ATCC 33406 | 116 |
| Bacillus cereus AH187 | 116 |
| Photorhabdus asymbiotica chromosome | 115 |
| Bacillus anthracis str. Ames | 115 |
| Bacteroides vulgatus ATCC 8482 chromosome | 115 |
| Pseudomonas putida F1 | 115 |
| Azotobacter vinelandii DJ chromosome | 115 |
| Caulobacter segnis ATCC 21756 chromosome | 115 |
| Pedobacter heparinus DSM 2366 | 114 |
| Geobacter sp. M21 | 114 |
| Mycobacterium sp. MCS | 114 |
| Bacillus cereus ATCC 10987 | 114 |
| Desulfobacterium autotrophicum HRM2 | 114 |
| Rhodopseudomonas palustris HaA2 | 114 |
| Escherichia coli APEC O1 | 114 |
| Xanthomonas oryzae pv. oryzae MAFF 311018 | 114 |
| Pseudomonas syringae pv. tomato str. DC3000 chromosome | 113 |
| Gordonia bronchialis DSM 43247 | 113 |
| Escherichia coli UTI89 | 113 |
| Methanosarcina acetivorans C2A chromosome | 113 |
| Rhodopseudomonas palustris BisA53 | 113 |
| Bacillus cereus E33L | 113 |
| Colwellia psychrerythraea 34H | 112 |
| Polaromonas sp. JS666 | 112 |
| Planctomyces limnophilus DSM 3776 chromosome | 112 |
| Mycobacterium avium 104 | 112 |
| Frankia sp. CcI3 | 111 |
| Rhodoferax ferrireducens T118 | 111 |
| Shewanella woodyi ATCC 51908 | 111 |
| Rhodopseudomonas palustris CGA009 chromosome | 111 |
| Marinomonas sp. MWYL1 chromosome | 111 |
| Yersinia pestis biovar Microtus str. 91001 | 110 |
| Syntrophobacter fumaroxidans MPOB | 110 |
| Xanthomonas campestris pv. campestris str. 8004 chromosome | 110 |
| Thauera sp. MZ1T | 110 |
| Bacillus cereus AH820 | 110 |
| Salmonella enterica subsp. enterica serovar Typhi str. Ty2 chromosome | 110 |
| Escherichia coli E24377A | 109 |
| Shigella flexneri 2a str. 2457T | 109 |
| Rhizobium etli CIAT 652 | 109 |
| Chromobacterium violaceum ATCC 12472 | 109 |
| Yersinia pseudotuberculosis IP 32953 | 108 |
| Shewanella baltica OS195 | 108 |
| Xanthomonas axonopodis pv. citri str. 306 chromosome | 108 |
| Teredinibacter turnerae T7901 | 108 |
| Caulobacter crescentus CB15 | 108 |
| Bacteroides fragilis YCH46 chromosome | 108 |
| Escherichia coli str. K-12 substr. W3110 chromosome | 107 |
| Caulobacter sp. K31 chromosome | 107 |
| Salmonella enterica subsp. enterica serovar Paratyphi B str. SPB7 chromosome | 107 |
| Bacillus thuringiensis str. Al Hakam | 107 |
| Paenibacillus polymyxa E681 chromosome | 106 |
| Pseudoalteromonas atlantica T6c | 106 |
| Arthrobacter aurescens TC1 | 106 |
| Chloroflexus aggregans DSM 9485 chromosome | 106 |
| Rhodopseudomonas palustris BisB5 | 106 |
| Escherichia coli 536 | 106 |
| Salmonella enterica subsp. enterica serovar Schwarzengrund str. CVM19633 | 106 |
| Yersinia pestis Nepal516 | 106 |
| Escherichia coli B str. REL606 chromosome | 105 |
| Burkholderia xenovorans LB400 chromosome 1 | 105 |
| Shigella flexneri 2a str. 301 | 105 |
| Azorhizobium caulinodans ORS 571 | 105 |
| Desulfitobacterium hafniense Y51 | 104 |
| Escherichia coli HS | 104 |
| Bacillus cereus G9842 | 104 |
| Gloeobacter violaceus PCC 7421 chromosome | 104 |
| Leptospira interrogans serovar Lai str. 56601 chromosome chromosome I | 104 |
| Shewanella pealeana ATCC 700345 | 104 |
| Bacillus cereus ATCC 14579 | 104 |
| Shewanella baltica OS155 | 104 |
| Clostridium phytofermentans ISDg | 104 |
| Mycobacterium tuberculosis F11 | 104 |
| Ralstonia solanacearum GMI1000 | 103 |
| Pseudomonas stutzeri A1501 | 103 |
| Escherichia coli ATCC 8739 | 103 |
| Acidovorax sp. JS42 | 103 |
| Cyanothece sp. PCC 8802 | 103 |
| Salmonella enterica subsp. enterica serovar Heidelberg str. SL476 | 103 |
| Sebaldella termitidis ATCC 33386 | 102 |
| Shewanella baltica OS223 chromosome | 102 |
| Parabacteroides distasonis ATCC 8503 | 102 |
| Acinetobacter sp. DR1 chromosome | 101 |
| Bdellovibrio bacteriovorus HD100 | 101 |
| Yersinia pseudotuberculosis YPIII chromosome | 101 |
| Xanthomonas campestris pv. campestris str. ATCC 33913 | 101 |
| Hyphomonas neptunium ATCC 15444 | 101 |
| Psychromonas ingrahamii 37 | 100 |
| Methanosarcina barkeri str. fusaro chromosome | 100 |
| Geodermatophilus obscurus DSM 43160 | 100 |
| Bacillus cereus Q1 chromosome | 100 |
| Candidatus Nitrospira defluvii | 100 |
| Shewanella denitrificans OS217 | 99 |
| Escherichia coli str. K-12 substr. DH10B | 99 |
| Nocardioides sp. JS614 | 99 |
| Clostridium difficile 630 | 99 |
| Cyanothece sp. PCC 7425 | 99 |
| Clostridium kluyveri DSM 555 | 99 |
| Mycobacterium tuberculosis H37Rv | 98 |
| Shewanella frigidimarina NCIMB 400 | 98 |
| Beutenbergia cavernae DSM 12333 | 98 |
| Klebsiella pneumoniae 342 | 98 |
| Yersinia pestis Antiqua | 98 |
| Bordetella bronchiseptica RB50 | 98 |
| Aromatoleum aromaticum EbN1 | 98 |
| Rhizobium leguminosarum bv. viciae 3841 | 98 |
| Aeromonas hydrophila subsp. hydrophila ATCC 7966 | 97 |
| Desulfovibrio salexigens DSM 2638 | 97 |
| Clostridium botulinum F str. Langeland | 97 |
| Bacteroides fragilis NCTC 9343 chromosome | 97 |
| Xanthobacter autotrophicus Py2 | 97 |
| Enterobacter sp. 638 | 96 |
| Rhizobium leguminosarum bv. trifolii WSM1325 | 96 |
| Azoarcus sp. BH72 | 96 |
| Shigella boydii Sb227 | 96 |
| Nitrobacter hamburgensis X14 | 95 |
| Pectobacterium atrosepticum SCRI1043 chromosome | 95 |
| Bacillus anthracis str. Sterne chromosome | 95 |
| Cellulomonas flavigena DSM 20109 chromosome | 95 |
| Stenotrophomonas maltophilia K279a chromosome | 94 |
| Mycobacterium tuberculosis H37Ra | 94 |
| Salmonella enterica subsp. enterica serovar Agona str. SL483 | 94 |
| Rhizobium etli CFN 42 | 93 |
| Rhodobacter capsulatus SB 1003 chromosome | 93 |
| Clostridium botulinum A3 str. Loch Maree | 93 |
| Desulfotomaculum acetoxidans DSM 771 | 93 |
| Aeromonas salmonicida subsp. salmonicida A449 | 93 |
| Yersinia pestis Pestoides F | 93 |
| Clostridium botulinum Ba4 str. 657 | 93 |
| Cyanothece sp. ATCC 51142 chromosome chromosome circular | 92 |
| Geobacter bemidjiensis Bem | 92 |
| Mycobacterium tuberculosis CDC1551 | 92 |
| Gamma proteobacterium HdN1 | 92 |
| Geobacter sulfurreducens PCA chromosome | 92 |
| Roseobacter denitrificans OCh 114 | 92 |
| Salmonella enterica subsp. enterica serovar Typhi str. CT18 chromosome | 91 |
| Salmonella enterica subsp. enterica serovar Dublin str. CT_02021853 | 91 |
| Salmonella enterica subsp. enterica serovar Paratyphi A str. ATCC 9150 chromosome | 91 |
| Rhodospirillum centenum SW chromosome | 91 |
| Methylocella silvestris BL2 chromosome | 91 |
| Escherichia coli str. K-12 substr. MG1655 chromosome | 91 |
| Agrobacterium radiobacter K84 chromosome 1 | 90 |
| Mycobacterium bovis BCG str. Pasteur 1173P2 | 90 |
| Salmonella enterica subsp. enterica serovar Newport str. SL254 | 90 |
| Burkholderia vietnamiensis G4 chromosome 1 | 90 |
| Yersinia enterocolitica subsp. enterocolitica 8081 | 90 |
| Pseudomonas mendocina ymp | 90 |
| Cellvibrio japonicus Ueda107 | 90 |
| Mesorhizobium sp. BNC1 | 89 |
| Clostridium botulinum A str. ATCC 3502 | 89 |
| Dechloromonas aromatica RCB | 89 |
| Marinobacter aquaeolei VT8 | 89 |
| Acinetobacter baumannii ACICU | 89 |
| Arthrobacter chlorophenolicus A6 | 88 |
| Thermobispora bispora DSM 43833 chromosome | 88 |
| Photobacterium profundum SS9 chromosome 1 | 88 |
| Pelobacter carbinolicus DSM 2380 | 88 |
| Yersinia pestis CO92 | 88 |
| Burkholderia thailandensis E264 chromosome I | 88 |
| Burkholderia pseudomallei 1106a chromosome I | 87 |
| Kineococcus radiotolerans SRS30216 | 87 |
| Clostridium botulinum B1 str. Okra | 87 |
| Desulfomicrobium baculatum DSM 4028 | 87 |
| Burkholderia pseudomallei 1710b chromosome I | 87 |
| Clostridium thermocellum ATCC 27405 | 87 |
| Rhodospirillum rubrum ATCC 11170 | 87 |
| Geobacter uraniireducens Rf4 chromosome | 87 |
| Yersinia pestis Angola | 87 |
| Bordetella pertussis Tohama I | 87 |
| Chromohalobacter salexigens DSM 3043 | 86 |
| Symbiobacterium thermophilum IAM 14863 | 86 |
| Ralstonia pickettii 12J chromosome 1 | 86 |
| Xanthomonas oryzae pv. oryzae KACC10331 chromosome | 85 |
| Gramella forsetii KT0803 | 85 |
| Shigella sonnei Ss046 | 85 |
| Burkholderia pseudomallei K96243 chromosome 1 | 85 |
| Vibrio vulnificus CMCP6 chromosome I | 85 |
| Clostridium botulinum A str. Hall | 85 |
| Jannaschia sp. CCS1 | 85 |
| Azospirillum sp. B510 chromosome | 85 |
| Shewanella amazonensis SB2B | 85 |
| Legionella pneumophila str. Paris | 84 |
| Acinetobacter baumannii AB0057 | 84 |
| Herminiimonas arsenicoxydans | 84 |
| Edwardsiella tarda EIB202 chromosome | 84 |
| Acinetobacter sp. ADP1 | 84 |
| Shigella dysenteriae Sd197 | 83 |
| Butyrivibrio proteoclasticus B316 chromosome 1 | 83 |
| Mycobacterium tuberculosis KZN 1435 | 83 |
| Shigella flexneri 5 str. 8401 | 83 |
| Burkholderia phytofirmans PsJN chromosome 1 | 83 |
| Saccharomonospora viridis DSM 43017 | 83 |
| Eggerthella lenta DSM 2243 | 83 |
| Arthrobacter sp. FB24 chromosome chromosome 1 | 82 |
| Janthinobacterium sp. Marseille | 82 |
| Phenylobacterium zucineum HLK1 | 82 |
| Xylanimonas cellulosilytica DSM 15894 | 82 |
| Bacillus pseudofirmus OF4 chromosome | 82 |
| Burkholderia mallei NCTC 10247 chromosome I | 82 |
| Yersinia pestis KIM 10 chromosome | 82 |
| Desulfovibrio vulgaris str. Hildenborough chromosome | 81 |
| Rhizobium leguminosarum bv. trifolii WSM2304 | 81 |
| Burkholderia multivorans ATCC 17616 chromosome 1 | 81 |
| Nitrosococcus halophilus Nc4 chromosome | 81 |
| Clostridium botulinum A str. ATCC 19397 | 81 |
| Fibrobacter succinogenes subsp. succinogenes S85 | 80 |
| Clostridium botulinum B str. Eklund 17B | 80 |
| Vibrio sp. Ex25 chromosome 1 | 80 |
| Clostridium botulinum E3 str. Alaska E43 | 80 |
| Burkholderia mallei ATCC 23344 chromosome 1 | 80 |
| Xanthomonas albilineans chromosome | 80 |
| Burkholderia phytofirmans PsJN chromosome 2 | 79 |
| Desulfotalea psychrophila LSv54 | 79 |
| Cyanothece sp. PCC 8801 | 79 |
| Exiguobacterium sp. AT1b | 79 |
| Desulfovibrio vulgaris subsp. vulgaris DP4 | 79 |
| Burkholderia cenocepacia AU 1054 chromosome 1 | 79 |
| Legionella pneumophila str. Corby chromosome | 79 |
| Geobacter lovleyi SZ | 79 |
| Acinetobacter baumannii SDF | 79 |
| Ralstonia eutropha JMP134 chromosome 1 | 79 |
| Desulfovibrio desulfuricans subsp. desulfuricans str. G20 chromosome | 79 |
| Burkholderia pseudomallei 668 chromosome I | 79 |
| Burkholderia mallei SAVP1 chromosome I | 78 |
| Ralstonia eutropha H16 chromosome 1 | 78 |
| Alkaliphilus oremlandii OhILAs | 78 |
| Rhizobium sp. NGR234 | 78 |
| Clostridium acetobutylicum ATCC 824 | 78 |
| Burkholderia ambifaria AMMD chromosome 1 | 77 |
| Dinoroseobacter shibae DFL 12 | 77 |
| Flavobacteriales bacterium HTCC2170 chromosome | 77 |
| Sinorhizobium meliloti 1021 | 77 |
| Acidobacterium capsulatum ATCC 51196 | 77 |
| Agrobacterium vitis S4 chromosome 1 | 77 |
| Rhodobacter sphaeroides ATCC 17029 chromosome chromosome 1 | 77 |
| Salmonella enterica subsp. enterica serovar Paratyphi C strain RKS4594 | 76 |
| Ralstonia eutropha H16 chromosome 2 | 76 |
| Synechocystis sp. PCC 6803 chromosome | 76 |
| Burkholderia pseudomallei 668 chromosome II | 76 |
| Syntrophus aciditrophicus SB chromosome | 76 |
| Bordetella avium 197N | 76 |
| Beijerinckia indica subsp. indica ATCC 9039 | 76 |
| Bacillus amyloliquefaciens FZB42 | 76 |
| Sanguibacter keddieii DSM 10542 | 75 |
| Salinibacter ruber DSM 13855 | 75 |
| Methanosarcina mazei Go1 chromosome | 75 |
| Burkholderia mallei NCTC 10229 chromosome I | 75 |
| Haloquadratum walsbyi DSM 16790 | 75 |
| Thermobifida fusca YX | 75 |
| Methylibium petroleiphilum PM1 chromosome | 75 |
| Bacillus subtilis subsp. subtilis str. 168 | 74 |
| Renibacterium salmoninarum ATCC 33209 chromosome | 74 |
| Burkholderia sp. 383 chromosome 1 | 74 |
| Sinorhizobium medicae WSM419 | 74 |
| Meiothermus ruber DSM 1279 chromosome | 74 |
| Burkholderia cenocepacia HI2424 chromosome 1 | 74 |
| Acinetobacter baumannii AYE | 74 |
| Alicyclobacillus acidocaldarius subsp. acidocaldarius DSM 446 | 73 |
| Acinetobacter baumannii ATCC 17978 | 73 |
| Denitrovibrio acetiphilus DSM 12809 chromosome | 73 |
| Croceibacter atlanticus HTCC2559 chromosome | 73 |
| Caldicellulosiruptor saccharolyticus DSM 8903 | 73 |
| Burkholderia phymatum STM815 chromosome 1 | 73 |
| Geobacillus sp. WCH70 | 72 |
| Pelobacter propionicus DSM 2379 | 72 |
| Synechococcus sp. PCC 7002 chromosome | 72 |
| Corynebacterium glutamicum R chromosome | 72 |
| Candidatus Desulfococcus oleovorans Hxd3 | 72 |
| Xenorhabdus nematophila ATCC 19061 | 72 |
| Listeria welshimeri serovar 6b str. SLCC5334 | 71 |
| Vibrio cholerae O395 chromosome 2 | 71 |
| Clostridium cellulolyticum H10 | 71 |
| Maricaulis maris MCS10 | 71 |
| Synechococcus elongatus PCC 6301 | 71 |
| Clavibacter michiganensis subsp. sepedonicus | 70 |
| Burkholderia xenovorans LB400 chromosome 2 | 70 |
| Polaromonas naphthalenivorans CJ2 | 70 |
| Robiginitalea biformata HTCC2501 | 70 |
| Geobacillus kaustophilus HTA426 | 70 |
| Aliivibrio salmonicida LFI1238 chromosome 1 | 70 |
| Methylococcus capsulatus str. Bath | 70 |
| Vibrio vulnificus YJ016 chromosome I | 69 |
| Bacillus selenitireducens MLS10 chromosome | 69 |
| Clostridium perfringens ATCC 13124 | 69 |
| Burkholderia sp. 383 chromosome 2 | 69 |
| Kangiella koreensis DSM 16069 | 69 |
| Rubrobacter xylanophilus DSM 9941 | 69 |
| Oceanobacillus iheyensis HTE831 | 69 |
| Eubacterium rectale ATCC 33656 | 69 |
| Slackia heliotrinireducens DSM 20476 | 68 |
| Jonesia denitrificans DSM 20603 | 68 |
| Burkholderia pseudomallei 1106a chromosome II | 68 |
| Methanocella paludicola SANAE chromosome | 68 |
| Sulfolobus islandicus L.S.2.15 chromosome | 68 |
| Brachybacterium faecium DSM 4810 | 68 |
| Nitrosococcus oceani ATCC 19707 | 68 |
| Ralstonia eutropha JMP134 chromosome 2 | 68 |
| Capnocytophaga ochracea DSM 7271 | 68 |
| Ralstonia pickettii 12D chromosome 1 | 68 |
| Xylella fastidiosa Temecula1 | 68 |
| Burkholderia phymatum STM815 chromosome 2 | 68 |
| Prevotella ruminicola 23 chromosome | 68 |
| Listeria innocua Clip11262 | 68 |
| Vibrio parahaemolyticus RIMD 2210633 chromosome 1 | 68 |
| Chlorobium phaeobacteroides DSM 266 | 67 |
| Chloroherpeton thalassium ATCC 35110 | 67 |
| Burkholderia cenocepacia J2315 chromosome chromosome 1 | 67 |
| Sphingopyxis alaskensis RB2256 | 67 |
| Lactobacillus plantarum WCFS1 | 67 |
| Cupriavidus taiwanensis str. LMG19424 chromosome I | 67 |
| Staphylococcus epidermidis ATCC 12228 | 67 |
| Staphylococcus aureus subsp. aureus USA300_FPR3757 | 67 |
| Enterococcus faecalis V583 chromosome | 67 |
| Haloarcula marismortui ATCC 43049 chromosome chromosome I | 67 |
| Oligotropha carboxidovorans OM5 | 67 |
| Sulfolobus islandicus M.16.27 chromosome | 66 |
| Vibrio cholerae O1 biovar El Tor str. N16961 chromosome I | 66 |
| Tsukamurella paurometabola DSM 20162 chromosome | 66 |
| Cupriavidus metallidurans CH34 chromosome chromosome 1 | 66 |
| Yersinia pseudotuberculosis IP 31758 | 66 |
| Pelotomaculum thermopropionicum SI chromosome | 66 |
| Mycobacterium leprae TN chromosome | 66 |
| Heliobacterium modesticaldum Ice1 | 66 |
| Ruegeria sp. TM1040 | 65 |
| Desulfovibrio desulfuricans subsp. desulfuricans str. ATCC 27774 | 65 |
| Idiomarina loihiensis L2TR | 65 |
| Bifidobacterium dentium Bd1 | 65 |
| Staphylococcus aureus subsp. aureus Mu3 | 64 |
| Staphylococcus epidermidis RP62A | 64 |
| Acidithiobacillus ferrooxidans ATCC 23270 | 64 |
| Methylobacillus flagellatus KT | 64 |
| Novosphingobium aromaticivorans DSM 12444 | 64 |
| Thiomonas intermedia K12 chromosome | 64 |
| Sulfolobus islandicus M.16.4 chromosome | 64 |
| Sulfolobus islandicus Y.G.57.14 chromosome | 63 |
| Haloferax volcanii DS2 chromosome | 63 |
| Erythrobacter litoralis HTCC2594 chromosome | 63 |
| Clostridium novyi NT | 63 |
| Clavibacter michiganensis subsp. michiganensis NCPPB 382 | 63 |
| Syntrophomonas wolfei subsp. wolfei str. Goettingen | 62 |
| Cupriavidus taiwanensis str. LMG19424 chromosome 2 | 62 |
| Pseudoalteromonas haloplanktis TAC125 chromosome I | 62 |
| Thermoanaerobacter sp. X514 | 62 |
| Xylella fastidiosa M12 chromosome | 62 |
| Brachyspira murdochii DSM 12563 chromosome | 62 |
| Meiothermus silvanus DSM 9946 chromosome | 61 |
| Chlorobium chlorochromatii CaD3 | 61 |
| Cupriavidus metallidurans CH34 megaplasmid | 61 |
| Rhizobium sp. NGR234 plasmid pNGR234b | 61 |
| Ochrobactrum anthropi ATCC 49188 chromosome 1 | 61 |
| Synechococcus sp. WH 8102 | 61 |
| Lactobacillus casei ATCC 334 | 61 |
| Candidatus Methanoregula boonei 6A8 chromosome | 60 |
| Bifidobacterium longum DJO10A | 60 |
| Thiobacillus denitrificans ATCC 25259 | 60 |
| Sulfolobus tokodaii str. 7 chromosome | 60 |
| Gluconobacter oxydans 621H chromosome | 60 |
| Burkholderia mallei NCTC 10247 chromosome II | 60 |
| Rhodothermus marinus DSM 4252 | 60 |
| Burkholderia multivorans ATCC 17616 chromosome chromosome 1 | 60 |
| Corynebacterium diphtheriae NCTC 13129 | 60 |
| Streptococcus gordonii str. Challis substr. CH1 | 60 |
| Corynebacterium jeikeium K411 | 60 |
| Paracoccus denitrificans PD1222 chromosome 1 | 60 |
| Bartonella tribocorum CIP 105476 | 60 |
| Lactobacillus casei str. Zhang chromosome | 59 |
| Legionella pneumophila str. Lens | 59 |
| Listeria monocytogenes str. 4b F2365 | 59 |
| Acidithiobacillus ferrooxidans ATCC 53993 | 59 |
| Clostridium tetani E88 chromosome | 59 |
| Staphylococcus aureus RF122 | 59 |
| Staphylococcus aureus subsp. aureus NCTC 8325 | 59 |
| Legionella pneumophila subsp. pneumophila str. Philadelphia 1 chromosome | 59 |
| Treponema denticola ATCC 35405 chromosome | 59 |
| Burkholderia pseudomallei 1710b chromosome II | 58 |
| Vibrio fischeri ES114 chromosome I | 58 |
| Synechococcus elongatus PCC 7942 chromosome | 58 |
| Desulfohalobium retbaense DSM 5692 | 58 |
| Clostridium perfringens SM101 | 58 |
| Granulibacter bethesdensis CGDNIH1 chromosome | 58 |
| Staphylococcus aureus subsp. aureus MRSA252 chromosome | 58 |
| Alcanivorax borkumensis SK2 | 58 |
| Porphyromonas gingivalis ATCC 33277 | 58 |
| Clostridium perfringens str. 13 | 58 |
| Nitrosospira multiformis ATCC 25196 chromosome | 58 |
| Staphylococcus aureus subsp. aureus USA300_TCH1516 chromosome | 57 |
| Actinobacillus pleuropneumoniae L20 | 57 |
| Thermoanaerobacter tengcongensis MB4 | 57 |
| Agrobacterium tumefaciens str. C58 chromosome circular | 57 |
| Micrococcus luteus NCTC 2665 | 57 |
| Moorella thermoacetica ATCC 39073 | 57 |
| Chlorobium phaeobacteroides BS1 | 57 |
| Pasteurella multocida subsp. multocida str. Pm70 | 57 |
| Nitrosomonas europaea ATCC 19718 | 57 |
| Staphylococcus aureus subsp. aureus MSSA476 chromosome | 57 |
| Candidatus Puniceispirillum marinum IMCC1322 chromosome | 57 |
| Rhodobacter sphaeroides ATCC 17025 chromosome | 56 |
| Sulfolobus solfataricus P2 chromosome | 56 |
| Streptococcus pneumoniae 70585 | 56 |
| Brucella melitensis biovar Abortus 2308 chromosome I | 56 |
| Neisseria meningitidis 053442 | 56 |
| Halomicrobium mukohataei DSM 12286 | 56 |
| Actinobacillus pleuropneumoniae serovar 3 str. JL03 | 56 |
| Chlorobaculum parvum NCIB 8327 | 55 |
| Synechococcus sp. CC9605 | 55 |
| Burkholderia vietnamiensis G4 chromosome 2 | 55 |
| Propionibacterium acnes KPA171202 | 55 |
| Rhodobacter sphaeroides 2.4.1 chromosome 1 | 55 |
| Streptococcus sanguinis SK36 | 55 |
| Nitrosomonas eutropha C91 | 55 |
| Sulfolobus islandicus M.14.25 chromosome | 55 |
| Leifsonia xyli subsp. xyli str. CTCB07 | 55 |
| Burkholderia thailandensis E264 chromosome II | 55 |
| Vibrio fischeri MJ11 chromosome I | 55 |
| Synechococcus sp. CC9311 | 54 |
| Streptococcus suis 98HAH33 | 54 |
| Staphylococcus aureus subsp. aureus MW2 | 54 |
| Eubacterium eligens ATCC 27750 | 54 |
| Staphylococcus aureus subsp. aureus JH1 | 54 |
| Parvularcula bermudensis HTCC2503 chromosome | 54 |
| Burkholderia cenocepacia J2315 chromosome chromosome 2 | 53 |
| Prosthecochloris aestuarii DSM 271 | 53 |
| Lactococcus lactis subsp. cremoris MG1363 | 53 |
| Natronomonas pharaonis DSM 2160 | 53 |
| Kytococcus sedentarius DSM 20547 | 53 |
| Ammonifex degensii KC4 | 53 |
| Sulfolobus islandicus L.D.8.5 chromosome | 53 |
| Halorhabdus utahensis DSM 12940 chromosome | 53 |
| Brucella melitensis bv. 1 str. 16M chromosome chromosome I | 53 |
| Kosmotoga olearia TBF 19.5.1 | 53 |
| Chlorobium luteolum DSM 273 | 52 |
| Neisseria gonorrhoeae NCCP11945 chromosome | 52 |
| Thermosipho africanus TCF52B | 52 |
| Sphaerobacter thermophilus DSM 20745 chromosome 1 | 52 |
| Sulfolobus islandicus Y.N.15.51 chromosome | 52 |
| Arcobacter butzleri RM4018 | 52 |
| Metallosphaera sedula DSM 5348 chromosome | 51 |
| Staphylococcus aureus subsp. aureus Mu50 | 51 |
| Xylella fastidiosa 9a5c | 51 |
| Vibrio sp. Ex25 chromosome 2 | 51 |
| Halothermothrix orenii H 168 | 51 |
| Kocuria rhizophila DC2201 | 51 |
| Francisella tularensis subsp. tularensis SCHU S4 | 51 |
| Burkholderia cenocepacia AU 1054 chromosome 2 | 51 |
| Psychrobacter cryohalolentis K5 | 51 |
| Haemophilus somnus 2336 | 50 |
| Deinococcus geothermalis DSM 11300 | 50 |
| Burkholderia mallei ATCC 23344 chromosome 2 | 50 |
| Methanococcus vannielii SB | 50 |
| Synechococcus sp. WH 7803 | 50 |
| Burkholderia cenocepacia HI2424 chromosome 2 | 50 |
| Staphylococcus haemolyticus JCSC1435 | 50 |
| Streptococcus mutans UA159 chromosome | 50 |
| Campylobacter curvus 525.92 | 50 |
| Archaeoglobus fulgidus DSM 4304 | 50 |
| Leptotrichia buccalis DSM 1135 | 50 |
| Prochlorococcus marinus str. MIT 9303 | 50 |
| Streptococcus equi subsp. zooepidemicus | 49 |
| Staphylococcus aureus subsp. aureus str. Newman | 49 |
| Picrophilus torridus DSM 9790 chromosome | 49 |
| Prochlorococcus marinus str. MIT 9313 chromosome | 48 |
| Lactobacillus brevis ATCC 367 | 48 |
| Petrotoga mobilis SJ95 | 48 |
| Staphylococcus aureus subsp. aureus COL | 48 |
| Streptococcus agalactiae NEM316 | 48 |
| Caldicelulosiruptor becscii DSM 6725 chromosome | 48 |
| Streptococcus pyogenes M1 GAS chromosome | 48 |
| Chlorobium limicola DSM 245 | 48 |
| Fusobacterium nucleatum subsp. nucleatum ATCC 25586 chromosome | 48 |
| Streptococcus pneumoniae R6 | 47 |
| Acidothermus cellulolyticus 11B | 47 |
| Wolinella succinogenes DSM 1740 chromosome | 47 |
| Staphylococcus aureus subsp. aureus N315 | 47 |
| Francisella tularensis subsp. mediasiatica FSC147 | 47 |
| Bifidobacterium longum subsp. infantis ATCC 15697 | 47 |
| Burkholderia cepacia AMMD chromosome 2 | 47 |
| Streptococcus mutans NN2025 | 47 |
| Thermomicrobium roseum DSM 5159 | 47 |
| Agrobacterium tumefaciens str. C58 chromosome linear | 47 |
| Corynebacterium urealyticum DSM 7109 | 47 |
| Xylella fastidiosa M23 | 47 |
| Pyrococcus furiosus DSM 3638 | 46 |
| Neisseria meningitidis FAM18 | 46 |
| Staphylococcus saprophyticus subsp. saprophyticus ATCC 15305 | 46 |
| Staphylococcus aureus subsp. aureus JH9 | 46 |
| Streptococcus pneumoniae JJA | 46 |
| Methanoculleus marisnigri JR1 chromosome | 46 |
| Streptococcus pneumoniae G54 chromosome | 46 |
| Burkholderia mallei SAVP1 chromosome II | 45 |
| Halorhodospira halophila SL1 | 45 |
| Thermococcus onnurineus NA1 | 45 |
| Ochrobactrum anthropi ATCC 49188 chromosome 2 | 45 |
| Orientia tsutsugamushi Boryong | 45 |
| Methylacidiphilum infernorum V4 | 45 |
| Ralstonia solanacearum GMI1000 plasmid pGMI1000MP | 45 |
| Thermoanaerobacter pseudethanolicus ATCC 33223 | 45 |
| Lactobacillus sakei subsp. sakei 23K | 45 |
| Vibrio vulnificus YJ016 chromosome II | 45 |
| Brucella abortus S19 chromosome 1 | 45 |
| Agrobacterium radiobacter K84 chromosome 2 | 44 |
| Burkholderia mallei NCTC 10229 chromosome II | 44 |
| Candidatus Protochlamydia amoebophila UWE25 | 44 |
| Bifidobacterium longum NCC2705 chromosome | 44 |
| Deinococcus radiodurans R1 chromosome 1 | 44 |
| Prochlorococcus marinus str. NATL2A | 44 |
| Streptococcus pyogenes MGAS315 | 44 |
| Vibrio parahaemolyticus RIMD 2210633 chromosome 2 | 44 |
| Thermotoga sp. RQ2 | 44 |
| Prochlorococcus marinus str. MIT 9515 | 44 |
| Chlorobium tepidum TLS | 44 |
| Streptococcus pyogenes NZ131 chromosome | 44 |
| Actinobacillus succinogenes 130Z | 44 |
| Methanococcoides burtonii DSM 6242 | 43 |
| Thermotoga neapolitana DSM 4359 | 43 |
| Lactococcus lactis subsp. cremoris SK11 | 43 |
| Pyrobaculum arsenaticum DSM 13514 | 43 |
| Streptococcus pyogenes MGAS10750 chromosome | 43 |
| Lactococcus lactis subsp. lactis Il1403 | 43 |
| Halorubrum lacusprofundi ATCC 49239 chromosome 1 | 43 |
| Streptococcus pyogenes MGAS2096 chromosome | 43 |
| Coxiella burnetii CbuK_Q154 chromosome | 43 |
| Lactobacillus fermentum IFO 3956 | 43 |
| Sulfurospirillum deleyianum DSM 6946 | 43 |
| Staphylothermus marinus F1 chromosome | 43 |
| Anaerococcus prevotii DSM 20548 | 43 |
| Anoxybacillus flavithermus WK1 | 43 |
| Lactobacillus acidophilus NCFM chromosome | 43 |
| Porphyromonas gingivalis W83 chromosome | 43 |
| Thermanaerovibrio acidaminovorans DSM 6589 | 43 |
| Zymomonas mobilis subsp. mobilis ZM4 chromosome | 42 |
| Sulfurihydrogenibium sp. YO3AOP1 | 42 |
| Lactobacillus johnsonii NCC 533 | 42 |
| Pyrococcus horikoshii OT3 | 42 |
| Lactobacillus helveticus DPC 4571 | 42 |
| Thermococcus kodakarensis KOD1 | 42 |
| Pyrobaculum calidifontis JCM 11548 chromosome | 42 |
| Streptococcus mitis B6 | 42 |
| Paracoccus denitrificans PD1222 chromosome 2 | 42 |
| Streptococcus uberis 0140J chromosome | 42 |
| Dictyoglomus turgidum DSM 6724 | 42 |
| Methanococcus maripaludis C5 | 42 |
| Mannheimia succiniciproducens MBEL55E | 42 |
| Orientia tsutsugamushi str. Ikeda | 42 |
| Candidatus Korarchaeum cryptofilum OPF8 chromosome | 41 |
| Lactobacillus delbrueckii subsp. bulgaricus ATCC 11842 | 41 |
| Acidimicrobium ferrooxidans DSM 10331 | 41 |
| Bifidobacterium adolescentis ATCC 15703 chromosome | 41 |
| Haemophilus influenzae PittGG | 41 |
| Brucella suis 1330 chromosome I | 41 |
| Coxiella burnetii CbuG_Q212 chromosome | 41 |
| Candidatus Desulforudis audaxviator MP104C | 41 |
| Streptococcus pyogenes MGAS5005 chromosome | 41 |
| Burkholderia pseudomallei K96243 chromosome 2 | 41 |
| Pyrococcus abyssi GE5 chromosome | 41 |
| Burkholderia multivorans ATCC 17616 chromosome 3 | 41 |
| Streptococcus pneumoniae D39 | 41 |
| Cryptobacterium curtum DSM 15641 | 40 |
| Halobacterium salinarum R1 | 40 |
| Haemophilus somnus 129PT | 40 |
| Prochlorococcus marinus str. NATL1A | 40 |
| Coxiella burnetii RSA 493 | 40 |
| Methanococcus maripaludis C6 | 40 |
| Methanothermobacter thermautotrophicus str. Delta H chromosome | 40 |
| Neisseria meningitidis MC58 | 40 |
| Synechococcus sp. RCC307 | 40 |
| Methanocorpusculum labreanum Z chromosome | 40 |
| Aciduliprofundum boonei T469 chromosome | 39 |
| Synechococcus sp. CC9902 chromosome | 39 |
| Vibrio vulnificus CMCP6 chromosome II | 39 |
| Candidatus Amoebophilus asiaticus 5a2 chromosome | 39 |
| Sulfurimonas denitrificans DSM 1251 | 39 |
| Hyperthermus butylicus DSM 5456 chromosome | 39 |
| Neisseria meningitidis Z2491 | 39 |
| Streptococcus pyogenes MGAS9429 chromosome | 39 |
| Helicobacter hepaticus ATCC 51449 chromosome | 38 |
| Bifidobacterium animalis subsp. lactis AD011 | 38 |
| Haemophilus influenzae Rd KW20 | 38 |
| Neisseria gonorrhoeae FA 1090 | 38 |
| Pyrobaculum islandicum DSM 4184 chromosome | 38 |
| Leuconostoc mesenteroides subsp. mesenteroides ATCC 8293 | 38 |
| Burkholderia phymatum STM815 plasmid pBPHY01 | 38 |
| Campylobacter jejuni subsp. jejuni NCTC 11168 chromosome | 38 |
| Streptococcus thermophilus CNRZ1066 chromosome | 38 |
| Streptococcus pneumoniae TIGR4 chromosome | 38 |
| Ehrlichia canis str. Jake | 38 |
| Francisella tularensis subsp. tularensis FSC198 | 38 |
| Thermus thermophilus HB27 | 38 |
| Pyrobaculum aerophilum str. IM2 chromosome | 38 |
| Francisella tularensis subsp. novicida U112 | 38 |
| Streptococcus pyogenes MGAS10270 chromosome | 37 |
| Leuconostoc citreum KM20 | 37 |
| Prochlorococcus marinus str. MIT 9301 | 37 |
| Methanosphaera stadtmanae DSM 3091 chromosome | 37 |
| Vibrio cholerae O1 biovar eltor str. N16961 chromosome II | 37 |
| Coxiella burnetii RSA 331 chromosome | 37 |
| Atopobium parvulum DSM 20469 | 37 |
| Streptococcus agalactiae A909 | 37 |
| Prochlorococcus marinus str. MIT 9211 | 37 |
| Thermosipho melanesiensis BI429 | 37 |
| Sinorhizobium meliloti 1021 plasmid pSymB | 36 |
| Ehrlichia ruminantium str. Welgevonden chromosome | 36 |
| Finegoldia magna ATCC 29328 | 36 |
| Campylobacter concisus 13826 | 36 |
| Streptococcus pyogenes MGAS10394 chromosome | 36 |
| Helicobacter acinonychis str. Sheeba chromosome | 36 |
| Dehalococcoides ethenogenes 195 | 36 |
| Brucella ovis ATCC 25840 chromosome I | 36 |
| Methanococcus maripaludis S2 | 36 |
| Thermotoga lettingae TMO | 35 |
| Haemophilus influenzae PittEE | 35 |
| Brucella suis ATCC 23445 chromosome I | 35 |
| Thermofilum pendens Hrk 5 chromosome | 35 |
| Streptococcus pyogenes MGAS6180 chromosome | 35 |
| Burkholderia xenovorans LB400 chromosome 3 | 35 |
| Streptococcus pyogenes MGAS8232 | 35 |
| Aquifex aeolicus VF5 | 35 |
| Rickettsia typhi str. Wilmington | 35 |
| Sulfolobus acidocaldarius DSM 639 chromosome | 35 |
| Coprothermobacter proteolyticus DSM 5265 | 35 |
| Methanopyrus kandleri AV19 | 35 |
| Veillonella parvula DSM 2008 | 35 |
| Lactobacillus gasseri ATCC 33323 | 34 |
| Ehrlichia ruminantium str. Gardel | 34 |
| Brucella canis ATCC 23365 chromosome I | 34 |
| Aeropyrum pernix K1 | 34 |
| Photobacterium profundum SS9 chromosome 2 | 34 |
| Lactobacillus salivarius UCC118 | 34 |
| Brucella suis 1330 chromosome II | 34 |
| Anaplasma phagocytophilum HZ | 34 |
| Prochlorococcus marinus str. MIT 9312 | 34 |
| Streptobacillus moniliformis DSM 12112 | 34 |
| Thermus thermophilus HB8 | 34 |
| Methanococcus maripaludis C7 | 34 |
| Thermoplasma volcanium GSS1 chromosome | 34 |
| Methanocaldococcus jannaschii DSM 2661 chromosome | 34 |
| Campylobacter jejuni RM1221 | 34 |
| Streptococcus suis 05ZYH33 | 34 |
| Helicobacter pylori J99 | 34 |
| Prochlorococcus marinus subsp. marinus str. CCMP1375 | 34 |
| Brucella suis ATCC 23445 chromosome II | 33 |
| Chlamydia muridarum Nigg | 33 |
| Hydrogenobaculum sp. Y04AAS1 | 33 |
| Methanothermobacter marburgensis str. Marburg chromosome | 33 |
| Vibrio cholerae O395 chromosome 1 | 33 |
| Campylobacter jejuni subsp. jejuni 81116 | 33 |
| Ralstonia pickettii 12D chromosome 2 | 33 |
| Helicobacter pylori Shi470 | 33 |
| Streptococcus pneumoniae CGSP14 | 33 |
| Thermoplasma acidophilum DSM 1728 chromosome | 33 |
| Nitrosopumilus maritimus SCM1 chromosome | 33 |
| Rickettsia massiliae MTU5 | 33 |
| Haemophilus ducreyi 35000HP | 33 |
| Thermodesulfovibrio yellowstonii DSM 11347 | 32 |
| Thermotoga maritima MSB8 chromosome | 32 |
| Prochlorococcus marinus str. AS9601 | 32 |
| Prosthecochloris vibrioformis DSM 265 | 32 |
| Agrobacterium vitis S4 chromosome 2 | 32 |
| Wolbachia sp. wRi | 32 |
| Methanococcus voltae A3 chromosome | 32 |
| Methanosaeta thermophila PT chromosome | 32 |
| Pediococcus pentosaceus ATCC 25745 | 31 |
| Vibrio fischeri MJ11 chromosome II | 31 |
| Streptococcus pyogenes str. Manfredo | 31 |
| Sinorhizobium medicae WSM419 plasmid pSMED01 | 31 |
| Francisella tularensis subsp. holarctica | 31 |
| Francisella tularensis subsp. holarctica OSU18 | 31 |
| Burkholderia vietnamiensis G4 chromosome 3 | 31 |
| Francisella philomiragia subsp. philomiragia ATCC 25017 | 31 |
| Sinorhizobium meliloti 1021 plasmid pSymA | 31 |
| Rickettsia conorii str. Malish 7 | 30 |
| Brucella canis ATCC 23365 chromosome II | 30 |
| Burkholderia sp. 383 chromosome 3 | 30 |
| Helicobacter pylori HPAG1 | 30 |
| Rhodococcus jostii RHA1 plasmid pRHL1 | 30 |
| Ehrlichia ruminantium str. Welgevonden | 30 |
| Bartonella quintana str. Toulouse | 29 |
| Rickettsia canadensis str. McKiel | 29 |
| Rhizobium leguminosarum bv. trifolii WSM2304 plasmid pRLG201 | 29 |
| Mycoplasma mycoides subsp. mycoides SC str. PG1 chromosome | 29 |
| Brucella ovis ATCC 25840 chromosome II | 29 |
| Brucella abortus S19 chromosome 2 | 28 |
| Macrococcus caseolyticus JCSC5402 | 28 |
| Rhodobacter sphaeroides ATCC 17029 chromosome chromosome 2 | 28 |
| Methanobrevibacter smithii ATCC 35061 chromosome | 28 |
| Candidatus Vesicomyosocius okutanii HA | 27 |
| Chlamydophila pneumoniae AR39 | 27 |
| Azospirillum sp. B510 plasmid pAB510a | 27 |
| Burkholderia cepacia AMMD chromosome 3 | 27 |
| Polynucleobacter necessarius subsp. necessarius STIR1 | 27 |
| Prochlorococcus marinus str. MIT 9215 | 27 |
| Rickettsia rickettsii str. Iowa chromosome | 27 |
| Sinorhizobium medicae WSM419 plasmid pSMED02 | 27 |
| Dichelobacter nodosus VCS1703A | 27 |
| Prochlorococcus marinus subsp. pastoris str. CCMP1986 | 26 |
| Dehalococcoides sp. VS chromosome | 26 |
| Thermoproteus neutrophilus V24Sta chromosome | 26 |
| Campylobacter jejuni subsp. doylei 269.97 | 26 |
| Campylobacter lari RM2100 | 26 |
| Elusimicrobium minutum Pei191 | 26 |
| Chlamydophila pneumoniae J138 | 26 |
| Streptococcus thermophilus LMG 18311 chromosome | 26 |
| Candidatus Pelagibacter ubique HTCC1062 chromosome | 25 |
| Helicobacter pylori 26695 | 25 |
| Vibrio fischeri ES114 chromosome chromosome II | 25 |
| Ehrlichia chaffeensis str. Arkansas | 25 |
| Dehalococcoides sp. BAV1 | 24 |
| Burkholderia cenocepacia HI2424 chromosome 3 | 24 |
| Burkholderia cenocepacia AU 1054 chromosome 3 | 24 |
| Chlamydophila pneumoniae CWL029 | 24 |
| Methylobacterium extorquens AM1 megaplasmid | 24 |
| Anaplasma marginale str. St. Maries | 24 |
| Dehalococcoides sp. CBDB1 chromosome | 23 |
| Bartonella bacilliformis KC583 | 23 |
| Ralstonia pickettii 12J chromosome 2 | 23 |
| Rickettsia prowazekii str. Madrid E chromosome | 23 |
| Mycoplasma crocodyli MP145 chromosome | 23 |
| Brucella melitensis bv. 1 str. 16M chromosome chromosome II | 23 |
| Eubacterium eligens ATCC 27750 plasmid unnamed | 22 |
| Mycoplasma capricolum subsp. capricolum ATCC 27343 | 22 |
| Rhodobacter sphaeroides 2.4.1 chromosome 2 | 22 |
| Rickettsia felis URRWXCal2 | 22 |
| Mycoplasma agalactiae PG2 | 22 |
| Wolbachia endosymbiont of Drosophila melanogaster | 22 |
| Mycoplasma hyopneumoniae 7448 | 22 |
| Paracoccus denitrificans PD1222 plasmid 1 | 21 |
| Rhizobium leguminosarum bv. viciae 3841 plasmid pRL12 | 21 |
| Nocardiopsis dassonvillei subsp. dassonvillei DSM 43111 plasmid pNDAS01 | 21 |
| Mycoplasma pulmonis UAB CTIP | 21 |
| Candidatus Blochmannia pennsylvanicus str. BPEN | 21 |
| Candidatus Phytoplasma australiense | 21 |
| Rickettsia akari str. Hartford | 20 |
| Treponema pallidum subsp. pallidum str. Nichols chromosome | 20 |
| Burkholderia cenocepacia J2315 chromosome chromosome 3 | 20 |
| Desulfurococcus kamchatkensis 1221n chromosome | 20 |
| Neorickettsia risticii str. Illinois | 20 |
| Mycoplasma mobile 163K | 20 |
| Rhizobium etli CIAT 652 plasmid pC | 20 |
| Anaplasma marginale str. Florida | 20 |
| Ureaplasma parvum serovar 3 str. ATCC 27815 chromosome | 19 |
| Treponema pallidum subsp. pallidum SS14 | 19 |
| Chlamydophila caviae GPIC | 19 |
| Aliivibrio salmonicida LFI1238 chromosome 2 | 19 |
| Borrelia turicatae 91E135 chromosome | 19 |
| Borrelia garinii PBi chromosome chromosome linear | 19 |
| Mycoplasma hyopneumoniae J | 19 |
| Methylobacterium nodulans ORS 2060 plasmid pMNOD01 | 19 |
| Azospirillum sp. B510 plasmid pAB510b | 19 |
| Tropheryma whipplei str. Twist | 18 |
| Borrelia recurrentis A1 | 18 |
| Burkholderia phymatum STM815 plasmid pBPHY02 | 18 |
| Thermomicrobium roseum DSM 5159 plasmid unnamed | 18 |
| Brucella melitensis biovar Abortus 2308 chromosome II | 18 |
| Ureaplasma parvum serovar 3 str. ATCC 700970 | 18 |
| Borrelia duttonii Ly | 18 |
| Sphaerobacter thermophilus DSM 20745 chromosome 2 | 17 |
| Wolbachia endosymbiont strain TRS of Brugia malayi | 17 |
| Rhizobium leguminosarum bv. trifolii WSM1325 plasmid pR132503 | 17 |
| Mycoplasma genitalium G37 | 17 |
| Mycoplasma synoviae 53 | 16 |
| Borrelia hermsii DAH chromosome | 16 |
| Rhizobium etli CFN 42 plasmid p42f | 16 |
| Nostoc sp. PCC 7120 plasmid pCC7120alpha | 15 |
| Cupriavidus taiwanensis plasmid pRALTA | 15 |
| Ruegeria sp. TM1040 mega plasmid | 15 |
| Methylibium petroleiphilum PM1 plasmid RPME01 | 15 |
| Arthrobacter chlorophenolicus A6 plasmid pACHL01 | 15 |
| Blattabacterium sp. (Blattella germanica) str. Bge | 15 |
| Candidatus Phytoplasma mali | 15 |
| Rhodobacter sphaeroides ATCC 17025 plasmid pRSPA01 | 15 |
| Agrobacterium vitis S4 plasmid pAtS4e | 14 |
| Neorickettsia sennetsu str. Miyayama | 14 |
| Rhizobium leguminosarum bv. trifolii WSM1325 plasmid pR132502 | 14 |
| Agrobacterium tumefaciens str. C58 plasmid At | 14 |
| Cyanothece sp. PCC 7822 plasmid Cy782201 | 14 |
| Pseudomonas fluorescens SBW25 plasmid pQBR103 | 13 |
| Halorubrum lacusprofundi ATCC 49239 chromosome 2 | 13 |
| Borrelia afzelii PKo | 13 |
| Rhizobium leguminosarum bv. viciae 3841 plasmid pRL11 | 13 |
| Borrelia burgdorferi ZS7 | 13 |
| Rhizobium leguminosarum bv. trifolii WSM2304 plasmid pRLG202 | 13 |
| Mycoplasma pneumoniae M129 | 12 |
| Mycobacterium sp. KMS plasmid pMKMS01 | 12 |
| Mycoplasma hyopneumoniae 232 | 12 |
| Haloferax volcanii DS2 plasmid pHV4 | 12 |
| Haloferax volcanii DS2 plasmid pHV3 | 12 |
| Methylobacterium nodulans ORS 2060 plasmid pMNOD02 | 12 |
| Rhizobium etli CFN 42 plasmid p42e | 12 |
| Rhizobium sp. NGR234 plasmid pNGR234a | 12 |
| Wigglesworthia glossinidia endosymbiont of Glossina brevipalpis chromosome | 12 |
| Rhizobium etli CFN 42 symbiotic plasmid p42d | 11 |
| Halorubrum lacusprofundi ATCC 49239 plasmid pHLAC01 | 11 |
| Cyanothece sp. PCC 7822 plasmid Cy782202 | 11 |
| Anabaena variabilis ATCC 29413 plasmid C | 11 |
| Rhodococcus jostii RHA1 plasmid pRHL2 | 11 |
| Ralstonia eutropha JMP134 megaplasmid | 11 |
| Acaryochloris marina MBIC11017 plasmid pREB1 | 11 |
| Candidatus Riesia pediculicola USDA chromosome | 11 |
| Mesoplasma florum L1 | 10 |
| Bacillus cereus AH187 plasmid pAH187_270 | 10 |
| Clostridium botulinum B1 str. Okra plasmid pCLD | 10 |
| Meiothermus silvanus DSM 9946 plasmid pMESIL01 | 10 |
| Azospirillum sp. B510 plasmid pAB510c | 10 |
| Bacillus weihenstephanensis KBAB4 plasmid pBWB401 | 10 |
| Xanthobacter autotrophicus Py2 plasmid pXAUT01 | 10 |
| Agrobacterium radiobacter K84 plasmid pAtK84b | 10 |
| NC_001318 Borrelia burgdorferi B31 chromosome | 10 |
| Methylobacterium radiotolerans JCM 2831 plasmid pMRAD01 | 10 |
| Rhizobium leguminosarum bv. trifolii WSM1325 plasmid pR132501 | 10 |
| Acaryochloris marina MBIC11017 plasmid pREB2 | 10 |
| Haloarcula marismortui ATCC 43049 plasmid pNG700 | 9 |
| Sphingomonas wittichii RW1 plasmid pSWIT01 | 9 |
| Bacillus cereus E33L plasmid pE33L466 | 9 |
| Rhodobacter sphaeroides ATCC 17029 plasmid pRSPH01 | 9 |
| Rhizobium etli CIAT 652 plasmid pB | 9 |
| Nocardioides sp. JS614 plasmid pNOCA01 | 9 |
| Butyrivibrio proteoclasticus B316 plasmid pCY360 | 9 |
| Rhodococcus jostii RHA1 plasmid pRHL3 | 9 |
| Mesorhizobium loti MAFF303099 plasmid pMLa | 9 |
| Rhodobacter sphaeroides ATCC 17025 plasmid pRSPA02 | 9 |
| Arthrobacter aurescens TC1 plasmid TC1 | 9 |
| Cyanothece sp. PCC 7424 plasmid pP742401 | 8 |
| Anabaena variabilis ATCC 29413 plasmid A | 8 |
| Thermus thermophilus HB27 plasmid pTT27 | 8 |
| Mycobacterium sp. MCS plasmid1 | 8 |
| Rhodococcus erythropolis PR4 plasmid pREL1 | 8 |
| Nitrobacter hamburgensis X14 plasmid 2 | 8 |
| Rhizobium leguminosarum bv. viciae 3841 plasmid pRL9 | 8 |
| Clostridium botulinum Ba4 str. 657 plasmid pCLJ | 8 |
| Azospirillum sp. B510 plasmid pAB510e | 8 |
| Ralstonia pickettii 12D plasmid pRp12D01 | 8 |
| Caulobacter sp. K31 plasmid pCAUL01 | 8 |
| Polaromonas naphthalenivorans CJ2 plasmid pPNAP01 | 8 |
| Pseudoalteromonas haloplanktis TAC125 chromosome II | 8 |
| Ralstonia pickettii 12D plasmid pRp12D02 | 8 |
| Rhizobium leguminosarum bv. viciae 3841 plasmid pRL10 | 8 |
| Azospirillum sp. B510 plasmid pAB510d | 8 |
| Candidatus Blochmannia floridanus chromosome | 8 |
| Deinococcus geothermalis DSM 11300 plasmid pDGEO02 | 8 |
| Haloarcula marismortui ATCC 43049 chromosome II | 8 |
| Rhizobium etli CIAT 652 plasmid pA | 7 |
| Burkholderia vietnamiensis G4 plasmid pBVIE01 | 7 |
| Nostoc punctiforme PCC 73102 plasmid pNPUN01 | 7 |
| Shigella sonnei Ss046 plasmid pSS_046 | 7 |
| Nitrobacter hamburgensis X14 plasmid 1 | 7 |
| Deinococcus geothermalis DSM 11300 plasmid pDGEO01 | 7 |
| Methylobacterium chloromethanicum CM4 plasmid pMCHL01 | 7 |
| Cyanothece sp. ATCC 51142 chromosome chromosome linear | 7 |
| Dinoroseobacter shibae DFL 12 plasmid pDSHI03 | 7 |
| Cyanothece sp. PCC 7425 plasmid pP742501 | 7 |
| Herpetosiphon aurantiacus ATCC 23779 plasmid pHAU01 | 7 |
| Cupriavidus necator megaplasmid pHG1 | 7 |
| Marinobacter aquaeolei VT8 plasmid pMAQU01 | 6 |
| Bacillus pseudofirmus OF4 plasmid pBpOF4-01 | 6 |
| Polaromonas sp. JS666 plasmid 1 | 6 |
| Spirosoma linguale DSM 74 plasmid pSLIN02 | 6 |
| Polaromonas sp. JS666 plasmid 2 | 6 |
| Bacillus cereus G9842 plasmid pG9842_209 | 6 |
| Cupriavidus metallidurans CH34 plasmid pMOL30 | 6 |
| Phenylobacterium zucineum HLK1 plasmid unnamed | 6 |
| Acaryochloris marina MBIC11017 plasmid pREB4 | 6 |
| Rhizobium leguminosarum bv. trifolii WSM1325 plasmid pR132505 | 6 |
| Rhodobacter sphaeroides ATCC 17025 plasmid pRSPA03 | 6 |
| Acaryochloris marina MBIC11017 plasmid pREB7 | 6 |
| Rhizobium leguminosarum bv. trifolii WSM2304 plasmid pRLG203 | 6 |
| Thermus thermophilus HB8 plasmid pTT27 | 6 |
| Burkholderia vietnamiensis G4 plasmid pBVIE02 | 6 |
| Rhizobium leguminosarum bv. trifolii WSM1325 plasmid pR132504 | 6 |
| Acaryochloris marina MBIC11017 plasmid pREB8 | 5 |
| Burkholderia multivorans ATCC 17616 plasmid pBMUL01 | 5 |
| Acaryochloris marina MBIC11017 plasmid pREB6 | 5 |
| Mesorhizobium sp. BNC1 plasmid 1 | 5 |
| Ochrobactrum anthropi ATCC 49188 plasmid pOANT01 | 5 |
| Acaryochloris marina MBIC11017 plasmid pREB3 | 5 |
| Burkholderia phytofirmans PsJN plasmid pBPHYT01 | 5 |
| Natronomonas pharaonis DSM 2160 plasmid PL131 | 5 |
| Azoarcus sp. EbN1 plasmid 1 | 5 |
| Candidatus Carsonella ruddii PV | 5 |
| Leptospira interrogans serovar Lai str. 56601 chromosome chromosome II | 5 |
| Cyanothece sp. PCC 7822 plasmid Cy782203 | 5 |
| Nocardia farcinica IFM 10152 plasmid pNF1 | 5 |
| Azoarcus sp. EbN1 plasmid 2 | 5 |
| Nitrobacter hamburgensis X14 plasmid 3 | 5 |
| Clostridium botulinum A3 str. Loch Maree plasmid pCLK | 5 |
| Roseobacter denitrificans plasmid pTB2 | 5 |
| Salmonella enterica subsp. enterica serovar Typhi str. CT18 plasmid pHCM1 | 5 |
| Dinoroseobacter shibae DFL 12 plasmid pDSHI02 | 5 |
| Clostridium acetobutylicum ATCC 824 plasmid pSOL1 | 5 |
| Synechocystis sp. PCC 6803 plasmid pSYSX | 5 |
| Azospirillum sp. B510 plasmid pAB510f | 5 |
| Mycobacterium ulcerans AGY99 plasmid pMUM001 | 5 |
| Borrelia garinii pBi plasmid | 5 |
| Bacillus cereus AH820 plasmid pAH820_272 | 5 |
| Nostoc punctiforme PCC 73102 plasmid pNPUN02 | 5 |
| Borrelia duttonii Ly plasmid pl165 | 5 |
| Nostoc sp. PCC 7120 plasmid pCC7120beta | 5 |
| Arthrobacter sp. FB24 plasmid 2 | 4 |
| Halomicrobium mukohataei DSM 12286 plasmid pHmuk01 | 4 |
| Deinococcus radiodurans R1 chromosome 2 | 4 |
| Pseudomonas syringae pv. tomato str. DC3000 plasmid pDC3000A | 4 |
| Shewanella baltica OS195 plasmid pS19503 | 4 |
| Halobacterium salinarum R1 plasmid PHS3 | 4 |
| Mycobacterium sp. KMS plasmid pMKMS02 | 4 |
| Pseudomonas syringae pv. phaseolicola 1448A large plasmid | 4 |
| Marinobacter aquaeolei VT8 plasmid pMAQU02 | 4 |
| Meiothermus silvanus DSM 9946 plasmid pMESIL02 | 4 |
| Rhizobium leguminosarum bv. viciae 3841 plasmid pRL7 | 4 |
| Geobacter lovleyi SZ plasmid pGLOV01 | 4 |
| Herpetosiphon aurantiacus ATCC 23779 plasmid pHAU02 | 4 |
| Butyrivibrio proteoclasticus B316 plasmid pCY186 | 4 |
| Rhizobium leguminosarum bv. trifolii WSM2304 plasmid pRLG204 | 4 |
| Xenorhabdus nematophila ATCC 19061 plasmid XNC1_p | 4 |
| Mesorhizobium loti MAFF303099 plasmid pMLb | 4 |
| Agrobacterium vitis S4 plasmid pAtS4c | 4 |
| Rhodobacter sphaeroides 2.4.1 plasmid B | 4 |
| Rhodobacter sphaeroides 2.4.1 plasmid A | 4 |
| Cyanothece sp. PCC 7425 plasmid pP742502 | 4 |
| Burkholderia multivorans ATCC 17616 plasmid pTGL1 | 4 |
| Shigella flexneri 2a str. 301 plasmid pCP301 | 4 |
| Enterobacter sp. 638 plasmid pENTE01 | 4 |
| Yersinia pestis Angola plasmid pMT-pPCP | 4 |
| Candidatus Sulcia muelleri GWSS | 4 |
| Yersinia pestis Antiqua plasmid pCD | 4 |
| Yersinia pestis biovar Microtus str. 91001 plasmid pCD1 | 4 |
| Escherichia coli APEC O1 plasmid pAPEC-O1-ColBM | 4 |
| Anaerococcus prevotii DSM 20548 plasmid pAPRE01 | 4 |
| Agrobacterium radiobacter K84 plasmid pAtK84c | 4 |
| Sinorhizobium medicae WSM419 plasmid pSMED03 | 4 |
| Caulobacter sp. K31 plasmid pCAUL02 | 4 |
| Burkholderia cepacia AMMD plasmid 1 | 4 |
| Agrobacterium vitis S4 plasmid pAtS4a | 4 |
| Sphingomonas wittichii RW1 plasmid pSWIT02 | 4 |
| Gluconobacter oxydans 621H plasmid pGOX1 | 4 |
| Burkholderia vietnamiensis G4 plasmid pBVIE03 | 3 |
| Rhizobium etli CFN 42 plasmid p42b | 3 |
| Alicyclobacillus acidocaldarius subsp. acidocaldarius DSM 446 plasmid pAACI02 | 3 |
| Leuconostoc citreum KM20 plasmid pLCK2 | 3 |
| Acaryochloris marina MBIC11017 plasmid pREB5 | 3 |
| Enterococcus faecalis V583 plasmid pTEF1 | 3 |
| Pelobacter propionicus DSM 2379 plasmid pPRO1 | 3 |
| Cyanothece sp. PCC 7424 plasmid pP742402 | 3 |
| Shigella boydii Sb227 plasmid pSB4_227 | 3 |
| Vibrio fischeri MJ11 plasmid pMJ100 | 3 |
| Ochrobactrum anthropi ATCC 49188 plasmid pOANT04 | 3 |
| Deinococcus radiodurans R1 plasmid MP1 | 3 |
| Anabaena variabilis ATCC 29413 incision element | 3 |
| Acinetobacter baumannii ACICU plasmid pACICU2 | 3 |
| Aliivibrio salmonicida LFI1238 plasmid pVSAL840 | 3 |
| Arthrobacter chlorophenolicus A6 plasmid pACHL02 | 3 |
| Salmonella enterica subsp. enterica serovar Newport str. SL254 plasmid pSN254 | 3 |
| Lactobacillus salivarius UCC118 plasmid pMP118 | 3 |
| Polaromonas naphthalenivorans CJ2 plasmid pPNAP04 | 3 |
| Rhodoferax ferrireducens T118 plasmid1 | 3 |
| Haloarcula marismortui ATCC 43049 plasmid pNG600 | 3 |
| Legionella pneumophila str. Paris plasmid pLPP | 3 |
| Haloarcula marismortui ATCC 43049 plasmid pNG500 | 3 |
| Polaromonas naphthalenivorans CJ2 plasmid pPNAP03 | 3 |
| Staphylococcus epidermidis ATCC 12228 plasmid pSE-12228-06 | 3 |
| Yersinia pseudotuberculosis IP 32953 plasmid pYV | 3 |
| Yersinia pseudotuberculosis IP 31758 plasmid_153kb | 3 |
| Agrobacterium vitis S4 plasmid pAtS4b | 3 |
| Klebsiella pneumoniae 342 plasmid pKP187 | 3 |
| Anabaena variabilis ATCC 29413 plasmid B | 3 |
| Dinoroseobacter shibae DFL 12 plasmid pDSHI01 | 3 |
| Shewanella baltica OS195 plasmid pS19502 | 3 |
| Escherichia coli APEC O1 plasmid pAPEC-O1-R | 3 |
| Shewanella baltica OS155 plasmid pSbal02 | 3 |
| Novosphingobium aromaticivorans DSM 12444 plasmid pNL2 | 3 |
| Novosphingobium aromaticivorans DSM 12444 plasmid pNL1 | 3 |
| Bradyrhizobium sp. BTAi1 plasmid pBBta01 | 3 |
| Nostoc sp. PCC 7120 plasmid pCC7120gamma | 3 |
| Dinoroseobacter shibae DFL 12 plasmid pDSHI05 | 3 |
| Ruegeria sp. TM1040 plasmid unnamed | 3 |
| Kineococcus radiotolerans SRS30216 plasmid pKRAD01 | 3 |
| Klebsiella pneumoniae subsp. pneumoniae MGH 78578 plasmid pKPN3 | 3 |
| Agrobacterium vitis S4 plasmid pTiS4 | 3 |
| Bacillus cereus G9842 plasmid pG9842_140 | 3 |
| Yersinia enterocolitica subsp. enterocolitica 8081 plasmid pYVe8081 | 3 |
| Bacillus cereus Q1 plasmid pBc239 | 3 |
| Cyanothece sp. PCC 7822 plasmid Cy782205 | 3 |
| Desulfovibrio vulgaris subsp. vulgaris DP4 plasmid pDVUL01 | 3 |
| Borrelia burgdorferi B31 plasmid cp32-1 | 3 |
| Rhizobium leguminosarum bv. viciae 3841 plasmid pRL8 | 3 |
| Pseudomonas syringae pv. tomato str. DC3000 plasmid pDC3000B | 3 |
| Finegoldia magna ATCC 29328 plasmid pFMC | 2 |
| Dinoroseobacter shibae DFL 12 plasmid pDSHI04 | 2 |
| Polaromonas naphthalenivorans CJ2 plasmid pPNAP05 | 2 |
| Macrococcus caseolyticus JCSC5402 plasmid pMCCL1 | 2 |
| Shewanella baltica OS223 plasmid pS22302 | 2 |
| Shigella dysenteriae Sd197 plasmid pSD1_197 | 2 |
| Yersinia pseudotuberculosis IP 31758 plasmid_59kb | 2 |
| Macrococcus caseolyticus JCSC5402 plasmid pMCCL2 | 2 |
| Acinetobacter baumannii AYE plasmid p3ABAYE | 2 |
| Gordonia bronchialis DSM 43247 plasmid pGBRO01 | 2 |
| Bacillus weihenstephanensis KBAB4 plasmid pBWB402 | 2 |
| Borrelia burgdorferi B31 plasmid cp32-6 | 2 |
| Beijerinckia indica subsp. indica ATCC 9039 plasmid pBIND01 | 2 |
| Nocardia farcinica IFM 10152 plasmid pNF2 | 2 |
| Listeria innocua Clip11262 plasmid pLI100 | 2 |
| Alicyclobacillus acidocaldarius subsp. acidocaldarius DSM 446 plasmid pAACI01 | 2 |
| Rhizobium etli CFN 42 plasmid p42c | 2 |
| Photobacterium profundum SS9 plasmid pPBPR1 | 2 |
| Methylobacterium radiotolerans JCM 2831 plasmid pMRAD07 | 2 |
| Klebsiella pneumoniae subsp. pneumoniae MGH 78578 plasmid pKPN5 | 2 |
| Mesorhizobium sp. BNC1 plasmid 2 | 2 |
| Rhodobacter sphaeroides ATCC 17025 plasmid pRSPA04 | 2 |
| Rhodospirillum rubrum ATCC 11170 plasmid unnamed | 2 |
| Desulfotalea psychrophila LSv54 plasmid large | 2 |
| Yersinia pestis biovar Microtus str. 91001 plasmid pPCP1 | 2 |
| Cyanothece sp. PCC 8801 plasmid pP880103 | 2 |
| Synechococcus sp. PCC 7002 plasmid pAQ7 | 2 |
| Staphylococcus aureus subsp. aureus JH9 plasmid pSJH901 | 2 |
| Deinococcus radiodurans R1 plasmid CP1 | 2 |
| Yersinia pestis Angola plasmid new_pCD | 2 |
| Rickettsia felis URRWXCal2 plasmid pRF | 2 |
| Aeromonas salmonicida subsp. salmonicida A449 plasmid 5 | 2 |
| Rhodobacter sphaeroides 2.4.1 plasmid D | 2 |
| Yersinia pestis Pestoides F plasmid MT | 2 |
| Nitrosomonas eutropha C91 plasmid1 | 2 |
| Edwardsiella tarda EIB202 plasmid pEIB202 | 2 |
| Yersinia pestis Nepal516 plasmid pMT | 2 |
| Desulfohalobium retbaense DSM 5692 plasmid pDRET01 | 2 |
| Synechocystis sp. PCC 6803 plasmid pSYSA | 2 |
| Synechocystis sp. PCC 6803 plasmid pSYSM | 2 |
| Synechococcus sp. PCC 7002 plasmid pAQ6 | 2 |
| Yersinia pestis biovar Microtus str. 91001 plasmid pCRY | 2 |
| Psychrobacter cryohalolentis K5 plasmid 1 | 2 |
| Salmonella enterica subsp. enterica serovar Dublin str. CT_02021853 plasmid pCT02021853_74 | 2 |
| Salmonella enterica subsp. enterica serovar Schwarzengrund str. CVM19633 plasmid pCVM19633_110 | 2 |
| Aliivibrio salmonicida LFI1238 plasmid pVSAL54 | 2 |
| Agrobacterium tumefaciens str. C58 plasmid Ti | 2 |
| Clostridium tetani E88 plasmid pE88 | 2 |
| Yersinia pestis Pestoides F plasmid CD | 2 |
| Staphylococcus aureus subsp. aureus Mu50 plasmid VRSAp | 2 |
| Escherichia coli UTI89 plasmid pUTI89 | 2 |
| Yersinia pestis Antiqua plasmid pMT | 2 |
| Yersinia pestis CO92 plasmid pMT1 | 2 |
| Ammonifex degensii KC4 plasmid pADEG01 | 2 |
| Escherichia coli E24377A plasmid pETEC_73 | 2 |
| Roseobacter denitrificans plasmid pTB1 | 2 |
| Synechococcus sp. PCC 7002 plasmid pAQ4 | 2 |
| Nostoc punctiforme PCC 73102 plasmid pNPUN03 | 2 |
| Arthrobacter sp. FB24 plasmid 1 | 2 |
| Arthrobacter sp. FB24 plasmid 3 | 2 |
| Salmonella enterica subsp. enterica serovar Typhi str. CT18 plasmid pHCM2 | 2 |
| Burkholderia cenocepacia HI2424 plasmid 1 | 2 |
| Cupriavidus metallidurans CH34 plasmid pMOL28 | 1 |
| Escherichia coli E24377A plasmid pETEC_35 | 1 |
| Polaromonas naphthalenivorans CJ2 plasmid pPNAP06 | 1 |
| Thiomonas intermedia K12 plasmid pTINT02 | 1 |
| Pseudomonas syringae pv. phaseolicola 1448A small plasmid | 1 |
| Polaromonas naphthalenivorans CJ2 plasmid pPNAP02 | 1 |
| Rhodobacter capsulatus SB 1003 plasmid pRCB133 | 1 |
| Halobacterium salinarum R1 plasmid PHS2 | 1 |
| Natronomonas pharaonis DSM 2160 plasmid PL23 | 1 |
| Sulfolobus islandicus L.D.8.5 plasmid pLD8501 | 1 |
| Sulfolobus islandicus Y.N.15.51 plasmid pYN01 | 1 |
| Gluconobacter oxydans 621H plasmid pGOX4 | 1 |
| Vibrio vulnificus YJ016 plasmid pYJ016 | 1 |
| Gluconacetobacter diazotrophicus PAl 5 plasmid pGDIA01 | 1 |
| Burkholderia cenocepacia J2315 plasmid pBCJ2315 | 1 |
| Campylobacter lari RM2100 megaplasmid pCL2100 | 1 |
| Desulfobacterium autotrophicum HRM2 plasmid pHRM2a | 1 |
| Rickettsia felis URRWXCal2 plasmid pRFdelta | 1 |
| Aeromonas salmonicida salmonicida A449 plasmid pAsa2 | 1 |
| Aeromonas salmonicida subsp. salmonicida A449 plasmid 4 | 1 |
| Thermofilum pendens Hrk 5 plasmid pTPEN01 | 1 |
| Candidatus Riesia pediculicola USDA plasmid pPAN | 1 |
| Rhodobacter sphaeroides 2.4.1 plasmid E | 1 |
| Rhodobacter sphaeroides 2.4.1 plasmid C | 1 |
| Desulfovibrio vulgaris str. Hildenborough plasmid pDV | 1 |
| Haloarcula marismortui ATCC 43049 plasmid pNG100 | 1 |
| Methylobacterium nodulans ORS 2060 plasmid pMNOD07 | 1 |
| Ralstonia pickettii 12J plasmid pRPIC01 | 1 |
| Methanocaldococcus jannaschii DSM 2661 plasmid large ECE | 1 |
| Beijerinckia indica subsp. indica ATCC 9039 plasmid pBIND02 | 1 |
| Ochrobactrum anthropi ATCC 49188 plasmid pOANT03 | 1 |
| Ralstonia pickettii 12D plasmid pRp12D03 | 1 |
| Roseobacter denitrificans plasmid pTB3 | 1 |
| Ochrobactrum anthropi ATCC 49188 plasmid pOANT02 | 1 |
| Methanothermobacter marburgensis str. Marburg plasmid pMTBMA4 | 1 |
| Klebsiella pneumoniae 342 plasmid pKP91 | 1 |
| Methylobacterium extorquens AM1 plasmid p3META1 | 1 |
| Halobacterium salinarum R1 plasmid PHS4 | 1 |
| Burkholderia vietnamiensis G4 plasmid pBVIE05 | 1 |
| Haloferax volcanii DS2 plasmid pHV1 | 1 |
| Methylobacterium chloromethanicum CM4 plasmid pMCHL02 | 1 |
| Halobacterium salinarum R1 plasmid PHS1 | 1 |
| Methylobacterium radiotolerans JCM 2831 plasmid pMRAD05 | 1 |
| Methylobacterium radiotolerans JCM 2831 plasmid pMRAD08 | 1 |
| Ralstonia eutropha JMP134 plasmid 1 | 1 |
| Bacillus cereus E33L plasmid pE33L54 | 1 |
| Borrelia duttonii Ly plasmid pl40 | 1 |
| Borrelia duttonii Ly plasmid pl42 | 1 |
| Xanthomonas axonopodis pv. citri str. 306 plasmid pXAC64 | 1 |
| Cyanothece sp. PCC 8802 plasmid pP880204 | 1 |
| Borrelia duttonii Ly plasmid pl36 | 1 |
| Borrelia duttonii Ly plasmid pl32 | 1 |
| Borrelia duttonii Ly plasmid pl23 | 1 |
| Borrelia duttonii Ly plasmid pl23b | 1 |
| Borrelia duttonii Ly plasmid pl26 | 1 |
| Borrelia duttonii Ly plasmid pl31 | 1 |
| Agrobacterium radiobacter K84 plasmid pAgK84 | 1 |
| Cyanothece sp. PCC 7424 plasmid pP742405 | 1 |
| Synechococcus sp. PCC 7002 plasmid pAQ5 | 1 |
| Coxiella burnetii RSA 331 plasmid QpH1 | 1 |
| Sebaldella termitidis ATCC 33386 plasmid pSTERM01 | 1 |
| Xylella fastidiosa M23 plasmid pXFAS01 | 1 |
| Cyanothece sp. PCC 8801 plasmid pP880101 | 1 |
| Cyanothece sp. ATCC 51142 plasmid A | 1 |
| Cyanothece sp. PCC 7424 plasmid pP742403 | 1 |
| Xylella fastidiosa 9a5c plasmid pXF51 | 1 |
| Cyanothece sp. ATCC 51142 plasmid C | 1 |
| Cyanothece sp. ATCC 51142 plasmid B | 1 |
| Borrelia recurrentis A1 plasmid pl53 | 1 |
| Borrelia recurrentis A1 plasmid pl35 | 1 |
| Borrelia burgdorferi B31 plasmid lp56 | 1 |
| Borrelia burgdorferi plasmid cp26 | 1 |
| Borrelia burgdorferi ZS7 plasmid ZS7_cp26 | 1 |
| Borrelia burgdorferi ZS7 plasmid ZS7_cp32-1 | 1 |
| Borrelia burgdorferi B31 plasmid lp28-2 | 1 |
| Borrelia burgdorferi B31 plasmid lp17 | 1 |
| Coxiella burnetii CbuK_Q154 plasmid pQpRS_K_Q154 | 1 |
| Nitrosococcus oceani ATCC 19707 plasmid A | 1 |
| Borrelia burgdorferi B31 plasmid cp32-3 | 1 |
| Borrelia burgdorferi B31 plasmid cp32-4 | 1 |
| Borrelia burgdorferi ZS7 plasmid ZS7_cp32-12 | 1 |
| Borrelia burgdorferi ZS7 plasmid ZS7_cp32-4 | 1 |
| Borrelia afzelii PKo plasmid lp60-2 | 1 |
| Nostoc sp. PCC 7120 plasmid pCC7120delta | 1 |
| Borrelia recurrentis A1 plasmid pl124 | 1 |
| Borrelia recurrentis A1 plasmid pl33 | 1 |
| Nostoc punctiforme PCC 73102 plasmid pNPUN04 | 1 |
| Borrelia garinii PBi plasmid lp54 | 1 |
| Borrelia burgdorferi ZS7 plasmid ZS7_lp17 | 1 |
| Borrelia burgdorferi ZS7 plasmid ZS7_lp25 | 1 |
| Borrelia burgdorferi ZS7 plasmid ZS7_lp28-4 | 1 |
| Borrelia burgdorferi ZS7 plasmid ZS7_lp54 | 1 |
| Aquifex aeolicus VF5 plasmid ece1 | 1 |
| Butyrivibrio proteoclasticus B316 chromosome 2 | 1 |
| Bacillus cereus ATCC 10987 plasmid pBc10987 | 1 |
| Yersinia pestis CO92 plasmid pCD1 | 1 |
| Kineococcus radiotolerans SRS30216 plasmid pKRAD02 | 1 |
| Macrococcus caseolyticus JCSC5402 plasmid pMCCL4 | 1 |
| Shewanella baltica OS155 plasmid pSbal01 | 1 |
| Yersinia pestis KIM plasmid pMT-1 | 1 |
| Bacillus cereus E33L plasmid pE33L9 | 1 |
| Bacillus cereus E33L plasmid pE33L8 | 1 |
| Tsukamurella paurometabola DSM 20162 plasmid pTpau01 | 1 |
| Shewanella baltica OS195 plasmid pS19501 | 1 |
| Staphylococcus saprophyticus subsp. saprophyticus ATCC 15305 plasmid pSSP1 | 1 |
| Staphylococcus epidermidis ATCC 12228 plasmid pSE-12228-04 | 1 |
| Bacteroides fragilis NCTC 9343 plasmid pBF9343 | 1 |
| Yersinia pestis biovar Microtus str. 91001 plasmid pMT1 | 1 |
| Spirosoma linguale DSM 74 plasmid pSLIN05 | 1 |
| Spirosoma linguale DSM 74 plasmid pSLIN01 | 1 |
| Yersinia pestis CO92 plasmid pPCP1 | 1 |
| Bifidobacterium longum NCC2705 plasmid pBLO1 | 1 |
| Staphylococcus epidermidis RP62A plasmid pSERP | 1 |
| Streptosporangium roseum DSM 43021 plasmid pSROS01 | 1 |
| Staphylococcus aureus subsp. aureus USA300_FPR3757 plasmid pUSA03 | 1 |
| Prosthecochloris aestuarii DSM 271 plasmid pPAES01 | 1 |
| Shewanella baltica OS223 plasmid pS22301 | 1 |
| Bacillus weihenstephanensis KBAB4 plasmid pBWB403 | 1 |
| Clavibacter michiganensis subsp. michiganensis NCPPB 382 plasmid pCM2 | 1 |
| Salmonella enterica subsp. enterica serovar Agona str. SL483 plasmid unnamed | 1 |
| Clostridium botulinum B str. Eklund 17B plasmid pCLL | 1 |
| Xylanimonas cellulosilytica DSM 15894 plasmid pXCEL01 | 1 |
| Clostridium botulinum F str. Langeland plasmid pCLI | 1 |
| Clavibacter michiganensis subsp. sepedonicus plasmid pCSL1 | 1 |
| Clostridium kluyveri DSM 555 plasmid pCKL555A | 1 |
| Arthrobacter aurescens TC1 plasmid TC2 | 1 |
| Clostridium difficile 630 plasmid pCD630 | 1 |
| Salmonella enterica subsp. enterica serovar Heidelberg str. SL476 plasmid pSL476_91 | 1 |
| Salmonella typhimurium LT2 plasmid pSLT | 1 |
| Lactococcus lactis subsp. cremoris SK11 plasmid 4 | 1 |
| Lactobacillus plantarum WCFS1 plasmid pWCFS103 | 1 |
| Lactobacillus casei ATCC 334 plasmid 1 | 1 |
| Rhizobium etli CFN 42 plasmid p42a | 1 |
| Bacillus pseudofirmus OF4 plasmid pBpOF4-02 | 1 |
| Corynebacterium glutamicum R plasmid pCGR1 | 1 |
| Legionella pneumophila str. Lens plasmid pLPL | 1 |
| Rhodococcus erythropolis PR4 plasmid pREC1 | 1 |
| Lactobacillus brevis ATCC 367 plasmid 1 | 1 |
| Lactobacillus salivarius UCC118 plasmid pSF118-44 | 1 |
| Thauera sp. MZ1T plasmid pTha01 | 1 |
| *MetaSim 3* |  |
| Colwellia psychrerythraea 34H chromosome | 8640 |
| Teredinibacter turnerae T7901 chromosome | 8450 |
| Pseudoalteromonas atlantica T6c chromosome | 8255 |
| Psychromonas ingrahamii 37 chromosome | 7324 |
| Acinetobacter oleivorans DR1 chromosome | 6674 |
| Acinetobacter baumannii AYE chromosome | 6214 |
| Pseudoalteromonas haloplanktis TAC125 chromosome I | 5221 |
| Idiomarina loihiensis L2TR chromosome | 4602 |
| Canarypox virus | 1494 |
| Fowlpox virus | 1320 |
| Pseudoalteromonas haloplanktis TAC125 chromosome II | 1028 |
| Sheeppox virus 17077-99 | 618 |
| Acinetobacter baumannii AYE plasmid p3ABAYE | 139 |
| Acinetobacter baumannii AYE plasmid p2ABAYE | 12 |
| Acinetobacter baumannii AYE plasmid p1ABAYE | 5 |
| Acinetobacter baumannii AYE plasmid p4ABAYE | 4 |
| *Metasim 4* |  |
| Pseudoalteromonas atlantica T6c chromosome | 10548 |
| Teredinibacter turnerae T7901 chromosome | 10377 |
| Psychromonas ingrahamii 37 chromosome | 9163 |
| Acinetobacter oleivorans DR1 chromosome | 8456 |
| Acinetobacter baumannii AYE chromosome | 8010 |
| Pseudoalteromonas haloplanktis TAC125 chromosome I | 6464 |
| Idiomarina loihiensis L2TR chromosome | 5717 |
| Pseudoalteromonas haloplanktis TAC125 chromosome II | 1231 |
| Acinetobacter baumannii AYE plasmid p2ABAYE | 27 |
| Acinetobacter baumannii AYE plasmid p4ABAYE | 7 |
| *iMESS1* |  |
| Staphylococcus aureus | 2414 |
| Yersinia pseudotuberculosis | 1940 |
| Brucella suis | 1375 |
| Mycobacterium tuberculosis | 1206 |
| Salmonella enterica | 912 |
| Escherichia coli | 882 |
| Shigella boydii | 870 |
| Acinetobacter baumannii | 766 |
| Shigella flexneri | 682 |
| Aromatoleum aromaticum | 655 |
| Legionella pneumophila | 655 |
| Leptospira borgpetersenii | 650 |
| Desulfovibrio vulgaris | 632 |
| Corynebacterium glutamicum | 627 |
| Yersinia pestis | 607 |
| Oceanobacillus iheyensis | 581 |
| Renibacterium salmoninarum | 578 |
| Shewanella putrefaciens | 575 |
| Enterobacter sp. 638 | 523 |
| Listeria monocytogenes | 521 |
| Shewanella sp. MR-7 | 508 |
| Sodalis glossinidius | 493 |
| Azoarcus sp. BH72 | 477 |
| Parvibaculum lavamentivorans | 476 |
| Bordetella pertussis | 465 |
| Geobacter sulfurreducens | 454 |
| Geobacillus kaustophilus | 452 |
| Xylella fastidiosa | 435 |
| Geobacter metallireducens | 434 |
| Chelativorans sp. BNC1 | 431 |
| Pseudomonas stutzeri | 431 |
| Rhodopseudomonas palustris | 414 |
| Mycobacterium avium | 409 |
| Parabacteroides distasonis | 409 |
| Lysinibacillus sphaericus | 408 |
| Xanthomonas oryzae | 406 |
| Aeromonas hydrophila | 406 |
| Cytophaga hutchinsonii | 399 |
| Clostridium acetobutylicum | 398 |
| Cronobacter sakazakii | 398 |
| Novosphingobium aromaticivorans | 398 |
| Methanosarcina barkeri | 397 |
| Gloeobacter violaceus | 397 |
| Dechloromonas aromatica | 396 |
| Gluconacetobacter diazotrophicus | 393 |
| Shewanella amazonensis | 390 |
| Bordetella parapertussis | 389 |
| Bacillus licheniformis | 387 |
| Psychromonas ingrahamii | 385 |
| Mycobacterium leprae | 382 |
| Pelobacter propionicus | 379 |
| Bacillus amyloliquefaciens | 378 |
| Rhodospirillum rubrum | 378 |
| Aliivibrio fischeri | 375 |
| Desulfococcus oleovorans | 375 |
| Methylococcus capsulatus | 374 |
| Thermobifida fusca | 373 |
| Bacillus clausii | 364 |
| Shewanella sp. MR-4 | 364 |
| Synechocystis sp. PCC 6803 | 351 |
| Methanosarcina mazei | 345 |
| Enterococcus faecalis | 345 |
| Clostridium kluyveri | 336 |
| Bacillus cytotoxicus | 336 |
| Geobacter lovleyi | 332 |
| Chlorobium phaeobacteroides | 330 |
| Hyphomonas neptunium | 327 |
| Leptospira biflexa | 326 |
| Methanospirillum hungatei | 325 |
| Clostridium thermocellum | 321 |
| Methylobacillus flagellatus | 318 |
| Candidatus Protochlamydia amoebophila | 316 |
| Geobacillus thermodenitrificans | 315 |
| Chromohalobacter salexigens | 314 |
| Lactobacillus plantarum | 311 |
| Bordetella avium | 309 |
| Symbiobacterium thermophilum | 309 |
| Nitrosospira multiformis | 296 |
| Nitrosococcus oceani | 292 |
| Lactobacillus casei | 290 |
| Nitrobacter winogradskyi | 285 |
| Thermoanaerobacter pseudethanolicus | 280 |
| Synechococcus sp. JA-2-3B'a(2-13) | 280 |
| Corynebacterium efficiens | 278 |
| Alcanivorax borkumensis | 274 |
| Flavobacterium psychrophilum | 273 |
| Sulfurovum sp. NBC37-1 | 272 |
| Psychrobacter sp. PRwf-1 | 272 |
| Nitrosomonas eutropha | 264 |
| Erythrobacter litoralis | 263 |
| Chlorobium chlorochromatii | 260 |
| Listeria welshimeri | 260 |
| Nitrosomonas europaea | 259 |
| Psychrobacter cryohalolentis | 258 |
| Listeria innocua | 256 |
| Gluconobacter oxydans | 252 |
| Thiobacillus denitrificans | 251 |
| Brucella abortus | 249 |
| Pelotomaculum thermopropionicum | 248 |
| Lactococcus lactis | 245 |
| Shewanella sp. W3-18-1 | 244 |
| Halobacterium salinarum | 238 |
| Staphylococcus haemolyticus | 233 |
| Corynebacterium diphtheriae | 233 |
| Methanoregula boonei | 232 |
| Prochlorococcus marinus | 231 |
| Granulibacter bethesdensis | 224 |
| Caldanaerobacter subterraneus | 224 |
| Halorhodospira halophila | 221 |
| Thermosynechococcus elongatus | 217 |
| Synechococcus sp. WH 8102 | 212 |
| Synechococcus sp. CC9605 | 207 |
| Carboxydothermus hydrogenoformans | 198 |
| Synechococcus sp. WH 7803 | 197 |
| Porphyromonas gingivalis | 195 |
| Brucella melitensis | 177 |
| Ruegeria pomeroyi | 161 |
| Thermoanaerobacter sp. X514 | 129 |
| Brucella ovis | 64 |
| Brucella canis | 33 |
| Staphylococcus epidermidis | 28 |
| Citrobacter koseri | 5 |
| Bacillus pumilus | 4 |
| Acidovorax sp. JS42 | 1 |
| Mycobacterium bovis | 1 |
| *iMESS2* |  |
| Staphylococcus aureus | 2348 |
| Yersinia pseudotuberculosis | 2121 |
| Salmonella enterica | 1970 |
| Mycobacterium tuberculosis | 1513 |
| Brucella suis | 1307 |
| Xanthomonas oryzae | 974 |
| Shigella boydii | 947 |
| Shigella flexneri | 690 |
| Acinetobacter baumannii | 688 |
| Corynebacterium glutamicum | 635 |
| Escherichia coli | 617 |
| Listeria monocytogenes | 490 |
| Bordetella pertussis | 482 |
| Sodalis glossinidius | 475 |
| Shewanella loihica | 466 |
| Yersinia pestis | 452 |
| Thermobifida fusca | 449 |
| Dechloromonas aromatica | 448 |
| Yersinia enterocolitica | 446 |
| Leptospira interrogans | 437 |
| Psychromonas ingrahamii | 437 |
| Chlorobium phaeobacteroides | 431 |
| Prochlorococcus marinus | 427 |
| Clostridium phytofermentans | 425 |
| Rhodopseudomonas palustris | 424 |
| Bacillus cytotoxicus | 422 |
| Parabacteroides distasonis | 420 |
| Geobacter metallireducens | 418 |
| Lactococcus lactis | 418 |
| Shewanella sp. MR-4 | 415 |
| Albidiferax ferrireducens | 409 |
| Aromatoleum aromaticum | 404 |
| Gloeobacter violaceus | 403 |
| Cronobacter sakazakii | 401 |
| Clostridium acetobutylicum | 400 |
| Methylibium petroleiphilum | 400 |
| Leptothrix cholodnii | 400 |
| Azoarcus sp. BH72 | 399 |
| Jannaschia sp. CCS1 | 398 |
| Aliivibrio fischeri | 394 |
| Beijerinckia indica | 392 |
| Geobacter sulfurreducens | 389 |
| Enterobacter sp. 638 | 389 |
| Chromohalobacter salexigens | 388 |
| Erwinia tasmaniensis | 380 |
| Rhodobacter sphaeroides | 379 |
| Methanosarcina mazei | 378 |
| Legionella pneumophila | 371 |
| Shewanella denitrificans | 370 |
| Geobacter lovleyi | 367 |
| Roseobacter denitrificans | 362 |
| Novosphingobium aromaticivorans | 360 |
| Psychrobacter cryohalolentis | 354 |
| Ruegeria sp. TM1040 | 352 |
| Bordetella avium | 351 |
| Vibrio cholerae | 348 |
| Desulfococcus oleovorans | 348 |
| Pelobacter carbinolicus | 346 |
| Caulobacter vibrioides | 345 |
| Halobacterium salinarum | 339 |
| Janthinobacterium sp. Marseille | 335 |
| Leptospira biflexa | 334 |
| Gluconacetobacter diazotrophicus | 334 |
| Synechocystis sp. PCC 6803 | 326 |
| Thiomicrospira crunogena | 320 |
| Desulfovibrio vulgaris | 317 |
| Pseudoalteromonas haloplanktis | 316 |
| Methylobacillus flagellatus | 315 |
| Desulfotalea psychrophila | 311 |
| Bacillus pumilus | 310 |
| Sphingopyxis alaskensis | 309 |
| Gramella forsetii | 308 |
| Hyphomonas neptunium | 307 |
| Bdellovibrio bacteriovorus | 307 |
| Desulfotomaculum reducens | 296 |
| Maricaulis maris | 294 |
| Psychrobacter sp. PRwf-1 | 293 |
| Renibacterium salmoninarum | 293 |
| Alcanivorax borkumensis | 292 |
| Nitrosococcus oceani | 291 |
| Geobacillus kaustophilus | 291 |
| Caldanaerobacter subterraneus | 289 |
| Alkaliphilus oremlandii | 285 |
| Synechococcus sp. PCC 7002 | 282 |
| Mycobacterium leprae | 281 |
| Idiomarina loihiensis | 280 |
| Oceanobacillus iheyensis | 278 |
| Exiguobacterium sibiricum | 275 |
| Listeria welshimeri | 272 |
| Deinococcus geothermalis | 270 |
| Gluconobacter oxydans | 265 |
| Listeria innocua | 260 |
| Thiobacillus denitrificans | 259 |
| Syntrophomonas wolfei | 257 |
| Shewanella sp. W3-18-1 | 242 |
| Granulibacter bethesdensis | 240 |
| Synechococcus elongatus | 232 |
| Flavobacterium psychrophilum | 231 |
| Treponema denticola | 230 |
| Nitrosomonas eutropha | 229 |
| Staphylococcus epidermidis | 226 |
| Halorhodospira halophila | 219 |
| Psychrobacter arcticus | 217 |
| Synechococcus sp. CC9605 | 216 |
| Corynebacterium jeikeium | 214 |
| Thermosynechococcus elongatus | 212 |
| Chlorobium chlorochromatii | 209 |
| Streptococcus sanguinis | 208 |
| Methanococcoides burtonii | 207 |
| Xylella fastidiosa | 207 |
| Corynebacterium diphtheriae | 205 |
| Mycobacterium avium | 204 |
| Porphyromonas gingivalis | 203 |
| Methanoculleus marisnigri | 201 |
| Bifidobacterium longum | 199 |
| Candidatus Protochlamydia amoebophila | 198 |
| Synechococcus sp. WH 7803 | 197 |
| Shewanella putrefaciens | 182 |
| Brucella ovis | 179 |
| Brucella abortus | 153 |
| Brucella melitensis | 147 |
| Shewanella sp. MR-7 | 50 |
| Mycobacterium bovis | 37 |
| Brucella canis | 22 |
| Staphylococcus haemolyticus | 14 |
| Citrobacter koseri | 7 |
| Shewanella amazonensis | 3 |
| Pelotomaculum thermopropionicum | 1 |
| Bordetella parapertussis | 1 |
| Corynebacterium efficiens | 1 |
| Lysinibacillus sphaericus | 1 |
| Bacillus amyloliquefaciens | 1 |
| Aeromonas hydrophila | 1 |
| Pseudomonas stutzeri | 1 |
| Clostridium thermocellum | 1 |

Supplementary Table S4: List of genomes used to build BLAST database and to train Phymm and NBC.

| **Genome** | **Accession numbers** |
| --- | --- |
| Bacteria |  |
| Acaryochloris marina MBIC11017 | NC_009925 NC_009926 NC_009927 NC_009928 NC_009929 NC_009930 NC_009931 NC_009932 NC_009933 NC_009934 |
| Acetobacter pasteurianus IFO 3283-01 | NC_013209 NC_013210 NC_013211 NC_013212 NC_013213 NC_013214 NC_013215 |
| Acetohalobium arabaticum DSM 5501 | NC_014378 |
| Acholeplasma laidlawii PG-8A | NC_010163 |
| Achromobacter xylosoxidans A8 | NC_014640 NC_014641 NC_014642 |
| Acidaminococcus fermentans DSM 20731 | NC_013740 |
| Acidilobus saccharovorans 345-15 | NC_014374 |
| Acidimicrobium ferrooxidans DSM 10331 | NC_013124 |
| Acidiphilium cryptum JF-5 | NC_009467 NC_009468 NC_009469 NC_009470 NC_009471 NC_009472 NC_009473 NC_009474 NC_009484 |
| Acidithiobacillus ferrooxidans ATCC 23270 | NC_011761 |
| Acidithiobacillus ferrooxidans ATCC 53993 | NC_011206 |
| Acidobacterium capsulatum ATCC 51196 | NC_012483 |
| Acidothermus cellulolyticus 11B | NC_008578 |
| Acidovorax citrulli AAC00-1 | NC_008752 |
| Acidovorax ebreus TPSY | NC_011992 |
| Acidovorax sp. JS42 | NC_008765 NC_008766 NC_008782 |
| Aciduliprofundum boonei T469 | NC_013926 |
| Acinetobacter baumannii AB0057 | NC_011585 NC_011586 |
| Acinetobacter baumannii AB307-0294 | NC_011595 |
| Acinetobacter baumannii ACICU | NC_010605 NC_010606 NC_010611 |
| Acinetobacter baumannii ATCC 17978 | NC_009083 NC_009084 NC_009085 |
| Acinetobacter baumannii AYE | NC_010401 NC_010402 NC_010403 NC_010404 NC_010410 |
| Acinetobacter baumannii SDF | NC_010395 NC_010396 NC_010398 NC_010400 |
| Acinetobacter sp. ADP1 | NC_005966 |
| Acinetobacter sp. DR1 | NC_014259 |
| Actinobacillus pleuropneumoniae serovar 3 str. JL03 | NC_010278 |
| Actinobacillus pleuropneumoniae serovar 5b str. L20 | NC_009053 |
| Actinobacillus pleuropneumoniae serovar 7 str. AP76 | NC_010939 NC_010940 NC_010941 NC_010942 |
| Actinobacillus succinogenes 130Z | NC_009655 |
| Actinosynnema mirum DSM 43827 | NC_013093 |
| Aeromonas hydrophila subsp. hydrophila ATCC 7966 | NC_008570 |
| Aeromonas salmonicida subsp. salmonicida A449 | NC_004923 NC_004924 NC_004925 NC_009348 NC_009349 NC_009350 |
| Aeropyrum pernix K1 | NC_000854 |
| Aggregatibacter actinomycetemcomitans D11S-1 | NC_013416 NC_013438 NC_013597 NC_014629 |
| Aggregatibacter aphrophilus NJ8700 | NC_012913 |
| Agrobacterium radiobacter K84 | NC_011983 NC_011985 NC_011987 NC_011990 NC_011994 |
| Agrobacterium tumefaciens str. C58 | NC_003062 NC_003063 NC_003064 NC_003065 |
| Agrobacterium vitis S4 | NC_011981 NC_011982 NC_011984 NC_011986 NC_011988 NC_011989 NC_011991 |
| Akkermansia muciniphila ATCC BAA-835 | NC_010655 |
| Alcanivorax borkumensis SK2 | NC_008260 |
| Alicyclobacillus acidocaldarius subsp. acidocaldarius DSM 446 | NC_013205 NC_013206 NC_013207 NC_013208 |
| Aliivibrio salmonicida LFI1238 | NC_011311 NC_011312 NC_011313 NC_011314 NC_011315 NC_011316 |
| Alkalilimnicola ehrlichii MLHE-1 | NC_008340 |
| Alkaliphilus metalliredigens QYMF | NC_009633 |
| Alkaliphilus oremlandii OhILAs | NC_009922 |
| Allochromatium vinosum DSM 180 | NC_013851 NC_013852 NC_013862 |
| Alteromonas macleodii SINGLEQUOTEDeep ecotypeSINGLEQUOTE | NC_011138 |
| Aminobacterium colombiense DSM 12261 | NC_014011 |
| Ammonifex degensii KC4 | NC_013385 NC_013386 |
| Amycolatopsis mediterranei U32 | NC_014318 |
| Anabaena variabilis ATCC 29413 LPARENAnabaena flos-aquae UTEX 1444RPAREN | NC_007410 NC_007411 NC_007412 NC_007413 NC_014000 |
| Anaerococcus prevotii DSM 20548 | NC_013164 NC_013171 |
| Anaeromyxobacter dehalogenans 2CP-1 | NC_011891 |
| Anaeromyxobacter dehalogenans 2CP-C | NC_007760 |
| Anaeromyxobacter sp. Fw109-5 | NC_009675 |
| Anaeromyxobacter sp. K | NC_011145 |
| Anaplasma centrale str. Israel LPARENAnaplasma marginale subsp. centrale str. IsraelRPAREN | NC_013532 |
| Anaplasma marginale str. Florida | NC_012026 |
| Anaplasma marginale str. St. Maries | NC_004842 |
| Anaplasma phagocytophilum HZ | NC_007797 |
| Anoxybacillus flavithermus WK1 | NC_011567 |
| Aquifex aeolicus VF5 | NC_000918 NC_001880 |
| Arcanobacterium haemolyticum DSM 20595 | NC_014218 |
| Archaeoglobus fulgidus DSM 4304 | NC_000917 |
| Archaeoglobus profundus DSM 5631 | NC_013741 NC_013742 |
| Arcobacter butzleri RM4018 | NC_009850 |
| Arcobacter nitrofigilis DSM 7299 | NC_014166 |
| Aromatoleum aromaticum EbN1 | NC_006513 NC_006823 NC_006824 |
| Arthrobacter arilaitensis Re117 | NC_014548 NC_014549 NC_014550 |
| Arthrobacter aurescens TC1 | NC_008711 NC_008712 NC_008713 |
| Arthrobacter chlorophenolicus A6 | NC_011879 NC_011881 NC_011886 |
| Arthrobacter sp. FB24 | NC_008537 NC_008538 NC_008539 NC_008541 |
| Aster yellows witchesSINGLEQUOTE-broom phytoplasma AYWB | NC_007716 NC_007717 NC_007718 NC_007719 NC_007720 |
| Asticcacaulis excentricus CB 48 | NC_014816 NC_014817 NC_014818 NC_014819 |
| Atopobium parvulum DSM 20469 | NC_013203 |
| Azoarcus sp. BH72 | NC_008702 |
| Azorhizobium caulinodans ORS 571 | NC_009937 |
| Azospirillum sp. B510 | NC_013854 NC_013855 NC_013856 NC_013857 NC_013858 NC_013859 NC_013860 |
| Azotobacter vinelandii DJ | NC_012560 |
| Bacillus amyloliquefaciens DSM7 | NC_014551 |
| Bacillus amyloliquefaciens FZB42 | NC_009725 |
| Bacillus anthracis str. A0248 | NC_012655 NC_012656 NC_012659 |
| Bacillus anthracis str. Ames | NC_003997 |
| Bacillus anthracis str. CDC 684 | NC_012577 NC_012579 NC_012581 |
| Bacillus anthracis str. SINGLEQUOTEAmes AncestorSINGLEQUOTE | NC_007322 NC_007323 NC_007530 |
| Bacillus anthracis str. Sterne | NC_005945 |
| Bacillus atrophaeus 1942 | NC_014639 |
| Bacillus cereus 03BB102 | NC_012472 NC_012473 |
| Bacillus cereus AH187 | NC_011654 NC_011655 NC_011656 NC_011657 NC_011658 |
| Bacillus cereus AH820 | NC_011771 NC_011773 NC_011776 NC_011777 |
| Bacillus cereus ATCC 10987 | NC_003909 NC_005707 |
| Bacillus cereus ATCC 14579 | NC_004721 NC_004722 |
| Bacillus cereus B4264 | NC_011725 |
| Bacillus cereus E33L | NC_006274 NC_007103 NC_007104 NC_007105 NC_007106 NC_007107 |
| Bacillus cereus G9842 | NC_011772 NC_011774 NC_011775 |
| Bacillus cereus Q1 | NC_011969 NC_011971 NC_011973 |
| Bacillus cereus biovar anthracis str. CI | NC_014331 NC_014332 NC_014333 NC_014335 |
| Bacillus clausii KSM-K16 | NC_006582 |
| Bacillus cytotoxicus NVH 391-98 | NC_009673 NC_009674 |
| Bacillus halodurans C-125 | NC_002570 |
| Bacillus licheniformis ATCC 14580 LPARENDSM 13RPAREN | NC_006270 NC_006322 |
| Bacillus megaterium DSM 319 | NC_014103 |
| Bacillus megaterium QM B1551 | NC_004604 NC_010008 NC_010009 NC_010010 NC_014019 NC_014023 NC_014025 NC_014031 |
| Bacillus pseudofirmus OF4 | NC_013791 NC_013792 NC_013793 |
| Bacillus pumilus SAFR-032 | NC_009848 |
| Bacillus selenitireducens MLS10 | NC_014219 |
| Bacillus subtilis subsp. spizizenii str. W23 | NC_014479 |
| Bacillus subtilis subsp. subtilis str. 168 | NC_000964 |
| Bacillus thuringiensis BMB171 | NC_014171 NC_014172 |
| Bacillus thuringiensis serovar konkukian str. 97-27 | NC_005957 NC_006578 |
| Bacillus thuringiensis str. Al Hakam | NC_008598 NC_008600 |
| Bacillus tusciae DSM 2912 | NC_014098 |
| Bacillus weihenstephanensis KBAB4 | NC_010180 NC_010181 NC_010182 NC_010183 NC_010184 |
| Bacteroides fragilis NCTC 9343 | NC_003228 NC_006873 |
| Bacteroides fragilis YCH46 | NC_006297 NC_006347 |
| Bacteroides thetaiotaomicron VPI-5482 | NC_004663 NC_004703 |
| Bacteroides vulgatus ATCC 8482 | NC_009614 |
| Bartonella bacilliformis KC583 | NC_008783 |
| Bartonella grahamii as4aup | NC_012846 NC_012847 |
| Bartonella henselae str. Houston-1 | NC_005956 |
| Bartonella quintana str. Toulouse | NC_005955 |
| Bartonella tribocorum CIP 105476 | NC_010160 NC_010161 |
| Baumannia cicadellinicola str. Hc LPARENHomalodisca coagulataRPAREN | NC_007984 |
| Bdellovibrio bacteriovorus HD100 | NC_005363 |
| Beijerinckia indica subsp. indica ATCC 9039 | NC_010578 NC_010580 NC_010581 |
| Beutenbergia cavernae DSM 12333 | NC_012669 |
| Bifidobacterium adolescentis ATCC 15703 | NC_008618 |
| Bifidobacterium animalis subsp. lactis AD011 | NC_011835 |
| Bifidobacterium animalis subsp. lactis Bl-04 | NC_012814 |
| Bifidobacterium animalis subsp. lactis DSM 10140 | NC_012815 |
| Bifidobacterium bifidum PRL2010 | NC_014638 |
| Bifidobacterium bifidum S17 | NC_014616 |
| Bifidobacterium dentium Bd1 | NC_013714 |
| Bifidobacterium longum DJO10A | NC_004252 NC_004253 NC_010816 |
| Bifidobacterium longum NCC2705 | NC_004307 NC_004943 |
| Bifidobacterium longum subsp. infantis ATCC 15697 | NC_011593 |
| Bifidobacterium longum subsp. longum BBMN68 | NC_014656 |
| Bifidobacterium longum subsp. longum JDM301 | NC_014169 |
| Blattabacterium sp. LPARENBlattella germanicaRPAREN str. Bge | NC_013454 |
| Blattabacterium sp. LPARENPeriplaneta americanaRPAREN str. BPLAN | NC_013418 NC_013419 |
| Bordetella avium 197N | NC_010645 |
| Bordetella bronchiseptica RB50 | NC_002927 |
| Bordetella parapertussis 12822 | NC_002928 |
| Bordetella pertussis Tohama I | NC_002929 |
| Bordetella petrii DSM 12804 | NC_010170 |
| Borrelia afzelii PKo | NC_008273 NC_008274 NC_008277 NC_008564 NC_008565 NC_008566 NC_008567 NC_008568 NC_008569 |
| Borrelia burgdorferi B31 | NC_000948 NC_000949 NC_000950 NC_000951 NC_000952 NC_000953 NC_000954 NC_000955 NC_000956 NC_000957 NC_001318 NC_001849 NC_001850 NC_001851 NC_001852 NC_001853 NC_001854 NC_001855 NC_001856 NC_001857 NC_001903 NC_001904 |
| Borrelia burgdorferi ZS7 | NC_011720 NC_011722 NC_011724 NC_011728 NC_011731 NC_011735 NC_011736 NC_011778 NC_011779 NC_011780 NC_011781 NC_011782 NC_011783 NC_011784 NC_011785 |
| Borrelia duttonii Ly | NC_011224 NC_011226 NC_011229 NC_011245 NC_011247 NC_011248 NC_011249 NC_011250 NC_011251 NC_011254 NC_011256 NC_011257 NC_011259 NC_011261 NC_011262 NC_011264 NC_011265 |
| Borrelia garinii PBi | NC_006128 NC_006129 NC_006156 |
| Borrelia hermsii DAH | NC_010673 |
| Borrelia recurrentis A1 | NC_011244 NC_011246 NC_011252 NC_011253 NC_011255 NC_011258 NC_011260 NC_011263 |
| Borrelia turicatae 91E135 | NC_008710 |
| Brachybacterium faecium DSM 4810 | NC_013172 |
| Brachyspira hyodysenteriae WA1 | NC_012225 NC_012226 |
| Brachyspira murdochii DSM 12563 | NC_014150 |
| Brachyspira pilosicoli 95SLASH1000 | NC_014330 |
| Bradyrhizobium japonicum USDA 110 | NC_004463 |
| Bradyrhizobium sp. BTAi1 | NC_009475 NC_009485 |
| Bradyrhizobium sp. ORS278 | NC_009445 |
| Brevibacillus brevis NBRC 100599 | NC_012491 |
| Brevundimonas subvibrioides ATCC 15264 | NC_014375 |
| Brucella abortus S19 | NC_010740 NC_010742 |
| Brucella abortus bv. 1 str. 9-941 | NC_006932 NC_006933 |
| Brucella canis ATCC 23365 | NC_010103 NC_010104 |
| Brucella melitensis ATCC 23457 | NC_012441 NC_012442 |
| Brucella melitensis biovar Abortus 2308 | NC_007618 NC_007624 |
| Brucella melitensis bv. 1 str. 16M | NC_003317 NC_003318 |
| Brucella microti CCM 4915 | NC_013118 NC_013119 |
| Brucella ovis ATCC 25840 | NC_009504 NC_009505 |
| Brucella suis 1330 | NC_004310 NC_004311 |
| Brucella suis ATCC 23445 | NC_010167 NC_010169 |
| Buchnera aphidicola str. 5A LPARENAcyrthosiphon pisumRPAREN | NC_011833 |
| Buchnera aphidicola str. APS LPARENAcyrthosiphon pisumRPAREN | NC_002252 NC_002253 NC_002528 |
| Buchnera aphidicola str. Bp LPARENBaizongia pistaciaeRPAREN | NC_004545 NC_004555 |
| Buchnera aphidicola str. Cc LPARENCinara cedriRPAREN | NC_008513 NC_011878 |
| Buchnera aphidicola str. Sg LPARENSchizaphis graminumRPAREN | NC_004061 |
| Buchnera aphidicola str. Tuc7 LPARENAcyrthosiphon pisumRPAREN | NC_011834 |
| Burkholderia ambifaria AMMD | NC_008385 NC_008390 NC_008391 NC_008392 |
| Burkholderia ambifaria MC40-6 | NC_010551 NC_010552 NC_010553 NC_010557 |
| Burkholderia cenocepacia AU 1054 | NC_008060 NC_008061 NC_008062 |
| Burkholderia cenocepacia HI2424 | NC_008542 NC_008543 NC_008544 NC_008545 |
| Burkholderia cenocepacia J2315 | NC_011000 NC_011001 NC_011002 NC_011003 |
| Burkholderia cenocepacia MC0-3 | NC_010508 NC_010512 NC_010515 |
| Burkholderia glumae BGR1 | NC_012718 NC_012720 NC_012721 NC_012723 NC_012724 NC_012725 |
| Burkholderia mallei ATCC 23344 | NC_006348 NC_006349 |
| Burkholderia mallei NCTC 10229 | NC_008835 NC_008836 |
| Burkholderia mallei NCTC 10247 | NC_009079 NC_009080 |
| Burkholderia mallei SAVP1 | NC_008784 NC_008785 |
| Burkholderia multivorans ATCC 17616 | NC_010070 NC_010084 NC_010086 NC_010087 NC_010801 NC_010802 NC_010804 NC_010805 |
| Burkholderia phymatum STM815 | NC_010622 NC_010623 NC_010625 NC_010627 |
| Burkholderia phytofirmans PsJN | NC_010676 NC_010679 NC_010681 |
| Burkholderia pseudomallei 1106a | NC_009076 NC_009078 |
| Burkholderia pseudomallei 1710b | NC_007434 NC_007435 |
| Burkholderia pseudomallei 668 | NC_009074 NC_009075 |
| Burkholderia pseudomallei K96243 | NC_006350 NC_006351 |
| Burkholderia pseudomallei MSHR346 | NC_012695 |
| Burkholderia rhizoxinica HKI 454 | NC_014718 NC_014722 |
| Burkholderia sp. 383 | NC_007509 NC_007510 NC_007511 |
| Burkholderia sp. CCGE1002 | NC_014117 NC_014118 NC_014119 NC_014120 |
| Burkholderia sp. CCGE1003 | NC_014539 NC_014540 |
| Burkholderia thailandensis E264 | NC_007650 NC_007651 |
| Burkholderia vietnamiensis G4 | NC_009226 NC_009227 NC_009228 NC_009229 NC_009230 NC_009254 NC_009255 NC_009256 |
| Burkholderia xenovorans LB400 | NC_007951 NC_007952 NC_007953 |
| Butyrivibrio proteoclasticus B316 | NC_014387 NC_014388 NC_014389 NC_014390 |
| Caldicellulosiruptor bescii DSM 6725 | NC_012034 NC_012036 NC_012037 |
| Caldicellulosiruptor hydrothermalis 108 | NC_014652 |
| Caldicellulosiruptor kristjanssonii 177R1B | NC_014719 NC_014721 |
| Caldicellulosiruptor kronotskyensis 2002 | NC_014720 |
| Caldicellulosiruptor obsidiansis OB47 | NC_014392 |
| Caldicellulosiruptor owensensis OL | NC_014657 |
| Caldicellulosiruptor saccharolyticus DSM 8903 | NC_009437 |
| Calditerrivibrio nitroreducens DSM 19672 | NC_014749 NC_014758 |
| Caldivirga maquilingensis IC-167 | NC_009954 |
| Campylobacter concisus 13826 | NC_009795 NC_009796 NC_009802 |
| Campylobacter curvus 525.92 | NC_009715 |
| Campylobacter fetus subsp. fetus 82-40 | NC_008599 |
| Campylobacter hominis ATCC BAA-381 | NC_009713 NC_009714 |
| Campylobacter jejuni RM1221 | NC_003912 |
| Campylobacter jejuni subsp. doylei 269.97 | NC_009707 |
| Campylobacter jejuni subsp. jejuni 81-176 | NC_008770 NC_008787 NC_008790 |
| Campylobacter jejuni subsp. jejuni 81116 | NC_009839 |
| Campylobacter jejuni subsp. jejuni ICDCCJ07001 | NC_014801 NC_014802 |
| Campylobacter jejuni subsp. jejuni NCTC 11168 | NC_002163 |
| Campylobacter lari RM2100 | NC_012039 NC_012040 |
| Candidatus Accumulibacter phosphatis clade IIA str. UW-1 | NC_013190 NC_013191 NC_013193 NC_013194 |
| Candidatus Amoebophilus asiaticus 5a2 | NC_010830 |
| Candidatus Azobacteroides pseudotrichonymphae genomovar. CFP2 | NC_011561 NC_011562 NC_011563 NC_011564 NC_011565 |
| Candidatus Blochmannia floridanus | NC_005061 |
| Candidatus Blochmannia pennsylvanicus str. BPEN | NC_007292 |
| Candidatus Carsonella ruddii PV | NC_008512 |
| Candidatus Desulforudis audaxviator MP104C | NC_010424 |
| Candidatus Hamiltonella defensa 5AT LPARENAcyrthosiphon pisumRPAREN | NC_012751 NC_012752 |
| Candidatus Hodgkinia cicadicola Dsem | NC_012960 |
| Candidatus Korarchaeum cryptofilum OPF8 | NC_010482 |
| Candidatus Koribacter versatilis Ellin345 | NC_008009 |
| Candidatus Liberibacter asiaticus str. psy62 | NC_012985 |
| Candidatus Liberibacter solanacearum CLso-ZC1 | NC_014774 |
| Candidatus Methanoregula boonei 6A8 | NC_009712 |
| Candidatus Nitrospira defluvii | NC_014355 |
| Candidatus Pelagibacter ubique HTCC1062 | NC_007205 |
| Candidatus Phytoplasma australiense | NC_010544 |
| Candidatus Phytoplasma mali | NC_011047 |
| Candidatus Protochlamydia amoebophila UWE25 | NC_005861 |
| Candidatus Puniceispirillum marinum IMCC1322 | NC_014010 |
| Candidatus Riesia pediculicola USDA | NC_013962 NC_014109 |
| Candidatus Ruthia magnifica str. Cm LPARENCalyptogena magnificaRPAREN | NC_008610 |
| Candidatus Solibacter usitatus Ellin6076 | NC_008536 |
| Candidatus Sulcia muelleri CARI | NC_014499 |
| Candidatus Sulcia muelleri DMIN | NC_014004 |
| Candidatus Sulcia muelleri GWSS | NC_010118 |
| Candidatus Sulcia muelleri SMDSEM | NC_013123 |
| Candidatus Vesicomyosocius okutanii HA LPARENCandidatus Vesicomyosocius okutanii str. HARPAREN | NC_009465 |
| Candidatus Zinderia insecticola CARI | NC_014497 |
| Capnocytophaga ochracea DSM 7271 | NC_013162 |
| Carboxydothermus hydrogenoformans Z-2901 | NC_007503 |
| Catenulispora acidiphila DSM 44928 | NC_013131 |
| Caulobacter crescentus CB15 | NC_002696 |
| Caulobacter crescentus NA1000 | NC_011916 |
| Caulobacter segnis ATCC 21756 | NC_014100 |
| Caulobacter sp. K31 | NC_010333 NC_010335 NC_010338 |
| Cellulomonas flavigena DSM 20109 | NC_014151 |
| Cellvibrio japonicus Ueda107 | NC_010995 |
| Cenarchaeum symbiosum A | NC_014820 |
| Chelativorans sp. BNC1 | NC_008242 NC_008243 NC_008244 NC_008254 |
| Chitinophaga pinensis DSM 2588 | NC_013132 |
| Chlamydia muridarum Nigg | NC_002182 NC_002620 |
| Chlamydia trachomatis 434SLASHBu | NC_010287 |
| Chlamydia trachomatis ASLASHHAR-13 | NC_007429 NC_007430 |
| Chlamydia trachomatis BSLASHJali20SLASHOT | NC_012686 |
| Chlamydia trachomatis BSLASHTZ1A828SLASHOT | NC_012687 |
| Chlamydia trachomatis DSLASHUW-3SLASHCX | NC_000117 |
| Chlamydia trachomatis L2bSLASHUCH-1SLASHproctitis | NC_010280 |
| Chlamydophila abortus S26SLASH3 | NC_004552 |
| Chlamydophila caviae GPIC | NC_003361 NC_004720 |
| Chlamydophila felis FeSLASHC-56 | NC_007899 NC_007900 |
| Chlamydophila pneumoniae AR39 | NC_002179 NC_002180 |
| Chlamydophila pneumoniae CWL029 | NC_000922 |
| Chlamydophila pneumoniae J138 | NC_002491 |
| Chlamydophila pneumoniae TW-183 | NC_005043 |
| Chlorobaculum parvum NCIB 8327 | NC_011027 |
| Chlorobium chlorochromatii CaD3 | NC_007514 |
| Chlorobium limicola DSM 245 | NC_010803 |
| Chlorobium luteolum DSM 273 | NC_007512 |
| Chlorobium phaeobacteroides BS1 | NC_010831 |
| Chlorobium phaeobacteroides DSM 266 | NC_008639 |
| Chlorobium phaeovibrioides DSM 265 LPARENProsthecochloris vibrioformis DSM 265RPAREN | NC_009337 |
| Chlorobium tepidum TLS | NC_002932 |
| Chloroflexus aggregans DSM 9485 | NC_011831 |
| Chloroflexus aurantiacus J-10-fl | NC_010175 |
| Chloroflexus sp. Y-400-fl | NC_012032 |
| Chloroherpeton thalassium ATCC 35110 | NC_011026 |
| Chromobacterium violaceum ATCC 12472 | NC_005085 |
| Chromohalobacter salexigens DSM 3043 | NC_007963 |
| Citrobacter koseri ATCC BAA-895 | NC_009792 NC_009793 NC_009794 |
| Citrobacter rodentium ICC168 | NC_013716 NC_013717 NC_013718 NC_013719 |
| Clavibacter michiganensis subsp. michiganensis NCPPB 382 | NC_009478 NC_009479 NC_009480 |
| Clavibacter michiganensis subsp. sepedonicus | NC_010399 NC_010407 NC_010408 |
| Clostridiales genomosp. BVAB3 str. UPII9-5 | NC_013895 |
| Clostridium acetobutylicum ATCC 824 | NC_001988 NC_003030 |
| Clostridium beijerinckii NCIMB 8052 | NC_009617 |
| Clostridium botulinum A2 str. Kyoto | NC_012563 |
| Clostridium botulinum A3 str. Loch Maree | NC_010418 NC_010520 |
| Clostridium botulinum A str. ATCC 19397 | NC_009697 |
| Clostridium botulinum A str. ATCC 3502 | NC_009495 NC_009496 |
| Clostridium botulinum A str. Hall | NC_009698 |
| Clostridium botulinum B1 str. Okra | NC_010379 NC_010516 |
| Clostridium botulinum B str. Eklund 17B | NC_010674 NC_010680 |
| Clostridium botulinum Ba4 str. 657 | NC_012654 NC_012657 NC_012658 |
| Clostridium botulinum E3 str. Alaska E43 | NC_010723 |
| Clostridium botulinum F str. Langeland | NC_009699 NC_009700 |
| Clostridium cellulolyticum H10 | NC_011898 |
| Clostridium cellulovorans 743B | NC_014393 |
| Clostridium difficile 630 | NC_008226 NC_009089 |
| Clostridium difficile CD196 | NC_013315 |
| Clostridium difficile R20291 | NC_013316 |
| Clostridium kluyveri DSM 555 | NC_009466 NC_009706 |
| Clostridium kluyveri NBRC 12016 | NC_011836 NC_011837 |
| Clostridium ljungdahlii DSM 13528 | NC_014328 |
| Clostridium novyi NT | NC_008593 |
| Clostridium perfringens ATCC 13124 | NC_008261 |
| Clostridium perfringens SM101 | NC_008262 NC_008263 NC_008264 |
| Clostridium perfringens str. 13 | NC_003042 NC_003366 |
| Clostridium phytofermentans ISDg | NC_010001 |
| Clostridium saccharolyticum WM1 | NC_014376 |
| Clostridium sticklandii DSM 519 | NC_014614 |
| Clostridium tetani E88 | NC_004557 NC_004565 |
| Clostridium thermocellum ATCC 27405 | NC_009012 |
| Colwellia psychrerythraea 34H | NC_003910 |
| Comamonas sp. CNB-1 | NC_010935 NC_013446 |
| Conexibacter woesei DSM 14684 | NC_013739 |
| Coprothermobacter proteolyticus DSM 5265 | NC_011295 |
| Coraliomargarita akajimensis DSM 45221 | NC_014008 |
| Corynebacterium aurimucosum ATCC 700975 | NC_010813 NC_012590 |
| Corynebacterium diphtheriae NCTC 13129 | NC_002935 |
| Corynebacterium efficiens YS-314 | NC_004319 NC_004320 NC_004369 |
| Corynebacterium glutamicum ATCC 13032 | NC_003450 NC_006958 |
| Corynebacterium glutamicum R | NC_009342 NC_009343 |
| Corynebacterium jeikeium K411 | NC_003080 NC_007164 |
| Corynebacterium kroppenstedtii DSM 44385 | NC_012704 |
| Corynebacterium pseudotuberculosis FRC41 | NC_014329 |
| Corynebacterium urealyticum DSM 7109 | NC_010545 |
| Coxiella burnetii CbuGUNDERSCOREQ212 | NC_011527 |
| Coxiella burnetii CbuKUNDERSCOREQ154 | NC_011526 NC_011528 |
| Coxiella burnetii Dugway 5J108-111 | NC_009726 NC_009727 |
| Coxiella burnetii RSA 331 | NC_010115 NC_010117 |
| Coxiella burnetii RSA 493 | NC_002971 NC_004704 |
| Croceibacter atlanticus HTCC2559 | NC_014230 |
| Cronobacter sakazakii ATCC BAA-894 | NC_009778 NC_009779 NC_009780 |
| Cronobacter turicensis | NC_013282 NC_013283 NC_013284 NC_013285 |
| Cryptobacterium curtum DSM 15641 | NC_013170 |
| Cupriavidus metallidurans CH34 | NC_007971 NC_007972 NC_007973 NC_007974 |
| Cupriavidus taiwanensis | NC_010528 NC_010529 NC_010530 |
| Cyanothece sp. ATCC 51142 | NC_010539 NC_010541 NC_010542 NC_010543 NC_010546 NC_010547 |
| Cyanothece sp. PCC 7424 | NC_011729 NC_011730 NC_011732 NC_011733 NC_011734 NC_011737 NC_011738 |
| Cyanothece sp. PCC 7425 | NC_011880 NC_011882 NC_011884 NC_011885 |
| Cyanothece sp. PCC 7822 | NC_014501 NC_014502 NC_014503 NC_014504 NC_014533 NC_014534 NC_014535 |
| Cyanothece sp. PCC 8801 | NC_011721 NC_011723 NC_011726 NC_011727 |
| Cyanothece sp. PCC 8802 | NC_013160 NC_013161 NC_013163 NC_013167 NC_013168 |
| Cytophaga hutchinsonii ATCC 33406 | NC_008255 |
| Dechloromonas aromatica RCB | NC_007298 |
| Deferribacter desulfuricans SSM1 | NC_013939 NC_013940 |
| Dehalococcoides ethenogenes 195 | NC_002936 |
| Dehalococcoides sp. BAV1 | NC_009455 |
| Dehalococcoides sp. CBDB1 | NC_007356 |
| Dehalococcoides sp. GT | NC_013890 |
| Dehalococcoides sp. VS | NC_013552 |
| Dehalogenimonas lykanthroporepellens BL-DC-9 | NC_014314 |
| Deinococcus deserti VCD115 | NC_012526 NC_012527 NC_012528 NC_012529 |
| Deinococcus geothermalis DSM 11300 | NC_008010 NC_008025 NC_009939 |
| Deinococcus radiodurans R1 | NC_000958 NC_000959 NC_001263 NC_001264 |
| Delftia acidovorans SPH-1 | NC_010002 |
| Denitrovibrio acetiphilus DSM 12809 | NC_013943 |
| Desulfarculus baarsii DSM 2075 | NC_014365 |
| Desulfatibacillum alkenivorans AK-01 | NC_011768 |
| Desulfitobacterium hafniense DCB-2 | NC_011830 |
| Desulfitobacterium hafniense Y51 | NC_007907 |
| Desulfobacterium autotrophicum HRM2 | NC_012108 NC_012109 |
| Desulfococcus oleovorans Hxd3 | NC_009943 |
| Desulfohalobium retbaense DSM 5692 | NC_013223 NC_013224 |
| Desulfomicrobium baculatum DSM 4028 | NC_013173 |
| Desulfotalea psychrophila LSv54 | NC_006138 NC_006139 NC_006140 |
| Desulfotomaculum acetoxidans DSM 771 | NC_013216 |
| Desulfotomaculum reducens MI-1 | NC_009253 |
| Desulfovibrio desulfuricans subsp. desulfuricans str. ATCC 27774 | NC_011883 |
| Desulfovibrio desulfuricans subsp. desulfuricans str. G20 | NC_007519 |
| Desulfovibrio magneticus RS-1 | NC_012795 NC_012796 NC_012797 |
| Desulfovibrio salexigens DSM 2638 | NC_012881 |
| Desulfovibrio vulgaris DP4 | NC_008741 NC_008751 |
| Desulfovibrio vulgaris str. Hildenborough | NC_002937 NC_005863 |
| Desulfovibrio vulgaris str. SINGLEQUOTEMiyazaki FSINGLEQUOTE | NC_011769 |
| Desulfurivibrio alkaliphilus AHT2 | NC_014216 |
| Desulfurococcus kamchatkensis 1221n | NC_011766 |
| Dichelobacter nodosus VCS1703A | NC_009446 |
| Dickeya dadantii 3937 | NC_014500 |
| Dickeya dadantii Ech586 | NC_013592 |
| Dickeya dadantii Ech703 | NC_012880 |
| Dickeya zeae Ech1591 | NC_012912 |
| Dictyoglomus thermophilum H-6-12 | NC_011297 |
| Dictyoglomus turgidum DSM 6724 | NC_011661 |
| Dinoroseobacter shibae DFL 12 | NC_009952 NC_009955 NC_009956 NC_009957 NC_009958 NC_009959 |
| Dyadobacter fermentans DSM 18053 | NC_013037 |
| Edwardsiella ictaluri 93-146 | NC_012779 |
| Edwardsiella tarda EIB202 | NC_013508 NC_013509 |
| Eggerthella lenta DSM 2243 | NC_013204 |
| Ehrlichia canis str. Jake | NC_007354 |
| Ehrlichia chaffeensis str. Arkansas | NC_007799 |
| Ehrlichia ruminantium str. Gardel | NC_006831 |
| Ehrlichia ruminantium str. Welgevonden | NC_005295 NC_006832 |
| Elusimicrobium minutum Pei191 | NC_010644 |
| Enterobacter cloacae SCF1 | NC_014618 |
| Enterobacter cloacae subsp. cloacae ATCC 13047 | NC_014107 NC_014108 NC_014121 |
| Enterobacter sp. 638 | NC_009425 NC_009436 |
| Enterococcus faecalis V583 | NC_004668 NC_004669 NC_004670 NC_004671 |
| Erwinia amylovora ATCC 49946 | NC_013971 NC_013972 NC_013973 |
| Erwinia amylovora CFBP1430 | NC_013957 NC_013961 |
| Erwinia billingiae Eb661 | NC_014304 NC_014305 NC_014306 |
| Erwinia pyrifoliae Ep1SLASH96 | NC_012214 NC_013263 NC_013264 NC_013265 NC_013954 |
| Erwinia tasmaniensis Et1SLASH99 | NC_010693 NC_010694 NC_010695 NC_010696 NC_010697 NC_010699 |
| Erythrobacter litoralis HTCC2594 | NC_007722 |
| Escherichia coli 536 | NC_008253 |
| Escherichia coli 55989 | NC_011748 |
| Escherichia coli APEC O1 | NC_008563 NC_009837 NC_009838 |
| Escherichia coli ATCC 8739 | NC_010468 |
| Escherichia coli BW2952 | NC_012759 |
| Escherichia coli B str. REL606 | NC_012967 |
| Escherichia coli CFT073 | NC_004431 |
| Escherichia coli E24377A | NC_009786 NC_009787 NC_009788 NC_009789 NC_009790 NC_009791 NC_009801 |
| Escherichia coli ED1a | NC_011745 |
| Escherichia coli HS | NC_009800 |
| Escherichia coli IAI1 | NC_011741 |
| Escherichia coli IAI39 | NC_011750 |
| Escherichia coli O103COLONCOLONH2 str. 12009 | NC_013353 NC_013354 |
| Escherichia coli O111COLONCOLONH- str. 11128 | NC_013364 NC_013365 NC_013366 NC_013367 NC_013368 NC_013370 |
| Escherichia coli O127COLONCOLONH6 str. E2348SLASH69 | NC_011601 NC_011602 NC_011603 |
| Escherichia coli O157COLONCOLONH7 str. EC4115 | NC_011350 NC_011351 NC_011353 |
| Escherichia coli O157COLONCOLONH7 str. EDL933 | NC_002655 NC_007414 |
| Escherichia coli O157COLONCOLONH7 str. Sakai | NC_002127 NC_002128 NC_002695 |
| Escherichia coli O157COLONCOLONH7 str. TW14359 | NC_013008 NC_013010 |
| Escherichia coli O26COLONCOLONH11 str. 11368 | NC_013361 NC_013362 NC_013363 NC_013369 NC_014543 |
| Escherichia coli O55COLONCOLONH7 str. CB9615 | NC_013941 NC_013942 |
| Escherichia coli S88 | NC_011742 NC_011747 |
| Escherichia coli SE11 | NC_011407 NC_011408 NC_011411 NC_011413 NC_011415 NC_011416 NC_011419 |
| Escherichia coli SINGLEQUOTEBL21-GoldLPARENDE3RPARENpLysS AGSINGLEQUOTE | NC_012947 |
| Escherichia coli SMS-3-5 | NC_010485 NC_010486 NC_010487 NC_010488 NC_010498 |
| Escherichia coli UMN026 | NC_011739 NC_011749 NC_011751 |
| Escherichia coli UTI89 | NC_007941 NC_007946 |
| Escherichia coli str. K-12 substr. DH10B | NC_010473 |
| Escherichia coli str. K-12 substr. MG1655 | NC_000913 |
| Escherichia fergusonii ATCC 35469 | NC_011740 NC_011743 |
| Eubacterium eligens ATCC 27750 | NC_012778 NC_012780 NC_012782 |
| Eubacterium limosum KIST612 | NC_014624 |
| Eubacterium rectale ATCC 33656 | NC_012781 |
| Exiguobacterium sibiricum 255-15 | NC_010549 NC_010550 NC_010556 |
| Exiguobacterium sp. AT1b | NC_012673 |
| Ferrimonas balearica DSM 9799 | NC_014541 |
| Ferroglobus placidus DSM 10642 | NC_013849 |
| Fervidobacterium nodosum Rt17-B1 | NC_009718 |
| Fibrobacter succinogenes subsp. succinogenes S85 | NC_013410 |
| Finegoldia magna ATCC 29328 | NC_010371 NC_010376 |
| Flavobacteriaceae bacterium 3519-10 | NC_013062 |
| Flavobacterium johnsoniae UW101 | NC_009441 |
| Flavobacterium psychrophilum JIP02SLASH86 | NC_009613 |
| Francisella novicida U112 | NC_008601 |
| Francisella philomiragia subsp. philomiragia ATCC 25017 | NC_010331 NC_010336 |
| Francisella tularensis subsp. holarctica FTNF002-00 | NC_009749 |
| Francisella tularensis subsp. holarctica LVS | NC_007880 |
| Francisella tularensis subsp. holarctica OSU18 | NC_008369 |
| Francisella tularensis subsp. mediasiatica FSC147 | NC_010677 |
| Francisella tularensis subsp. tularensis FSC198 | NC_008245 |
| Francisella tularensis subsp. tularensis SCHU S4 | NC_006570 |
| Francisella tularensis subsp. tularensis WY96-3418 | NC_009257 |
| Frankia alni ACN14a | NC_008278 |
| Frankia sp. CcI3 | NC_007777 |
| Frankia sp. EAN1pec | NC_009921 |
| Frankia sp. EuI1c | NC_014666 |
| Fusobacterium nucleatum subsp. nucleatum ATCC 25586 | NC_003454 |
| Gallionella capsiferriformans ES-2 | NC_014394 |
| Gardnerella vaginalis 409-05 | NC_013721 |
| Gardnerella vaginalis ATCC 14019 | NC_014644 |
| Gemmatimonas aurantiaca T-27 | NC_012489 |
| Geobacillus kaustophilus HTA426 | NC_006509 NC_006510 |
| Geobacillus sp. C56-T3 | NC_014206 |
| Geobacillus sp. WCH70 | NC_012790 NC_012793 NC_012794 |
| Geobacillus sp. Y4.1MC1 | NC_014650 NC_014651 |
| Geobacillus sp. Y412MC61 | NC_013411 NC_013412 |
| Geobacillus thermodenitrificans NG80-2 | NC_009328 NC_009329 |
| Geobacter bemidjiensis Bem | NC_011146 |
| Geobacter lovleyi SZ | NC_010814 NC_010815 |
| Geobacter metallireducens GS-15 | NC_007515 NC_007517 |
| Geobacter sp. FRC-32 | NC_011979 |
| Geobacter sp. M21 | NC_012918 |
| Geobacter sulfurreducens PCA | NC_002939 |
| Geobacter uraniireducens Rf4 | NC_009483 |
| Geodermatophilus obscurus DSM 43160 | NC_013757 |
| Gloeobacter violaceus PCC 7421 | NC_005125 |
| Gluconacetobacter diazotrophicus PAl 5 | NC_010123 NC_010124 NC_010125 NC_011365 NC_011367 |
| Gluconobacter oxydans 621H | NC_006672 NC_006673 NC_006674 NC_006675 NC_006676 NC_006677 |
| Gordonia bronchialis DSM 43247 | NC_013441 NC_013442 |
| Gramella forsetii KT0803 | NC_008571 |
| Granulibacter bethesdensis CGDNIH1 | NC_008343 |
| Haemophilus ducreyi 35000HP | NC_002940 |
| Haemophilus influenzae 86-028NP | NC_007146 |
| Haemophilus influenzae PittEE | NC_009566 |
| Haemophilus influenzae PittGG | NC_009567 |
| Haemophilus influenzae Rd KW20 | NC_000907 |
| Haemophilus parasuis SH0165 | NC_011852 |
| Haemophilus somnus 129PT | NC_006298 NC_008309 |
| Haemophilus somnus 2336 | NC_010519 |
| Hahella chejuensis KCTC 2396 | NC_007645 |
| Halalkalicoccus jeotgali B3 | NC_014297 NC_014298 NC_014299 NC_014300 NC_014301 NC_014302 NC_014303 |
| Halanaerobium sp. SINGLEQUOTEsapolanicusSINGLEQUOTE | NC_014654 |
| Haliangium ochraceum DSM 14365 | NC_013440 |
| Haloarcula marismortui ATCC 43049 | NC_006389 NC_006390 NC_006391 NC_006392 NC_006393 NC_006394 NC_006395 NC_006396 NC_006397 |
| Halobacterium salinarum R1 | NC_010364 NC_010366 NC_010367 NC_010368 NC_010369 |
| Halobacterium sp. NRC-1 | NC_001869 NC_002607 NC_002608 |
| Haloferax volcanii DS2 | NC_013964 NC_013965 NC_013966 NC_013967 NC_013968 |
| Halogeometricum borinquense DSM 11551 | NC_014729 NC_014731 NC_014732 NC_014735 NC_014736 NC_014737 |
| Halomicrobium mukohataei DSM 12286 | NC_013201 NC_013202 |
| Halomonas elongata DSM 2581 | NC_014532 |
| Haloquadratum walsbyi DSM 16790 | NC_008212 NC_008213 |
| Halorhabdus utahensis DSM 12940 | NC_013158 |
| Halorhodospira halophila SL1 | NC_008789 |
| Halorubrum lacusprofundi ATCC 49239 | NC_012028 NC_012029 NC_012030 |
| Haloterrigena turkmenica DSM 5511 | NC_013743 NC_013744 NC_013745 NC_013746 NC_013747 NC_013748 NC_013749 |
| Halothermothrix orenii H 168 | NC_011899 |
| Halothiobacillus neapolitanus c2 | NC_013422 |
| Helicobacter acinonychis str. Sheeba | NC_008229 NC_008230 |
| Helicobacter felis ATCC 49179 | NC_014810 |
| Helicobacter hepaticus ATCC 51449 | NC_004917 |
| Helicobacter mustelae 12198 | NC_013949 |
| Helicobacter pylori 26695 | NC_000915 |
| Helicobacter pylori B38 | NC_012973 |
| Helicobacter pylori B8 | NC_014256 NC_014257 |
| Helicobacter pylori G27 | NC_011333 NC_011334 |
| Helicobacter pylori HPAG1 | NC_008086 NC_008087 |
| Helicobacter pylori J99 | NC_000921 |
| Helicobacter pylori P12 | NC_011498 NC_011499 |
| Helicobacter pylori PeCan4 | NC_014555 NC_014556 |
| Helicobacter pylori SJM180 | NC_014560 |
| Helicobacter pylori Shi470 | NC_010698 |
| Heliobacterium modesticaldum Ice1 | NC_010337 |
| Herbaspirillum seropedicae SmR1 | NC_014323 |
| Herminiimonas arsenicoxydans | NC_009138 |
| Herpetosiphon aurantiacus ATCC 23779 | NC_009972 NC_009973 NC_009974 |
| Hirschia baltica ATCC 49814 | NC_012982 NC_012983 |
| Hydrogenobacter thermophilus TK-6 | NC_013799 |
| Hydrogenobaculum sp. Y04AAS1 | NC_011126 |
| Hyperthermus butylicus DSM 5456 | NC_008818 |
| Hyphomicrobium denitrificans ATCC 51888 | NC_014313 |
| Hyphomonas neptunium ATCC 15444 | NC_008358 |
| Idiomarina loihiensis L2TR | NC_006512 |
| Ignicoccus hospitalis KIN4SLASHI | NC_009776 |
| Ignisphaera aggregans DSM 17230 | NC_014471 |
| Ilyobacter polytropus DSM 2926 | NC_014632 NC_014633 NC_014634 |
| Jannaschia sp. CCS1 | NC_007801 NC_007802 |
| Janthinobacterium sp. Marseille | NC_009659 |
| Jonesia denitrificans DSM 20603 | NC_013174 |
| Kangiella koreensis DSM 16069 | NC_013166 |
| Ketogulonicigenium vulgare Y25 | NC_014621 NC_014625 NC_014626 |
| Kineococcus radiotolerans SRS30216 | NC_009660 NC_009664 NC_009806 |
| Klebsiella pneumoniae 342 | NC_011281 NC_011282 NC_011283 |
| Klebsiella pneumoniae NTUH-K2044 | NC_006625 NC_012731 |
| Klebsiella pneumoniae subsp. pneumoniae MGH 78578 | NC_009648 NC_009649 NC_009650 NC_009651 NC_009652 NC_009653 |
| Klebsiella variicola At-22 | NC_013850 |
| Kocuria rhizophila DC2201 | NC_010617 |
| Kosmotoga olearia TBF 19.5.1 | NC_012785 |
| Kribbella flavida DSM 17836 | NC_013729 |
| Kytococcus sedentarius DSM 20547 | NC_013169 |
| Lactobacillus acidophilus NCFM | NC_006814 |
| Lactobacillus amylovorus GRL 1112 | NC_014724 |
| Lactobacillus brevis ATCC 367 | NC_008497 NC_008498 NC_008499 |
| Lactobacillus casei ATCC 334 | NC_008502 NC_008526 |
| Lactobacillus casei BL23 | NC_010999 |
| Lactobacillus casei str. Zhang | NC_011352 NC_014334 |
| Lactobacillus crispatus ST1 | NC_014106 |
| Lactobacillus delbrueckii subsp. bulgaricus ATCC 11842 | NC_008054 |
| Lactobacillus delbrueckii subsp. bulgaricus ATCC BAA-365 | NC_008529 |
| Lactobacillus delbrueckii subsp. bulgaricus ND02 | NC_014727 NC_014728 |
| Lactobacillus fermentum IFO 3956 | NC_010610 |
| Lactobacillus gasseri ATCC 33323 | NC_008530 |
| Lactobacillus helveticus DPC 4571 | NC_010080 |
| Lactobacillus johnsonii FI9785 | NC_012552 NC_013504 NC_013505 |
| Lactobacillus johnsonii NCC 533 | NC_005362 |
| Lactobacillus plantarum JDM1 | NC_012984 |
| Lactobacillus plantarum WCFS1 | NC_004567 NC_006375 NC_006376 NC_006377 |
| Lactobacillus plantarum subsp. plantarum ST-III | NC_014554 NC_014558 |
| Lactobacillus reuteri DSM 20016 | NC_009513 |
| Lactobacillus reuteri JCM 1112 | NC_010609 |
| Lactobacillus rhamnosus GG | NC_013198 |
| Lactobacillus rhamnosus Lc 705 | NC_013199 NC_013200 |
| Lactobacillus sakei subsp. sakei 23K | NC_007576 |
| Lactobacillus salivarius UCC118 | NC_006529 NC_006530 NC_007929 NC_007930 |
| Lactococcus lactis subsp. cremoris MG1363 | NC_009004 |
| Lactococcus lactis subsp. cremoris SK11 | NC_008503 NC_008504 NC_008505 NC_008506 NC_008507 NC_008527 |
| Lactococcus lactis subsp. lactis Il1403 | NC_002662 |
| Lactococcus lactis subsp. lactis KF147 | NC_013656 NC_013657 |
| Laribacter hongkongensis HLHK9 | NC_012559 |
| Lawsonia intracellularis PHESLASHMN1-00 | NC_008011 NC_008012 NC_008013 NC_008014 |
| Leadbetterella byssophila DSM 17132 | NC_014655 |
| Legionella longbeachae NSW150 | NC_013861 NC_014544 |
| Legionella pneumophila 2300SLASH99 Alcoy | NC_014125 |
| Legionella pneumophila str. Corby | NC_009494 |
| Legionella pneumophila str. Lens | NC_006366 NC_006369 |
| Legionella pneumophila str. Paris | NC_006365 NC_006368 |
| Legionella pneumophila subsp. pneumophila str. Philadelphia 1 | NC_002942 |
| Leifsonia xyli subsp. xyli str. CTCB07 | NC_006087 |
| Leptospira biflexa serovar Patoc strain SINGLEQUOTEPatoc 1 LPARENAmesRPARENSINGLEQUOTE | NC_010842 NC_010845 NC_010846 |
| Leptospira biflexa serovar Patoc strain SINGLEQUOTEPatoc 1 LPARENParisRPARENSINGLEQUOTE | NC_010602 NC_010843 NC_010844 |
| Leptospira borgpetersenii serovar Hardjo-bovis JB197 | NC_008510 NC_008511 |
| Leptospira borgpetersenii serovar Hardjo-bovis L550 | NC_008508 NC_008509 |
| Leptospira interrogans serovar Copenhageni str. Fiocruz L1-130 | NC_005823 NC_005824 |
| Leptospira interrogans serovar Lai str. 56601 | NC_004342 NC_004343 |
| Leptothrix cholodnii SP-6 | NC_010524 |
| Leptotrichia buccalis C-1013-b | NC_013192 |
| Leuconostoc citreum KM20 | NC_010466 NC_010467 NC_010469 NC_010470 NC_010471 |
| Leuconostoc gasicomitatum LMG 18811 | NC_014319 |
| Leuconostoc kimchii IMSNU 11154 | NC_014131 NC_014132 NC_014133 NC_014134 NC_014135 NC_014136 |
| Leuconostoc mesenteroides subsp. mesenteroides ATCC 8293 | NC_008496 NC_008531 |
| Listeria innocua Clip11262 | NC_003212 NC_003383 |
| Listeria monocytogenes 08-5578 | NC_013766 NC_013767 |
| Listeria monocytogenes 08-5923 | NC_013768 |
| Listeria monocytogenes EGD-e | NC_003210 |
| Listeria monocytogenes HCC23 | NC_011660 |
| Listeria monocytogenes serotype 4b str. CLIP 80459 | NC_012488 |
| Listeria monocytogenes serotype 4b str. F2365 | NC_002973 |
| Listeria seeligeri serovar 1SLASH2b str. SLCC3954 | NC_013891 |
| Listeria welshimeri serovar 6b str. SLCC5334 | NC_008555 |
| Lysinibacillus sphaericus C3-41 | NC_010381 NC_010382 |
| Macrococcus caseolyticus JCSC5402 | NC_011995 NC_011996 NC_011997 NC_011998 NC_011999 NC_012000 NC_012001 NC_012002 NC_012003 |
| Magnetococcus sp. MC-1 | NC_008576 |
| Magnetospirillum magneticum AMB-1 | NC_007626 |
| Mannheimia succiniciproducens MBEL55E | NC_006300 |
| Maribacter sp. HTCC2170 | NC_014472 |
| Maricaulis maris MCS10 | NC_008347 |
| Marinobacter aquaeolei VT8 | NC_008738 NC_008739 NC_008740 |
| Marinomonas sp. MWYL1 | NC_009654 |
| Marivirga tractuosa DSM 4126 | NC_014750 NC_014759 |
| Meiothermus ruber DSM 1279 | NC_013946 |
| Meiothermus silvanus DSM 9946 | NC_014212 NC_014213 NC_014214 |
| Mesoplasma florum L1 | NC_006055 |
| Mesorhizobium loti MAFF303099 | NC_002678 NC_002679 NC_002682 |
| Metallosphaera sedula DSM 5348 | NC_009440 |
| Methanobrevibacter ruminantium M1 | NC_013790 |
| Methanobrevibacter smithii ATCC 35061 | NC_009515 |
| Methanocaldococcus fervens AG86 | NC_013156 NC_013157 |
| Methanocaldococcus infernus ME | NC_014122 |
| Methanocaldococcus jannaschii DSM 2661 | NC_000909 NC_001732 NC_001733 |
| Methanocaldococcus sp. FS406-22 | NC_013887 NC_013888 |
| Methanocaldococcus vulcanius M7 | NC_013407 NC_013408 NC_013409 |
| Methanocella paludicola SANAE | NC_013665 |
| Methanococcoides burtonii DSM 6242 | NC_007955 |
| Methanococcus aeolicus Nankai-3 | NC_009635 |
| Methanococcus maripaludis C5 | NC_009135 NC_009136 |
| Methanococcus maripaludis C6 | NC_009975 |
| Methanococcus maripaludis C7 | NC_009637 |
| Methanococcus maripaludis S2 | NC_005791 |
| Methanococcus vannielii SB | NC_009634 |
| Methanococcus voltae A3 | NC_014222 |
| Methanocorpusculum labreanum Z | NC_008942 |
| Methanoculleus marisnigri JR1 | NC_009051 |
| Methanohalobium evestigatum Z-7303 | NC_014253 NC_014254 |
| Methanohalophilus mahii DSM 5219 | NC_014002 |
| Methanoplanus petrolearius DSM 11571 | NC_014507 |
| Methanopyrus kandleri AV19 | NC_003551 |
| Methanosaeta thermophila PT LPARENMethanothrix thermophila PTRPAREN | NC_008553 |
| Methanosarcina acetivorans C2A | NC_003552 |
| Methanosarcina barkeri str. Fusaro | NC_007349 NC_007355 |
| Methanosarcina mazei Go1 | NC_003901 |
| Methanosphaera stadtmanae DSM 3091 | NC_007681 |
| Methanosphaerula palustris E1-9c | NC_011832 |
| Methanospirillum hungatei JF-1 | NC_007796 |
| Methanothermobacter marburgensis str. Marburg | NC_014408 NC_014409 |
| Methanothermobacter thermautotrophicus str. Delta H LPARENMethanobacterium thermoautotrophicum str. deltaHRPAREN | NC_000916 |
| Methanothermus fervidus DSM 2088 | NC_014658 |
| Methylacidiphilum infernorum V4 | NC_010794 |
| Methylibium petroleiphilum PM1 | NC_008825 NC_008826 |
| Methylobacillus flagellatus KT | NC_007947 |
| Methylobacterium chloromethanicum CM4 | NC_011757 NC_011758 NC_011760 |
| Methylobacterium extorquens AM1 | NC_012807 NC_012808 NC_012809 NC_012810 NC_012811 |
| Methylobacterium extorquens DM4 LPARENMethylobacterium dichloromethanicumRPAREN | NC_012987 NC_012988 NC_012989 |
| Methylobacterium extorquens PA1 | NC_010172 |
| Methylobacterium nodulans ORS 2060 | NC_011887 NC_011888 NC_011889 NC_011890 NC_011892 NC_011893 NC_011894 NC_011895 |
| Methylobacterium populi BJ001 | NC_010721 NC_010725 NC_010727 |
| Methylobacterium radiotolerans JCM 2831 | NC_010502 NC_010504 NC_010505 NC_010507 NC_010509 NC_010510 NC_010514 NC_010517 NC_010518 |
| Methylobacterium sp. 4-46 | NC_010373 NC_010374 NC_010511 |
| Methylocella silvestris BL2 | NC_011666 |
| Methylococcus capsulatus str. Bath | NC_002977 |
| Methylotenera mobilis JLW8 | NC_012968 |
| Methylotenera sp. 301 | NC_014207 |
| Methylovorus sp. MP688 | NC_014733 |
| Methylovorus sp. SIP3-4 | NC_012969 NC_012970 NC_012972 |
| Micrococcus luteus NCTC 2665 | NC_012803 |
| Microcystis aeruginosa NIES-843 | NC_010296 |
| Micromonospora aurantiaca ATCC 27029 | NC_014391 |
| Micromonospora sp. L5 | NC_014815 |
| Mobiluncus curtisii ATCC 43063 | NC_014246 |
| Moorella thermoacetica ATCC 39073 | NC_007644 |
| Moraxella catarrhalis RH4 | NC_014147 |
| Mycobacterium abscessus ATCC 19977 | NC_010394 NC_010397 |
| Mycobacterium avium 104 | NC_008595 |
| Mycobacterium avium subsp. paratuberculosis K-10 | NC_002944 |
| Mycobacterium bovis AF2122SLASH97 | NC_002945 |
| Mycobacterium bovis BCG str. Pasteur 1173P2 | NC_008769 |
| Mycobacterium bovis BCG str. Tokyo 172 | NC_012207 |
| Mycobacterium gilvum PYR-GCK | NC_009338 NC_009339 NC_009340 NC_009341 |
| Mycobacterium leprae Br4923 | NC_011896 |
| Mycobacterium leprae TN | NC_002677 |
| Mycobacterium marinum M | NC_010604 NC_010612 |
| Mycobacterium smegmatis str. MC2 155 | NC_008596 |
| Mycobacterium sp. JLS | NC_009077 |
| Mycobacterium sp. KMS | NC_008703 NC_008704 NC_008705 |
| Mycobacterium sp. MCS | NC_008146 NC_008147 |
| Mycobacterium sp. Spyr1 | NC_014811 NC_014812 NC_014814 |
| Mycobacterium tuberculosis CDC1551 | NC_002755 |
| Mycobacterium tuberculosis F11 | NC_009565 |
| Mycobacterium tuberculosis H37Ra | NC_009525 |
| Mycobacterium tuberculosis H37Rv | NC_000962 |
| Mycobacterium tuberculosis KZN 1435 | NC_012943 |
| Mycobacterium ulcerans Agy99 | NC_005916 NC_008611 |
| Mycobacterium vanbaalenii PYR-1 | NC_008726 |
| Mycoplasma agalactiae | NC_013948 |
| Mycoplasma agalactiae PG2 | NC_009497 |
| Mycoplasma arthritidis 158L3-1 | NC_011025 |
| Mycoplasma bovis PG45 | NC_014760 |
| Mycoplasma capricolum subsp. capricolum ATCC 27343 | NC_007633 |
| Mycoplasma conjunctivae HRCSLASH581 | NC_012806 |
| Mycoplasma crocodyli MP145 | NC_014014 |
| Mycoplasma fermentans JER | NC_014552 |
| Mycoplasma gallisepticum str. RLPARENlowRPAREN | NC_004829 |
| Mycoplasma genitalium G37 | NC_000908 |
| Mycoplasma hominis ATCC 23114 | NC_013511 |
| Mycoplasma hyopneumoniae 232 | NC_006360 |
| Mycoplasma hyopneumoniae 7448 | NC_007332 |
| Mycoplasma hyopneumoniae J | NC_007295 |
| Mycoplasma hyorhinis HUB-1 | NC_014448 |
| Mycoplasma leachii PG50 | NC_014751 |
| Mycoplasma mobile 163K | NC_006908 |
| Mycoplasma mycoides subsp. mycoides SC str. PG1 | NC_005364 |
| Mycoplasma penetrans HF-2 | NC_004432 |
| Mycoplasma pneumoniae M129 | NC_000912 |
| Mycoplasma pulmonis UAB CTIP | NC_002771 |
| Mycoplasma synoviae 53 | NC_007294 |
| Myxococcus xanthus DK 1622 | NC_008095 |
| Nakamurella multipartita DSM 44233 | NC_013235 |
| Nanoarchaeum equitans Kin4-M | NC_005213 |
| Natranaerobius thermophilus JWSLASHNM-WN-LF | NC_010715 NC_010718 NC_010724 |
| Natrialba magadii ATCC 43099 | NC_013922 NC_013923 NC_013924 NC_013925 |
| Natronomonas pharaonis DSM 2160 | NC_007426 NC_007427 NC_007428 |
| Nautilia profundicola AmH | NC_012115 |
| Neisseria gonorrhoeae FA 1090 | NC_002946 |
| Neisseria gonorrhoeae NCCP11945 | NC_011034 NC_011035 |
| Neisseria lactamica 020-06 | NC_014752 |
| Neisseria meningitidis 053442 | NC_010120 |
| Neisseria meningitidis FAM18 | NC_008767 |
| Neisseria meningitidis MC58 | NC_003112 |
| Neisseria meningitidis Z2491 | NC_003116 |
| Neisseria meningitidis alpha14 | NC_013016 |
| Neorickettsia risticii str. Illinois | NC_013009 |
| Neorickettsia sennetsu str. Miyayama | NC_007798 |
| Nitratiruptor sp. SB155-2 | NC_009662 |
| Nitrobacter hamburgensis X14 | NC_007959 NC_007960 NC_007961 NC_007964 |
| Nitrobacter winogradskyi Nb-255 | NC_007406 |
| Nitrosococcus halophilus Nc4 | NC_013958 NC_013960 |
| Nitrosococcus oceani ATCC 19707 | NC_007483 NC_007484 |
| Nitrosococcus watsoni C-113 | NC_014315 NC_014316 NC_014317 |
| Nitrosomonas europaea ATCC 19718 | NC_004757 |
| Nitrosomonas eutropha C91 | NC_008341 NC_008342 NC_008344 |
| Nitrosopumilus maritimus SCM1 | NC_010085 |
| Nitrosospira multiformis ATCC 25196 | NC_007614 NC_007615 NC_007616 NC_007617 |
| Nocardia farcinica IFM 10152 | NC_006361 NC_006362 NC_006363 |
| Nocardioides sp. JS614 | NC_008697 NC_008699 |
| Nocardiopsis dassonvillei subsp. dassonvillei DSM 43111 | NC_014210 NC_014211 |
| Nostoc punctiforme PCC 73102 LPARENNostoc punctiforme ATCC 29133RPAREN | NC_010628 NC_010629 NC_010630 NC_010631 NC_010632 NC_010633 |
| Nostoc sp. PCC 7120 | NC_003240 NC_003241 NC_003267 NC_003270 NC_003272 NC_003273 NC_003276 |
| Novosphingobium aromaticivorans DSM 12444 | NC_007794 NC_009426 NC_009427 |
| Oceanithermus profundus DSM 14977 | NC_014753 NC_014761 |
| Oceanobacillus iheyensis HTE831 | NC_004193 |
| Ochrobactrum anthropi ATCC 49188 | NC_009667 NC_009668 NC_009669 NC_009670 NC_009671 NC_009672 |
| Oenococcus oeni PSU-1 | NC_008528 |
| Oligotropha carboxidovorans OM5 | NC_011386 |
| Olsenella uli DSM 7084 | NC_014363 |
| Onion yellows phytoplasma OY-M | NC_005303 |
| Opitutus terrae PB90-1 | NC_010571 |
| Orientia tsutsugamushi str. Boryong | NC_009488 |
| Orientia tsutsugamushi str. Ikeda | NC_010793 |
| Paenibacillus polymyxa E681 | NC_014483 |
| Paenibacillus polymyxa SC2 | NC_014622 NC_014628 |
| Paenibacillus sp. JDR-2 | NC_012914 |
| Paenibacillus sp. Y412MC10 | NC_013406 |
| Paludibacter propionicigenes WB4 | NC_014734 |
| Pantoea ananatis LMG 20103 | NC_013956 |
| Pantoea vagans C9-1 | NC_014258 NC_014561 NC_014562 NC_014563 |
| Parabacteroides distasonis ATCC 8503 | NC_009615 |
| Paracoccus denitrificans PD1222 | NC_008686 NC_008687 NC_008688 |
| Parvibaculum lavamentivorans DS-1 | NC_009719 |
| Parvularcula bermudensis HTCC2503 | NC_014414 |
| Pasteurella multocida subsp. multocida str. Pm70 | NC_002663 |
| Pectobacterium atrosepticum SCRI1043 | NC_004547 |
| Pectobacterium carotovorum subsp. carotovorum PC1 | NC_012917 |
| Pectobacterium wasabiae WPP163 | NC_013421 |
| Pediococcus pentosaceus ATCC 25745 | NC_008525 |
| Pedobacter heparinus DSM 2366 | NC_013061 |
| Pelobacter carbinolicus DSM 2380 | NC_007498 |
| Pelobacter propionicus DSM 2379 | NC_008607 NC_008608 NC_008609 |
| Pelodictyon phaeoclathratiforme BU-1 | NC_011060 |
| Pelotomaculum thermopropionicum SI | NC_009454 |
| Persephonella marina EX-H1 | NC_012439 NC_012440 |
| Petrotoga mobilis SJ95 | NC_010003 |
| Phenylobacterium zucineum HLK1 | NC_011143 NC_011144 |
| Photobacterium profundum SS9 | NC_005871 NC_006370 NC_006371 |
| Photorhabdus asymbiotica | NC_012961 NC_012962 |
| Photorhabdus luminescens subsp. laumondii TTO1 | NC_005126 |
| Picrophilus torridus DSM 9790 | NC_005877 |
| Pirellula staleyi DSM 6068 | NC_013720 |
| Planctomyces limnophilus DSM 3776 | NC_014148 NC_014149 |
| Polaromonas naphthalenivorans CJ2 | NC_008757 NC_008758 NC_008759 NC_008760 NC_008761 NC_008762 NC_008763 NC_008764 NC_008781 |
| Polaromonas sp. JS666 | NC_007948 NC_007949 NC_007950 |
| Polynucleobacter necessarius subsp. asymbioticus QLW-P1DMWA-1 | NC_009379 |
| Polynucleobacter necessarius subsp. necessarius STIR1 | NC_010531 |
| Porphyromonas gingivalis ATCC 33277 | NC_010729 |
| Porphyromonas gingivalis W83 | NC_002950 |
| Prevotella melaninogenica ATCC 25845 | NC_014370 NC_014371 |
| Prevotella ruminicola 23 | NC_014033 |
| Prochlorococcus marinus str. AS9601 | NC_008816 |
| Prochlorococcus marinus str. MIT 9211 | NC_009976 |
| Prochlorococcus marinus str. MIT 9215 | NC_009840 |
| Prochlorococcus marinus str. MIT 9301 | NC_009091 |
| Prochlorococcus marinus str. MIT 9303 | NC_008820 |
| Prochlorococcus marinus str. MIT 9312 | NC_007577 |
| Prochlorococcus marinus str. MIT 9313 | NC_005071 |
| Prochlorococcus marinus str. MIT 9515 | NC_008817 |
| Prochlorococcus marinus str. NATL1A | NC_008819 |
| Prochlorococcus marinus str. NATL2A | NC_007335 |
| Prochlorococcus marinus subsp. marinus str. CCMP1375 LPARENProchlorococcus marinus SS120RPAREN | NC_005042 |
| Prochlorococcus marinus subsp. pastoris str. CCMP1986 LPARENProchlorococcus marinus MED4RPAREN | NC_005072 |
| Propionibacterium acnes KPA171202 | NC_006085 |
| Propionibacterium acnes SK137 | NC_014039 |
| Propionibacterium freudenreichii subsp. shermanii CIRM-BIA1 | NC_014215 |
| Prosthecochloris aestuarii DSM 271 | NC_011059 NC_011061 |
| Proteus mirabilis HI4320 | NC_010554 NC_010555 |
| Pseudoalteromonas atlantica T6c | NC_008228 |
| Pseudoalteromonas haloplanktis TAC125 | NC_007481 NC_007482 |
| Pseudoalteromonas sp. SM9913 | NC_014800 NC_014803 |
| Pseudomonas aeruginosa LESB58 | NC_011770 |
| Pseudomonas aeruginosa PA7 | NC_009656 |
| Pseudomonas aeruginosa PAO1 | NC_002516 |
| Pseudomonas aeruginosa UCBPP-PA14 | NC_008463 |
| Pseudomonas entomophila L48 | NC_008027 |
| Pseudomonas fluorescens Pf-5 | NC_004129 |
| Pseudomonas fluorescens Pf0-1 | NC_007492 |
| Pseudomonas fluorescens SBW25 | NC_009444 NC_012660 |
| Pseudomonas mendocina ymp | NC_009439 |
| Pseudomonas putida F1 | NC_009512 |
| Pseudomonas putida GB-1 | NC_010322 |
| Pseudomonas putida KT2440 | NC_002947 |
| Pseudomonas putida W619 | NC_010501 |
| Pseudomonas stutzeri A1501 | NC_009434 |
| Pseudomonas syringae pv. phaseolicola 1448A | NC_005773 NC_007274 NC_007275 |
| Pseudomonas syringae pv. syringae B728a | NC_007005 |
| Pseudomonas syringae pv. tomato str. DC3000 | NC_004578 NC_004632 NC_004633 |
| Psychrobacter arcticus 273-4 | NC_007204 |
| Psychrobacter cryohalolentis K5 | NC_007968 NC_007969 |
| Psychrobacter sp. PRwf-1 | NC_009516 NC_009517 NC_009524 |
| Psychromonas ingrahamii 37 | NC_008709 |
| Pyrobaculum aerophilum str. IM2 | NC_003364 |
| Pyrobaculum arsenaticum DSM 13514 | NC_009376 |
| Pyrobaculum calidifontis JCM 11548 | NC_009073 |
| Pyrobaculum islandicum DSM 4184 | NC_008701 |
| Pyrococcus abyssi GE5 | NC_000868 NC_001773 |
| Pyrococcus furiosus DSM 3638 | NC_003413 |
| Pyrococcus horikoshii OT3 | NC_000961 |
| Ralstonia eutropha H16 | NC_005241 NC_008313 NC_008314 |
| Ralstonia eutropha JMP134 | NC_007336 NC_007337 NC_007347 NC_007348 |
| Ralstonia pickettii 12D | NC_012849 NC_012851 NC_012855 NC_012856 NC_012857 |
| Ralstonia pickettii 12J | NC_010678 NC_010682 NC_010683 |
| Ralstonia solanacearum CFBP2957 | NC_014307 |
| Ralstonia solanacearum GMI1000 | NC_003295 NC_003296 |
| Ralstonia solanacearum PSI07 | NC_014310 NC_014311 |
| Renibacterium salmoninarum ATCC 33209 | NC_010168 |
| Rhizobium etli CFN 42 | NC_004041 NC_007761 NC_007762 NC_007763 NC_007764 NC_007765 NC_007766 |
| Rhizobium etli CIAT 652 | NC_010994 NC_010996 NC_010997 NC_010998 |
| Rhizobium leguminosarum bv. trifolii WSM1325 | NC_012848 NC_012850 NC_012852 NC_012853 NC_012854 NC_012858 |
| Rhizobium leguminosarum bv. trifolii WSM2304 | NC_011366 NC_011368 NC_011369 NC_011370 NC_011371 |
| Rhizobium leguminosarum bv. viciae 3841 | NC_008378 NC_008379 NC_008380 NC_008381 NC_008382 NC_008383 NC_008384 |
| Rhodobacter capsulatus SB 1003 | NC_014034 NC_014035 |
| Rhodobacter sphaeroides 2.4.1 | NC_007488 NC_007489 NC_007490 NC_007493 NC_007494 NC_009007 NC_009008 |
| Rhodobacter sphaeroides ATCC 17025 | NC_009428 NC_009429 NC_009430 NC_009431 NC_009432 NC_009433 |
| Rhodobacter sphaeroides ATCC 17029 | NC_009040 NC_009049 NC_009050 |
| Rhodobacter sphaeroides KD131 | NC_011958 NC_011960 NC_011962 NC_011963 |
| Rhodococcus equi 103S | NC_014659 |
| Rhodococcus erythropolis PR4 | NC_007486 NC_007487 NC_007491 NC_012490 |
| Rhodococcus jostii RHA1 | NC_008268 NC_008269 NC_008270 NC_008271 |
| Rhodococcus opacus B4 | NC_006969 NC_006970 NC_012520 NC_012521 NC_012522 NC_012523 |
| Rhodoferax ferrireducens T118 | NC_007901 NC_007908 |
| Rhodomicrobium vannielii ATCC 17100 | NC_014664 |
| Rhodopirellula baltica SH 1 | NC_005027 |
| Rhodopseudomonas palustris BisA53 | NC_008435 |
| Rhodopseudomonas palustris BisB18 | NC_007925 |
| Rhodopseudomonas palustris BisB5 | NC_007958 |
| Rhodopseudomonas palustris CGA009 | NC_005296 NC_005297 |
| Rhodopseudomonas palustris DX-1 | NC_014834 |
| Rhodopseudomonas palustris HaA2 | NC_007778 |
| Rhodopseudomonas palustris TIE-1 | NC_011004 |
| Rhodospirillum centenum SW LPARENRhodocista centenaria SWRPAREN | NC_011420 |
| Rhodospirillum rubrum ATCC 11170 | NC_007641 NC_007643 |
| Rhodothermus marinus DSM 4252 | NC_013501 NC_013502 |
| Rickettsia africae ESF-5 | NC_012633 NC_012634 |
| Rickettsia akari str. Hartford | NC_009881 |
| Rickettsia bellii OSU 85-389 | NC_009883 |
| Rickettsia bellii RML369-C | NC_007940 |
| Rickettsia canadensis str. McKiel | NC_009879 |
| Rickettsia conorii str. Malish 7 | NC_003103 |
| Rickettsia felis URRWXCal2 | NC_007109 NC_007110 NC_007111 |
| Rickettsia massiliae MTU5 | NC_009897 NC_009900 |
| Rickettsia peacockii str. Rustic | NC_012730 NC_012732 |
| Rickettsia prowazekii str. Madrid E | NC_000963 |
| Rickettsia rickettsii str. Iowa | NC_010263 |
| Rickettsia rickettsii str. SINGLEQUOTESheila SmithSINGLEQUOTE | NC_009882 |
| Rickettsia typhi str. Wilmington | NC_006142 |
| Riemerella anatipestifer DSM 15868 | NC_014738 |
| Robiginitalea biformata HTCC2501 | NC_013222 |
| Roseiflexus castenholzii DSM 13941 | NC_009767 |
| Roseiflexus sp. RS-1 | NC_009523 |
| Roseobacter denitrificans OCh 114 | NC_008209 NC_008386 NC_008387 NC_008388 NC_008389 |
| Rothia dentocariosa ATCC 17931 | NC_014643 |
| Rothia mucilaginosa DY-18 | NC_013715 |
| Rubrobacter xylanophilus DSM 9941 | NC_008148 |
| Ruegeria pomeroyi DSS-3 | NC_003911 NC_006569 |
| Ruegeria sp. TM1040 | NC_008042 NC_008043 NC_008044 |
| SINGLEQUOTENostoc azollaeSINGLEQUOTE 0708 | NC_014248 NC_014249 NC_014250 |
| Saccharomonospora viridis DSM 43017 | NC_013159 |
| Saccharophagus degradans 2-40 | NC_007912 |
| Saccharopolyspora erythraea NRRL 2338 | NC_009142 |
| Salinibacter ruber DSM 13855 | NC_007677 NC_007678 |
| Salinibacter ruber M8 | NC_014026 NC_014028 NC_014030 NC_014032 |
| Salinispora arenicola CNS-205 | NC_009953 |
| Salinispora tropica CNB-440 | NC_009380 |
| Salmonella enterica subsp. arizonae serovar 62COLONCOLONz4,z23COLONCOLON-- str. RSK2980 | NC_010067 |
| Salmonella enterica subsp. enterica serovar Agona str. SL483 | NC_011148 NC_011149 |
| Salmonella enterica subsp. enterica serovar Choleraesuis str. SC-B67 | NC_006855 NC_006856 NC_006905 |
| Salmonella enterica subsp. enterica serovar Dublin str. CTUNDERSCORE02021853 | NC_011204 NC_011205 |
| Salmonella enterica subsp. enterica serovar Enteritidis str. P125109 | NC_011294 |
| Salmonella enterica subsp. enterica serovar Gallinarum str. 287SLASH91 | NC_011274 |
| Salmonella enterica subsp. enterica serovar Heidelberg str. SL476 | NC_011081 NC_011082 NC_011083 |
| Salmonella enterica subsp. enterica serovar Newport str. SL254 | NC_009140 NC_011079 NC_011080 |
| Salmonella enterica subsp. enterica serovar Paratyphi A str. AKUUNDERSCORE12601 | NC_011147 |
| Salmonella enterica subsp. enterica serovar Paratyphi A str. ATCC 9150 | NC_006511 |
| Salmonella enterica subsp. enterica serovar Paratyphi B str. SPB7 | NC_010102 |
| Salmonella enterica subsp. enterica serovar Paratyphi C strain RKS4594 | NC_012124 NC_012125 |
| Salmonella enterica subsp. enterica serovar Schwarzengrund str. CVM19633 | NC_011092 NC_011093 NC_011094 |
| Salmonella enterica subsp. enterica serovar Typhi str. CT18 | NC_003198 NC_003384 NC_003385 |
| Salmonella enterica subsp. enterica serovar Typhi str. Ty2 | NC_004631 |
| Salmonella enterica subsp. enterica serovar Typhimurium str. LT2 | NC_003197 NC_003277 |
| Sanguibacter keddieii DSM 10542 | NC_013521 |
| Sebaldella termitidis ATCC 33386 | NC_013517 NC_013518 NC_013519 |
| Segniliparus rotundus DSM 44985 | NC_014168 |
| Serratia proteamaculans 568 | NC_009829 NC_009832 |
| Shewanella amazonensis SB2B | NC_008700 |
| Shewanella baltica OS155 | NC_009035 NC_009036 NC_009037 NC_009038 NC_009052 |
| Shewanella baltica OS185 | NC_009661 NC_009665 |
| Shewanella baltica OS195 | NC_009997 NC_009998 NC_009999 NC_010000 |
| Shewanella baltica OS223 | NC_011663 NC_011664 NC_011665 NC_011668 |
| Shewanella denitrificans OS217 | NC_007954 |
| Shewanella frigidimarina NCIMB 400 | NC_008345 |
| Shewanella halifaxensis HAW-EB4 | NC_010334 |
| Shewanella loihica PV-4 | NC_009092 |
| Shewanella oneidensis MR-1 | NC_004347 NC_004349 |
| Shewanella pealeana ATCC 700345 | NC_009901 |
| Shewanella piezotolerans WP3 | NC_011566 |
| Shewanella putrefaciens CN-32 | NC_009438 |
| Shewanella sediminis HAW-EB3 | NC_009831 |
| Shewanella sp. ANA-3 | NC_008573 NC_008577 |
| Shewanella sp. MR-4 | NC_008321 |
| Shewanella sp. MR-7 | NC_008320 NC_008322 |
| Shewanella sp. W3-18-1 | NC_008750 |
| Shewanella violacea DSS12 | NC_014012 |
| Shewanella woodyi ATCC 51908 | NC_010506 |
| Shigella boydii CDC 3083-94 | NC_010656 NC_010657 NC_010658 NC_010659 NC_010660 NC_010672 |
| Shigella boydii Sb227 | NC_007608 NC_007613 |
| Shigella dysenteriae Sd197 | NC_007606 NC_007607 NC_009344 |
| Shigella flexneri 2a str. 2457T | NC_004741 |
| Shigella flexneri 2a str. 301 | NC_004337 NC_004851 |
| Shigella flexneri 5 str. 8401 | NC_008258 |
| Shigella sonnei Ss046 | NC_007384 NC_007385 NC_009345 NC_009346 NC_009347 |
| Sideroxydans lithotrophicus ES-1 | NC_013959 |
| Sinorhizobium fredii NGR234 | NC_000914 NC_012586 NC_012587 |
| Sinorhizobium medicae WSM419 | NC_009620 NC_009621 NC_009622 NC_009636 |
| Sinorhizobium meliloti 1021 | NC_003037 NC_003047 NC_003078 |
| Slackia heliotrinireducens DSM 20476 | NC_013165 |
| Sodalis glossinidius str. SINGLEQUOTEmorsitansSINGLEQUOTE | NC_007712 NC_007713 NC_007714 NC_007715 |
| Sorangium cellulosum SINGLEQUOTESo ce 56SINGLEQUOTE | NC_010162 |
| Sphaerobacter thermophilus DSM 20745 | NC_013523 NC_013524 |
| Sphingobium japonicum UT26S | NC_014005 NC_014006 NC_014007 NC_014009 NC_014013 |
| Sphingomonas wittichii RW1 | NC_009507 NC_009508 NC_009511 |
| Sphingopyxis alaskensis RB2256 | NC_008036 NC_008048 |
| Spirochaeta smaragdinae DSM 11293 | NC_014364 |
| Spirochaeta thermophila DSM 6192 | NC_014484 |
| Spirosoma linguale DSM 74 | NC_013730 NC_013731 NC_013732 NC_013733 NC_013734 NC_013735 NC_013736 NC_013737 NC_013738 |
| Stackebrandtia nassauensis DSM 44728 | NC_013947 |
| Staphylococcus aureus RF122 | NC_007622 |
| Staphylococcus aureus subsp. aureus COL | NC_002951 NC_006629 |
| Staphylococcus aureus subsp. aureus ED98 | NC_013450 NC_013451 NC_013452 NC_013453 |
| Staphylococcus aureus subsp. aureus JH1 | NC_009619 NC_009632 |
| Staphylococcus aureus subsp. aureus JH9 | NC_009477 NC_009487 |
| Staphylococcus aureus subsp. aureus MRSA252 | NC_002952 |
| Staphylococcus aureus subsp. aureus MSSA476 | NC_002953 NC_005951 |
| Staphylococcus aureus subsp. aureus MW2 | NC_003923 |
| Staphylococcus aureus subsp. aureus Mu3 | NC_009782 |
| Staphylococcus aureus subsp. aureus Mu50 | NC_002758 NC_002774 |
| Staphylococcus aureus subsp. aureus N315 | NC_002745 NC_003140 |
| Staphylococcus aureus subsp. aureus NCTC 8325 | NC_007795 |
| Staphylococcus aureus subsp. aureus USA300UNDERSCOREFPR3757 | NC_007790 NC_007791 NC_007792 NC_007793 |
| Staphylococcus aureus subsp. aureus USA300UNDERSCORETCH1516 | NC_010063 NC_010079 NC_012417 |
| Staphylococcus aureus subsp. aureus str. Newman | NC_009641 |
| Staphylococcus carnosus subsp. carnosus TM300 | NC_012121 |
| Staphylococcus epidermidis ATCC 12228 | NC_004461 NC_005003 NC_005004 NC_005005 NC_005006 NC_005007 NC_005008 |
| Staphylococcus epidermidis RP62A | NC_002976 NC_006663 |
| Staphylococcus haemolyticus JCSC1435 | NC_007168 NC_007169 NC_007170 NC_007171 |
| Staphylococcus lugdunensis HKU09-01 | NC_013893 |
| Staphylococcus saprophyticus subsp. saprophyticus ATCC 15305 | NC_007350 NC_007351 NC_007352 |
| Staphylothermus hellenicus DSM 12710 | NC_014205 |
| Staphylothermus marinus F1 | NC_009033 |
| Starkeya novella DSM 506 | NC_014217 |
| Stenotrophomonas maltophilia K279a | NC_010943 |
| Stenotrophomonas maltophilia R551-3 | NC_011071 |
| Streptobacillus moniliformis DSM 12112 | NC_013515 NC_013516 |
| Streptococcus agalactiae 2603VSLASHR | NC_004116 |
| Streptococcus agalactiae A909 | NC_007432 |
| Streptococcus agalactiae NEM316 | NC_004368 |
| Streptococcus dysgalactiae subsp. equisimilis GGSUNDERSCORE124 | NC_012891 |
| Streptococcus equi subsp. equi 4047 | NC_012471 |
| Streptococcus equi subsp. zooepidemicus | NC_012470 |
| Streptococcus equi subsp. zooepidemicus MGCS10565 | NC_011134 |
| Streptococcus gallolyticus UCN34 | NC_013798 |
| Streptococcus gordonii str. Challis substr. CH1 | NC_009785 |
| Streptococcus mitis B6 | NC_013853 |
| Streptococcus mutans NN2025 | NC_013928 |
| Streptococcus mutans UA159 | NC_004350 |
| Streptococcus pneumoniae 670-6B | NC_014498 |
| Streptococcus pneumoniae 70585 | NC_012468 |
| Streptococcus pneumoniae AP200 | NC_014494 |
| Streptococcus pneumoniae ATCC 700669 | NC_011900 |
| Streptococcus pneumoniae CGSP14 | NC_010582 |
| Streptococcus pneumoniae D39 | NC_008533 |
| Streptococcus pneumoniae G54 | NC_011072 |
| Streptococcus pneumoniae Hungary19A-6 | NC_010380 |
| Streptococcus pneumoniae JJA | NC_012466 |
| Streptococcus pneumoniae P1031 | NC_012467 |
| Streptococcus pneumoniae R6 | NC_003098 |
| Streptococcus pneumoniae TCH8431SLASH19A | NC_014251 |
| Streptococcus pneumoniae TIGR4 | NC_003028 |
| Streptococcus pneumoniae Taiwan19F-14 | NC_012469 |
| Streptococcus pyogenes M1 GAS | NC_002737 |
| Streptococcus pyogenes MGAS10270 | NC_008022 |
| Streptococcus pyogenes MGAS10394 | NC_006086 |
| Streptococcus pyogenes MGAS10750 | NC_008024 |
| Streptococcus pyogenes MGAS2096 | NC_008023 |
| Streptococcus pyogenes MGAS315 | NC_004070 |
| Streptococcus pyogenes MGAS5005 | NC_007297 |
| Streptococcus pyogenes MGAS6180 | NC_007296 |
| Streptococcus pyogenes MGAS8232 | NC_003485 |
| Streptococcus pyogenes MGAS9429 | NC_008021 |
| Streptococcus pyogenes NZ131 | NC_011375 |
| Streptococcus pyogenes SSI-1 | NC_004606 |
| Streptococcus pyogenes str. Manfredo | NC_009332 |
| Streptococcus sanguinis SK36 | NC_009009 |
| Streptococcus suis 05ZYH33 | NC_009442 |
| Streptococcus suis 98HAH33 | NC_009443 |
| Streptococcus suis BM407 | NC_012923 NC_012926 |
| Streptococcus suis P1SLASH7 | NC_012925 |
| Streptococcus suis SC84 | NC_012924 |
| Streptococcus thermophilus CNRZ1066 | NC_006449 |
| Streptococcus thermophilus LMD-9 | NC_008500 NC_008501 NC_008532 |
| Streptococcus thermophilus LMG 18311 | NC_006448 |
| Streptococcus uberis 0140J | NC_012004 |
| Streptomyces avermitilis MA-4680 | NC_003155 NC_004719 |
| Streptomyces coelicolor A3LPAREN2RPAREN | NC_003888 NC_003903 NC_003904 |
| Streptomyces griseus subsp. griseus NBRC 13350 | NC_010572 |
| Streptomyces scabiei 87.22 | NC_013929 |
| Streptosporangium roseum DSM 43021 | NC_013595 NC_013596 |
| Sulfolobus acidocaldarius DSM 639 | NC_007181 |
| Sulfolobus islandicus L.D.8.5 | NC_013769 NC_013770 |
| Sulfolobus islandicus L.S.2.15 | NC_012589 |
| Sulfolobus islandicus M.14.25 | NC_012588 |
| Sulfolobus islandicus M.16.27 | NC_012632 |
| Sulfolobus islandicus M.16.4 | NC_012726 |
| Sulfolobus islandicus Y.G.57.14 | NC_012622 |
| Sulfolobus islandicus Y.N.15.51 | NC_012623 NC_012624 |
| Sulfolobus solfataricus P2 | NC_002754 |
| Sulfolobus tokodaii str. 7 | NC_003106 |
| Sulfuricurvum kujiense DSM 16994 | NC_014754 NC_014755 NC_014756 NC_014762 NC_014763 |
| Sulfurihydrogenibium azorense Az-Fu1 | NC_012438 |
| Sulfurihydrogenibium sp. YO3AOP1 | NC_010730 |
| Sulfurimonas autotrophica DSM 16294 | NC_014506 |
| Sulfurimonas denitrificans DSM 1251 | NC_007575 |
| Sulfurospirillum deleyianum DSM 6946 | NC_013512 |
| Sulfurovum sp. NBC37-1 | NC_009663 |
| Symbiobacterium thermophilum IAM 14863 | NC_006177 |
| Synechococcus elongatus PCC 6301 | NC_006576 |
| Synechococcus elongatus PCC 7942 | NC_007595 NC_007604 |
| Synechococcus sp. CC9311 | NC_008319 |
| Synechococcus sp. CC9605 | NC_007516 |
| Synechococcus sp. CC9902 | NC_007513 |
| Synechococcus sp. JA-2-3BSINGLEQUOTEaLPAREN2-13RPAREN | NC_007776 |
| Synechococcus sp. JA-3-3Ab | NC_007775 |
| Synechococcus sp. PCC 7002 | NC_010474 NC_010475 NC_010476 NC_010477 NC_010478 NC_010479 NC_010480 |
| Synechococcus sp. RCC307 | NC_009482 |
| Synechococcus sp. WH 7803 | NC_009481 |
| Synechococcus sp. WH 8102 | NC_005070 |
| Synechocystis sp. PCC 6803 | NC_000911 NC_005229 NC_005230 NC_005231 NC_005232 |
| Syntrophobacter fumaroxidans MPOB | NC_008554 |
| Syntrophomonas wolfei subsp. wolfei str. Goettingen | NC_008346 |
| Syntrophothermus lipocalidus DSM 12680 | NC_014220 |
| Syntrophus aciditrophicus SB | NC_007759 |
| Teredinibacter turnerae T7901 | NC_012997 |
| Thauera sp. MZ1T | NC_011662 NC_011667 |
| Thermanaerovibrio acidaminovorans DSM 6589 | NC_013522 |
| Thermincola potens JR | NC_014152 |
| Thermoanaerobacter italicus Ab9 | NC_013921 |
| Thermoanaerobacter mathranii subsp. mathranii str. A3 | NC_014209 |
| Thermoanaerobacter pseudethanolicus ATCC 33223 | NC_010321 |
| Thermoanaerobacter sp. X513 | NC_014538 |
| Thermoanaerobacter sp. X514 | NC_010320 |
| Thermoanaerobacter tengcongensis MB4 LPARENCaldanaerobacter subterraneus subsp. tengcongensis MB4RPAREN | NC_003869 |
| Thermoanaerobacterium thermosaccharolyticum DSM 571 | NC_014410 |
| Thermobaculum terrenum ATCC BAA-798 | NC_013525 NC_013526 |
| Thermobifida fusca YX | NC_007333 |
| Thermobispora bispora DSM 43833 | NC_014165 |
| Thermococcus barophilus MP | NC_014804 |
| Thermococcus gammatolerans EJ3 | NC_012804 |
| Thermococcus kodakarensis KOD1 | NC_006624 |
| Thermococcus onnurineus NA1 | NC_011529 |
| Thermococcus sibiricus MM 739 | NC_012883 |
| Thermocrinis albus DSM 14484 | NC_013894 |
| Thermodesulfovibrio yellowstonii DSM 11347 | NC_011296 |
| Thermofilum pendens Hrk 5 | NC_008696 NC_008698 |
| Thermomicrobium roseum DSM 5159 | NC_011959 NC_011961 |
| Thermomonospora curvata DSM 43183 | NC_013510 |
| Thermoplasma acidophilum DSM 1728 | NC_002578 |
| Thermoplasma volcanium GSS1 | NC_002689 |
| Thermoproteus neutrophilus V24Sta | NC_010525 |
| Thermosediminibacter oceani DSM 16646 | NC_014377 |
| Thermosipho africanus TCF52B | NC_011653 |
| Thermosipho melanesiensis BI429 | NC_009616 |
| Thermosphaera aggregans DSM 11486 | NC_014160 |
| Thermosynechococcus elongatus BP-1 | NC_004113 |
| Thermotoga lettingae TMO | NC_009828 |
| Thermotoga maritima MSB8 | NC_000853 |
| Thermotoga naphthophila RKU-10 | NC_013642 |
| Thermotoga neapolitana DSM 4359 | NC_011978 |
| Thermotoga petrophila RKU-1 | NC_009486 |
| Thermotoga sp. RQ2 | NC_010483 |
| Thermus thermophilus HB27 | NC_005835 NC_005838 |
| Thermus thermophilus HB8 | NC_006461 NC_006462 NC_006463 |
| Thioalkalivibrio sp. HL-EbGR7 | NC_011901 |
| Thioalkalivibrio sp. K90mix | NC_013889 NC_013930 |
| Thiobacillus denitrificans ATCC 25259 | NC_007404 |
| Thiomicrospira crunogena XCL-2 | NC_007520 |
| Thiomonas intermedia K12 | NC_014153 NC_014154 NC_014155 |
| Tolumonas auensis DSM 9187 | NC_012691 |
| Treponema denticola ATCC 35405 | NC_002967 |
| Treponema pallidum subsp. pallidum SS14 | NC_010741 |
| Treponema pallidum subsp. pallidum str. Nichols | NC_000919 |
| Trichodesmium erythraeum IMS101 | NC_008312 |
| Tropheryma whipplei TW08SLASH27 | NC_004551 |
| Tropheryma whipplei str. Twist | NC_004572 |
| Truepera radiovictrix DSM 17093 | NC_014221 |
| Tsukamurella paurometabola DSM 20162 | NC_014158 NC_014159 |
| Ureaplasma parvum serovar 3 str. ATCC 27815 | NC_010503 |
| Ureaplasma parvum serovar 3 str. ATCC 700970 | NC_002162 |
| Ureaplasma urealyticum serovar 10 str. ATCC 33699 | NC_011374 |
| Variovorax paradoxus S110 | NC_012791 NC_012792 |
| Veillonella parvula DSM 2008 | NC_013520 |
| Verminephrobacter eiseniae EF01-2 | NC_008771 NC_008786 |
| Vibrio cholerae M66-2 | NC_012578 NC_012580 |
| Vibrio cholerae MJ-1236 | NC_012667 NC_012668 |
| Vibrio cholerae O1 biovar El Tor str. N16961 | NC_002505 NC_002506 |
| Vibrio cholerae O395 | NC_009456 NC_009457 |
| Vibrio fischeri ES114 | NC_006840 NC_006841 NC_006842 |
| Vibrio fischeri MJ11 | NC_011184 NC_011185 NC_011186 |
| Vibrio harveyi ATCC BAA-1116 | NC_009777 NC_009783 NC_009784 |
| Vibrio parahaemolyticus RIMD 2210633 | NC_004603 NC_004605 |
| Vibrio sp. Ex25 | NC_013456 NC_013457 |
| Vibrio splendidus LGP32 | NC_011744 NC_011753 |
| Vibrio vulnificus CMCP6 | NC_004459 NC_004460 |
| Vibrio vulnificus YJ016 | NC_005128 NC_005139 NC_005140 |
| Vulcanisaeta distributa DSM 14429 | NC_014537 |
| Waddlia chondrophila WSU 86-1044 | NC_014225 NC_014226 |
| Wigglesworthia glossinidia endosymbiont of Glossina brevipalpis | NC_003425 NC_004344 |
| Wolbachia endosymbiont of Culex quinquefasciatus Pel | NC_010981 |
| Wolbachia endosymbiont of Drosophila melanogaster | NC_002978 |
| Wolbachia endosymbiont strain TRS of Brugia malayi | NC_006833 |
| Wolbachia sp. wRi LPARENWolbachia endosymbiont of Drosophila simulansRPAREN | NC_012416 |
| Wolinella succinogenes DSM 1740 | NC_005090 |
| Xanthobacter autotrophicus Py2 | NC_009717 NC_009720 |
| Xanthomonas albilineans | NC_013722 |
| Xanthomonas axonopodis pv. citri str. 306 | NC_003919 NC_003921 NC_003922 |
| Xanthomonas campestris pv. campestris str. 8004 | NC_007086 |
| Xanthomonas campestris pv. campestris str. ATCC 33913 | NC_003902 |
| Xanthomonas campestris pv. campestris str. B100 | NC_010688 |
| Xanthomonas campestris pv. vesicatoria str. 85-10 | NC_007504 NC_007505 NC_007506 NC_007507 NC_007508 |
| Xanthomonas oryzae pv. oryzae KACC10331 | NC_006834 |
| Xanthomonas oryzae pv. oryzae MAFF 311018 | NC_007705 |
| Xanthomonas oryzae pv. oryzae PXO99A | NC_010717 |
| Xenorhabdus bovienii SS-2004 | NC_013892 |
| Xenorhabdus nematophila ATCC 19061 | NC_014170 NC_014228 |
| Xylanimonas cellulosilytica DSM 15894 | NC_013530 NC_013531 |
| Xylella fastidiosa 9a5c | NC_002488 NC_002489 NC_002490 |
| Xylella fastidiosa M12 | NC_010513 |
| Xylella fastidiosa M23 | NC_010577 NC_010579 |
| Xylella fastidiosa Temecula1 | NC_004554 NC_004556 |
| Yersinia enterocolitica subsp. enterocolitica 8081 | NC_008791 NC_008800 |
| Yersinia pestis Angola | NC_010157 NC_010158 NC_010159 |
| Yersinia pestis Antiqua | NC_008120 NC_008121 NC_008122 NC_008150 |
| Yersinia pestis CO92 | NC_003131 NC_003132 NC_003134 NC_003143 |
| Yersinia pestis KIM 10 | NC_004088 NC_004838 |
| Yersinia pestis Nepal516 | NC_008118 NC_008119 NC_008149 |
| Yersinia pestis Pestoides F | NC_009377 NC_009378 NC_009381 |
| Yersinia pestis Z176003 | NC_014017 NC_014022 NC_014027 NC_014029 |
| Yersinia pestis biovar Microtus str. 91001 | NC_005810 NC_005813 NC_005814 NC_005815 NC_005816 |
| Yersinia pseudotuberculosis IP 31758 | NC_009704 NC_009705 NC_009708 |
| Yersinia pseudotuberculosis IP 32953 | NC_006153 NC_006154 NC_006155 |
| Yersinia pseudotuberculosis PB1SLASH+ | NC_010634 NC_010635 |
| Yersinia pseudotuberculosis YPIII | NC_010465 |
| Zunongwangia profunda SM-A87 | NC_014041 |
| Zymomonas mobilis subsp. mobilis NCIMB 11163 | NC_013355 NC_013356 NC_013357 NC_013358 |
| Zymomonas mobilis subsp. mobilis ZM4 | NC_006526 |
| cyanobacterium UCYN-A | NC_013771 |
| gamma proteobacterium HdN1 | NC_014366 |
| uncultured methanogenic archaeon RC-I | NC_009464 |
| Draft Bacteria |  |
| Abiotrophia defectiva ATCC 49176 | NZ_ACIN02000001.1 - NZ_ACIN02000026.1 |
| Acetivibrio cellulolyticus CD2 | NZ_AEDB01000001.1 - NZ_AEDB01000193.1 |
| Achromobacter piechaudii ATCC 43553 | NZ_ADMS01000001.1 - NZ_ADMS01000154.1 |
| Acidaminococcus D21 | NZ_ACGB01000001.1 - NZ_ACGB01000079.1 |
| Acidithiobacillus caldus ATCC 51756 | NZ_ACVD01000001.1 - NZ_ACVD01000139.1 |
| Acidobacterium MP5ACTX8 | NZ_ADVX01000001.1 - NZ_ADVX01000045.1 |
| Acidovorax delafieldii 2AN | NZ_ACQT01000001.1 - NZ_ACQT01000638.1 |
| Aciduliprofundum boonei T469 | ABSD01000001.1 - ABSD01000063.1 |
| Acinetobacter 6013113 | NZ_ACYR01000001.1 - NZ_ACYR01000242.1 |
| Acinetobacter 6013150 | NZ_ACYQ01000001.1 - NZ_ACYQ01000208.1 |
| Acinetobacter 6014059 | NZ_ACYS01000001.1 - NZ_ACYS01000238.1 |
| Acinetobacter ATCC 27244 | NZ_ABYN01000001.1 - NZ_ABYN01000255.1 |
| Acinetobacter RUH2624 | ACQF01000001.1 - ACQF01000116.1 |
| Acinetobacter SH024 | ADCH01000001.1 - ADCH01000089.1 |
| Acinetobacter baumannii AB056 | NZ_ADGZ01000001.1 - NZ_ADGZ01000959.1 |
| Acinetobacter baumannii AB058 | NZ_ADHA01000001.1 - NZ_ADHA01001040.1 |
| Acinetobacter baumannii AB059 | NZ_ADHB01000001.1 - NZ_ADHB01001062.1 |
| Acinetobacter baumannii AB900 | NZ_ABXK01000001.1 - NZ_ABXK01000068.1 |
| Acinetobacter baumannii ATCC 19606 | ACQB01000001.1 - ACQB01000100.1 |
| Acinetobacter calcoaceticus RUH2202 | ACPK01000001.1 - ACPK01000081.1 |
| Acinetobacter haemolyticus ATCC 19194 | NZ_ADMT01000001.1 - NZ_ADMT01000253.1 |
| Acinetobacter johnsonii SH046 | ACPL01000001.1 - ACPL01000188.1 |
| Acinetobacter junii SH205 | ACPM01000001.1 - ACPM01000176.1 |
| Acinetobacter lwoffii SH145 | ACPN01000001.1 - ACPN01000250.1 |
| Acinetobacter radioresistens SH164 | ACPO01000001.1 - ACPO01000052.1 |
| Acinetobacter radioresistens SK82 | NZ_ACVR01000001.1 - NZ_ACVR01000082.1 |
| Actinobacillus minor 202 | NZ_ACFT01000001.1 - NZ_ACFT01000154.1 |
| Actinobacillus minor NM305 | NZ_ACQL01000001.1 - NZ_ACQL01000197.1 |
| Actinobacillus pleuropneumoniae serovar 10 D13039 | NZ_ADOJ01000001.1 - NZ_ADOJ01000044.1 |
| Actinobacillus pleuropneumoniae serovar 11 56153 | NZ_ADOK01000001.1 - NZ_ADOK01000047.1 |
| Actinobacillus pleuropneumoniae serovar 12 1096 | NZ_ADOL01000001.1 - NZ_ADOL01000063.1 |
| Actinobacillus pleuropneumoniae serovar 13 N273 | NZ_ADOM01000001.1 - NZ_ADOM01000068.1 |
| Actinobacillus pleuropneumoniae serovar 1 4074 | NZ_ADOD01000001.1 - NZ_AACK01000140.1 |
| Actinobacillus pleuropneumoniae serovar 2 4226 | NZ_ADXN01000001.1 - NZ_ADXN01000038.1 |
| Actinobacillus pleuropneumoniae serovar 2 S1536 | NZ_ADOE01000001.1 - NZ_ADOE01000056.1 |
| Actinobacillus pleuropneumoniae serovar 4 M62 | NZ_ADOF01000001.1 - NZ_ADOF01000089.1 |
| Actinobacillus pleuropneumoniae serovar 6 Femo | NZ_ADOG01000001.1 - NZ_ADXO01000036.1 |
| Actinobacillus pleuropneumoniae serovar 9 CVJ13261 | NZ_ADOI01000001.1 - NZ_ADOI01000065.1 |
| Actinomyces coleocanis DSM 15436 | NZ_ACFG01000001.1 - NZ_ACFG01000040.1 |
| Actinomyces odontolyticus ATCC 17982 | NZ_AAYI02000001.1 - NZ_AAYI02000004.1 |
| Actinomyces odontolyticus F0309 | NZ_ACYT02000001.1 - NZ_ACYT02000097.1 |
| Actinomyces oral taxon 171 F0337 | NZ_AECW01000001.1 - NZ_AECW01000580.1 |
| Actinomyces oral taxon 178 F0338 | NZ_AEUH01000001.1 - NZ_AEUH01000341.1 |
| Actinomyces oral taxon 180 F0310 | NZ_AEPP01000001.1 - NZ_AEPP01000045.1 |
| Actinomyces oral taxon 848 F0332 | NZ_ACUY02000001.1 - NZ_ACUY02000019.1 |
| Actinomyces urogenitalis DSM 15434 | NZ_ACFH01000001.1 - NZ_ACFH01000230.1 |
| Actinomyces viscosus C505 | NZ_ACRE01000001.1 - NZ_ACRE01000111.1 |
| Acyrthosiphon pisum | NC_000935.1 - NC_000935.1 |
| Aerococcus viridans ATCC 11563 | NZ_ADNT01000001.1 - NZ_ADNT01000150.1 |
| Aeromicrobium marinum DSM 15272 | NZ_ACLF03000001.1 - NZ_ACLF03000016.1 |
| Afipia 1NLS2 | NZ_ADVZ01000001.1 - NZ_ADVZ01000010.1 |
| Aggregatibacter actinomycetemcomitans D7S 1 | NZ_ADCF01000001.1 - NZ_ADCF01000001.1 |
| Aggregatibacter segnis ATCC 33393 | NZ_AEPS01000001.1 - NZ_AEPS01000019.1 |
| Ahrensia R2A130 | NZ_AEEB01000001.1 - NZ_AEEB01000059.1 |
| Alcanivorax DG881 | ABRW01000001.1 - ABRW01000070.1 |
| Algoriphagus PR1 | NZ_AAXU02000001.1 - NZ_AAXU02000001.1 |
| Alicyclobacillus acidocaldarius LAA1 | NZ_ACCS01000001.1 - NZ_ACCS01000058.1 |
| Alistipes putredinis DSM 17216 | NZ_ABFK02000001.1 - NZ_ABFK02000020.1 |
| Alteromonas macleodii ATCC 27126 | NZ_ABQB01000001.1 - NZ_ABQB01000716.1 |
| Aminomonas paucivorans DSM 12260 | AEIV01000001.1 - AEIV01000002.1 |
| Anaerobaculum hydrogeniformans ATCC BAA 1850 | NZ_ACJX02000001.1 - NZ_ACJX02000205.1 |
| Anaerococcus hydrogenalis DSM 7454 | NZ_ABXA01000001.1 - NZ_ABXA01000052.1 |
| Anaerococcus lactolyticus ATCC 51172 | NZ_ABYO01000001.1 - NZ_ABYO01000298.1 |
| Anaerococcus tetradius ATCC 35098 | NZ_ACGC01000001.1 - NZ_ACGC01000143.1 |
| Anaerococcus vaginalis ATCC 51170 | NZ_ACXU01000001.1 - NZ_ACXU01000028.1 |
| Anaerofustis stercorihominis DSM 17244 | NZ_ABIL02000001.1 - NZ_ABIL02000006.1 |
| Anaerostipes 3 2 56FAA | NZ_ACWB01000001.1 - NZ_ACWB01000114.1 |
| Anaerostipes caccae DSM 14662 | NZ_ABAX03000001.1 - NZ_ABAX03000039.1 |
| Anaerotruncus colihominis DSM 17241 | NZ_ABGD02000001.1 - NZ_ABGD02000036.1 |
| Anaplasma marginale Mississippi | NZ_ABOP01000001.1 - NZ_ABOP01000082.1 |
| Anaplasma marginale Puerto Rico | NZ_ABOQ01000001.1 - NZ_ABOQ01000059.1 |
| Anaplasma marginale Virginia | NZ_ABOR01000001.1 - NZ_ABOR01000070.1 |
| Arcobacter butzleri JV22 | NZ_AEPT01000001.1 - NZ_AEPT01000078.1 |
| Arthrospira maxima CS 328 | NZ_ABYK01000001.1 - NZ_ABYK01000129.1 |
| Arthrospira platensis Paraca | NZ_ACSK01000001.1 - NZ_ACSK01001820.1 |
| Atopobium rimae ATCC 49626 | NZ_ACFE01000001.1 - NZ_ACFE01000009.1 |
| Atopobium vaginae DSM 15829 | NZ_ADNA01000001.1 - NZ_ACGK01000061.1 |
| Atopobium vaginae PB189 T1 4 | NZ_AEDQ01000001.1 - NZ_AEDQ01000034.1 |
| Aurantimonas manganoxydans SI85 9A1 | NZ_AAPJ01000001.1 - NZ_AAPJ01000035.1 |
| Bacillus 2 A 57 CT2 | NZ_ACWD01000001.1 - NZ_ACWD01000114.1 |
| Bacillus B14905 | NZ_AAXV01000001.1 - NZ_AAXV01000099.1 |
| Bacillus BT1B CT2 | NZ_ACWC01000001.1 - NZ_ACWC01000049.1 |
| Bacillus NRRL B 14911 | NZ_AAOX01000001.1 - NZ_AAOX01000109.1 |
| Bacillus SG 1 | NZ_ABCF01000001.1 - NZ_ABCF01000186.1 |
| Bacillus anthracis A0174 | NZ_ABLT01000001.1 - NZ_ABLT01000060.1 |
| Bacillus anthracis A0193 | NZ_ABKF01000001.1 - NZ_ABKF01000060.1 |
| Bacillus anthracis A0389 | NZ_ABLB01000001.1 - NZ_ABLB01000068.1 |
| Bacillus anthracis A0442 | NZ_ABKG01000001.1 - NZ_ABKG01000046.1 |
| Bacillus anthracis A0465 | NZ_ABLH01000001.1 - NZ_ABLH01000057.1 |
| Bacillus anthracis A0488 | NZ_ABJC01000001.2 - NZ_ABJC01000063.1 |
| Bacillus anthracis A1055 | NZ_AAEO01000001.3 - NZ_AAEO01000042.1 |
| Bacillus anthracis A2012 | NZ_AAAC02000001.1 - NZ_AAAC02000001.1 |
| Bacillus anthracis Australia 94 | NZ_AAES01000001.1 - NZ_AAES01000049.1 |
| Bacillus anthracis CNEVA 9066 | NZ_AAEN01000001.1 - NZ_AAEN01000030.3 |
| Bacillus anthracis Kruger B | NZ_AAEQ01000001.1 - NZ_AAEQ01000064.3 |
| Bacillus anthracis Tsiankovskii I | NZ_ABDN02000001.1 - NZ_ABDN02000062.1 |
| Bacillus anthracis Vollum | NZ_AAEP01000001.1 - NZ_AAEP01000052.1 |
| Bacillus anthracis Western North America USA6153 | NZ_AAER01000001.1 - NZ_AAER01000044.1 |
| Bacillus cereus 03BB108 | NZ_ABDM02000001.1 - NZ_ABDM02000066.1 |
| Bacillus cereus 172560W | NZ_ACLV01000001.1 - NZ_ACLV01000152.1 |
| Bacillus cereus 95 8201 | NZ_ACMF01000001.1 - NZ_ACMF01000228.1 |
| Bacillus cereus AH1134 | NZ_ABDA02000001.1 - NZ_ABDA02000037.1 |
| Bacillus cereus AH1271 | NZ_ACMR01000001.1 - NZ_ACMR01000367.1 |
| Bacillus cereus AH1272 | NZ_ACMS01000001.1 - NZ_ACMS01000416.1 |
| Bacillus cereus AH1273 | NZ_ACMT01000001.1 - NZ_ACMT01000412.1 |
| Bacillus cereus AH603 | NZ_ACMP01000001.1 - NZ_ACMP01000251.1 |
| Bacillus cereus AH621 | NZ_ACLX01000001.1 - NZ_ACLX01000190.1 |
| Bacillus cereus AH676 | NZ_ACMQ01000001.1 - NZ_ACMQ01000315.1 |
| Bacillus cereus ATCC 10876 | NZ_ACLT01000001.1 - NZ_ACLT01000245.1 |
| Bacillus cereus ATCC 4342 | NZ_ACLZ01000001.1 - NZ_ACLZ01000105.1 |
| Bacillus cereus BDRD Cer4 | NZ_ACME01000001.1 - NZ_ACME01000117.1 |
| Bacillus cereus BDRD ST196 | NZ_ACMD01000001.1 - NZ_ACMD01000284.1 |
| Bacillus cereus BDRD ST24 | NZ_ACMB01000001.1 - NZ_ACMB01000185.1 |
| Bacillus cereus BDRD ST26 | NZ_ACMC01000001.1 - NZ_ACMC01000182.1 |
| Bacillus cereus BGSC 6E1 | NZ_ACLU01000001.1 - NZ_ACLU01000202.1 |
| Bacillus cereus F65185 | NZ_ACMO01000001.1 - NZ_ACMO01000267.1 |
| Bacillus cereus G9241 | NZ_AAEK01000001.1 - NZ_AAEK01000207.1 |
| Bacillus cereus H3081 97 | NZ_ABDL02000001.1 - NZ_ABDL02000080.1 |
| Bacillus cereus MM3 | NZ_ACLW01000001.1 - NZ_ACLW01000197.1 |
| Bacillus cereus NVH0597 99 | NZ_ABDK02000001.1 - NZ_ABDK02000071.1 |
| Bacillus cereus R309803 | NZ_ACLY01000001.1 - NZ_ACLY01000227.1 |
| Bacillus cereus Rock1 15 | NZ_ACMH01000001.1 - NZ_ACMH01000199.1 |
| Bacillus cereus Rock1 3 | NZ_ACMG01000001.1 - NZ_ACMG01000171.1 |
| Bacillus cereus Rock3 28 | NZ_ACMI01000001.1 - NZ_ACMI01001149.1 |
| Bacillus cereus Rock3 29 | NZ_ACMJ01000001.1 - NZ_ACMJ01000187.1 |
| Bacillus cereus Rock3 42 | NZ_ACMK01000001.1 - NZ_ACMK01000192.1 |
| Bacillus cereus Rock3 44 | NZ_ACML01000001.1 - NZ_ACML01001055.1 |
| Bacillus cereus Rock4 18 | NZ_ACMN01000001.1 - NZ_ACMN01000176.1 |
| Bacillus cereus Rock4 2 | NZ_ACMM01000001.1 - NZ_ACMM01000323.1 |
| Bacillus cereus SJ1 | NZ_ADFM01000001.1 - NZ_ADFM01000254.1 |
| Bacillus cereus W | NZ_ABCZ02000001.1 - NZ_ABCZ02000102.1 |
| Bacillus cereus m1293 | NZ_ACLS01000001.1 - NZ_ACLS01000241.1 |
| Bacillus cereus m1550 | NZ_ACMA01000001.1 - NZ_ACMA01000124.1 |
| Bacillus coagulans 36D1 | NZ_AAWV02000001.1 - NZ_AAWV02000004.1 |
| Bacillus coahuilensis m4 4 | NZ_ABFU01000001.1 - NZ_ABFU01000138.1 |
| Bacillus m3 13 | NZ_ACPC01000001.1 - NZ_ACPC01000050.1 |
| Bacillus mycoides DSM 2048 | NZ_ACMU01000001.1 - NZ_ACMU01000200.1 |
| Bacillus mycoides Rock1 4 | NZ_ACMV01000001.1 - NZ_ACMV01000611.1 |
| Bacillus mycoides Rock3 17 | NZ_ACMW01000001.1 - NZ_ACMW01000311.1 |
| Bacillus pseudomycoides DSM 12442 | NZ_ACMX01000001.1 - NZ_ACMX01000305.1 |
| Bacillus pumilus ATCC 7061 | NZ_ABRX01000001.1 - NZ_ABRX01000016.1 |
| Bacillus subtilis 168 | NZ_ABQK01000001.1 - NZ_ABQK01000005.1 |
| Bacillus subtilis JH642 | NZ_ABQM01000001.1 - NZ_ABQM01000009.1 |
| Bacillus subtilis NCIB 3610 | NZ_ABQL01000001.1 - NZ_ABQL01000084.1 |
| Bacillus subtilis SMY | NZ_ABQN01000001.1 - NZ_ABQN01000009.1 |
| Bacillus subtilis spizizenii ATCC 6633 | NZ_ADGS01000001.1 - NZ_ADGS01000037.1 |
| Bacillus thuringiensis Bt407 | NZ_ACMZ01000001.1 - NZ_ACMZ01000187.1 |
| Bacillus thuringiensis IBL 200 | NZ_ACNK01000001.1 - NZ_ACNK01000244.1 |
| Bacillus thuringiensis IBL 4222 | NZ_ACNL01000001.1 - NZ_ACNL01000383.1 |
| Bacillus thuringiensis serovar T01001 | NZ_ACNA01000001.1 - NZ_ACNA01000246.1 |
| Bacillus thuringiensis serovar andalousiensis BGSC 4AW1 | NZ_ACNG01000001.1 - NZ_ACNG01000257.1 |
| Bacillus thuringiensis serovar berliner ATCC 10792 | NZ_ACNF01000001.1 - NZ_ACNF01000253.1 |
| Bacillus thuringiensis serovar huazhongensis BGSC 4BD1 | NZ_ACNI01000001.1 - NZ_ACNI01000278.1 |
| Bacillus thuringiensis serovar israelensis ATCC 35646 | NZ_AAJM01000001.1 - NZ_AAJM01000866.1 |
| Bacillus thuringiensis serovar kurstaki T03a001 | NZ_ACND01000001.1 - NZ_ACND01000337.1 |
| Bacillus thuringiensis serovar monterrey BGSC 4AJ1 | NZ_ACNE01000001.1 - NZ_ACNE01000250.1 |
| Bacillus thuringiensis serovar pakistani T13001 | NZ_ACNC01000001.1 - NZ_ACNC01000617.1 |
| Bacillus thuringiensis serovar pondicheriensis BGSC 4BA1 | NZ_ACNH01000001.1 - NZ_ACNH01000185.1 |
| Bacillus thuringiensis serovar pulsiensis BGSC 4CC1 | NZ_ACNJ01000001.1 - NZ_ACNJ01000258.1 |
| Bacillus thuringiensis serovar sotto T04001 | NZ_ACNB01000001.1 - NZ_ACNB01000481.1 |
| Bacillus thuringiensis serovar tochigiensis BGSC 4Y1 | NZ_ACMY01000001.1 - NZ_ACMY01000219.1 |
| Bacteroides 1 1 14 | ACRP01000001.1 - ACRP01000189.1 |
| Bacteroides 1 1 6 | ACIC01000001.1 - ACIC01000263.1 |
| Bacteroides 20 3 | ACRQ01000001.1 - ACRQ01000125.1 |
| Bacteroides 2 1 16 | ACPP01000001.1 - ACPP01000064.1 |
| Bacteroides 2 1 22 | ACPQ01000001.1 - ACPQ01000133.1 |
| Bacteroides 2 1 33B | ACPR01000001.1 - ACPR01000068.1 |
| Bacteroides 2 1 7 | NZ_ABZY01000001.1 - NZ_ABZY01000106.1 |
| Bacteroides 2 2 4 | ABZZ01000001.1 - ABZZ01000204.1 |
| Bacteroides 3 1 19 | ADCJ01000001.1 - ADCJ01000085.1 |
| Bacteroides 3 1 23 | ACRS01000001.1 - ACRS01000087.1 |
| Bacteroides 3 1 33FAA | ACPS01000001.1 - ACPS01000099.1 |
| Bacteroides 3 1 40A | NZ_ACRT01000001.1 - NZ_ACRT01000186.1 |
| Bacteroides 3 2 5 | ACIB01000001.1 - ACIB01000115.1 |
| Bacteroides 4 1 36 | NZ_ACTC01000001.1 - NZ_ACTC01000158.1 |
| Bacteroides 4 3 47FAA | ACDR01000001.1 - ACDR01000212.1 |
| Bacteroides 9 1 42FAA | ACAA01000001.1 - ACAA01000121.1 |
| Bacteroides D1 | ACAB01000001.1 - ACAB01000209.1 |
| Bacteroides D2 | ACGA01000001.1 - ACGA01000088.1 |
| Bacteroides D20 | ACPT01000001.1 - ACPT01000062.1 |
| Bacteroides D22 | ADCK01000001.1 - ADCK01000226.1 |
| Bacteroides caccae ATCC 43185 | NZ_AAVM02000001.1 - NZ_AAVM02000021.1 |
| Bacteroides capillosus ATCC 29799 | NZ_AAXG02000001.1 - NZ_AAXG02000066.1 |
| Bacteroides cellulosilyticus DSM 14838 | NZ_ACCH01000001.1 - NZ_ACCH01000525.1 |
| Bacteroides coprocola DSM 17136 | NZ_ABIY02000001.1 - NZ_ABIY02000135.1 |
| Bacteroides coprophilus DSM 18228 | NZ_ACBW01000001.1 - NZ_ACBW01000285.1 |
| Bacteroides dorei 5 1 36 D4 | ACDI01000001.1 - ACDI01000111.1 |
| Bacteroides dorei DSM 17855 | NZ_ABWZ01000001.1 - NZ_ABWZ01000121.1 |
| Bacteroides eggerthii 1 2 48FAA | NZ_ACWG01000001.1 - NZ_ACWG01000085.1 |
| Bacteroides eggerthii DSM 20697 | NZ_ABVO01000001.1 - NZ_ABVO01000070.1 |
| Bacteroides finegoldii DSM 17565 | NZ_ABXI02000001.1 - NZ_ABXI02000138.1 |
| Bacteroides fragilis 3 1 12 | ABZX01000001.1 - ABZX01000104.1 |
| Bacteroides intestinalis DSM 17393 | NZ_ABJL02000001.1 - NZ_ABJL02000008.1 |
| Bacteroides ovatus ATCC 8483 | NZ_AAXF02000001.1 - NZ_AAXF02000054.1 |
| Bacteroides ovatus SD CC 2a | NZ_ADMP01000001.1 - NZ_ADMP01000305.1 |
| Bacteroides ovatus SD CMC 3f | NZ_ADMO01000001.1 - NZ_ADMO01000156.1 |
| Bacteroides pectinophilus ATCC 43243 | NZ_ABVQ01000001.1 - NZ_ABVQ01000037.1 |
| Bacteroides plebeius DSM 17135 | NZ_ABQC02000001.1 - NZ_ABQC02000025.1 |
| Bacteroides stercoris ATCC 43183 | NZ_ABFZ02000001.1 - NZ_ABFZ02000023.1 |
| Bacteroides uniformis ATCC 8492 | NZ_AAYH02000001.1 - NZ_AAYH02000049.1 |
| Bacteroides vulgatus EK4 2 | NZ_ADKN01000001.1 - NZ_ADKN01000079.1 |
| Bacteroides vulgatus PC510 | NZ_ADKO01000001.1 - NZ_ADKO01000117.1 |
| Bacteroides xylanisolvens SD CC 1b | NZ_ADKP01000086.1 - NZ_ADKP01000236.1 |
| Beggiatoa PS | NZ_ABBZ01000001.1 - NZ_ABBZ01006769.1 |
| Beggiatoa SS | NZ_ABBY01000001.1 - NZ_ABBY01001090.1 |
| Bermanella marisrubri | NZ_AAQH01000001.1 - NZ_AAQH01000047.1 |
| Bifidobacterium 12 1 47BFAA | NZ_ADCN01000001.1 - NZ_ADCN01000086.1 |
| Bifidobacterium adolescentis L2 32 | NZ_AAXD02000001.1 - NZ_AAXD02000074.1 |
| Bifidobacterium angulatum DSM 20098 | NZ_ABYS02000001.1 - NZ_ABYS02000017.1 |
| Bifidobacterium animalis lactis HN019 | NZ_ABOT01000001.1 - NZ_ABOT01000028.1 |
| Bifidobacterium bifidum NCIMB 41171 | ABQP01000001.1 - ABQP01000033.1 |
| Bifidobacterium breve DSM 20213 | NZ_ACCG02000001.1 - NZ_ACCG02000117.1 |
| Bifidobacterium catenulatum DSM 16992 | NZ_ABXY01000001.1 - NZ_ABXY01000031.1 |
| Bifidobacterium dentium ATCC 27678 | NZ_ABIX02000001.1 - NZ_ABIX02000002.1 |
| Bifidobacterium dentium ATCC 27679 | NZ_AEEQ01000001.1 - NZ_AEEQ01000024.1 |
| Bifidobacterium dentium JCVIHMP022 | NZ_AEHJ01000001.1 - NZ_AEHJ01000035.1 |
| Bifidobacterium gallicum DSM 20093 | NZ_ABXB03000001.1 - NZ_ABXB03000027.1 |
| Bifidobacterium longum DJO10A | NZ_AABM02000001.1 - NZ_AABM02000120.1 |
| Bifidobacterium longum infantis ATCC 55813 | NZ_ACHI01000001.1 - NZ_ACHI01000140.1 |
| Bifidobacterium longum infantis CCUG 52486 | ABQQ01000001.1 - ABQQ01000055.1 |
| Bifidobacterium pseudocatenulatum DSM 20438 | NZ_ABXX02000001.1 - NZ_ABXX02000036.1 |
| Bilophila wadsworthia 3 1 6 | NZ_ADCP01000001.1 - NZ_ADCP01000185.1 |
| Blastopirellula marina DSM 3645 | NZ_AANZ01000001.1 - NZ_AANZ01000064.1 |
| Blautia hansenii DSM 20583 | NZ_ABYU02000001.1 - NZ_ABYU02000063.1 |
| Blautia hydrogenotrophica DSM 10507 | NZ_ACBZ01000001.1 - NZ_ACBZ01000233.1 |
| Borrelia SV1 | NZ_ABJZ02000001.1 - NZ_ABJZ02000007.1 |
| Borrelia afzelii ACA 1 | NZ_ABCU02000001.1 - NZ_ABCU02000002.1 |
| Borrelia burgdorferi 118a | NZ_ABGI02000001.1 - NZ_ABGI02000008.1 |
| Borrelia burgdorferi 156a | NZ_ABCV02000001.1 - NZ_ABCV02000013.1 |
| Borrelia burgdorferi 29805 | NZ_ABJX02000001.1 - NZ_ABJX02000038.1 |
| Borrelia burgdorferi 64b | NZ_ABKA02000001.1 - NZ_ABKA02000006.1 |
| Borrelia burgdorferi 72a | NZ_ABGJ02000001.1 - NZ_ABGJ02000006.1 |
| Borrelia burgdorferi 80a | NZ_ABJU01000001.1 - NZ_ABJU01000252.1 |
| Borrelia burgdorferi 94a | NZ_ABGK02000001.1 - NZ_ABGK02000012.1 |
| Borrelia burgdorferi Bol26 | NZ_ABCW02000001.1 - NZ_ABCW02000010.1 |
| Borrelia burgdorferi CA 11 2a | NZ_ABJY02000001.1 - NZ_ABJY02000014.1 |
| Borrelia burgdorferi WI91 23 | NZ_ABJW02000001.1 - NZ_ABJW02000034.1 |
| Borrelia garinii Far04 | NZ_ABPZ02000001.1 - NZ_ABPZ02000033.1 |
| Borrelia garinii PBr | NZ_ABJV02000001.1 - NZ_ABJV02000005.1 |
| Borrelia spielmanii A14S | NZ_ABKB02000001.1 - NZ_ABKB02000041.1 |
| Borrelia valaisiana VS116 | NZ_ABCY02000001.1 - NZ_ABCY02000001.1 |
| Brevibacterium linens BL2 | NZ_AAGP01000001.1 - NZ_AAGP01000076.1 |
| Brevibacterium mcbrellneri ATCC 49030 | NZ_ADNU01000001.1 - NZ_ADNU01000096.1 |
| Brevundimonas BAL3 | ABRU01000001.1 - ABRU01000063.1 |
| Brucella 83 13 | ACBQ01000001.1 - ACBQ01000077.1 |
| Brucella BO1 | NZ_ADEZ01000001.1 - NZ_ADEZ01000055.1 |
| Brucella BO2 | NZ_ADFA01000001.1 - NZ_ADFA01000174.1 |
| Brucella F5 99 | ACFF01000001.1 - ACFF01000085.1 |
| Brucella NF 2653 | NZ_ADFB01000001.1 - NZ_ADFB01000113.1 |
| Brucella NVSL 07 0026 | ACXD01000001.1 - ACXD01000087.1 |
| Brucella abortus 2308 A | NZ_ACOR01000001.1 - NZ_ACOR01000009.1 |
| Brucella abortus NCTC 8038 | ACJJ01000001.1 - ACJJ01000045.1 |
| Brucella abortus bv 2 86 8 59 | ACBJ01000001.1 - ACBJ01000130.1 |
| Brucella abortus bv 3 Tulya | ACBI01000001.1 - ACBI01000060.1 |
| Brucella abortus bv 4 292 | ACBH01000001.1 - ACBH01000047.1 |
| Brucella abortus bv 5 B3196 | ACXC01000001.1 - ACXC01000130.1 |
| Brucella abortus bv 6 870 | ACBG01000001.1 - ACBG01000055.1 |
| Brucella abortus bv 9 C68 | ACEL01000001.1 - ACEL01000050.1 |
| Brucella ceti B1 94 | ACEK01000001.1 - ACEK01000102.1 |
| Brucella ceti Cudo | NZ_ACJD01000001.1 - NZ_ACJD01000007.1 |
| Brucella ceti M13 05 1 | ACBP01000001.1 - ACBP01000118.1 |
| Brucella ceti M490 95 1 | ACEJ01000001.1 - ACEJ01000142.1 |
| Brucella ceti M644 93 1 | ACBO01000001.1 - ACBO01000104.1 |
| Brucella melitensis bv 1 16M | ACJL01000001.1 - ACJL01000056.1 |
| Brucella melitensis bv 1 Rev 1 | ACEG01000001.1 - ACEG01000093.1 |
| Brucella melitensis bv 2 63 9 | NZ_ACEM01000001.1 - NZ_ACEM01000060.1 |
| Brucella melitensis bv 3 Ether | ACEI01000001.1 - ACEI01000105.1 |
| Brucella neotomae 5K33 | ACEH01000001.1 - ACEH01000068.1 |
| Brucella pinnipedialis B2 94 | ACBN01000001.1 - ACBN01000094.1 |
| Brucella pinnipedialis M163 99 10 | ACBM01000001.1 - ACBM01000418.1 |
| Brucella pinnipedialis M292 94 1 | ACEF01000001.1 - ACEF01000080.1 |
| Brucella suis bv 3 686 | ACBL01000001.1 - ACBL01000129.1 |
| Brucella suis bv 4 40 | ACJK01000001.1 - ACJK01000044.1 |
| Brucella suis bv 5 513 | ACBK01000001.1 - ACBK01000113.1 |
| Buchnera aphidicola LSR1 Acyrthosiphon pisum | NZ_ACFK01000001.1 - NZ_ACFK01000001.1 |
| Bulleidia extructa W1219 | NZ_ADFR01000001.1 - NZ_ADFR01000016.1 |
| Burkholderia CCGE1001 | NZ_ADDJ01000001.1 - NZ_ADDJ01000046.1 |
| Burkholderia Ch1 1 | NZ_ADNR01000001.1 - NZ_ADNR01000200.1 |
| Burkholderia H160 | NZ_ABYL01000001.1 - NZ_ABYL01000310.1 |
| Burkholderia ambifaria IOP40 10 | NZ_ABLC01000001.1 - NZ_ABLC01000629.1 |
| Burkholderia ambifaria MEX 5 | NZ_ABLK01000001.1 - NZ_ABLK01000706.1 |
| Burkholderia cenocepacia PC184 | AAKX01000001.1 - AAKX01000174.1 |
| Burkholderia dolosa AUO158 | AAKY01000001.1 - AAKY01000233.1 |
| Burkholderia graminis C4D1M | NZ_ABLD01000001.1 - NZ_ABLD01000070.1 |
| Burkholderia mallei 2002721280 | AANX02000001.1 - AANX02000208.1 |
| Burkholderia mallei ATCC 10399 | AAHN02000001.1 - AAHN02000106.1 |
| Burkholderia mallei FMH | AAIQ02000001.1 - AAIQ02000205.1 |
| Burkholderia mallei GB8 horse 4 | NZ_AAHO01000001.1 - NZ_AAHO01000181.1 |
| Burkholderia mallei JHU | AAIR02000001.1 - AAIR02000184.1 |
| Burkholderia mallei PRL 20 | NZ_AAZP01000001.1 - NZ_AAZP01000274.1 |
| Burkholderia multivorans CGD1 | NZ_ACFB01000001.1 - NZ_ACFB01000034.1 |
| Burkholderia multivorans CGD2 | NZ_ACFC01000001.1 - NZ_ACFC01000037.1 |
| Burkholderia multivorans CGD2M | NZ_ACFD01000001.1 - NZ_ACFD01000027.1 |
| Burkholderia oklahomensis C6786 | NZ_ABBG01000001.1 - NZ_ABBG01000633.1 |
| Burkholderia oklahomensis EO147 | NZ_ABBF01000001.1 - NZ_ABBF01000886.1 |
| Burkholderia pseudomallei 1106b | AAMB02000001.1 - AAMB02000113.1 |
| Burkholderia pseudomallei 112 | NZ_ABBP01000001.1 - NZ_ABBP01001274.1 |
| Burkholderia pseudomallei 14 | NZ_ABBJ01000001.1 - NZ_ABBJ01001888.1 |
| Burkholderia pseudomallei 1655 | AAHR02000001.1 - AAHR02000194.1 |
| Burkholderia pseudomallei 1710a | AAHS03000001.1 - AAHS03000108.1 |
| Burkholderia pseudomallei 305 | NZ_AAYX01000001.1 - NZ_AAYX01000036.1 |
| Burkholderia pseudomallei 406e | AAMM02000001.1 - AAMM02000271.1 |
| Burkholderia pseudomallei 576 | NZ_ACCE01000001.1 - NZ_ACCE01000021.1 |
| Burkholderia pseudomallei 7894 | NZ_ABBO01000001.1 - NZ_ABBO01001568.1 |
| Burkholderia pseudomallei 9 | NZ_ABBL01000001.1 - NZ_ABBL01001762.1 |
| Burkholderia pseudomallei 91 | NZ_ABBK01000001.1 - NZ_ABBK01001690.1 |
| Burkholderia pseudomallei B7210 | NZ_ABBN01000001.1 - NZ_ABBN01001424.1 |
| Burkholderia pseudomallei BCC215 | NZ_ABBR01000001.1 - NZ_ABBR01001030.1 |
| Burkholderia pseudomallei DM98 | NZ_ABBI01000001.1 - NZ_ABBI01002371.1 |
| Burkholderia pseudomallei MSHR346 | NZ_ACOJ01000001.1 - NZ_ACOJ01000001.1 |
| Burkholderia pseudomallei NCTC 13177 | NZ_ABBQ01000001.1 - NZ_ABBQ01001077.1 |
| Burkholderia pseudomallei Pakistan 9 | NZ_ACKA01000001.1 - NZ_ACKA01000071.1 |
| Burkholderia pseudomallei Pasteur 52237 | AAHV02000001.1 - AAHV02000217.1 |
| Burkholderia pseudomallei S13 | AAHW02000001.1 - AAHW02000169.1 |
| Burkholderia thailandensis Bt4 | NZ_ABBH01000001.1 - NZ_ABBH01000803.1 |
| Burkholderia thailandensis E264 | NZ_AACX01000001.1 - NZ_AACX01000044.1 |
| Burkholderia thailandensis MSMB43 | NZ_ABBM01000001.1 - NZ_ABBM01001230.1 |
| Burkholderia thailandensis TXDOH | NZ_ABBD01000001.1 - NZ_ABBD01000810.1 |
| Burkholderia ubonensis Bu | NZ_ABBE01000001.1 - NZ_ABBE01001143.1 |
| Butyrivibrio crossotus DSM 2876 | NZ_ABWN01000001.1 - NZ_ABWN01000051.1 |
| Caldicellulosiruptor lactoaceticus 6A | NZ_AEKD01000001.1 - NZ_AEKD01000188.1 |
| Caminibacter mediatlanticus TB 2 | NZ_ABCJ01000001.1 - NZ_ABCJ01000035.1 |
| Campylobacter coli JV20 | NZ_AEER01000001.1 - NZ_AEER01000034.1 |
| Campylobacter coli RM2228 | NZ_AAFL01000001.1 - NZ_AAFL01000038.1 |
| Campylobacter fetus venerealis Azul 94 | NZ_ACLG01000001.1 - NZ_ACLG01001187.1 |
| Campylobacter gracilis RM3268 | NZ_ACYG01000001.1 - NZ_ACYG01000033.1 |
| Campylobacter jejuni 1336 | NZ_ADGL01000001.1 - NZ_ADGL01000035.1 |
| Campylobacter jejuni 260 94 | NZ_AANK01000001.1 - NZ_AANK01000010.1 |
| Campylobacter jejuni 414 | NZ_ADGM01000001.1 - NZ_ADGM01000035.1 |
| Campylobacter jejuni 81 176 | NZ_AASL01000001.1 - NZ_AASL01000001.1 |
| Campylobacter jejuni 84 25 | NZ_AANT02000001.1 - NZ_AANT02000005.1 |
| Campylobacter jejuni BH 01 0142 | NZ_ABKD01000001.1 - NZ_ABKD01000053.1 |
| Campylobacter jejuni CF93 6 | NZ_AANJ01000001.1 - NZ_AANJ01000014.1 |
| Campylobacter jejuni CG8421 | NZ_ABGQ01000001.1 - NZ_ABGQ01000020.1 |
| Campylobacter jejuni CG8486 | NZ_AASY01000001.2 - NZ_AASY01000019.1 |
| Campylobacter jejuni HB93 13 | NZ_AANQ01000001.1 - NZ_AANQ01000035.1 |
| Campylobacter rectus RM3267 | NZ_ACFU01000001.1 - NZ_ACFU01000089.1 |
| Campylobacter showae RM3277 | NZ_ACVQ01000001.1 - NZ_ACVQ01000033.1 |
| Campylobacter upsaliensis JV21 | NZ_AEPU01000001.1 - NZ_AEPU01000049.1 |
| Campylobacter upsaliensis RM3195 | NZ_AAFJ01000001.1 - NZ_AAFJ01000020.1 |
| Candidatus Zinderia Candidatus Pelagibacter HTCC7211 | ABVS01000001.1 - ABVS01000001.1 |
| Candidatus Zinderia Candidatus Pelagibacter ubique HTCC1002 | NZ_AAPV01000001.1 - NZ_AAPV01000005.1 |
| Candidatus Zinderia Candidatus Poribacteria WGA A3 | NZ_ADFK01000001.1 - NZ_ADFK01001597.1 |
| Candidatus Zinderia Candidatus Regiella insecticola LSR1 | ACYF01000001.1 - ACYF01000181.1 |
| Candidatus Zinderia Candidatus Sulcia muelleri Hc Homalodisca coagulata | NZ_AANL01000001.1 - NZ_AANL01000066.1 |
| Capnocytophaga gingivalis ATCC 33624 | NZ_ACLQ01000001.1 - NZ_ACLQ01000037.1 |
| Capnocytophaga ochracea F0287 | NZ_AEOH01000001.1 - NZ_AEOH01000061.1 |
| Capnocytophaga sputigena ATCC 33612 | NZ_ABZV01000001.1 - NZ_ABZV01000065.1 |
| Cardiobacterium hominis ATCC 15826 | NZ_ACKY01000001.1 - NZ_ACKY01000142.1 |
| Carnobacterium AT7 | NZ_ABHH01000001.1 - NZ_ABHH01000069.1 |
| Catenibacterium mitsuokai DSM 15897 | NZ_ACCK01000001.1 - NZ_ACCK01000475.1 |
| Catonella morbi ATCC 51271 | NZ_ACIL02000001.1 - NZ_ACIL02000020.1 |
| Cellulosilyticum Clostridium lentocellum DSM 5427 | NZ_ADVF01000001.1 - NZ_ADVF01000117.1 |
| Chlamydia muridarum MopnTet14 | NZ_ACUJ01000001.3 - NZ_ACUJ01000001.3 |
| Chlamydia muridarum Nigg | NZ_ACOV01000001.1 - NZ_ACOV01000005.1 |
| Chlamydia muridarum Weiss | NZ_ACOW01000001.1 - NZ_ACOW01000005.1 |
| Chlamydia trachomatis 6276 | NZ_ABYD01000001.1 - NZ_ABYD01000001.1 |
| Chlamydia trachomatis 6276s | NZ_ABYE01000001.1 - NZ_ABYE01000001.1 |
| Chlamydia trachomatis 70 | NZ_ABYF01000001.1 - NZ_ABYF01000001.1 |
| Chlamydia trachomatis 70s | NZ_ABYG01000001.1 - NZ_ABYG01000001.1 |
| Chlamydia trachomatis D s 2923 | NZ_ACFJ01000001.1 - NZ_ACFJ01000001.1 |
| Chlamydia trachomatis L2tet1 | NZ_ACUI01000001.1 - NZ_ACUI01000001.1 |
| Chlorobium ferrooxidans DSM 13031 | NZ_AASE01000001.1 - NZ_AASE01000047.1 |
| Chryseobacterium gleum ATCC 35910 | NZ_ACKQ02000001.1 - NZ_ACKQ02000007.1 |
| Chthoniobacter flavus Ellin428 | NZ_ABVL01000001.1 - NZ_ABVL01000062.1 |
| Citrobacter 30 2 | ACDJ01000001.1 - ACDJ01000061.1 |
| Citrobacter youngae ATCC 29220 | NZ_ABWL02000001.1 - NZ_ABWL02000049.1 |
| Citromicrobium bathyomarinum JL354 | NZ_ADAE01000001.1 - NZ_ADAE01000068.1 |
| Clostridium 7 2 43FAA | ACDK01000001.1 - ACDK01000132.1 |
| Clostridium HGF2 | NZ_AENW01000001.1 - NZ_AENW01000060.1 |
| Clostridium L2 50 | NZ_AAYW02000001.1 - NZ_AAYW02000025.1 |
| Clostridium M62 1 | NZ_ACFX02000001.1 - NZ_ACFX02000085.1 |
| Clostridium SS2 1 | NZ_ABGC03000001.1 - NZ_ABGC03000042.1 |
| Clostridium asparagiforme DSM 15981 | NZ_ACCJ01000001.1 - NZ_ACCJ01000640.1 |
| Clostridium bartlettii DSM 16795 | NZ_ABEZ02000001.1 - NZ_ABEZ02000022.1 |
| Clostridium bolteae ATCC BAA 613 | NZ_ABCC02000001.1 - NZ_ABCC02000078.1 |
| Clostridium botulinum Bf | NZ_ABDP01000001.1 - NZ_ABDP01000070.1 |
| Clostridium botulinum C Eklund | NZ_ABDQ01000001.1 - NZ_ABDQ01000076.1 |
| Clostridium botulinum D 1873 | NZ_ACSJ01000001.1 - NZ_ACSJ01000019.1 |
| Clostridium botulinum E1 BoNT E Beluga | NZ_ACSC01000001.1 - NZ_ACSC01000006.1 |
| Clostridium botulinum NCTC 2916 | NZ_ABDO02000001.1 - NZ_ABDO02000049.1 |
| Clostridium butyricum 5521 | NZ_ABDT01000001.2 - NZ_ABDT01000123.2 |
| Clostridium butyricum E4 BoNT E BL5262 | NZ_ACOM01000001.1 - NZ_ACOM01000013.1 |
| Clostridium carboxidivorans P7 | ADEK01000001.1 - NZ_ACVI01000252.1 |
| Clostridium cellulovorans 743B | NZ_BABR01000001.1 - NZ_BABR01000546.1 |
| Clostridium difficile BI9 | NZ_ABKJ02000001.1 - NZ_ABKJ02000055.1 |
| Clostridium difficile CIP 107932 | NZ_ABKK02000001.1 - NZ_ABKK02000055.1 |
| Clostridium difficile NAP07 | NZ_ADVM01000001.1 - NZ_ADVM01000100.1 |
| Clostridium difficile NAP08 | NZ_ADNX01000001.1 - NZ_ADNX01000111.1 |
| Clostridium difficile QCD 23m63 | NZ_ABKL02000001.1 - NZ_ABKL02000061.1 |
| Clostridium difficile QCD 32g58 | NZ_AAML04000001.1 - NZ_AAML04000016.1 |
| Clostridium difficile QCD 37x79 | NZ_ABHG02000001.1 - NZ_ABHG02000045.1 |
| Clostridium difficile QCD 63q42 | NZ_ABHD02000001.1 - NZ_ABHD02000060.1 |
| Clostridium difficile QCD 66c26 | NZ_ABFD02000001.1 - NZ_CM000441.1 |
| Clostridium difficile QCD 76w55 | NZ_ABHE02000001.1 - NZ_ABHE02000066.1 |
| Clostridium difficile QCD 97b34 | NZ_ABHF02000001.1 - NZ_ABHF02000060.1 |
| Clostridium hathewayi DSM 13479 | NZ_ACIO01000001.1 - NZ_ACIO01001294.1 |
| Clostridium hiranonis DSM 13275 | NZ_ABWP01000001.1 - NZ_ABWP01000101.1 |
| Clostridium hylemonae DSM 15053 | NZ_ABYI02000001.1 - NZ_ABYI02000167.1 |
| Clostridium leptum DSM 753 | NZ_ABCB02000001.1 - NZ_ABCB02000022.1 |
| Clostridium methylpentosum DSM 5476 | NZ_ACEC01000001.1 - NZ_ACEC01000146.1 |
| Clostridium nexile DSM 1787 | NZ_ABWO01000001.1 - NZ_ABWO01000316.1 |
| Clostridium papyrosolvens DSM 2782 | NZ_ACXX01000001.1 - NZ_ACXX01000121.1 |
| Clostridium perfringens B ATCC 3626 | NZ_ABDV01000001.1 - NZ_ABDV01000098.1 |
| Clostridium perfringens CPE F4969 | NZ_ABDX01000001.1 - NZ_ABDX01000074.1 |
| Clostridium perfringens C JGS1495 | NZ_ABDU01000001.2 - NZ_ABDU01000084.2 |
| Clostridium perfringens D JGS1721 | NZ_ABOO01000001.1 - NZ_ABOO01000221.1 |
| Clostridium perfringens E JGS1987 | NZ_ABDW01000001.1 - NZ_ABDW01000101.1 |
| Clostridium perfringens NCTC 8239 | NZ_ABDY01000001.1 - NZ_ABDY01000055.1 |
| Clostridium scindens ATCC 35704 | NZ_ABFY02000001.1 - NZ_ABFY02000068.1 |
| Clostridium sporogenes ATCC 15579 | NZ_ABKW02000001.1 - NZ_ABKW02000004.1 |
| Clostridium thermocellum DSM 2360 | NZ_ACVX01000001.1 - NZ_ACVX01000110.1 |
| Clostridium thermocellum JW20 | NZ_ABVG02000001.1 - NZ_ABVG02000021.1 |
| Collinsella aerofaciens ATCC 25986 | NZ_AAVN02000001.1 - NZ_AAVN02000025.1 |
| Collinsella intestinalis DSM 13280 | NZ_ABXH02000001.1 - NZ_ABXH02000055.1 |
| Collinsella stercoris DSM 13279 | NZ_ABXJ01000001.1 - NZ_ABXJ01000177.1 |
| Comamonas testosteroni KF 1 | NZ_AAUJ02000001.1 - NZ_AAUJ02000001.1 |
| Comamonas testosteroni S44 | NZ_ADVQ01000001.1 - NZ_ADVQ01000112.1 |
| Congregibacter litoralis KT71 | NZ_AAOA01000001.1 - NZ_AAOA01000032.1 |
| Coprobacillus 29 1 | NZ_ADKX01000001.1 - NZ_ADKX01000080.1 |
| Coprobacillus D7 | ACDT01000001.1 - ACDT01000260.1 |
| Coprococcus comes ATCC 27758 | NZ_ABVR01000001.1 - NZ_ABVR01000046.1 |
| Coprococcus eutactus ATCC 27759 | NZ_ABEY02000001.1 - NZ_ABEY02000032.1 |
| Corynebacterium accolens ATCC 49725 | NZ_ACGD01000001.1 - NZ_ACGD01000064.1 |
| Corynebacterium accolens ATCC 49726 | NZ_AEED01000001.1 - NZ_AEED01000165.1 |
| Corynebacterium ammoniagenes DSM 20306 | NZ_ADNS01000001.1 - NZ_ADNS01000034.1 |
| Corynebacterium amycolatum SK46 | NZ_ABZU01000001.1 - NZ_ABZU01000048.1 |
| Corynebacterium aurimucosum ATCC 700975 | NZ_ACLH01000001.1 - NZ_ACLH01000101.1 |
| Corynebacterium efficiens YS 314 | NZ_ACLI01000001.1 - NZ_ACLI01000137.1 |
| Corynebacterium genitalium ATCC 33030 | NZ_ACLJ02000001.1 - NZ_ACLJ02000003.1 |
| Corynebacterium glucuronolyticum ATCC 51866 | NZ_ACHF01000001.1 - NZ_ACHF01000142.1 |
| Corynebacterium glucuronolyticum ATCC 51867 | NZ_ABYP01000001.1 - NZ_ABYP01000106.1 |
| Corynebacterium jeikeium ATCC 43734 | NZ_ACYW01000001.1 - NZ_ACYW01000093.1 |
| Corynebacterium lipophiloflavum DSM 44291 | NZ_ACHJ01000001.1 - NZ_ACHJ01000178.1 |
| Corynebacterium matruchotii ATCC 14266 | NZ_ACSH02000001.1 - NZ_ACSH02000008.1 |
| Corynebacterium matruchotii ATCC 33806 | NZ_ACEB01000001.1 - NZ_ACEB01000058.1 |
| Corynebacterium pseudogenitalium ATCC 33035 | NZ_ABYQ02000001.1 - NZ_ABYQ02000016.1 |
| Corynebacterium resistens DSM 45100 | NZ_ADGN01000001.1 - NZ_ADGN01000082.1 |
| Corynebacterium striatum ATCC 6940 | NZ_ACGE01000001.1 - NZ_ACGE01000176.1 |
| Corynebacterium tuberculostearicum SK141 | NZ_ACVP01000001.1 - NZ_ACVP01000037.1 |
| Corynebacterium variabile DSM 44702 | NZ_AEAU01000001.1 - NZ_AEAU01000126.1 |
| Coxiella burnetii MSU Goat Q177 | NZ_AAUP02000001.2 - NZ_AAUP02000009.2 |
| Coxiella burnetii RSA 334 | NZ_AAYJ01000001.1 - NZ_AAYJ01000148.1 |
| Crocosphaera watsonii WH 8501 | NZ_AADV02000001.1 - NZ_AADV02000323.1 |
| Cyanobium PCC 7001 | ABSE01000001.1 - ABSE01000018.1 |
| Cyanothece CCY0110 | NZ_AAXW01000001.1 - NZ_AAXW01000163.1 |
| Cylindrospermopsis raciborskii CS 505 | NZ_ACYA01000001.1 - NZ_ACYA01000093.1 |
| Dermacoccus Ellin185 | NZ_AEIQ01000001.1 - NZ_AEIQ01000131.1 |
| Desulfonatronospira thiodismutans ASO3 1 | NZ_ACJN02000001.1 - NZ_ACJN02000005.1 |
| Desulfovibrio 3 1 syn3 | ADDR01000001.1 - ADDR01000287.1 |
| Desulfovibrio FW1012B | NZ_ADFE01000001.1 - NZ_ADFE01000097.1 |
| Desulfovibrio fructosovorans JJ | NZ_AECZ01000001.1 - NZ_AECZ01000075.1 |
| Desulfovibrio piger ATCC 29098 | NZ_ABXU01000001.1 - NZ_ABXU01000127.1 |
| Desulfuromonas acetoxidans DSM 684 | NZ_AAEW02000001.1 - NZ_AAEW02000051.1 |
| Dethiobacter alkaliphilus AHT 1 | NZ_ACJM01000001.1 - NZ_ACJM01000034.1 |
| Dethiosulfovibrio peptidovorans DSM 11002 | NZ_ABTR02000001.1 - NZ_ABTR02000003.1 |
| Dialister invisus DSM 15470 | NZ_ACIM02000001.1 - NZ_ACIM02000002.1 |
| Dialister microaerophilus UPII 345 E | NZ_AENT01000001.1 - NZ_AENT01000032.1 |
| Dietzia cinnamea P4 | NZ_AEKG01000001.1 - NZ_AEKG01000428.1 |
| Dokdonia donghaensis MED134 | NZ_AAMZ01000001.1 - NZ_AAMZ01000017.1 |
| Dorea formicigenerans ATCC 27755 | NZ_AAXA02000001.1 - NZ_AAXA02000016.1 |
| Dorea longicatena DSM 13814 | NZ_AAXB02000001.1 - NZ_AAXB02000052.1 |
| Edwardsiella tarda ATCC 23685 | NZ_ADGK01000001.1 - NZ_ADGK01000372.1 |
| Eggerthella 1 3 56FAA | NZ_ACWN01000001.1 - NZ_ACWN01000159.1 |
| Ehrlichia chaffeensis Sapulpa | NZ_AAIF01000001.1 - NZ_AAIF01000125.1 |
| Eikenella corrodens ATCC 23834 | NZ_ACEA01000001.1 - NZ_ACEA01000065.1 |
| Enhydrobacter aerosaccus SK60 | NZ_ACYI01000001.1 - NZ_ACYI01000088.1 |
| Enterobacter cancerogenus ATCC 35316 | NZ_ABWM02000001.1 - NZ_ABWM02000062.1 |
| Enterococcus casseliflavus EC10 | ACAL01000001.1 - ACAL01000054.1 |
| Enterococcus casseliflavus EC20 | ACAO01000001.1 - ACAO01000057.1 |
| Enterococcus casseliflavus EC30 | ACAH01000001.1 - ACAH01000079.1 |
| Enterococcus faecalis ARO1 DG | ACAK01000001.1 - ACAK01000039.1 |
| Enterococcus faecalis ATCC 29200 | NZ_ACHK01000001.1 - NZ_ACHK01000123.1 |
| Enterococcus faecalis ATCC 4200 | ACAG01000001.1 - ACAG01000083.1 |
| Enterococcus faecalis CH188 | ACAV01000001.1 - ACAV01000120.1 |
| Enterococcus faecalis D6 | ACAT01000001.1 - ACAT01000046.1 |
| Enterococcus faecalis DAPTO 512 | NZ_AEBT01000001.1 - NZ_AEBT01000087.1 |
| Enterococcus faecalis DAPTO 516 | NZ_AEBS01000001.1 - NZ_AEBS01000097.1 |
| Enterococcus faecalis DS5 | ACAI01000001.1 - ACAI01000128.1 |
| Enterococcus faecalis E1Sol | ACAQ01000001.1 - ACAQ01000075.1 |
| Enterococcus faecalis Fly1 | ACAR01000001.1 - ACAR01000106.1 |
| Enterococcus faecalis HH22 | NZ_ACIX01000001.1 - NZ_ACIX01000307.1 |
| Enterococcus faecalis HIP11704 | ACAN01000001.1 - ACAN01000143.1 |
| Enterococcus faecalis JH1 | ACAP01000001.1 - ACAP01000112.1 |
| Enterococcus faecalis Merz96 | ACAM01000001.1 - ACAM01000106.1 |
| Enterococcus faecalis OG1RF | NZ_ABPI01000001.1 - NZ_ABPI01000001.1 |
| Enterococcus faecalis PC1 1 | NZ_ADMM01000001.1 - NZ_ADMM01000078.1 |
| Enterococcus faecalis R712 | NZ_ADDQ01000001.1 - NZ_ADDQ01000132.1 |
| Enterococcus faecalis S613 | NZ_ADDP01000001.1 - NZ_ADDP01000145.1 |
| Enterococcus faecalis T1 | ACAD01000001.1 - ACAD01000082.1 |
| Enterococcus faecalis T11 | ACAU01000001.1 - ACAU01000049.1 |
| Enterococcus faecalis T2 | ACAE01000001.1 - ACAE01000121.1 |
| Enterococcus faecalis T3 | ACAF01000001.1 - ACAF01000040.1 |
| Enterococcus faecalis T8 | ACOC01000001.1 - ACOC01000115.1 |
| Enterococcus faecalis TUSoD Ef11 | NZ_ACOX02000001.1 - NZ_ACOX02000011.1 |
| Enterococcus faecalis TX0102 | NZ_AEBD01000001.1 - NZ_AEBD01000083.1 |
| Enterococcus faecalis TX0104 | NZ_ACGL01000001.1 - NZ_ACGL01000237.1 |
| Enterococcus faecalis TX0109 | NZ_AEBY01000001.1 - NZ_AEBY01000101.1 |
| Enterococcus faecalis TX0411 | NZ_AECA01000001.1 - NZ_AECA01000113.1 |
| Enterococcus faecalis TX0470 | NZ_AECC01000001.1 - NZ_AECC01000086.1 |
| Enterococcus faecalis TX0635 | NZ_AEBZ01000001.1 - NZ_AEBZ01000097.1 |
| Enterococcus faecalis TX0855 | NZ_AEBV01000001.1 - NZ_AEBV01000093.1 |
| Enterococcus faecalis TX0860 | NZ_AEBX01000001.1 - NZ_AEBX01000088.1 |
| Enterococcus faecalis TX1322 | NZ_ACGM01000001.1 - NZ_ACGM01000116.1 |
| Enterococcus faecalis TX2134 | NZ_AEBW01000001.1 - NZ_AEBW01000098.1 |
| Enterococcus faecalis TX4248 | NZ_AEBR01000001.1 - NZ_AEBR01000112.1 |
| Enterococcus faecalis X98 | ACAW01000001.1 - ACAW01000077.1 |
| Enterococcus faecium 1 141 733 | ACAZ01000001.1 - ACAZ01000101.1 |
| Enterococcus faecium 1 230 933 | ACAS01000001.1 - ACAS01000304.1 |
| Enterococcus faecium 1 231 408 | ACBB01000001.1 - ACBB01000379.1 |
| Enterococcus faecium 1 231 410 | ACBA01000001.1 - ACBA01000230.1 |
| Enterococcus faecium 1 231 501 | ACAY01000001.1 - ACAY01000140.1 |
| Enterococcus faecium 1 231 502 | ACAX01000001.1 - ACAX01000220.1 |
| Enterococcus faecium C68 | ACJQ01000001.1 - ACJQ01000182.1 |
| Enterococcus faecium Com12 | ACBC01000001.1 - ACBC01000067.1 |
| Enterococcus faecium Com15 | ACBD01000001.1 - ACBD01000070.1 |
| Enterococcus faecium D344SRF | NZ_ACZZ01000001.1 - NZ_ACZZ01000215.1 |
| Enterococcus faecium DO | NZ_AAAK03000001.1 - NZ_ACIY01000623.1 |
| Enterococcus faecium E1039 | NZ_ACOS01000001.1 - NZ_ACOS01000124.1 |
| Enterococcus faecium E1071 | NZ_ABQI01000001.1 - NZ_ABQI01000096.1 |
| Enterococcus faecium E1162 | NZ_ABQJ01000001.1 - NZ_ABQJ01000139.1 |
| Enterococcus faecium E1636 | NZ_ABRY01000001.1 - NZ_ABRY01000223.1 |
| Enterococcus faecium E1679 | NZ_ABSC01000001.1 - NZ_ABSC01000340.1 |
| Enterococcus faecium E980 | NZ_ABQA01000001.1 - NZ_ABQA01000131.1 |
| Enterococcus faecium TC 6 | ACOB01000001.1 - ACOB01000278.1 |
| Enterococcus faecium TX0082 | NZ_AEBU01000001.1 - NZ_AEBU01000151.1 |
| Enterococcus faecium TX0133A | NZ_AECH01000001.1 - NZ_AECH01000223.1 |
| Enterococcus faecium TX0133B | NZ_AECI01000001.1 - NZ_AECI01000221.1 |
| Enterococcus faecium TX0133C | NZ_AEBG01000001.1 - NZ_AEBG01000215.1 |
| Enterococcus faecium TX0133a01 | NZ_AECJ01000001.1 - NZ_AECJ01000252.1 |
| Enterococcus faecium TX0133a04 | NZ_AEBC01000001.1 - NZ_AEBC01000211.1 |
| Enterococcus faecium TX1330 | NZ_ACHL01000001.1 - NZ_ACHL01000156.1 |
| Enterococcus faecium U0317 | NZ_ABSW01000001.1 - NZ_ABSW01000227.1 |
| Enterococcus gallinarum EG2 | ACAJ01000001.1 - ACAJ01000049.1 |
| Enterococcus italicus DSM 15952 | NZ_AEPV01000001.1 - NZ_AEPV01000157.1 |
| Epulopiscium N t morphotype B | NZ_ABEQ01000001.3 - NZ_ABEQ01000092.3 |
| Eremococcus coleocola ACS 139 V Col8 | NZ_AENN01000001.1 - NZ_AENN01000021.1 |
| Erysipelothrix rhusiopathiae ATCC 19414 | NZ_ACLK01000001.1 - NZ_ACLK01000031.1 |
| Erythrobacter NAP1 | NZ_AAMW01000001.1 - NZ_AAMW01000004.1 |
| Erythrobacter SD 21 | NZ_ABCG01000001.1 - NZ_ABCG01000019.1 |
| Escherichia albertii TW07627 | NZ_ABKX01000001.1 - NZ_ABKX01000064.1 |
| Escherichia coli 101 1 | NZ_AAMK02000001.1 - NZ_AAMK02000091.1 |
| Escherichia coli 1827 70 | NZ_ADUK01000001.1 - NZ_ADUK01000035.1 |
| Escherichia coli 2362 75 | NZ_ADUL01000001.1 - NZ_ADUL01000083.1 |
| Escherichia coli 53638 | NZ_AAKB02000001.1 - NZ_AAKB02000002.1 |
| Escherichia coli 83972 | NZ_ACGN01000001.1 - NZ_ACGN01000159.1 |
| Escherichia coli B088 | ACXE01000001.1 - ACXE01000148.1 |
| Escherichia coli B171 | NZ_AAJX02000001.1 - NZ_AAJX02000262.1 |
| Escherichia coli B185 | ACXF01000001.1 - ACXF01000115.1 |
| Escherichia coli B354 | ACXG01000001.1 - ACXG01000069.1 |
| Escherichia coli B7A | NZ_AAJT02000001.1 - NZ_AAJT02000289.1 |
| Escherichia coli E110019 | NZ_AAJW02000001.1 - NZ_AAJW02000137.1 |
| Escherichia coli E22 | NZ_AAJV02000001.1 - NZ_AAJV02000127.1 |
| Escherichia coli F11 | NZ_AAJU02000001.1 - NZ_AAJU02000119.1 |
| Escherichia coli FVEC1302 | ACXH01000001.1 - ACXH01000149.1 |
| Escherichia coli FVEC1412 | ACXI01000001.1 - ACXI01000137.1 |
| Escherichia coli H299 | NZ_ADBC01000001.1 - NZ_ADBC01000229.1 |
| Escherichia coli H591 | NZ_ADBB01000001.1 - NZ_ADBB01000190.1 |
| Escherichia coli H736 | NZ_ADAU01000001.1 - NZ_ADAU01000135.1 |
| Escherichia coli KO11 | NZ_AEDM01000001.1 - NZ_AEDM01000127.1 |
| Escherichia coli M605 | NZ_ADAV01000001.1 - NZ_ADAV01000162.1 |
| Escherichia coli M718 | NZ_ADAW01000001.1 - NZ_ADAW01000188.1 |
| Escherichia coli MS 107 1 | NZ_ADWV01000001.1 - NZ_ADWV01000072.1 |
| Escherichia coli MS 115 1 | NZ_ADTL01000001.1 - NZ_ADTL01000423.1 |
| Escherichia coli MS 116 1 | NZ_ADTZ01000001.1 - NZ_ADTZ01000484.1 |
| Escherichia coli MS 119 7 | NZ_ADWU01000001.1 - NZ_ADWU01000130.1 |
| Escherichia coli MS 124 1 | NZ_ADWT01000001.1 - NZ_ADWT01000176.1 |
| Escherichia coli MS 145 7 | NZ_ADWS01000001.1 - NZ_ADWS01000143.1 |
| Escherichia coli MS 146 1 | NZ_ADTN01000001.1 - NZ_ADTN01000355.1 |
| Escherichia coli MS 175 1 | NZ_ADUB01000001.1 - NZ_ADUB01000413.1 |
| Escherichia coli MS 182 1 | NZ_ADTM01000001.1 - NZ_ADTM01000456.1 |
| Escherichia coli MS 185 1 | NZ_ADUE01000001.1 - NZ_ADUE01000378.1 |
| Escherichia coli MS 187 1 | NZ_ADTQ01000001.1 - NZ_ADTQ01000310.1 |
| Escherichia coli MS 196 1 | NZ_ADUD01000001.1 - NZ_ADUD01000785.1 |
| Escherichia coli MS 198 1 | NZ_ADTJ01000001.1 - NZ_ADTJ01000564.1 |
| Escherichia coli MS 200 1 | NZ_ADUC01000001.1 - NZ_ADUC01000445.1 |
| Escherichia coli MS 21 1 | NZ_ADTR01000001.1 - NZ_ADTR01000540.1 |
| Escherichia coli MS 45 1 | NZ_ADTO01000001.1 - NZ_ADTO01000396.1 |
| Escherichia coli MS 69 1 | NZ_ADTP01000001.1 - NZ_ADTP01000425.1 |
| Escherichia coli MS 78 1 | NZ_ADTY01000001.1 - NZ_ADTY01000410.1 |
| Escherichia coli MS 84 1 | NZ_ADTK01000001.1 - NZ_ADTK01000432.1 |
| Escherichia coli NC101 | NZ_AEFA01000001.1 - NZ_AEFA01000027.1 |
| Escherichia coli O157 H7 EC4024 | NZ_ABJT01000001.1 - NZ_ABJT01000364.1 |
| Escherichia coli O157 H7 EC4042 | NZ_ABHM02000001.1 - NZ_ABHM02000004.1 |
| Escherichia coli O157 H7 EC4045 | NZ_ABHL02000001.1 - NZ_ABHL02000008.1 |
| Escherichia coli O157 H7 EC4076 | NZ_ABHQ01000001.1 - NZ_ABHQ01000135.1 |
| Escherichia coli O157 H7 EC4113 | NZ_ABHP01000001.1 - NZ_ABHP01000231.1 |
| Escherichia coli O157 H7 EC4196 | NZ_ABHO01000001.1 - NZ_ABHO01000186.1 |
| Escherichia coli O157 H7 EC4206 | NZ_ABHK02000001.1 - NZ_ABHK02000007.1 |
| Escherichia coli O157 H7 EC4401 | NZ_ABHR01000001.1 - NZ_ABHR01000186.1 |
| Escherichia coli O157 H7 EC4486 | NZ_ABHS01000001.1 - NZ_ABHS01000165.1 |
| Escherichia coli O157 H7 EC4501 | NZ_ABHT01000001.1 - NZ_ABHT01000250.1 |
| Escherichia coli O157 H7 EC508 | NZ_ABHW01000001.1 - NZ_ABHW01000272.1 |
| Escherichia coli O157 H7 EC869 | NZ_ABHU01000001.1 - NZ_ABHU01000147.1 |
| Escherichia coli O157 H7 FRIK2000 | NZ_ACXO01000001.1 - NZ_ACXO01000247.1 |
| Escherichia coli O157 H7 FRIK966 | NZ_ACXN01000001.1 - NZ_ACXN01000316.1 |
| Escherichia coli O157 H7 TW14588 | NZ_ABKY02000001.1 - NZ_ABKY02000010.1 |
| Escherichia coli OP50 | NZ_ADBT01000001.1 - NZ_ADBT01002939.1 |
| Escherichia coli TA143 | NZ_ADAY01000001.1 - NZ_ADAY01000168.1 |
| Escherichia coli TA206 | NZ_ADAX01000001.1 - NZ_ADAX01000187.1 |
| Escherichia coli TA271 | NZ_ADAZ01000001.1 - NZ_ADAZ01000191.1 |
| Escherichia coli TA280 | NZ_ADBA01000001.1 - NZ_ADBA01000168.1 |
| Escherichia coli W | NZ_AEDF01000001.1 - NZ_AEDF01000088.1 |
| Eubacterium cellulosolvens 6 | NZ_AEOA01000001.1 - NZ_AEOA01000107.1 |
| Eubacterium hallii DSM 3353 | NZ_ACEP01000001.1 - NZ_ACEP01000175.1 |
| Eubacterium saburreum DSM 3986 | NZ_AEPW01000001.1 - NZ_AEPW01000150.1 |
| Eubacterium saphenum ATCC 49989 | NZ_ACON01000001.1 - NZ_ACON01000005.1 |
| Eubacterium siraeum DSM 15702 | NZ_ABCA03000001.1 - NZ_ABCA03000055.1 |
| Eubacterium ventriosum ATCC 27560 | NZ_AAVL02000001.1 - NZ_AAVL02000038.1 |
| Eubacterium yurii margaretiae ATCC 43715 | NZ_AEES01000001.1 - NZ_AEES01000085.1 |
| Faecalibacterium cf prausnitzii KLE1255 | NZ_AECU01000001.1 - NZ_AECU01000249.1 |
| Faecalibacterium prausnitzii A2 165 | NZ_ACOP02000001.1 - NZ_ACOP02000115.1 |
| Faecalibacterium prausnitzii M21 2 | NZ_ABED02000001.1 - NZ_ABED02000029.1 |
| Ferroplasma acidarmanus fer1 | NZ_AABC05000001.1 - NZ_AABC05000018.1 |
| Finegoldia magna ACS 171 V Col3 | NZ_AECM01000001.1 - NZ_AECM01000030.1 |
| Finegoldia magna ATCC 53516 | NZ_ACHM02000001.1 - NZ_ACHM02000003.1 |
| Finegoldia magna BVS033A4 | NZ_AEDP01000001.1 - NZ_AEDP01000050.1 |
| Francisella novicida FTE | NZ_ABSS01000001.1 - NZ_ABSS01000008.1 |
| Francisella novicida FTG | NZ_ABXZ01000001.1 - NZ_ABXZ01000008.1 |
| Francisella novicida GA99 3548 | ABAH01000001.1 - ABAH01000018.1 |
| Francisella novicida GA99 3549 | AAYF01000001.1 - AAYF01000015.1 |
| Francisella philomiragia ATCC 25015 | ABYY01000001.1 - ABYY01000030.1 |
| Francisella tularensis FSC033 | AAYE01000001.1 - AAYE01000015.1 |
| Francisella tularensis MA00 2987 | ABRI01000001.1 - ABRI01000033.1 |
| Francisella tularensis holarctica 257 | AAUD01000001.1 - AAUD01000031.1 |
| Francisella tularensis holarctica FSC022 | AAYD01000001.1 - AAYD01000019.1 |
| Francisella tularensis holarctica FSC200 | NZ_AASP01000001.1 - NZ_AASP01000039.1 |
| Francisella tularensis holarctica URFT1 | NZ_ABAZ01000001.1 - NZ_ABAZ01000480.1 |
| Frankia EUN1f | NZ_ADGX01000001.1 - NZ_ADGX01000396.1 |
| Frankia symbiont of Datisca glomerata | NZ_ADGT01000001.1 - NZ_ADGT01000237.1 |
| Fulvimarina pelagi HTCC2506 | NZ_AATP01000001.1 - NZ_AATP01000020.1 |
| Fusobacterium 1 1 41FAA | ADGG01000001.1 - ADGG01000080.1 |
| Fusobacterium 2 1 31 | ACDC01000001.1 - ACDC01000202.1 |
| Fusobacterium 3 1 27 | ADGF01000001.1 - ADGF01000077.1 |
| Fusobacterium 3 1 33 | ACQE01000001.1 - ACQE01000198.1 |
| Fusobacterium 3 1 36A2 | ACPU01000001.1 - ACPU01000051.1 |
| Fusobacterium 3 1 5R | ACDD01000001.1 - ACDD01000099.1 |
| Fusobacterium 4 1 13 | ACDE01000001.1 - ACDE01000050.1 |
| Fusobacterium 7 1 | ACDF01000001.1 - ACDF01000095.1 |
| Fusobacterium D11 | ACDS01000001.1 - ACDS01000355.1 |
| Fusobacterium D12 | ACDG01000001.1 - ACDG01000224.1 |
| Fusobacterium gonidiaformans ATCC 25563 | ACET01000001.1 - ACET01000055.1 |
| Fusobacterium mortiferum ATCC 9817 | ACDB01000001.1 - ACDB01000080.1 |
| Fusobacterium nucleatum ATCC 23726 | NZ_ADVK01000001.1 - NZ_ADVK01000067.1 |
| Fusobacterium nucleatum polymorphum ATCC 10953 | AARG01000001.1 - AARG01000014.1 |
| Fusobacterium nucleatum vincentii ATCC 49256 | NZ_AABF02000001.1 - NZ_AABF02000302.1 |
| Fusobacterium periodonticum ATCC 33693 | NZ_ACJY01000001.1 - NZ_ACJY01000143.1 |
| Fusobacterium ulcerans ATCC 49185 | ACDH01000001.1 - ACDH01000123.1 |
| Fusobacterium varium ATCC 27725 | ACIE01000001.1 - ACIE01000100.1 |
| Gardnerella vaginalis 5 1 | NZ_ADAN01000001.1 - NZ_ADAN01000094.1 |
| Gardnerella vaginalis AMD | NZ_ADAM01000001.1 - NZ_ADAM01000117.1 |
| Gardnerella vaginalis ATCC 14018 | NZ_ADNB01000001.1 - NZ_ADNB01000145.1 |
| Gemella haemolysans ATCC 10379 | NZ_ACDZ02000001.1 - NZ_ACDZ02000015.1 |
| Gemella moribillum M424 | NZ_ACRX01000001.1 - NZ_ACRX01000039.1 |
| Gemmata obscuriglobus UQM 2246 | NZ_ABGO01000001.1 - NZ_ABGO01000922.1 |
| Geobacillus G11MC16 | NZ_ABVH01000001.1 - NZ_ABVH01000031.1 |
| Geobacillus thermoglucosidasius C56 YS93 | NZ_ADNQ01000001.1 - NZ_ADNQ01000097.1 |
| Glaciecola HTCC2999 | NZ_ABST01000001.1 - NZ_ABST01000056.1 |
| Gluconacetobacter hansenii ATCC 23769 | NZ_ADTV01000001.1 - NZ_ADTV01000071.1 |
| Gluconacetobacter xylinus NBRC 3288 | NZ_BABN01000001.1 - NZ_BABN01000001.1 |
| Granulicatella adiacens ATCC 49175 | NZ_ACKZ01000001.1 - NZ_ACKZ01000031.1 |
| Granulicatella elegans ATCC 700633 | ACRF01000001.1 - ACRF01000036.1 |
| Haemophilus influenzae 22 1 21 | NZ_AAZD01000001.1 - NZ_AAZD01000018.1 |
| Haemophilus influenzae 3655 | NZ_AAZF01000001.1 - NZ_AAZF01000023.1 |
| Haemophilus influenzae 6P18H1 | NZ_ABWW01000001.1 - NZ_ABWW01000028.1 |
| Haemophilus influenzae 7P49H1 | NZ_ABWV01000001.1 - NZ_ABWV01000019.1 |
| Haemophilus influenzae HK1212 | NZ_ABFC01000001.1 - NZ_ABFC01001262.1 |
| Haemophilus influenzae NT127 | NZ_ACSL01000001.1 - NZ_ACSL01000041.1 |
| Haemophilus influenzae PittAA | NZ_AAZG01000001.1 - NZ_AAZG01000040.1 |
| Haemophilus influenzae PittHH | NZ_AAZH01000001.1 - NZ_AAZH01000059.1 |
| Haemophilus influenzae PittII | NZ_AAZI01000001.1 - NZ_AAZI01000025.1 |
| Haemophilus influenzae R3021 | NZ_AAZJ01000001.1 - NZ_AAZE01000046.1 |
| Haemophilus influenzae RdAW | NZ_ACSM01000001.1 - NZ_ACSM01000032.1 |
| Haemophilus parasuis 29755 | NZ_ABKM01000001.1 - NZ_ABKM01000246.1 |
| Helicobacter bilis ATCC 43879 | ACDN01000001.1 - ACDN01000226.1 |
| Helicobacter canadensis MIT 98 5491 | ABQS01000001.1 - ACSF01000004.1 |
| Helicobacter cinaedi CCUG 18818 | ABQT01000001.1 - ABQT01000096.1 |
| Helicobacter pullorum MIT 98 5489 | ABQU01000001.1 - ABQU01000131.1 |
| Helicobacter pylori 98 10 | NZ_ABSX01000001.1 - NZ_ABSX01000051.1 |
| Helicobacter pylori B128 | NZ_ABSY01000001.1 - NZ_ABSY01000073.1 |
| Helicobacter pylori HPKX 438 AG0C1 | NZ_ABJO01000001.1 - NZ_ABJO01002602.1 |
| Helicobacter pylori HPKX 438 CA4C1 | NZ_ABJP01000001.1 - NZ_ABJP01003766.1 |
| Helicobacter winghamensis ATCC BAA 430 | ACDO01000001.1 - ACDO01000055.1 |
| Hoeflea phototrophica DFL 43 | NZ_ABIA02000001.1 - NZ_ABIA02000022.1 |
| Holdemania filiformis DSM 12042 | NZ_ACCF01000001.1 - NZ_ACCF01000289.1 |
| Hydrogenivirga 128 5 R1 1 | NZ_ABHJ01000001.1 - NZ_ABHJ01000551.1 |
| Idiomarina baltica OS145 | NZ_AAMX01000001.1 - NZ_AAMX01000070.1 |
| Janibacter HTCC2649 | NZ_AAMN01000001.1 - NZ_AAMN01000014.1 |
| Jonquetella anthropi E3 33 E1 | NZ_ACOO02000001.1 - NZ_ACOO02000037.1 |
| Kingella oralis ATCC 51147 | NZ_ACJW02000001.1 - NZ_ACJW02000012.1 |
| Klebsiella 1 1 55 | ACXA01000001.1 - ACXA01000096.1 |
| Klebsiella pneumoniae rhinoscleromatis ATCC 13884 | NZ_ACZD01000001.1 - NZ_ACZD01000268.1 |
| Kordia algicida OT 1 | NZ_ABIB01000001.1 - NZ_ABIB01000034.1 |
| Ktedonobacter racemifer DSM 44963 | NZ_ADVG01000001.1 - NZ_ADVG01000010.1 |
| Labrenzia aggregata IAM 12614 | NZ_AAUW01000001.1 - NZ_AAUW01000048.1 |
| Labrenzia alexandrii DFL 11 | ACCU01000001.1 - ACCU01000025.1 |
| Lactobacillus acidophilus ATCC 4796 | NZ_ACHN01000001.1 - NZ_ACHN01000069.1 |
| Lactobacillus amylolyticus DSM 11664 | NZ_ADNY01000001.1 - NZ_ADNY01000073.1 |
| Lactobacillus antri DSM 16041 | NZ_ACLL01000001.1 - NZ_ACLL01000076.1 |
| Lactobacillus brevis gravesensis ATCC 27305 | NZ_ACGG01000001.1 - NZ_ACGG01000178.1 |
| Lactobacillus buchneri ATCC 11577 | NZ_ACGH01000001.1 - NZ_ACGH01000165.1 |
| Lactobacillus coleohominis 101 4 CHN | ACOH01000001.1 - ACOH01000035.1 |
| Lactobacillus crispatus 125 2 CHN | ACPV01000001.1 - ACPV01000171.1 |
| Lactobacillus crispatus 214 1 | NZ_ADGR01000001.1 - NZ_ADGR01000187.1 |
| Lactobacillus crispatus CTV 05 | ADML01000001.1 - ADML01000080.1 |
| Lactobacillus crispatus JV V01 | NZ_ACKR01000001.1 - NZ_ACKR01000234.1 |
| Lactobacillus crispatus MV 1A US | ACOG01000001.1 - ACOG01000183.1 |
| Lactobacillus crispatus MV 3A US | ACQC01000001.1 - ACQC01000220.1 |
| Lactobacillus delbrueckii bulgaricus PB2003 044 T3 4 | NZ_AEAT01000001.1 - NZ_AEAT01000140.1 |
| Lactobacillus fermentum 28 3 CHN | ACQG01000001.1 - ACQG01000112.1 |
| Lactobacillus fermentum ATCC 14931 | NZ_ACGI01000001.1 - NZ_ACGI01000145.1 |
| Lactobacillus gasseri 202 4 | NZ_ACOZ01000001.1 - NZ_ACOZ01000070.1 |
| Lactobacillus gasseri 224 1 | NZ_ADFT01000001.1 - NZ_ADFT01000037.1 |
| Lactobacillus gasseri JV V03 | NZ_ACGO02000001.1 - NZ_ACGO02000010.1 |
| Lactobacillus gasseri MV 22 | ABWH02000001.1 - ABWH02000008.1 |
| Lactobacillus helveticus DSM 20075 | NZ_ACLM01000001.1 - NZ_ACLM01000235.1 |
| Lactobacillus hilgardii ATCC 8290 | NZ_ACGP01000001.1 - NZ_ACGP01000233.1 |
| Lactobacillus iners AB 1 | NZ_ADHG01000001.1 - NZ_ADHG01000007.1 |
| Lactobacillus iners ATCC 55195 | NZ_AEPX01000001.1 - NZ_AEPX01000019.1 |
| Lactobacillus iners DSM 13335 | NZ_ACLN01000001.1 - NZ_ACLN01000022.1 |
| Lactobacillus iners LEAF 2052A d | NZ_AEKI01000001.1 - NZ_AEKI01000028.1 |
| Lactobacillus iners LEAF 2053A b | NZ_AEKH01000001.1 - NZ_AEKH01000037.1 |
| Lactobacillus iners LEAF 2062A h1 | NZ_AEKJ01000001.1 - NZ_AEKJ01000024.1 |
| Lactobacillus iners LEAF 3008A a | NZ_AEKK01000001.1 - NZ_AEKK01000025.1 |
| Lactobacillus iners LactinV 01V1 a | NZ_AEHQ01000001.1 - NZ_AEHQ01000092.1 |
| Lactobacillus iners LactinV 03V1 b | NZ_AEHP01000001.1 - NZ_AEHP01000067.1 |
| Lactobacillus iners LactinV 09V1 c | NZ_AEHO01000001.1 - NZ_AEHO01000035.1 |
| Lactobacillus iners LactinV 11V1 d | NZ_AEHN01000001.1 - NZ_AEHN01000027.1 |
| Lactobacillus iners SPIN 2503V10 D | NZ_AEHR01000001.1 - NZ_AEHR01000031.1 |
| Lactobacillus jensenii 1153 | NZ_ABWG02000001.1 - NZ_ABWG02000009.1 |
| Lactobacillus jensenii 115 3 CHN | ACQN01000001.1 - ACQN01000058.1 |
| Lactobacillus jensenii 208 1 | NZ_ADEX01000001.1 - NZ_ADEX01000216.1 |
| Lactobacillus jensenii 269 3 | NZ_ACOY01000001.1 - NZ_ACOY01000057.1 |
| Lactobacillus jensenii 27 2 CHN | ACOF01000001.1 - ACOF01000056.1 |
| Lactobacillus jensenii JV V16 | NZ_ACGQ02000001.1 - NZ_ACGQ02000002.1 |
| Lactobacillus jensenii SJ 7A US | ACQD01000001.1 - ACQD01000075.1 |
| Lactobacillus johnsonii ATCC 33200 | NZ_ACGR01000001.1 - NZ_ACGR01000052.1 |
| Lactobacillus oris PB013 T2 3 | NZ_AEKL01000001.1 - NZ_AEKL01000089.1 |
| Lactobacillus paracasei 8700 2 | ABQV01000001.1 - ABQV01000090.1 |
| Lactobacillus paracasei ATCC 25302 | NZ_ACGY01000001.1 - NZ_ACGY01000174.1 |
| Lactobacillus plantarum ATCC 14917 | NZ_ACGZ02000001.1 - NZ_ACGZ02000036.1 |
| Lactobacillus reuteri 100 23 | NZ_AAPZ02000001.1 - NZ_AAPZ02000002.1 |
| Lactobacillus reuteri CF48 3A | NZ_ACHG01000001.1 - NZ_ACHG01000244.1 |
| Lactobacillus reuteri MM2 3 | NZ_ACLB01000001.1 - NZ_ACLB01000167.1 |
| Lactobacillus reuteri MM4 1A | NZ_ACGX01000001.1 - NZ_ACGX01000131.1 |
| Lactobacillus reuteri SD2112 | NZ_ACGW02000001.1 - NZ_ACGW02000014.1 |
| Lactobacillus rhamnosus HN001 | NZ_ABWJ01000001.1 - NZ_ABWJ01000094.1 |
| Lactobacillus rhamnosus LMS2 1 | NZ_ACIZ01000001.1 - NZ_ACIZ01000162.1 |
| Lactobacillus ruminis ATCC 25644 | NZ_ACGS01000001.1 - NZ_ACGS01000169.1 |
| Lactobacillus salivarius ACS 116 V Col5a | NZ_AEBA01000001.1 - NZ_AEBA01000154.1 |
| Lactobacillus salivarius ATCC 11741 | NZ_ACGT01000001.1 - NZ_ACGT01000054.1 |
| Lactobacillus ultunensis DSM 16047 | NZ_ACGU01000001.1 - NZ_ACGU01000116.1 |
| Lactobacillus vaginalis ATCC 49540 | NZ_ACGV01000001.1 - NZ_ACGV01000202.1 |
| Lautropia mirabilis ATCC 51599 | NZ_AEQP01000001.1 - NZ_AEQP01000030.1 |
| Leeuwenhoekiella blandensis MED217 | NZ_AANC01000001.1 - NZ_AANC01000015.1 |
| Legionella drancourtii LLAP12 | NZ_ACUL01000001.1 - NZ_ACUL01000263.1 |
| Legionella longbeachae D 4968 | NZ_ACZG01000001.1 - NZ_ACZG01000013.1 |
| Lentisphaera araneosa HTCC2155 | NZ_ABCK01000001.1 - NZ_ABCK01000081.1 |
| Leptolyngbya valderiana BDU 20041 | NZ_AAZV01000001.1 - NZ_AAZV01000096.1 |
| Leptotrichia goodfellowii F0264 | NZ_ADAD01000001.1 - NZ_ADAD01000208.1 |
| Leptotrichia hofstadii F0254 | NZ_ACVB02000001.1 - NZ_ACVB02000042.1 |
| Leuconostoc mesenteroides cremoris ATCC 19254 | NZ_ACKV01000001.1 - NZ_ACKV01000126.1 |
| Limnobacter MED105 | NZ_ABCT01000001.1 - NZ_ABCT01000046.1 |
| Listeria grayi DSM 20601 | NZ_ACCR02000001.1 - NZ_ACCR02000006.1 |
| Listeria ivanovii FSL F6 596 | NZ_ADXI01000001.1 - NZ_ADXI01001919.1 |
| Listeria marthii FSL S4 120 | NZ_ADXF01000001.1 - NZ_ADXF01001378.1 |
| Listeria monocytogenes 10403S | NZ_AARZ02000001.1 - NZ_AARZ02000021.1 |
| Listeria monocytogenes F6900 | NZ_AARU02000001.1 - NZ_AARU02000023.1 |
| Listeria monocytogenes FSL F2 515 | NZ_AARI02000001.1 - NZ_AARI02001728.1 |
| Listeria monocytogenes FSL J1 175 | NZ_AARK02000001.1 - NZ_AARK02000457.1 |
| Listeria monocytogenes FSL J1 194 | NZ_AARJ02000001.1 - NZ_AARJ02000030.1 |
| Listeria monocytogenes FSL J1 208 | NZ_AARL02000001.1 - NZ_AARL02001660.1 |
| Listeria monocytogenes FSL J2 003 | NZ_AARM02000001.1 - NZ_AARM02000795.1 |
| Listeria monocytogenes FSL J2 064 | NZ_AARO02000001.1 - NZ_AARO02000545.1 |
| Listeria monocytogenes FSL J2 071 | NZ_AARN04000001.1 - NZ_AARN04000053.1 |
| Listeria monocytogenes FSL N1 017 | NZ_AARP04000001.1 - NZ_AARP04000079.1 |
| Listeria monocytogenes FSL N3 165 | NZ_AARQ02000001.1 - NZ_AARQ02000039.1 |
| Listeria monocytogenes FSL R2 503 | NZ_AARR02000001.1 - NZ_AARR02000055.1 |
| Listeria monocytogenes FSL R2 561 | NZ_AARS01000001.1 - NZ_AARS01000037.1 |
| Listeria monocytogenes Finland 1988 | NZ_AART01000001.1 - NZ_AART01000049.1 |
| Listeria monocytogenes HPB2262 | NZ_AATL02000001.1 - NZ_AATL02000079.1 |
| Listeria monocytogenes J0161 | NZ_AARW02000001.1 - NZ_AARW02000025.1 |
| Listeria monocytogenes J2818 | NZ_AARX02000001.1 - NZ_AARX02000024.1 |
| Listeria monocytogenes LO28 | NZ_AARY02000001.1 - NZ_AARY02001150.1 |
| Listeria monocytogenes serotype 1 2a F6854 | NZ_AADQ01000001.1 - NZ_AADQ01000133.1 |
| Listeria monocytogenes serotype 4b H7858 | NZ_AADR01000001.1 - NZ_AADR01000181.1 |
| Loktanella vestfoldensis SKA53 | NZ_AAMS01000001.1 - NZ_AAMS01000014.1 |
| Lutiella nitroferrum 2002 | NZ_ACIS01000001.1 - NZ_ACIS01000020.1 |
| Lyngbya PCC 8106 | NZ_AAVU01000001.1 - NZ_AAVU01000110.1 |
| Lysinibacillus fusiformis ZC1 | NZ_ADJR01000001.1 - NZ_ADJR01000113.1 |
| Magnetospirillum magnetotacticum MS 1 | NZ_AAAP01000001.2 - NZ_AAAP01003880.1 |
| Mannheimia haemolytica PHL213 | AASA01000001.1 - AASA01000152.1 |
| Mannheimia haemolytica serotype A2 BOVINE | NZ_ACZY01000001.1 - NZ_ACZY01000084.1 |
| Mannheimia haemolytica serotype A2 OVINE | NZ_ACZX01000001.1 - NZ_ACZX01000144.1 |
| Marinobacter ELB17 | NZ_AAXY01000001.1 - NZ_AAXY01000075.1 |
| Marinobacter algicola DG893 | NZ_ABCP01000001.1 - NZ_ABCP01000104.1 |
| Marinomonas MED121 | NZ_AANE01000001.1 - NZ_AANE01000047.1 |
| Mariprofundus ferrooxydans PV 1 | NZ_AATS01000001.1 - NZ_AATS01000032.1 |
| Maritimibacter alkaliphilus HTCC2654 | NZ_AAMT01000001.1 - NZ_AAMT01000046.1 |
| Megasphaera micronuciformis F0359 | NZ_AECS01000001.1 - NZ_AECS01000049.1 |
| Mesorhizobium opportunistum WSM2075 | NZ_ACZA01000001.1 - NZ_ACZA01000089.1 |
| Methanobrevibacter smithii DSM 2374 | NZ_ABYV02000001.1 - NZ_ABYV02000025.1 |
| Methanobrevibacter smithii DSM 2375 | NZ_ABYW01000001.1 - NZ_ABYW01000024.1 |
| Methanothermococcus okinawensis IH1 | NZ_AEDA01000001.1 - NZ_AEDA01000057.1 |
| Methylobacter tundripaludum SV96 | NZ_AEGW01000001.1 - NZ_AEGW01000102.1 |
| Methylosinus trichosporium OB3b | NZ_ADVE01000001.1 - NZ_ADVE01000173.1 |
| Micrococcus luteus NCTC 2665 | NZ_CABC01000001.1 - NZ_CABC01000134.1 |
| Micrococcus luteus SK58 | NZ_ADCD01000001.1 - NZ_ADCD01000112.1 |
| Microcoleus chthonoplastes PCC 7420 | ABRS01000001.1 - ABRS01000142.1 |
| Micromonospora ATCC 39149 | ACES01000001.1 - ACES01000339.1 |
| Microscilla marina ATCC 23134 | NZ_AAWS01000001.1 - NZ_AAWS01000135.1 |
| Mitsuokella multacida DSM 20544 | NZ_ABWK02000001.1 - NZ_ABWK02000031.1 |
| Mobiluncus curtisii ATCC 35241 | NZ_AEEE01000001.1 - NZ_AEEE01000021.1 |
| Mobiluncus curtisii ATCC 51333 | NZ_AEPY01000001.1 - NZ_AEPY01000016.1 |
| Mobiluncus curtisii holmesii ATCC 35242 | NZ_AEPZ01000001.1 - NZ_AEPZ01000016.1 |
| Mobiluncus mulieris 28 1 | NZ_ADBR01000001.1 - NZ_ADBR01000055.1 |
| Mobiluncus mulieris ATCC 35239 | NZ_AEET01000001.1 - NZ_AEET01000066.1 |
| Mobiluncus mulieris ATCC 35243 | NZ_ACKW01000001.1 - NZ_ACKW01000065.1 |
| Mobiluncus mulieris FB024 16 | NZ_AEGV01000001.1 - NZ_AEGV01000046.1 |
| Moritella PE36 | NZ_ABCQ01000001.1 - NZ_ABCQ01000131.1 |
| Mucilaginibacter paludis DSM 18603 | NZ_AEIH01000001.1 - NZ_AEIH01000183.1 |
| Mycobacterium avium ATCC 25291 | NZ_ACFI01000001.1 - NZ_ACFI01000258.1 |
| Mycobacterium intracellulare ATCC 13950 | NZ_ABIN01000001.1 - NZ_ABIN01000353.1 |
| Mycobacterium kansasii ATCC 12478 | NZ_ACBV01000001.1 - NZ_ACBV01000299.1 |
| Mycobacterium parascrofulaceum ATCC BAA 614 | NZ_ADNV01000001.1 - NZ_ADNV01000405.1 |
| Mycobacterium tuberculosis 02 1987 | ABLM01000001.1 - ABLM01000215.1 |
| Mycobacterium tuberculosis 210 | NZ_ADAB01000001.1 - NZ_ADAB01000130.1 |
| Mycobacterium tuberculosis 94 M4241A | ABLL01000001.1 - ABLL01000192.1 |
| Mycobacterium tuberculosis C | AAKR01000001.1 - AAKR01000160.1 |
| Mycobacterium tuberculosis CPHL A | ACHP01000001.1 - ACHP01000077.1 |
| Mycobacterium tuberculosis EAS054 | ABOV01000001.1 - ABOV01000125.1 |
| Mycobacterium tuberculosis GM 1503 | ABQG01000001.1 - ABQG01000295.1 |
| Mycobacterium tuberculosis H37Ra | NZ_AAYK01000001.1 - NZ_AAYK01000272.1 |
| Pediococcus acidilactici 7 4 | ACXB01000001.1 - ACXB01000028.1 |
| Pedobacter BAL39 | NZ_ABCM01000001.1 - NZ_ABCM01000047.1 |
| Pedobacter saltans DSM 12145 | NZ_AEKX01000001.1 - NZ_AEKX01000023.1 |
| Pelagibaca bermudensis HTCC2601 | NZ_AATQ01000001.1 - NZ_AATQ01000103.1 |
| Peptoniphilus duerdenii ATCC BAA 1640 | NZ_AEEH01000001.1 - NZ_AEEH01000061.1 |
| Peptoniphilus harei ACS 146 V Sch2b | NZ_AENP01000001.1 - NZ_AENP01000032.1 |
| Peptoniphilus lacrimalis 315 B | NZ_ADDO01000001.1 - NZ_ADDO01000071.1 |
| Peptoniphilus oral taxon 386 F0131 | ADCS01000001.1 - ADCS01000072.1 |
| Peptoniphilus oral taxon 836 F0141 | NZ_AEAA01000001.1 - NZ_AEAA01000145.1 |
| Peptostreptococcus anaerobius 653 L | NZ_ADJN01000001.1 - NZ_ADJN01000073.1 |
| Peptostreptococcus stomatis DSM 17678 | NZ_ADGQ01000001.1 - NZ_ADGQ01000074.1 |
| Phaeobacter gallaeciensis 2 10 | NZ_ABIE01000001.1 - NZ_ABIE01000033.1 |
| Phaeobacter gallaeciensis BS107 | NZ_ABIF01000001.1 - NZ_ABIF01000024.1 |
| Photobacterium SKA34 | NZ_AAOU01000001.1 - NZ_AAOU01000088.1 |
| Photobacterium angustum S14 | NZ_AAOJ01000001.1 - NZ_AAOJ01000045.1 |
| Photobacterium damselae CIP 102761 | NZ_ADBS01000001.1 - NZ_ADBS01000008.1 |
| Photobacterium profundum 3TCK | NZ_AAPH01000001.1 - NZ_AAPH01000082.1 |
| Planctomyces brasiliensis DSM 5305 | NZ_AEIC01000001.1 - NZ_AEIC01000061.1 |
| Planctomyces maris DSM 8797 | NZ_ABCE01000001.1 - NZ_ABCE01000125.1 |
| Plesiocystis pacifica SIR 1 | NZ_ABCS01000001.1 - NZ_ABCS01000237.1 |
| Polaribacter MED152 | AANA01000001.1 - AANA01000002.1 |
| Polaribacter irgensii 23 P | NZ_AAOG01000001.1 - NZ_AAOG01000007.1 |
| Porphyromonas asaccharolytica PR426713P I | NZ_AENO01000001.1 - NZ_AENO01000058.1 |
| Porphyromonas endodontalis ATCC 35406 | NZ_ACNN01000001.1 - NZ_ACNN01000037.1 |
| Porphyromonas uenonis 60 3 | NZ_ACLR01000001.1 - NZ_ACLR01000250.1 |
| Prevotella amnii CRIS 21A A | NZ_ADFQ01000001.1 - NZ_ADFQ01000117.1 |
| Prevotella bergensis DSM 17361 | NZ_ACKS01000001.1 - NZ_ACKS01000112.1 |
| Prevotella bivia JCVIHMP010 | NZ_ADFO01000001.1 - NZ_ADFO01000121.1 |
| Prevotella bryantii B14 | NZ_ADWO01000001.1 - NZ_ADWO01000098.1 |
| Prevotella buccae ATCC 33574 | NZ_AEPD01000001.1 - NZ_AEPD01000069.1 |
| Prevotella buccae D17 | ACRB01000001.1 - ACRB01000264.1 |
| Prevotella buccalis ATCC 35310 | NZ_ADEG01000001.1 - NZ_ADEG01000118.1 |
| Prevotella copri DSM 18205 | NZ_ACBX02000001.1 - NZ_ACBX02000073.1 |
| Prevotella disiens FB035 09AN | NZ_AEDO01000001.1 - NZ_AEDO01000078.1 |
| Prevotella marshii DSM 16973 | NZ_AEEI01000001.1 - NZ_AEEI01000079.1 |
| Prevotella melaninogenica D18 | ACWY01000001.1 - ACWY01000173.1 |
| Prevotella oral taxon 299 F0039 | ACWZ01000001.1 - ACWZ01000032.1 |
| Prevotella oral taxon 317 F0108 | ACQH01000001.1 - ACQH01000205.1 |
| Prevotella oral taxon 472 F0295 | NZ_ACZS01000001.1 - NZ_ACZS01000190.1 |
| Prevotella oralis ATCC 33269 | NZ_AEPE01000001.1 - NZ_AEPE01000027.1 |
| Prevotella oris C735 | ADDV01000001.1 - ADDV01000127.1 |
| Prevotella oris F0302 | NZ_ACUZ02000001.1 - NZ_ACUZ02000087.1 |
| Prevotella salivae DSM 15606 | NZ_AEQO01000001.1 - NZ_AEQO01000258.1 |
| Prevotella tannerae ATCC 51259 | NZ_ACIJ02000001.1 - NZ_ACIJ02000034.1 |
| Prevotella timonensis CRIS 5C B1 | NZ_ADEF01000001.1 - NZ_ADEF01000072.1 |
| Prevotella veroralis F0319 | NZ_ACVA01000001.1 - NZ_ACVA01000098.1 |
| Prochlorococcus marinus MIT 9202 | ACDW01000001.1 - ACDW01000006.1 |
| Propionibacterium acnes J139 | NZ_ADFS01000001.1 - NZ_ADFS01000007.1 |
| Propionibacterium acnes J165 | NZ_ADJL01000001.1 - NZ_ADJL01000062.1 |
| Propionibacterium acnes SK187 | NZ_ADJM01000001.1 - NZ_ADJM01000037.1 |
| Proteus mirabilis ATCC 29906 | NZ_ACLE01000001.1 - NZ_ACLE01000115.1 |
| Proteus penneri ATCC 35198 | NZ_ABVP01000001.1 - NZ_ABVP01000026.1 |
| Providencia alcalifaciens DSM 30120 | NZ_ABXW01000001.1 - NZ_ABXW01000079.1 |
| Providencia rettgeri DSM 1131 | NZ_ACCI02000001.1 - NZ_ACCI02000177.1 |
| Providencia rustigianii DSM 4541 | NZ_ABXV02000001.1 - NZ_ABXV02000163.1 |
| Providencia stuartii ATCC 25827 | NZ_ABJD02000001.1 - NZ_ABJD02000120.1 |
| Pseudoalteromonas tunicata D2 | NZ_AAOH01000001.1 - NZ_AAOH01000042.1 |
| Pseudomonas UK4 | NZ_ACOQ01000001.1 - NZ_ACOQ01002971.1 |
| Pseudomonas aeruginosa 2192 | AAKW01000001.1 - AAKW01000082.1 |
| Pseudomonas aeruginosa 39016 | NZ_AEEX01000001.1 - NZ_AEEX01000349.1 |
| Pseudomonas aeruginosa C3719 | AAKV01000001.1 - AAKV01000124.1 |
| Pseudomonas aeruginosa PACS2 | NZ_AAQW01000001.1 - NZ_AAQW01000001.1 |
| Pseudomonas aeruginosa PAb1 | NZ_ABKZ01000001.1 - NZ_ABKZ01000658.1 |
| Pseudomonas fluorescens WH6 | NZ_AEAZ01000001.1 - NZ_AEAZ01000053.1 |
| Pseudomonas savastanoi NCPPB 3335 | ADMI01000001.1 - ADMI01000403.1 |
| Pseudomonas syringae 642 | NZ_ADGB01000001.1 - NZ_ADGB01000296.1 |
| Pseudomonas syringae FF5 | NZ_ACXZ01000001.1 - NZ_ACXZ01004578.1 |
| Pseudomonas syringae aesculi 2250 | NZ_ACXT01000001.1 - NZ_ACXT01000776.1 |
| Pseudomonas syringae aesculi NCPPB3681 | NZ_ACXS01000001.1 - NZ_ACXS01000841.1 |
| Pseudomonas syringae oryzae 1 6 | NZ_ABZR01000001.1 - NZ_ABZR01002855.1 |
| Pseudomonas syringae tabaci ATCC 11528 | NZ_ACHU02000001.1 - NZ_ACHU02001405.1 |
| Pseudomonas syringae tomato K40 | NZ_ADFY01000001.1 - NZ_ADFY01000582.1 |
| Pseudomonas syringae tomato Max13 | NZ_ADFZ01000001.1 - NZ_ADFZ01000349.1 |
| Pseudomonas syringae tomato NCPPB 1108 | NZ_ADGA01000001.1 - NZ_ADGA01000304.1 |
| Pseudomonas syringae tomato T1 | NZ_ABSM01000001.1 - NZ_ABSM01000122.1 |
| Pseudoramibacter alactolyticus ATCC 23263 | NZ_AEQN01000001.1 - NZ_AEQN01000075.1 |
| Pseudovibrio JE062 | ABXL01000001.1 - ABXL01000053.1 |
| Psychroflexus torquis ATCC 700755 | NZ_AAPR01000001.1 - NZ_AAPR01000508.1 |
| Psychromonas CNPT3 | NZ_AAPG01000001.1 - NZ_AAPG01000175.1 |
| Pyramidobacter piscolens W5455 | NZ_ADFP01000001.1 - NZ_ADFP01000140.1 |
| Ralstonia 5 7 47FAA | NZ_ACUF01000001.1 - NZ_ACUF01000093.1 |
| Ralstonia solanacearum UW551 | NZ_AAKL01000001.1 - NZ_AAKL01000577.1 |
| Raphidiopsis brookii D9 | NZ_ACYB01000001.1 - NZ_ACYB01000047.1 |
| Reinekea blandensis MED297 | NZ_AAOE01000001.1 - NZ_AAOE01000061.1 |
| Rhizobium etli 8C 3 | NZ_ABRA01000001.1 - NZ_ABRA01002725.1 |
| Rhizobium etli Brasil 5 | NZ_ABQZ01000001.1 - NZ_ABQZ01002724.1 |
| Rhizobium etli CIAT 894 | NZ_ABRD01000001.1 - NZ_ABRD01002844.1 |
| Rhizobium etli GR56 | NZ_ABRB01000001.1 - NZ_ABRB01002309.1 |
| Rhizobium etli IE4771 | NZ_ABRC01000001.1 - NZ_ABRC01002886.1 |
| Rhizobium etli Kim 5 | NZ_ABQY01000001.1 - NZ_ABQY01002745.1 |
| Rhodobacter SW2 | NZ_ACYY01000001.1 - NZ_ACYY01000059.1 |
| Rhodococcus erythropolis SK121 | NZ_ACNO01000001.1 - NZ_ACNO01000124.1 |
| Rickettsia endosymbiont of Ixodes scapularis | ACLC01000001.1 - ACLC01000135.1 |
| Rickettsia sibirica 246 | NZ_AABW01000001.1 - NZ_AABW01000001.1 |
| Rickettsiella grylli | NZ_AAQJ02000001.1 - NZ_AAQJ02000002.1 |
| Roseburia intestinalis L1 82 | NZ_ABYJ02000001.1 - NZ_ABYJ02000409.1 |
| Roseburia inulinivorans DSM 16841 | NZ_ACFY01000001.1 - NZ_ACFY01000179.1 |
| Roseibium TrichSKD4 | AEFL01000001.1 - AEFL01000108.1 |
| Roseobacter litoralis Och 149 | NZ_ABIG01000001.1 - NZ_ABIG01000027.1 |
| Roseomonas cervicalis ATCC 49957 | NZ_ADVL01000001.1 - NZ_ADVL01001015.1 |
| Roseovarius 217 | NZ_AAMV01000001.1 - NZ_AAMV01000037.1 |
| Roseovarius TM1035 | NZ_ABCL01000001.1 - NZ_ABCL01000015.1 |
| Roseovarius nubinhibens ISM | NZ_AALY01000001.1 - NZ_AALY01000010.1 |
| Rothia dentocariosa M567 | ADDW01000001.1 - ADDW01000026.1 |
| Rothia mucilaginosa ATCC 25296 | NZ_ACVO01000001.1 - NZ_ACVO01000025.1 |
| Ruegeria R11 | ABXM01000001.1 - ABXM01000017.1 |
| Ruegeria Silicibacter TrichCH4B | ACNZ01000001.1 - ACNZ01000129.1 |
| Ruegeria Silicibacter lacuscaerulensis ITI 1157 | ACNX01000001.1 - ACNX01000047.1 |
| Ruminococcus 5 1 39BFAA | ACII01000001.1 - ACII01000200.1 |
| Ruminococcus albus 8 | NZ_ADKM01000001.1 - NZ_ADKM01000245.1 |
| Ruminococcus flavefaciens FD 1 | NZ_ACOK01000001.1 - NZ_ACOK01000119.1 |
| Ruminococcus gnavus ATCC 29149 | NZ_AAYG02000001.1 - NZ_AAYG02000043.1 |
| Ruminococcus lactaris ATCC 29176 | NZ_ABOU02000001.1 - NZ_ABOU02000062.1 |
| Ruminococcus obeum ATCC 29174 | NZ_AAVO02000001.1 - NZ_AAVO02000062.1 |
| Ruminococcus torques ATCC 27756 | NZ_AAVP02000001.1 - NZ_AAVP02000064.1 |
| Saccharopolyspora erythraea NRRL 2338 | NZ_ABFV01000001.1 - NZ_ABFV01000241.1 |
| Sagittula stellata E 37 | NZ_AAYA01000001.1 - NZ_AAYA01000039.1 |
| Salmonella enterica serovar 4 5 12 i CVM23701 | NZ_ABAO01000001.3 - NZ_ABAO01000113.1 |
| Salmonella enterica serovar Hadar RI 05P066 | NZ_ABFG01000001.1 - NZ_ABFG01000050.1 |
| Salmonella enterica serovar Heidelberg SL486 | NZ_ABEL01000001.1 - NZ_ABEL01000048.1 |
| Salmonella enterica serovar Javiana GA MM04042433 | NZ_ABEH02000001.1 - NZ_ABEH02000019.1 |
| Salmonella enterica serovar Kentucky CDC 191 | NZ_ABEI01000001.1 - NZ_ABEI01000053.1 |
| Salmonella enterica serovar Kentucky CVM29188 | NZ_ABAK02000001.1 - NZ_ABAK02000001.1 |
| Salmonella enterica serovar Newport SL317 | NZ_ABEW01000001.1 - NZ_ABEW01000063.1 |
| Salmonella enterica serovar Saintpaul SARA23 | NZ_ABAM02000001.1 - NZ_ABAM02000002.1 |
| Salmonella enterica serovar Saintpaul SARA29 | NZ_ABAN01000001.3 - NZ_ABAN01000182.1 |
| Salmonella enterica serovar Schwarzengrund SL480 | NZ_ABEJ01000001.1 - NZ_ABEJ01000067.1 |
| Salmonella enterica serovar Tennessee CDC07 0191 | NZ_ACBF01000001.1 - NZ_ACBF01000094.1 |
| Salmonella enterica serovar Typhi 404ty | NZ_CAAQ01000001.1 - NZ_CAAQ01006441.1 |
| Salmonella enterica serovar Typhi AG3 | NZ_CAAY01000001.1 - NZ_CAAY01007336.1 |
| Salmonella enterica serovar Typhi E00 7866 | NZ_CAAR01000001.1 - NZ_CAAR01001445.1 |
| Salmonella enterica serovar Typhi E01 6750 | NZ_CAAS01000001.1 - NZ_CAAS01004564.1 |
| Salmonella enterica serovar Typhi E02 1180 | NZ_CAAT01000001.1 - NZ_CAAT01000422.1 |
| Salmonella enterica serovar Typhi E98 0664 | NZ_CAAU01000001.1 - NZ_CAAU01003939.1 |
| Salmonella enterica serovar Typhi E98 2068 | NZ_CAAV01000001.1 - NZ_CAAV01003682.1 |
| Salmonella enterica serovar Typhi E98 3139 | NZ_CAAZ01000001.1 - NZ_CAAZ01000415.1 |
| Salmonella enterica serovar Typhi J185 | NZ_CAAW01000001.1 - NZ_CAAW01001065.1 |
| Salmonella enterica serovar Typhi M223 | NZ_CAAX01000001.1 - NZ_CAAX01003024.1 |
| Salmonella enterica serovar Virchow SL491 | NZ_ABFH02000001.1 - NZ_ABFH02000003.1 |
| Salmonella enterica serovar Weltevreden HI N05 537 | NZ_ABFF01000001.1 - NZ_ABFF01000081.1 |
| Scardovia inopinata F0304 | ADCX01000001.1 - ADCX01000020.1 |
| Segniliparus rugosus ATCC BAA 974 | NZ_ACZI01000001.1 - NZ_ACZI01000262.1 |
| Selenomonas artemidis F0399 | NZ_AECV01000001.1 - NZ_AECV01000066.1 |
| Selenomonas flueggei ATCC 43531 | NZ_ACLA01000001.1 - NZ_ACLA01000033.1 |
| Selenomonas noxia ATCC 43541 | NZ_ACKT01000001.1 - NZ_ACKT01000056.1 |
| Selenomonas oral taxon 137 F0430 | NZ_AENV01000001.1 - NZ_AENV01000015.1 |
| Selenomonas oral taxon 149 67H29BP | NZ_AEEJ01000001.1 - NZ_AEEJ01000056.1 |
| Selenomonas sputigena ATCC 35185 | NZ_ACKP02000001.1 - NZ_ACKP02000061.1 |
| Serratia odorifera 4Rx13 | NZ_ADBX01000001.1 - NZ_ADBX01000017.1 |
| Serratia odorifera DSM 4582 | NZ_ADBY01000001.1 - NZ_ADBY01000091.1 |
| Serratia symbiotica Tucson | AENX01000001.1 - AENX01000601.1 |
| Shewanella baltica BA175 | NZ_AEDE01000001.1 - NZ_AEDE01000072.1 |
| Shewanella baltica OS183 | NZ_AECY01000001.1 - NZ_AECY01000050.1 |
| Shewanella benthica KT99 | NZ_ABIC01000001.1 - NZ_ABIC01000129.1 |
| Shigella D9 | NZ_ACDL01000001.1 - NZ_ACDL01000098.1 |
| Shigella dysenteriae 1012 | NZ_AAMJ02000001.1 - NZ_AAMJ02000189.1 |
| Shigella dysenteriae 1617 | NZ_ADUT01000001.1 - NZ_ADUT01000067.1 |
| Shuttleworthia satelles DSM 14600 | NZ_ACIP02000001.1 - NZ_ACIP02000010.1 |
| Simonsiella muelleri ATCC 29453 | ADCY01000001.1 - ADCY01000167.1 |
| Sinorhizobium meliloti AK83 | NZ_AEDH01000001.1 - NZ_AEDH01000233.1 |
| Sinorhizobium meliloti BL225C | NZ_AEDG01000001.1 - NZ_AEDG01000158.1 |
| Slackia exigua ATCC 700122 | NZ_ACUX02000001.1 - NZ_ACUX02000019.1 |
| Solobacterium moorei F0204 | NZ_AECQ01000001.1 - NZ_AECQ01000066.1 |
| Sphingobacterium spiritivorum ATCC 33300 | NZ_ACHB01000001.1 - NZ_ACHB01000122.1 |
| Sphingobacterium spiritivorum ATCC 33861 | NZ_ACHA02000001.1 - NZ_ACHA02000015.1 |
| Sphingobium chlorophenolicum L 1 | NZ_AEDK01000001.1 - NZ_AEDK01000022.1 |
| Sphingomonas SKA58 | NZ_AAQG01000001.1 - NZ_AAQG01000041.1 |
| Staphylococcus aureus 132 | NZ_ACOT01000001.1 - NZ_ACOT01000046.1 |
| Staphylococcus aureus 55 2053 | ACJR01000001.1 - ACJR01000094.1 |
| Staphylococcus aureus 58 424 | ACUT01000001.1 - ACUT01000079.1 |
| Staphylococcus aureus 65 1322 | ACJS01000001.1 - ACJS01000070.1 |
| Staphylococcus aureus 68 397 | ACJT01000001.1 - ACJT01000071.1 |
| Staphylococcus aureus 930918 3 | NZ_ABFA01000001.1 - NZ_ABFA01000736.1 |
| Staphylococcus aureus A017934 97 | ACYP01000001.1 - ACYP01000055.1 |
| Staphylococcus aureus A10102 | NZ_ACSO01000001.1 - NZ_ACSO01000045.1 |
| Staphylococcus aureus A5937 | NZ_ACKC01000001.1 - NZ_ACKC01000032.1 |
| Staphylococcus aureus A5948 | NZ_ACKD01000001.1 - NZ_ACKD01000065.1 |
| Staphylococcus aureus A6224 | NZ_ACKE01000001.1 - NZ_ACKE01000049.1 |
| Staphylococcus aureus A6300 | NZ_ACKF01000001.1 - NZ_ACKF01000049.1 |
| Staphylococcus aureus A8115 | NZ_ACKG01000001.1 - NZ_ACKG01000038.1 |
| Staphylococcus aureus A8117 | NZ_ACYO01000001.1 - NZ_ACYO01000032.1 |
| Staphylococcus aureus A8796 | NZ_ADJJ01000001.1 - NZ_ADJJ01000098.1 |
| Staphylococcus aureus A8819 | NZ_ADJK01000001.1 - NZ_ADJK01000048.1 |
| Staphylococcus aureus A9299 | NZ_ACKH01000001.1 - NZ_ACKH01000048.1 |
| Staphylococcus aureus A9635 | NZ_ACKI01000001.1 - NZ_ACKI01000040.1 |
| Staphylococcus aureus A9719 | NZ_ACKJ01000001.1 - NZ_ACKJ01000060.1 |
| Staphylococcus aureus A9754 | NZ_ADJI01000001.1 - NZ_ADJI01000059.1 |
| Staphylococcus aureus A9763 | NZ_ACKK01000001.1 - NZ_ACKK01000041.1 |
| Staphylococcus aureus A9765 | NZ_ACSN01000001.1 - NZ_ACSN01000086.1 |
| Staphylococcus aureus A9781 | NZ_ACKL01000001.1 - NZ_ACKL01000050.1 |
| Staphylococcus aureus ATCC 51811 | NZ_ADVP01000001.1 - NZ_ADVP01000047.1 |
| Staphylococcus aureus ATCC BAA 39 | NZ_AEEK01000001.1 - NZ_AEEK01000083.1 |
| Staphylococcus aureus Btn1260 | ACUU01000001.1 - ACUU01000051.1 |
| Staphylococcus aureus C101 | ACSP01000001.1 - ACSP01000053.1 |
| Staphylococcus aureus C160 | ACUV01000001.1 - ACUV01000063.1 |
| Staphylococcus aureus C427 | ACSQ01000001.1 - ACSQ01000054.1 |
| Staphylococcus aureus CF Marseille | NZ_CABA01000001.1 - NZ_CABA01000131.1 |
| Staphylococcus aureus D139 | ACSR01000001.1 - ACSR01000097.1 |
| Staphylococcus aureus D30 | NZ_ABFB01000001.1 - NZ_ABFB01000463.1 |
| Staphylococcus aureus E1410 | ACJU01000001.1 - ACJU01000056.1 |
| Staphylococcus aureus EMRSA16 | ADAT01000001.1 - ADAT01000070.1 |
| Staphylococcus aureus H19 | ACSS01000001.1 - ACSS01000063.1 |
| Staphylococcus aureus JKD6009 | NZ_ABSA01000001.1 - NZ_ABSA01000129.1 |
| Staphylococcus aureus M1015 | ACST01000001.1 - ACST01000048.1 |
| Staphylococcus aureus M809 | ACUS01000001.1 - ACUS01000085.1 |
| Staphylococcus aureus M876 | ACJV01000001.1 - ACJV01000052.1 |
| Staphylococcus aureus M899 | ACSU01000001.1 - ACSU01000045.1 |
| Staphylococcus aureus MN8 | NZ_ACJA02000001.1 - NZ_ACJA02000005.1 |
| Staphylococcus aureus MR1 | NZ_ACZQ01000001.1 - NZ_ACZQ01000339.1 |
| Staphylococcus aureus Mu50 omega | NZ_BABM01000001.1 - NZ_BABM01000001.1 |
| Staphylococcus aureus TCH130 | NZ_ACHD01000001.1 - NZ_ACHD01000284.1 |
| Staphylococcus aureus TCH70 | NZ_ACHH02000001.1 - NZ_ACHH02000018.1 |
| Staphylococcus aureus USA300 TCH959 | NZ_AASB02000001.1 - NZ_AASB02000256.1 |
| Staphylococcus aureus WBG10049 | ACSV01000001.1 - ACSV01000038.1 |
| Staphylococcus aureus WW2703 97 | ACSW01000001.1 - ACSW01000151.1 |
| Staphylococcus capitis SK14 | NZ_ACFR01000001.1 - NZ_ACFR01000032.1 |
| Staphylococcus caprae C87 | ACRH01000001.1 - ACRH01000046.1 |
| Staphylococcus epidermidis BCM HMP0060 | NZ_ACHE01000001.1 - NZ_ACHE01000115.1 |
| Staphylococcus epidermidis M23864 W1 | NZ_ACJB01000001.1 - NZ_ACJB01000078.1 |
| Staphylococcus epidermidis M23864 W2 grey | NZ_ADMU01000001.1 - NZ_ADMU01000067.1 |
| Staphylococcus epidermidis SK135 | NZ_ADEY01000001.1 - NZ_ADEY01000033.1 |
| Staphylococcus epidermidis W23144 | NZ_ACJC01000001.1 - NZ_ACJC01000192.1 |
| Staphylococcus hominis C80 | ACRM01000001.1 - ACRM01000041.1 |
| Staphylococcus hominis SK119 | NZ_ACLP01000001.1 - NZ_ACLP01000037.1 |
| Staphylococcus lugdunensis M23590 | NZ_AEQA01000001.1 - NZ_AEQA01000025.1 |
| Staphylococcus warneri L37603 | NZ_ACPZ01000001.1 - NZ_ACPZ01000077.1 |
| Stenotrophomonas SKA14 | ACDV01000001.1 - ACDV01000050.1 |
| Stigmatella aurantiaca DW4 3 1 | NZ_AAMD01000001.1 - NZ_AAMD01000579.1 |
| Streptococcus 2 1 36FAA | ACOI01000001.1 - ACOI01000031.1 |
| Streptococcus M143 | ACRK01000001.1 - ACRK01000028.1 |
| Streptococcus agalactiae 18RS21 | NZ_AAJO01000001.1 - NZ_AAJO01000553.1 |
| Streptococcus agalactiae 515 | NZ_AAJP01000001.1 - NZ_AAJP01000255.1 |
| Streptococcus agalactiae CJB111 | NZ_AAJQ01000001.1 - NZ_AAJQ01000155.1 |
| Streptococcus agalactiae COH1 | NZ_AAJR01000001.1 - NZ_AAJR01000393.1 |
| Streptococcus agalactiae H36B | NZ_AAJS01000001.1 - NZ_AAJS01000345.1 |
| Streptococcus anginosus 1 2 62CV | NZ_ADME01000001.1 - NZ_ADME01000018.1 |
| Streptococcus anginosus F0211 | NZ_AECT01000001.1 - NZ_AECT01000063.1 |
| Streptococcus australis ATCC 700641 | NZ_AEQR01000001.1 - NZ_AEQR01000027.1 |
| Streptococcus bovis ATCC 700338 | NZ_AEEL01000001.1 - NZ_AEEL01000038.1 |
| Streptococcus downei F0415 | NZ_AEKN01000001.1 - NZ_AEKN01000017.1 |
| Streptococcus equinus ATCC 9812 | NZ_AEVB01000001.1 - NZ_AEVB01000057.1 |
| Streptococcus gallolyticus TX20005 | NZ_AEEM01000001.1 - NZ_AEEM01000025.1 |
| Streptococcus infantarius ATCC BAA 102 | NZ_ABJK02000001.1 - NZ_ABJK02000022.1 |
| Streptococcus infantis SK1302 | NZ_AEDY01000001.1 - NZ_AEDY01000148.1 |
| Streptococcus mitis ATCC 6249 | NZ_AEEN01000001.1 - NZ_AEEN01000020.1 |
| Streptococcus mitis NCTC 12261 | NZ_AEDX01000001.1 - NZ_AEDX01000024.1 |
| Streptococcus mitis SK321 | NZ_AEDT01000001.1 - NZ_AEDT01000040.1 |
| Streptococcus mitis SK564 | NZ_AEDU01000001.1 - NZ_AEDU01000035.1 |
| Streptococcus mitis SK597 | NZ_AEDV01000001.1 - NZ_AEDV01000108.1 |
| Streptococcus oral taxon 071 73H25AP | NZ_AEEP01000001.1 - NZ_AEEP01000020.1 |
| Streptococcus oralis | NZ_ADMV01000001.1 - NZ_ADMV01000028.1 |
| Streptococcus oralis ATCC 35037 | NZ_AEDW01000001.1 - NZ_AEDW01000025.1 |
| Streptococcus parasanguinis ATCC 15912 | NZ_ADVN01000001.1 - NZ_ADVN01000047.1 |
| Streptococcus parasanguinis F0405 | NZ_AEKM01000001.1 - NZ_AEKM01000016.1 |
| Streptococcus pneumoniae BS397 | NZ_ABWC01000001.1 - NZ_ABWC01000022.1 |
| Streptococcus pneumoniae BS455 | NZ_ADHN01000001.1 - NZ_ADHN01000113.1 |
| Streptococcus pneumoniae BS457 | NZ_ABWB01000001.1 - NZ_ABWB01000097.1 |
| Streptococcus pneumoniae BS458 | NZ_ABWA01000001.1 - NZ_ABWA01000109.1 |
| Streptococcus pneumoniae CCRI 1974 | NZ_ABZC01000001.1 - NZ_ABZC01000121.1 |
| Streptococcus pneumoniae CCRI 1974M2 | NZ_ABZT01000001.1 - NZ_ABZT01000130.1 |
| Streptococcus pneumoniae CDC0288 04 | NZ_ABGF01000001.1 - NZ_ABGF01000038.1 |
| Streptococcus pneumoniae CDC1087 00 | NZ_ABFT01000001.1 - NZ_ABFT01000075.1 |
| Streptococcus pneumoniae CDC1873 00 | NZ_ABFS01000001.1 - NZ_ABFS01000053.1 |
| Streptococcus pneumoniae CDC3059 06 | NZ_ABGG01000001.1 - NZ_ABGG01000033.1 |
| Streptococcus pneumoniae Canada MDR 19A | NZ_ACNU01000001.1 - NZ_ACNU01000127.1 |
| Streptococcus pneumoniae Canada MDR 19F | NZ_ACNV01000001.1 - NZ_ACNV01000277.1 |
| Streptococcus pneumoniae MLV 016 | NZ_ABGH01000001.1 - NZ_ABGH01000126.1 |
| Streptococcus pneumoniae SP11 BS70 | NZ_ABAC01000001.1 - NZ_ABAC01000025.1 |
| Streptococcus pneumoniae SP14 BS292 | NZ_ABWQ01000001.1 - NZ_ABWQ01000025.1 |
| Streptococcus pneumoniae SP14 BS69 | NZ_ABAD01000001.1 - NZ_ABAD01000049.1 |
| Streptococcus pneumoniae SP18 BS74 | NZ_ABAE01000001.1 - NZ_ABAE01000028.1 |
| Streptococcus pneumoniae SP195 | NZ_ABGE01000001.1 - NZ_ABGE01000041.1 |
| Streptococcus pneumoniae SP19 BS75 | NZ_ABAF01000001.1 - NZ_ABAF01000030.1 |
| Streptococcus pneumoniae SP23 BS72 | NZ_ABAG01000001.1 - NZ_ABAG01000032.1 |
| Streptococcus pneumoniae SP3 BS71 | NZ_AAZZ01000001.1 - NZ_AAZZ01000024.1 |
| Streptococcus pneumoniae SP6 BS73 | NZ_ABAA01000001.1 - NZ_ABAA01000038.1 |
| Streptococcus pneumoniae SP9 BS68 | NZ_ABAB01000001.1 - NZ_ABAB01000061.1 |
| Streptococcus pneumoniae SP9v BS293 | NZ_ABWU01000001.1 - NZ_ABWU01000030.1 |
| Streptococcus pneumoniae TIGR4 | NZ_AAGY02000001.1 - NZ_AAGY02000218.1 |
| Streptococcus pseudoporcinus SPIN 20026 | NZ_AENS01000001.1 - NZ_AENS01000035.1 |
| Streptococcus pyogenes ATCC 10782 | NZ_AEEO01000001.1 - NZ_AEEO01000090.1 |
| Streptococcus pyogenes M49 591 | NZ_AAFV01000001.1 - NZ_AAFV01000295.1 |
| Streptococcus salivarius SK126 | NZ_ACLO01000001.1 - NZ_ACLO01000101.1 |
| Streptococcus sanguinis ATCC 49296 | NZ_AEPO01000001.1 - NZ_AEPO01000020.1 |
| Streptococcus suis 05HAH33 | NZ_AARD01000001.1 - NZ_AARD01000332.1 |
| Streptococcus suis 89 1591 | NZ_AAFA03000001.1 - NZ_AAFA03000082.1 |
| Streptococcus vestibularis F0396 | NZ_AEKO01000001.1 - NZ_AEKO01000011.1 |
| Streptomyces AA4 | ACEV01000001.1 - ACEV01000098.1 |
| Streptomyces ACTE | NZ_ADFD01000001.1 - NZ_ADFD01000050.1 |
| Streptomyces ACT 1 | NZ_ADFC01000001.1 - NZ_ADFC01000127.1 |
| Streptomyces C | ACEW01000001.1 - ACEW01000652.1 |
| Streptomyces Mg1 | ABJF01000001.1 - ABJF01000466.1 |
| Streptomyces SA3 actF | NZ_ADXB01000001.1 - NZ_ADXB01001275.1 |
| Streptomyces SA3 actG | NZ_ADXA01000001.1 - NZ_ADXA01000254.1 |
| Streptomyces SPB74 | ABJG02000478.1 - ABJG02000845.1 |
| Streptomyces SPB78 | ACEU01000001.1 - ACEU01000694.1 |
| Streptomyces albus J1074 | ABYC01000001.1 - ABYC01000501.1 |
| Streptomyces clavuligerus ATCC 27064 | ADGD01000001.1 - ABJH01000597.1 |
| Streptomyces e14 | ACUR01000001.1 - ACUR01000716.1 |
| Streptomyces ghanaensis ATCC 14672 | ABYA01000001.1 - ABYA01000616.1 |
| Streptomyces griseoflavus Tu4000 | ACFA01000001.1 - ACFA01000927.1 |
| Streptomyces hygroscopicus ATCC 53653 | ACEX01000001.1 - ACEX01000783.1 |
| Streptomyces lividans TK24 | ACEY01000001.1 - ACEY01000333.1 |
| Streptomyces pristinaespiralis ATCC 25486 | ABJI02000001.1 - ABJI02000844.1 |
| Streptomyces roseosporus NRRL 11379 | NZ_ABYX01000001.1 - NZ_ABYX01000280.1 |
| Streptomyces roseosporus NRRL 15998 | ABYB01000001.1 - ABYB01000371.1 |
| Streptomyces sviceus ATCC 29083 | ABJJ02000001.1 - ABJJ02000552.1 |
| Streptomyces violaceusniger Tu 4113 | NZ_AEDI01000001.1 - NZ_AEDI01000239.1 |
| Streptomyces viridochromogenes DSM 40736 | ACEZ01000001.1 - ACEZ01000226.1 |
| Subdoligranulum variabile DSM 15176 | NZ_ACBY02000001.1 - NZ_ACBY02000091.1 |
| Sulfitobacter EE 36 | NZ_AALV01000001.1 - NZ_AALV01000015.1 |
| Sulfitobacter NAS 14 1 | NZ_AALZ01000001.1 - NZ_AALZ01000027.1 |
| Sulfolobus solfataricus 98 2 | NZ_ACUK01000001.1 - NZ_ACUK01000506.1 |
| Sulfurihydrogenibium yellowstonense SS 5 | NZ_ABZS01000001.1 - NZ_ABZS01000228.1 |
| Sutterella wadsworthensis 3 1 45B | NZ_ADMF01000001.1 - NZ_ADMF01000061.1 |
| Synechococcus BL107 | NZ_AATZ01000001.1 - NZ_AATZ01000006.1 |
| Synechococcus CB0101 | NZ_ADXL01000001.1 - NZ_ADXL01000094.1 |
| Synechococcus CB0205 | NZ_ADXM01000001.1 - NZ_ADXM01000078.1 |
| Synechococcus PCC 7335 | ABRV01000001.1 - ABRV01000038.1 |
| Synechococcus RS9916 | NZ_AAUA01000001.1 - NZ_AAUA01000004.1 |
| Synechococcus RS9917 | NZ_AANP01000001.1 - NZ_AANP01000009.1 |
| Synechococcus WH 5701 | NZ_AANO01000001.1 - NZ_AANO01000135.1 |
| Synechococcus WH 7805 | NZ_AAOK01000001.1 - NZ_AAOK01000013.1 |
| Synechococcus WH 8109 | ACNY01000001.1 - ACNY01000005.1 |
| Thalassobium Thalassiobium R2A62 | ACOA01000001.1 - ACOA01000008.1 |
| Thermaerobacter subterraneus DSM 13965 | NZ_AENY01000001.1 - NZ_AENY01000103.1 |
| Thermoanaerobacter X561 | NZ_ACXP02000001.1 - NZ_ACXP02000008.1 |
| Thermoanaerobacter ethanolicus CCSD1 | NZ_ACXY01000001.1 - NZ_ACXY01000090.1 |
| Thermoanaerobacter wiegelii Rt8 B1 | NZ_ADXD01000001.1 - NZ_ADXD01000169.1 |
| Thermococcus AM4 | ABXN01000001.1 - ABXN01000017.1 |
| Thermosinus carboxydivorans Nor1 | NZ_AAWL01000001.1 - NZ_AAWL01000049.1 |
| Thermus aquaticus Y51MC23 | NZ_ABVK02000001.1 - NZ_ABVK02000022.1 |
| Treponema phagedenis F0421 | NZ_AEFH01000001.1 - NZ_AEFH01000277.1 |
| Treponema vincentii ATCC 35580 | NZ_ACYH01000001.1 - NZ_ACYH01000079.1 |
| Turicibacter PC909 | NZ_ADMN01000001.1 - NZ_ADMN01000125.1 |
| Ureaplasma parvum serovar 14 ATCC 33697 | NZ_ABER01000001.1 - NZ_ABER01000008.1 |
| Ureaplasma parvum serovar 1 ATCC 27813 | NZ_ABES01000001.1 - NZ_ABES01000012.1 |
| Ureaplasma parvum serovar 6 ATCC 27818 | NZ_AAZQ01000001.1 - NZ_AAZQ01000005.1 |
| Ureaplasma urealyticum serovar 11 ATCC 33695 | NZ_AAZS01000001.1 - NZ_AAZS01000009.1 |
| Ureaplasma urealyticum serovar 12 ATCC 33696 | NZ_AAZT01000001.1 - NZ_AAZT01000005.1 |
| Ureaplasma urealyticum serovar 13 ATCC 33698 | NZ_ABEV01000001.1 - NZ_ABEV01000005.1 |
| Ureaplasma urealyticum serovar 2 ATCC 27814 | NZ_ABFL02000001.1 - NZ_ABFL02000009.1 |
| Ureaplasma urealyticum serovar 4 ATCC 27816 | NZ_AAYO02000001.1 - NZ_AAYO02000004.1 |
| Ureaplasma urealyticum serovar 5 ATCC 27817 | NZ_AAZR01000001.1 - NZ_AAZR01000018.1 |
| Ureaplasma urealyticum serovar 7 ATCC 27819 | NZ_AAYP01000001.1 - NZ_AAYP01000020.1 |
| Ureaplasma urealyticum serovar 8 ATCC 27618 | NZ_AAYN02000001.1 - NZ_AAYN02000002.1 |
| Ureaplasma urealyticum serovar 9 ATCC 33175 | NZ_AAYQ02000001.1 - NZ_AAYQ02000010.1 |
| Veillonella 3 1 44 | ADCV01000001.1 - ADCV01000031.1 |
| Veillonella 6 1 27 | ADCW01000001.1 - ADCW01000022.1 |
| Veillonella atypica ACS 049 V Sch6 | NZ_AEDR01000001.1 - NZ_AEDR01000063.1 |
| Veillonella atypica ACS 134 V Col7a | NZ_AEDS01000001.1 - NZ_AEDS01000070.1 |
| Veillonella dispar ATCC 17748 | NZ_ACIK02000001.1 - NZ_ACIK02000025.1 |
| Veillonella oral taxon 158 F0412 | NZ_AENU01000001.1 - NZ_AENU01000021.1 |
| Veillonella parvula ATCC 17745 | NZ_ADFU01000001.1 - NZ_ADFU01000019.1 |
| Verrucomicrobium spinosum DSM 4136 | NZ_ABIZ01000001.1 - NZ_ABIZ01000001.1 |
| Vibrio AND4 | NZ_ABGR01000001.1 - NZ_ABGR01000143.1 |
| Vibrio Ex25 | AAKK02000001.1 - AAKK02000222.1 |
| Vibrio MED222 | NZ_AAND01000001.1 - NZ_AAND01000099.1 |
| Vibrio RC341 | NZ_ACZT01000001.1 - NZ_ACZT01000028.1 |
| Vibrio RC586 | NZ_ADBD01000001.1 - NZ_ADBD01000016.1 |
| Vibrio alginolyticus 12G01 | NZ_AAPS01000001.1 - NZ_AAPS01000106.1 |
| Vibrio alginolyticus 40B | NZ_ACZB01000001.1 - NZ_ACZB01000188.1 |
| Vibrio caribbenthicus ATCC BAA 2122 | NZ_AEIU01000001.1 - NZ_AEIU01000126.1 |
| Vibrio cholerae 12129 1 | NZ_ACFQ01000001.1 - NZ_ACFQ01000012.1 |
| Vibrio cholerae 1587 | NZ_AAUR01000001.1 - NZ_AAUR01000254.1 |
| Vibrio cholerae 2740 80 | NZ_AAUT01000001.1 - NZ_AAUT01000257.1 |
| Vibrio cholerae 623 39 | NZ_AAWG01000001.1 - NZ_AAWG01000314.1 |
| Vibrio cholerae AM 19226 | AATY01000001.1 - AATY01000154.1 |
| Vibrio cholerae B33 | NZ_AAWE01000001.1 - NZ_ACHZ01000017.1 |
| Vibrio cholerae BX 330286 | NZ_ACIA01000001.1 - NZ_ACIA01000008.1 |
| Vibrio cholerae CIRS101 | NZ_ACVW01000001.1 - NZ_ACVW01000018.1 |
| Vibrio cholerae CT 5369 93 | NZ_ADAL01000001.1 - NZ_ADAL01000269.1 |
| Vibrio cholerae INDRE 91 1 | NZ_ADAK01000001.1 - NZ_ADAK01000060.1 |
| Vibrio cholerae MAK 757 | AAUS02000001.1 - AAUS02000043.1 |
| Vibrio cholerae MO10 | AAKF03000001.1 - AAKF03000084.1 |
| Vibrio cholerae MZO 2 | NZ_AAWF01000001.1 - NZ_AAWF01000162.1 |
| Vibrio cholerae MZO 3 | NZ_AAUU01000001.1 - NZ_AAUU01000292.1 |
| Vibrio cholerae NCTC 8457 | NZ_AAWD01000001.1 - NZ_AAWD01000390.1 |
| Vibrio cholerae RC27 | NZ_ADAI01000001.1 - NZ_ADAI01000045.1 |
| Vibrio cholerae RC385 | AAKH03000001.1 - AAKH03000097.1 |
| Vibrio cholerae RC9 | NZ_ACHX01000001.1 - NZ_ACHX01000011.1 |
| Vibrio cholerae TMA 21 | NZ_ACHY01000001.1 - NZ_ACHY01000020.1 |
| Vibrio cholerae TM 11079 80 | NZ_ACHW01000001.1 - NZ_ACHW01000035.1 |
| Vibrio cholerae V51 | AAKI02000001.1 - AAKI02000360.1 |
| Vibrio cholerae V52 | NZ_AAKJ02000001.1 - NZ_AAKJ02000268.1 |
| Vibrio cholerae bv albensis VL426 | NZ_ACHV01000001.1 - NZ_ACHV01000005.1 |
| Vibrio coralliilyticus ATCC BAA 450 | NZ_ACZN01000001.1 - NZ_ACZN01000020.1 |
| Vibrio furnissii CIP 102972 | NZ_ACZP01000001.1 - NZ_ACZP01000024.1 |
| Vibrio harveyi 1DA3 | NZ_ACZC01000001.1 - NZ_ACZC01000140.1 |
| Vibrio harveyi HY01 | NZ_AAWP01000001.1 - NZ_AAWP01000349.1 |
| Vibrio metschnikovii CIP 69 14 | NZ_ACZO01000001.1 - NZ_ACZO01000011.1 |
| Vibrio mimicus MB 451 | NZ_ADAF01000001.1 - NZ_ADAF01000003.1 |
| Vibrio mimicus VM223 | NZ_ADAJ01000001.1 - NZ_ADAJ01000008.1 |
| Vibrio mimicus VM573 | NZ_ACYV01000001.1 - NZ_ACYV01000074.1 |
| Vibrio mimicus VM603 | NZ_ACYU01000001.1 - NZ_ACYU01000195.1 |
| Vibrio orientalis CIP 102891 | NZ_ACZV01000001.1 - NZ_ACZV01000005.1 |
| Vibrio parahaemolyticus 16 | ACCV01000001.1 - ACCV01000178.1 |
| Vibrio parahaemolyticus AN 5034 | NZ_ACFO01000001.1 - NZ_ACFO01000054.1 |
| Vibrio parahaemolyticus AQ3810 | NZ_AAWQ01000001.1 - NZ_AAWQ01001073.1 |
| Vibrio parahaemolyticus AQ4037 | NZ_ACFN01000001.1 - NZ_ACFN01000164.1 |
| Vibrio parahaemolyticus K5030 | NZ_ACKB01000001.1 - NZ_ACKB01000164.1 |
| Vibrio parahaemolyticus Peru 466 | NZ_ACFM01000001.1 - NZ_ACFM01000149.1 |
| Vibrio shilonii AK1 | NZ_ABCH01000001.1 - NZ_ABCH01000158.1 |
| Vibrio splendidus 12B01 | NZ_AAMR01000001.1 - NZ_AAMR01000119.1 |
| Victivallis vadensis ATCC BAA 548 | NZ_ABDE02000001.1 - NZ_ABDE02000027.1 |
| Weissella paramesenteroides ATCC 33313 | NZ_ACKU01000001.1 - NZ_ACKU01000036.1 |
| Wolbachia endosymbiont of Culex quinquefasciatus JHB | NZ_ABZA01000001.1 - NZ_ABZA01000021.1 |
| Wolbachia endosymbiont of Drosophila ananassae | NZ_AAGB01000001.1 - NZ_AAGB01000464.1 |
| Wolbachia endosymbiont of Drosophila simulans | NZ_AAGC01000001.1 - NZ_AAGC01000629.1 |
| Wolbachia endosymbiont of Drosophila willistoni TSC 14030 0811 24 | NZ_AAQP01000001.1 - NZ_AAQP01000260.1 |
| Wolbachia endosymbiont of Muscidifurax uniraptor | NZ_ACFP01000001.1 - NZ_ACFP01000256.1 |
| Xanthomonas campestris musacearum NCPPB4381 | NZ_ACHT01000001.1 - NZ_ACHT01000751.1 |
| Xanthomonas campestris vasculorum NCPPB702 | NZ_ACHS01000001.1 - NZ_ACHS01000488.1 |
| Xanthomonas fuscans aurantifolii ICPB 10535 | NZ_ACPY01000001.1 - NZ_ACPY01000351.1 |
| Xanthomonas fuscans aurantifolii ICPB 11122 | NZ_ACPX01000001.1 - NZ_ACPX01000237.1 |
| Xanthomonas oryzae oryzicola BLS256 | NZ_AAQN01000001.1 - NZ_AAQN01000001.1 |
| Xylella fastidiosa Dixon | NZ_AAAL02000001.1 - NZ_AAAL02000032.1 |
| Xylella fastidiosa sandyi Ann 1 | NZ_AAAM03000001.1 - NZ_AAAM03000219.1 |
| Yersinia aldovae ATCC 35236 | NZ_ACCB01000001.1 - NZ_ACCB01000210.1 |
| Yersinia bercovieri ATCC 43970 | NZ_AALC02000001.1 - NZ_AALC02000229.1 |
| Yersinia frederiksenii ATCC 33641 | NZ_AALE02000001.1 - NZ_AALE02000161.1 |
| Yersinia intermedia ATCC 29909 | NZ_AALF02000001.1 - NZ_AALF02000123.1 |
| Yersinia kristensenii ATCC 33638 | NZ_ACCA01000001.1 - NZ_ACCA01000153.1 |
| Yersinia mollaretii ATCC 43969 | NZ_AALD02000001.1 - NZ_AALD02000179.1 |
| Yersinia pestis CA88 4125 | NZ_ABCD01000001.1 - NZ_ABCD01000008.1 |
| Yersinia pestis FV 1 | NZ_AAUB01000001.1 - NZ_AAUB01000400.1 |
| Yersinia pestis KIM D27 | NZ_ADDC01000001.1 - NZ_ADDC01000009.1 |
| Yersinia pestis Nepal516 | NZ_ACNQ01000001.1 - NZ_ACNQ01000023.1 |
| Yersinia pestis Pestoides A | NZ_ACNT01000001.1 - NZ_ACNT01000037.1 |
| Yersinia pestis biovar Antiqua B42003004 | NZ_AAYU01000001.1 - NZ_AAYU01000069.1 |
| Yersinia pestis biovar Antiqua E1979001 | NZ_AAYV01000001.1 - NZ_AAYV01000075.1 |
| Yersinia pestis biovar Antiqua UG05 0454 | NZ_AAYR01000001.1 - NZ_AAYR01000098.1 |
| Yersinia pestis biovar Mediaevalis K1973002 | NZ_AAYT01000001.1 - NZ_AAYT01000073.1 |
| Yersinia pestis biovar Orientalis F1991016 | NZ_ABAT01000001.1 - NZ_ABAT01000107.1 |
| Yersinia pestis biovar Orientalis IP275 | NZ_AAOS02000001.1 - NZ_AAOS02000101.1 |
| Yersinia pestis biovar Orientalis India 195 | NZ_ACNR01000001.1 - NZ_ACNR01000033.1 |
| Yersinia pestis biovar Orientalis MG05 1020 | NZ_AAYS01000001.1 - NZ_AAYS01000080.1 |
| Yersinia pestis biovar Orientalis PEXU2 | NZ_ACNS01000001.1 - NZ_ACNS01000005.1 |
| Yersinia rohdei ATCC 43380 | NZ_ACCD01000001.1 - NZ_ACCD01000141.1 |
| Yersinia ruckeri ATCC 29473 | NZ_ACCC01000001.1 - NZ_ACCC01000174.1 |
| Zymomonas mobilis ATCC 10988 | NZ_ACQU01000001.1 - NZ_ACQU01000024.1 |
| Alteromonadales bacterium TW 7 | NZ_AAVS01000001.1 - NZ_AAVS01000076.1 |
| Bacteroidetes oral taxon 274 F0058 | ADCM01000001.1 - ADCM01000028.1 |
| Burkholderiales bacterium 1 1 47 | ADCQ01000001.1 - ADCQ01000096.1 |
| Campylobacterales bacterium GD 1 | ABXD01000001.1 - ABXD01000026.1 |
| Citreicella SE45 | ACNW01000001.1 - ACNW01000122.1 |
| Clostridiales bacterium 1 7 47FAA | ABQR01000001.1 - ABQR01000172.1 |
| Clostridium ramosum DSM 1402 | NZ_ABFX02000001.1 - NZ_ABFX02000016.1 |
| Clostridium spiroforme DSM 1552 | NZ_ABIK02000001.1 - NZ_ABIK02000016.1 |
| Enterobacteriaceae bacterium 9 2 54FAA | NZ_ADCU01000001.1 - NZ_ADCU01000062.1 |
| Erysipelotrichaceae bacterium 3 1 53 | NZ_ACTJ01000001.1 - NZ_ACTJ01000163.1 |
| Erysipelotrichaceae bacterium 5 2 54FAA | ACZW01000001.1 - ACZW01000087.1 |
| Escherichia 1 1 43 | ACID01000001.1 - ACID01000091.1 |
| Escherichia 3 2 53FAA | ACAC01000001.1 - ACAC01000173.1 |
| Escherichia 4 1 40B | NZ_ACDM01000001.1 - NZ_ACDM01000126.1 |
| Eubacterium biforme DSM 3989 | NZ_ABYT01000001.1 - NZ_ABYT01000161.1 |
| Eubacterium dolichum DSM 3991 | NZ_ABAW02000001.1 - NZ_ABAW02000025.1 |
| Flavobacteria bacterium BAL38 | NZ_AAXX01000001.1 - NZ_AAXX01000014.1 |
| Flavobacteria bacterium BBFL7 | NZ_AAPD01000001.1 - NZ_AAPD01000017.1 |
| Flavobacteria bacterium MS024 2A | NZ_ABVV01000001.1 - NZ_ABVV01000017.1 |
| Flavobacteria bacterium MS024 3C | NZ_ABVW01000001.1 - NZ_ABVW01000021.1 |
| Flavobacteriales bacterium ALC 1 | NZ_ABHI01000001.1 - NZ_ABHI01000008.1 |
| Grimontia hollisae CIP 101886 | NZ_ADAQ01000001.1 - NZ_ADAQ01000013.1 |
| Lachnospiraceae bacterium 5 1 63FAA | NZ_ACTS01000001.1 - NZ_ACTS01000094.1 |
| Lachnospiraceae bacterium 8 1 57FAA | NZ_ACWQ01000001.1 - NZ_ACWQ01000115.1 |
| Methylophaga thiooxidans DMS010 | ABXT01000001.1 - ABXT01000043.1 |
| Methylophilales bacterium HTCC2181 | NZ_AAUX01000001.1 - NZ_AAUX01000003.1 |
| Rhodobacteraceae bacterium KLH11 | ACCW01000001.1 - ACCW01000048.1 |
| Rhodobacterales bacterium HTCC2083 | ABXE01000001.1 - ABXE01000020.1 |
| Rhodobacterales bacterium HTCC2150 | NZ_AAXZ01000001.1 - NZ_AAXZ01000025.1 |
| Rhodobacterales bacterium HTCC2255 | NZ_AATR01000001.1 - NZ_AATR01000065.1 |
| Rhodobacterales bacterium Y4I | ABXF01000001.1 - ABXF01000063.1 |
| Roseobacter AzwK 3b | NZ_ABCR01000001.1 - NZ_ABCR01000031.1 |
| Roseobacter CCS2 | NZ_AAYB01000001.1 - NZ_AAYB01000011.1 |
| Roseobacter GAI101 | ABXS01000001.1 - ABXS01000067.1 |
| Roseobacter MED193 | NZ_AANB01000001.1 - NZ_AANB01000019.1 |
| Roseobacter SK209 2 6 | NZ_AAYC01000001.1 - NZ_AAYC01000029.1 |
| Ruminococcaceae bacterium D16 | NZ_ADDX01000001.1 - NZ_ADDX01000104.1 |
| Thermotogales bacterium mesG1 Ag 4 2 | NZ_AEDC01000001.1 - NZ_AEDC01000038.1 |
| Verrucomicrobiae bacterium DG1235 | ABSI01000001.1 - ABSI01000026.1 |
| Vibrionales bacterium SWAT 3 | NZ_AAZW01000001.1 - NZ_AAZW01000154.1 |
| alpha proteobacterium BAL199 | NZ_ABHC01000001.1 - NZ_ABHC01000069.1 |
| alpha proteobacterium HIMB114 | ADAC01000001.1 - ADAC01000026.1 |
| bacterium Ellin514 | NZ_ABOX02000001.1 - NZ_ABOX02000102.1 |
| beta proteobacterium KB13 | ABXG01000001.1 - ABXG01000004.1 |
| candidate division TM7 genomosp GTL1 | NZ_AAXS01000001.1 - NZ_AAXS01000132.1 |
| candidate division TM7 single cell isolate TM7a | NZ_ABBV01000001.1 - NZ_ABBV01008553.1 |
| candidate division TM7 single cell isolate TM7b | NZ_ABBW01000001.1 - NZ_ABBW01000085.1 |
| candidate division TM7 single cell isolate TM7c | NZ_ABBX01000001.1 - NZ_ABBX01000129.1 |
| delta proteobacterium MLMS 1 | NZ_AAQF01000001.1 - NZ_AAQF01000545.1 |
| delta proteobacterium NaphS2 | NZ_ADZZ01000001.1 - NZ_ADZZ01000810.1 |
| gamma proteobacterium HTCC2207 | NZ_AAPI01000001.1 - NZ_AAPI01000021.1 |
| gamma proteobacterium HTCC5015 | ABSJ01000001.1 - ABSJ01000062.1 |
| gamma proteobacterium NOR51 B | ACCY01000001.1 - ACCY01000029.1 |
| gamma proteobacterium NOR5 3 | ACCX01000001.1 - ACCX01000029.1 |
| marine actinobacterium PHSC20C1 | NZ_AAOB01000001.1 - NZ_AAOB01000023.1 |
| marine gamma proteobacterium HTCC2080 | NZ_AAVV01000001.1 - NZ_AAVV01000025.1 |
| marine gamma proteobacterium HTCC2143 | NZ_AAVT01000001.1 - NZ_AAVT01000030.1 |
| marine gamma proteobacterium HTCC2148 | ABXQ01000001.1 - ABXQ01000075.1 |
| unidentified eubacterium SCB49 | NZ_ABCO01000001.1 - NZ_ABCO01000025.1 |
| Viruses |  |
| AHJD-like viruses Staphylococcus phage 66 | NC_007046.1 |
| AHJD-like viruses Staphylococcus phage SAP 2 | NC_009875.1 |
| AHJD-like viruses Streptococcus phage C1 | NC_004814.1 |
| Acidovorax Acidovorax avenae ATCC 19860 | NZ_ADCB01000001.1 - NZ_ADCB01000084.1 |
| Alfamovirus Alfalfa mosaic virus | NC_001495.1 - NC_002025.1 |
| Allexivirus Garlic virus A | NC_003375.1 |
| Allexivirus Garlic virus C | NC_003376.1 |
| Allexivirus Garlic virus E | NC_004012.1 |
| Allexivirus Garlic virus X | NC_001800.1 |
| Allexivirus Shallot virus X | NC_003795.1 |
| Allolevivirus Enterobacteria phage FI sensu lato | NC_004301.1 |
| Alphabaculovirus Adoxophyes honmai NPV | NC_004690.1 |
| Alphabaculovirus Agrotis ipsilon multiple nucleopolyhedrovirus | NC_011345.1 |
| Alphabaculovirus Bombyx mandarina nucleopolyhedrovirus | NC_012672.1 |
| Alphabaculovirus Bombyx mori NPV | NC_001962.1 |
| Alphabaculovirus Choristoneura fumiferana DEF MNPV | NC_005137.2 |
| Alphabaculovirus Choristoneura fumiferana MNPV | NC_004778.3 |
| Alphabaculovirus Ecotropis obliqua NPV | NC_008586.1 |
| Alphabaculovirus Helicoverpa armigera NPV | NC_011354.1 |
| Alphabaculovirus Helicoverpa zea SNPV | NC_003349.1 |
| Alphabaculovirus Lymantria dispar MNPV | NC_001973.1 |
| Alphabaculovirus Mamestra configurata NPV B | NC_004117.1 |
| Alphabaculovirus Spodoptera exigua MNPV | NC_002169.1 |
| Alphabaculovirus Spodoptera frugiperda MNPV | NC_009011.2 |
| Alphabaculovirus Spodoptera litura NPV | NC_003102.1 |
| Alphabaculovirus Trichoplusia ni SNPV | NC_007383.1 |
| Alphacoronavirus Human coronavirus 229E | NC_002645.1 |
| Alphacoronavirus Human coronavirus NL63 | NC_005831.2 |
| Alphacryptovirus Beet cryptic virus 1 | NC_011556.1 NC_011557.1 |
| Alphacryptovirus Vicia cryptic virus | NC_007241.1 NC_007242.1 |
| Alphanodavirus Black beetle virus | NC_001411.2 NC_002037.1 |
| Alphanodavirus Boolarra virus | NC_004142.1 NC_004145.1 |
| Alphanodavirus Flock house virus | NC_004144.1 NC_004146.1 |
| Alphanodavirus Nodamura virus | NC_002690.1 NC_002691.1 |
| Alphanodavirus Pariacato virus | NC_003691.1 NC_003692.1 |
| Alphapapillomavirus Human papillomavirus 54 | NC_001676.1 |
| Alphapapillomavirus Rhesus monkey papillomavirus 1 | NC_001678.1 |
| Alpharetrovirus Avian leukosis virus | NC_001408.1 |
| Alpharetrovirus Fujinami sarcoma virus | NC_001403.1 |
| Alpharetrovirus Rous sarcoma virus | NC_001407.1 |
| Alpharetrovirus UR2 sarcoma virus | NC_001618.1 |
| Alpharetrovirus Y73 sarcoma virus | NC_008094.1 |
| Alphatorquevirus Torque teno virus 1 | NC_002076.2 |
| Alphatorquevirus Torque teno virus 10 | NC_014076.1 |
| Alphatorquevirus Torque teno virus 12 | NC_014075.1 |
| Alphatorquevirus Torque teno virus 14 | NC_014077.1 |
| Alphatorquevirus Torque teno virus 15 | NC_014096.1 |
| Alphatorquevirus Torque teno virus 16 | NC_014091.1 |
| Alphatorquevirus Torque teno virus 19 | NC_014078.1 |
| Alphatorquevirus Torque teno virus 25 | NC_014083.1 |
| Alphatorquevirus Torque teno virus 26 | NC_014079.1 |
| Alphatorquevirus Torque teno virus 27 | NC_014074.1 |
| Alphatorquevirus Torque teno virus 28 | NC_014073.1 |
| Alphatorquevirus Torque teno virus 3 | NC_014081.1 |
| Alphatorquevirus Torque teno virus 4 | NC_014069.1 |
| Alphatorquevirus Torque teno virus 6 | NC_014094.1 |
| Alphatorquevirus Torque teno virus 7 | NC_014080.1 |
| Alphatorquevirus Torque teno virus 8 | NC_014084.1 |
| Alphavirus Aura virus | NC_003900.1 |
| Alphavirus Barmah Forest virus | NC_001786.1 |
| Alphavirus Salmon pancreas disease virus | NC_003930.1 |
| Amdovirus Aleutian mink disease virus | NC_001662.1 |
| Ampelovirus Grapevine leafroll associated virus 3 | NC_004667.1 |
| Ampelovirus Little cherry virus 2 | NC_005065.1 |
| Ampelovirus Pineapple mealybug wilt associated virus 1 | NC_010178.1 |
| Ampullavirus Acidianus bottle shaped virus | NC_009452.1 |
| Aphthovirus Equine rhinitis A virus | NC_003982.1 |
| Aquabirnavirus Infectious pancreatic necrosis virus | NC_001915.1 NC_001916.1 |
| Aquareovirus Aquareovirus C | NC_005166.1 - NC_005176.1 |
| Arterivirus Equine arteritis virus | NC_002532.2 |
| Ascovirus Diadromus pulchellus ascovirus 4a | NC_011335.1 |
| Ascovirus Spodoptera frugiperda ascovirus 1a | NC_008361.1 |
| Asfivirus African swine fever virus | NC_001659.1 |
| Atadenovirus Bovine adenovirus D | NC_002685.2 |
| Atadenovirus Duck adenovirus A | NC_001813.1 |
| Atadenovirus Ovine adenovirus D | NC_004037.2 |
| Aureusvirus Cucumber leaf spot virus | NC_007816.1 |
| Aureusvirus Johnsongrass chlorotic stripe mosaic virus | NC_005287.1 |
| Aureusvirus Maize white line mosaic virus | NC_009533.1 |
| Aureusvirus Pothos latent virus | NC_000939.1 |
| Autographa Autographa californica | NC_001623.1 |
| Avastrovirus Chicken astrovirus | NC_003790.1 |
| Avastrovirus Turkey astrovirus | NC_002470.1 |
| Avenavirus Oat chlorotic stunt virus | NC_003633.1 |
| Aviadenovirus Fowl adenovirus A | NC_001720.1 |
| Aviadenovirus Fowl adenovirus D | NC_000899.1 |
| Avibirnavirus Infectious bursal disease virus | NC_004178.1 NC_004179.1 |
| Avihepadnavirus Duck hepatitis B virus | NC_001344.1 |
| Avihepadnavirus Heron hepatitis B virus | NC_001486.1 |
| Avipoxvirus Canarypox virus | NC_005309.1 |
| Avipoxvirus Fowlpox virus | NC_002188.1 |
| Avulavirus Avian paramyxovirus 6 | NC_003043.1 |
| Babuvirus Abaca bunchy top virus | NC_010314.1 - NC_010319.1 |
| Babuvirus Banana bunchy top virus | NC_003473.1 - NC_003479.1 |
| Bacillariodnavirus Chaetoceros salsugineum DNA virus | NC_007193.2 |
| Badnavirus Banana streak GF virus | NC_007002.1 |
| Badnavirus Banana streak Mys virus | NC_006955.1 |
| Badnavirus Banana streak OL virus | NC_003381.1 |
| Badnavirus Cacao swollen shoot virus | NC_001574.1 |
| Badnavirus Citrus yellow mosaic virus | NC_003382.1 |
| Badnavirus Commelina yellow mottle virus | NC_001343.1 |
| Badnavirus Dioscorea bacilliform virus | NC_009010.1 |
| Badnavirus Sugarcane bacilliform Mor virus | NC_008017.1 |
| Badnavirus Taro bacilliform virus | NC_004450.1 |
| Bafinivirus White bream virus | NC_008516.1 |
| Batrachovirus Ranid herpesvirus 1 | NC_008211.1 |
| Batrachovirus Ranid herpesvirus 2 | NC_008210.1 |
| Bdellomicrovirus Bdellovibrio phage phiMH2K | NC_002643.1 |
| Begomovirus Abutilon mosaic virus | NC_001928.2 NC_001929.2 |
| Begomovirus African cassava mosaic virus | NC_001467.1 NC_001468.1 |
| Begomovirus Ageratum enation virus | NC_003434.1 |
| Begomovirus Ageratum yellow vein China virus | NC_004090.1 |
| Begomovirus Ageratum yellow vein Sri Lanka virus | NC_002981.1 |
| Begomovirus Ageratum yellow vein Taiwan virus | NC_004627.1 |
| Begomovirus Ageratum yellow vein virus | NC_003403.1 NC_004626.1 |
| Begomovirus Bean calico mosaic virus | NC_003504.1 NC_003505.1 |
| Begomovirus Bean dwarf mosaic virus | NC_001930.1 NC_001931.1 |
| Begomovirus Bean golden mosaic virus | NC_004042.1 NC_004043.1 |
| Begomovirus Bean golden yellow mosaic virus | NC_001438.1 NC_001439.1 |
| Begomovirus Bhendi yellow vein mosaic virus | NC_003405.1 NC_003418.1 |
| Begomovirus Cabbage leaf curl virus | NC_003866.1 NC_003887.1 |
| Begomovirus Chilli leaf curl virus | NC_005048.1 NC_004628.1 |
| Begomovirus Chino del tomate virus | NC_003830.1 NC_003831.1 |
| Begomovirus Cotton leaf crumple virus | NC_004580.1 NC_004581.1 |
| Begomovirus Cotton leaf curl Alabad virus | NC_004582.1 |
| Begomovirus Cotton leaf curl Gezira virus | NC_006935.1 NC_002510.1 |
| Begomovirus Cotton leaf curl Kokhran virus | NC_004583.1 |
| Begomovirus Cotton leaf curl Multan virus | NC_004607.1 |
| Begomovirus Cotton leaf curl Rajasthan virus | NC_003199.1 |
| Begomovirus Croton yellow vein mosaic virus | NC_004300.1 |
| Begomovirus Cucurbit leaf crumple virus | NC_002984.1 NC_002985.1 |
| Begomovirus Dicliptera yellow mottle virus | NC_003856.1 NC_003857.1 |
| Begomovirus Dolichos yellow mosaic virus | NC_005338.1 |
| Begomovirus East African cassava mosaic Cameroon virus | NC_004625.1 NC_004630.1 |
| Begomovirus East African cassava mosaic Kenya virus | NC_011583.1 NC_011584.1 |
| Begomovirus East African cassava mosaic virus | NC_004674.1 NC_004676.1 |
| Begomovirus Honeysuckle yellow vein mosaic virus | NC_008793.1 |
| Begomovirus Honeysuckle yellow vein virus | NC_005807.1 |
| Begomovirus Ipomoea yellow vein virus | NC_013022.2 |
| Begomovirus Mungbean yellow mosaic India virus | NC_004608.1 NC_004609.1 |
| Begomovirus Okra leaf curl virus | NC_013017.1 |
| Begomovirus Papaya leaf curl virus | NC_004147.1 |
| Begomovirus Pepper leaf curl virus | NC_000882.1 |
| Begomovirus Sida golden mosaic Florida virus | NC_014447.1 |
| Begomovirus Sida mottle virus | NC_004637.1 |
| Begomovirus Sida yellow mosaic Yucatan virus | NC_008779.1 NC_008780.1 |
| Begomovirus Sida yellow mosaic virus | NC_006267.1 NC_004639.1 |
| Begomovirus Sida yellow vein virus | NC_004661.1 NC_004662.1 |
| Begomovirus Squash leaf curl China virus | NC_007339.1 |
| Begomovirus Squash leaf curl Yunnan virus | NC_004651.1 |
| Begomovirus Squash leaf curl virus | NC_001936.1 NC_001937.1 |
| Begomovirus Sri Lankan cassava mosaic virus | NC_003861.1 NC_003862.1 |
| Begomovirus Sweet potato leaf curl Bengal virus | NC_013640.2 |
| Begomovirus Sweet potato leaf curl Canary virus | NC_013465.1 |
| Begomovirus Sweet potato leaf curl Lanzarote virus | NC_013467.1 |
| Begomovirus Sweet potato leaf curl Spain virus | NC_011052.2 |
| Begomovirus Sweet potato leaf curl virus | NC_004650.1 |
| Begomovirus Tomato leaf curl Bangalore virus | NC_010148.1 |
| Begomovirus Tomato leaf curl Cameroon virus | NC_013639.1 |
| Begomovirus Tomato leaf curl Hsinchu virus | NC_008727.1 |
| Begomovirus Tomato leaf curl Pune virus | NC_008517.1 |
| Begomovirus Tomato leaf curl Sinaloa virus | NC_009605.1 NC_009606.1 |
| Begomovirus Tomato leaf curl virus | NC_003896.1 |
| Begomovirus Tomato mottle virus | NC_001938.1 NC_001939.1 |
| Begomovirus Tomato severe rugose virus | NC_009607.1 NC_009612.1 |
| Benyvirus Beet necrotic yellow vein virus | NC_003513.1 - NC_003517.1 |
| Benyvirus Beet soil borne mosaic virus | NC_003503.1 - NC_003508.1 |
| Betabaculovirus Adoxophyes orana granulovirus | NC_005038.1 |
| Betabaculovirus Cryptophlebia leucotreta granulovirus | NC_005068.1 |
| Betabaculovirus Cydia pomonella granulovirus | NC_002816.1 |
| Betabaculovirus Helicoverpa armigera granulovirus | NC_010240.1 |
| Betabaculovirus Pseudaletia unipuncta granulovirus | NC_013772.1 |
| Betacoronavirus Human coronavirus HKU1 | NC_006577.2 |
| Betacoronavirus Severe acute respiratory syndrome related coronavirus | NC_004718.3 |
| Betalipothrixvirus Acidianus filamentous virus 3 | NC_010155.1 |
| Betalipothrixvirus Acidianus filamentous virus 6 | NC_010152.1 |
| Betalipothrixvirus Acidianus filamentous virus 7 | NC_010153.1 |
| Betalipothrixvirus Acidianus filamentous virus 8 | NC_010154.1 |
| Betalipothrixvirus Acidianus filamentous virus 9 | NC_010537.1 |
| Betanodavirus Barfin flounder nervous necrosis virus | NC_013458.1 NC_013459.1 |
| Betanodavirus Redspotted grouper nervous necrosis virus | NC_008040.1 NC_008041.1 |
| Betanodavirus Tiger puffer nervous necrosis virus | NC_013460.1 NC_013461.1 |
| Betaretrovirus Squirrel monkey retrovirus | NC_001514.1 |
| Betatetravirus Nudaurelia capensis beta virus | NC_001990.1 |
| Betatetravirus Providence virus | NC_014126.1 |
| Betatorquevirus Torque teno mini virus 1 | NC_014097.1 |
| Betatorquevirus Torque teno mini virus 2 | NC_014086.1 |
| Betatorquevirus Torque teno mini virus 3 | NC_014088.1 |
| Betatorquevirus Torque teno mini virus 4 | NC_014090.1 |
| Betatorquevirus Torque teno mini virus 5 | NC_014089.1 |
| Betatorquevirus Torque teno mini virus 6 | NC_014095.1 |
| Betatorquevirus Torque teno mini virus 7 | NC_014082.1 |
| Betatorquevirus Torque teno mini virus 8 | NC_014068.1 |
| Betatorquevirus Torque teno mini virus 9 | NC_002195.1 |
| Bicaudavirus Acidianus two tailed virus | NC_007409.1 |
| Bicaudavirus Sulfolobus virus STSV1 | NC_006268.1 |
| Blosnavirus Blotched snakehead virus | NC_005982.1 NC_005983.1 |
| Bocavirus Bovine parvovirus | NC_001540.1 |
| Bocavirus Canine minute virus | NC_004442.1 |
| Bornavirus Borna disease virus | NC_001607.1 |
| Botrexvirus Botrytis virus X | NC_005132.1 |
| Bpp-1-like viruses Bordetella phage BIP 1 | NC_005809.1 |
| Bpp-1-like viruses Bordetella phage BMP 1 | NC_005808.1 |
| Bpp-1-like viruses Bordetella phage BPP 1 | NC_005357.1 |
| Bpp-1-like viruses Burkholderia phage BcepC6B | NC_005887.1 |
| Bracovirus Cotesia congregata bracovirus | NC_006633.1 - NC_006662.1 |
| Bracovirus Microplitis demolitor bracovirus | NC_007028.1 - NC_007044.1 |
| Brambyvirus Blackberry virus Y | NC_008558.1 |
| Brevidensovirus Aedes aegypti densovirus | NC_012636.1 |
| Brevidensovirus Aedes albopictus densovirus | NC_004285.1 |
| Bromovirus Broad bean mottle virus | NC_004006.1 - NC_004008.1 |
| Bromovirus Brome mosaic virus | NC_002026.1 - NC_002028.2 |
| Bromovirus Cassia yellow blotch virus | NC_006999.1 - NC_007001.1 |
| Bromovirus Cowpea chlorotic mottle virus | NC_003541.1 - NC_003543.1 |
| Bromovirus Melandrium yellow fleck virus | NC_013266.1 - NC_013268.1 |
| Bromovirus Spring beauty latent virus | NC_004120.1 - NC_004122.1 |
| Bymovirus Barley mild mosaic virus | NC_003482.1 NC_003483.1 |
| Bymovirus Barley yellow mosaic virus | NC_002990.1 NC_002991.1 |
| Bymovirus Oat mosaic virus | NC_004016.1 NC_004017.1 |
| Capillovirus Apple stem grooving virus | NC_001749.2 |
| Capillovirus Cherry virus A | NC_003689.1 |
| Capripoxvirus Goatpox virus | NC_004003.1 |
| Capripoxvirus Sheeppox virus | NC_004002.1 |
| Cardiovirus Encephalomyocarditis virus | NC_001479.1 |
| Cardiovirus Theilovirus | NC_001366.1 |
| Carlavirus Aconitum latent virus | NC_002795.1 |
| Carlavirus Blueberry scorch virus | NC_003499.1 |
| Carlavirus Chrysanthemum virus B | NC_009087.2 |
| Carlavirus Coleus vein necrosis virus | NC_009764.1 |
| Carlavirus Daphne virus S | NC_008020.1 |
| Carlavirus Helleborus net necrosis virus | NC_012038.1 |
| Carlavirus Hop latent virus | NC_002552.1 |
| Carlavirus Hop mosaic virus | NC_010538.1 |
| Carlavirus Kalanchoe latent virus | NC_013006.1 |
| Carlavirus Ligustrum necrotic ringspot virus | NC_010305.1 |
| Carlavirus Lily symptomless virus | NC_005138.1 |
| Carlavirus Narcissus common latent virus | NC_008266.1 |
| Carlavirus Poplar mosaic virus | NC_005343.1 |
| Carlavirus Potato latent virus | NC_011525.1 |
| Carlavirus Potato virus M | NC_001361.2 |
| Carlavirus Potato virus S | NC_007289.1 |
| Carlavirus Red clover vein mosaic virus | NC_012210.1 |
| Carmovirus Cardamine chlorotic fleck virus | NC_001600.1 |
| Carmovirus Carnation mottle virus | NC_001265.1 |
| Carmovirus Cowpea mottle virus | NC_003535.1 |
| Carmovirus Galinsoga mosaic virus | NC_001818.1 |
| Carmovirus Hibiscus chlorotic ringspot virus | NC_003608.1 |
| Carmovirus Japanese iris necrotic ring virus | NC_002187.1 |
| Carmovirus Melon necrotic spot virus | NC_001504.1 |
| Carmovirus Pea stem necrosis virus | NC_004995.1 |
| Carmovirus Pelargonium flower break virus | NC_005286.1 |
| Carmovirus Saguaro cactus virus | NC_001780.1 |
| Carmovirus Turnip crinkle virus | NC_003821.2 |
| Caulimovirus Carnation etched ring virus | NC_003498.1 |
| Caulimovirus Cauliflower mosaic virus | NC_001497.1 |
| Caulimovirus Cestrum yellow leaf curling virus | NC_004324.3 |
| Caulimovirus Figwort mosaic virus | NC_003554.1 |
| Caulimovirus Mirabilis mosaic virus | NC_004036.1 |
| Cavemovirus Cassava vein mosaic virus | NC_001648.1 |
| Cervidpoxvirus Deerpox virus W 848 83 | NC_006966.1 |
| Chelonia Chelonia mydas | NC_011531.1 |
| Cheravirus Apple latent spherical virus | NC_003787.1 NC_003788.1 |
| Cheravirus Cherry rasp leaf virus | NC_006271.1 NC_006272.1 |
| Chipapillomavirus Canine papillomavirus 3 | NC_008297.1 |
| Chlamydiamicrovirus Chlamydia phage Chp1 | NC_001741.1 |
| Chlamydiamicrovirus Chlamydia phage Chp2 | NC_002194.1 |
| Chlamydiamicrovirus Chlamydia phage phiCPG1 | NC_001998.1 |
| Chloriridovirus Invertebrate iridescent virus 3 | NC_008187.1 |
| Chlorovirus Paramecium bursaria Chlorella virus NY2A | NC_009898.1 |
| Chrysovirus Helminthosporium victoriae 145S virus | NC_005978.1 - NC_005981.1 |
| Chrysovirus Penicillium chrysogenum virus | NC_007539.1 - NC_007542.1 |
| Cilevirus Citrus leprosis virus C | NC_008169.1 NC_008170.1 |
| Circovirus Beak and feather disease virus | NC_001944.1 |
| Circovirus Canary circovirus | NC_003410.1 |
| Circovirus Goose circovirus | NC_003054.1 |
| Circovirus Porcine circovirus 1 | NC_001792.2 |
| Circovirus Porcine circovirus 2 | NC_005148.1 |
| Citrivirus Citrus leaf blotch virus | NC_003877.1 |
| Closterovirus Beet yellows virus | NC_001598.1 |
| Closterovirus Carrot yellow leaf virus | NC_013007.1 |
| Closterovirus Citrus tristeza virus | NC_001661.1 |
| Closterovirus Grapevine leafroll associated virus 2 | NC_007448.1 |
| Coccolithovirus Emiliania huxleyi virus 86 | NC_007346.1 |
| Coltivirus Colorado tick fever virus | NC_004180.1 - NC_004191.1 |
| Coltivirus Eyach virus | NC_003696.1 - NC_003707.1 |
| Comovirus Bean pod mottle virus | NC_003495.1 NC_003496.1 |
| Comovirus Cowpea mosaic virus | NC_003549.1 NC_003550.1 |
| Comovirus Cowpea severe mosaic virus | NC_003544.1 NC_003545.1 |
| Comovirus Radish mosaic virus | NC_010709.1 NC_010710.1 |
| Comovirus Red clover mottle virus | NC_003738.1 NC_003741.1 |
| Comovirus Squash mosaic virus | NC_003799.1 NC_003800.1 |
| Cosavirus Human cosavirus A | NC_012800.1 |
| Cosavirus Human cosavirus B | NC_012801.1 |
| Cosavirus Human cosavirus D | NC_012802.1 |
| Cosavirus Human cosavirus E | NC_012798.1 |
| Crinivirus Bean yellow disorder virus | NC_010560.1 NC_010561.1 |
| Crinivirus Beet pseudo yellows virus | NC_005209.2 NC_005210.2 |
| Crinivirus Blackberry yellow vein associated virus | NC_006962.2 NC_006963.2 |
| Crinivirus Cucurbit yellow stunting disorder virus | NC_004809.1 NC_004810.1 |
| Crinivirus Lettuce chlorosis virus | NC_012909.1 NC_012910.1 |
| Crinivirus Potato yellow vein virus | NC_006061.1 - NC_006063.1 |
| Crinivirus Tomato chlorosis virus | NC_007340.1 NC_007341.1 |
| Crinivirus Tomato infectious chlorosis virus | NC_013258.1 NC_013259.1 |
| Cripavirus Aphid lethal paralysis virus | NC_004365.1 |
| Cripavirus Black queen cell virus | NC_003784.1 |
| Cripavirus Cricket paralysis virus | NC_003924.1 |
| Cripavirus Drosophila C virus | NC_001834.1 |
| Cripavirus Himetobi P virus | NC_003782.1 |
| Cripavirus Homalodisca coagulata virus 1 | NC_008029.1 |
| Cripavirus Triatoma virus | NC_003783.1 |
| Cucumovirus Cucumber mosaic virus | NC_001440.1 - NC_002035.1 |
| Cucumovirus Peanut stunt virus | NC_002038.1 - NC_002040.1 |
| Cucumovirus Tomato aspermy virus | NC_003836.1 - NC_003838.1 |
| Curtovirus Beet curly top Iran virus | NC_010417.1 |
| Curtovirus Beet curly top virus | NC_001412.1 |
| Curtovirus Beet mild curly top virus | NC_004753.1 |
| Curtovirus Beet severe curly top virus | NC_004754.1 |
| Cypovirus Cypovirus 1 | NC_003016.1 - NC_003025.1 |
| Cypovirus Cypovirus 14 | NC_003006.1 - NC_003015.1 |
| Cypovirus Cypovirus 5 | NC_010661.1 - NC_010670.1 |
| Cyprinivirus Cyprinid herpesvirus 3 | NC_009127.1 |
| Cystovirus Pseudomonas phage phi12 | NC_004173.1 - NC_004175.1 |
| Cystovirus Pseudomonas phage phi13 | NC_004170.1 - NC_004172.1 |
| Cystovirus Pseudomonas phage phi6 | NC_003714.1 - NC_003716.1 |
| Cystovirus Pseudomonas phage phi8 | NC_003299.1 - NC_003301.1 |
| Cytomegalovirus Cercopithecine herpesvirus 5 | NC_012783.1 |
| Cytomegalovirus Human herpesvirus 5 | NC_006273.2 |
| Cytomegalovirus Macacine herpesvirus 3 | NC_006150.1 |
| Cytomegalovirus Panine herpesvirus 2 | NC_003521.1 |
| Cytorhabdovirus Lettuce necrotic yellows virus | NC_007642.1 |
| Cytorhabdovirus Lettuce yellow mottle virus | NC_011532.1 |
| Cytorhabdovirus Northern cereal mosaic virus | NC_002251.1 |
| Deltabaculovirus Culex nigripalpus NPV | NC_003084.1 |
| Deltalipothrixvirus Acidianus filamentous virus 2 | NC_009884.1 |
| Deltapapillomavirus Deer papillomavirus | NC_001523.1 |
| Deltapapillomavirus European elk papillomavirus | NC_001524.1 |
| Deltaretrovirus Bovine leukemia virus | NC_001414.1 |
| Deltavirus Hepatitis delta virus | NC_001653.2 |
| Densovirus Blattella germanica densovirus | NC_005041.1 |
| Densovirus Diatraea saccharalis densovirus | NC_001899.1 |
| Densovirus Junonia coenia densovirus | NC_004284.1 |
| Dependovirus Adeno associated virus 1 | NC_002077.1 |
| Dependovirus Adeno associated virus 2 | NC_001401.2 |
| Dependovirus Adeno associated virus 3 | NC_001729.1 |
| Dependovirus Adeno associated virus 4 | NC_001829.1 |
| Dependovirus Adeno associated virus 5 | NC_006152.1 |
| Dependovirus Avian adeno associated virus | NC_006263.1 |
| Dependovirus Bovine adeno associated virus | NC_005889.1 |
| Dependovirus Goose parvovirus | NC_001701.1 |
| Dianthovirus Carnation ringspot virus | NC_003530.1 NC_003531.1 |
| Dinornavirus Heterocapsa circularisquama RNA virus | NC_007518.1 |
| Dinovernavirus Aedes pseudoscutellaris reovirus | NC_007666.1 - NC_007674.1 |
| Dyoepsilonpapillomavirus Francolinus leucoscepus papillomavirus 1 | NC_013117.1 |
| Dyoiotapapillomavirus Equine papillomavirus 2 | NC_012123.1 |
| Ebolavirus Reston ebolavirus | NC_004161.1 |
| Ebolavirus Sudan ebolavirus | NC_006432.1 |
| Ebolavirus Zaire ebolavirus | NC_002549.1 |
| Emaravirus European mountain ash ringspot associated virus | NC_013105.1 - NC_013108.1 |
| Endoriftia Endoriftia persephone Hot96 1 Hot96 2 | NZ_AASF01000001.1 - NZ_AASF01002170.1 |
| Endornavirus Helicobasidium mompa endornavirus 1 | NC_013447.1 |
| Endornavirus Oryza rufipogon endornavirus | NC_007649.1 |
| Endornavirus Oryza sativa endornavirus | NC_007647.1 |
| Endornavirus Phytophthora endornavirus 1 | NC_007069.1 |
| Endornavirus Vicia faba endornavirus | NC_007648.1 |
| Enterovirus Bovine enterovirus | NC_001859.1 |
| Enterovirus Human enterovirus A | NC_001612.1 |
| Enterovirus Human enterovirus B | NC_001472.1 |
| Enterovirus Human enterovirus C | NC_001428.1 |
| Enterovirus Human enterovirus D | NC_001430.1 |
| Enterovirus Human rhinovirus A | NC_001617.1 |
| Enterovirus Porcine enterovirus B | NC_004441.1 |
| Enterovirus Simian enterovirus A | NC_003988.1 |
| Entomobirnavirus Drosophila x virus | NC_004169.1 NC_004177.1 |
| Ephemerovirus Bovine ephemeral fever virus | NC_002526.1 |
| Epsilonretrovirus Snakehead retrovirus | NC_001724.1 |
| Epsilontorquevirus Torque teno tamarin virus | NC_014085.1 |
| Equus Equus caballus | NC_003748.1 |
| Erinaceus Erinaceus europaeus | NC_011765.1 |
| Erythrovirus Human parvovirus B19 | NC_000883.1 |
| Etatorquevirus Torque teno felis virus | NC_014072.1 |
| Fabavirus Broad bean wilt virus 1 | NC_005289.1 NC_005290.1 |
| Fabavirus Broad bean wilt virus 2 | NC_003003.1 NC_003004.1 |
| Fabavirus Patchouli mild mosaic virus | NC_003974.1 NC_003975.2 |
| Flavivirus Apoi virus | NC_003676.1 |
| Flavivirus Aroa virus | NC_009026.2 |
| Flavivirus Bagaza virus | NC_012534.1 |
| Flavivirus Entebbe bat virus | NC_008718.1 |
| Flavivirus Ilheus virus | NC_009028.2 |
| Flavivirus Japanese encephalitis virus | NC_001437.1 |
| Flavivirus Kokobera virus | NC_009029.2 |
| Flavivirus Langat virus | NC_003690.1 |
| Flavivirus Louping ill virus | NC_001809.1 |
| Flavivirus Modoc virus | NC_003635.1 |
| Flavivirus Murray Valley encephalitis virus | NC_000943.1 |
| Flavivirus Omsk hemorrhagic fever virus | NC_005062.1 |
| Flavivirus Powassan virus | NC_003687.1 |
| Flavivirus Rio Bravo virus | NC_003675.1 |
| Flavivirus Sepik virus | NC_008719.1 |
| Flavivirus Usutu virus | NC_006551.1 |
| Flavivirus Wesselsbron virus | NC_012735.1 |
| Flavivirus West Nile virus | NC_001563.2 |
| Flavivirus Yellow fever virus | NC_002031.1 |
| Flavivirus Yokose virus | NC_005039.1 |
| Foveavirus Apple stem pitting virus | NC_003462.1 |
| Foveavirus Peach chlorotic mottle virus | NC_009892.1 |
| Furovirus Chinese wheat mosaic virus | NC_002356.1 NC_002359.1 |
| Furovirus Oat golden stripe virus | NC_002357.1 NC_002358.1 |
| Fusellovirus Sulfolobus virus 1 | NC_001338.1 |
| Gammabaculovirus Neodiprion abietis NPV | NC_008252.1 |
| Gammabaculovirus Neodiprion lecontii NPV | NC_005906.1 |
| Gammabaculovirus Neodiprion sertifer NPV | NC_005905.1 |
| Gammalipothrixvirus Acidianus filamentous virus 1 | NC_005830.1 |
| Gammatorquevirus Torque teno midi virus 1 | NC_009225.1 |
| Gammatorquevirus Torque teno midi virus 2 | NC_014093.1 |
| Giardiavirus Giardia lamblia virus | NC_003555.1 |
| Gyrovirus Chicken anemia virus | NC_001427.1 |
| Hantavirus Andes virus | NC_003466.1 - NC_003468.2 |
| Hantavirus Dobrava Belgrade virus | NC_005233.1 - NC_005235.1 |
| Hantavirus Hantaan virus | NC_005218.1 - NC_005222.1 |
| Hantavirus Puumala virus | NC_005223.1 NC_005224.1 |
| Hantavirus Seoul virus | NC_005236.1 - NC_005238.1 |
| Hantavirus Sin Nombre virus | NC_005215.1 - NC_005217.1 |
| Hantavirus Thottapalayam virus | NC_010704.1 - NC_010708.1 |
| Hantavirus Tula virus | NC_005226.1 - NC_005228.1 |
| Heliothis Heliothis virescens | NC_009233.1 |
| Henipavirus Hendra virus | NC_001906.2 |
| Henipavirus Nipah virus | NC_002728.1 |
| Hepacivirus Hepatitis C virus | NC_004102.1 |
| Hepatovirus Hepatitis A virus | NC_001489.1 |
| Hepevirus Hepatitis E virus | NC_001434.1 |
| Hordeivirus Barley stripe mosaic virus | NC_003469.1 - NC_003481.1 |
| Hypovirus Cryphonectria hypovirus 1 | NC_001492.1 |
| Hypovirus Cryphonectria hypovirus 2 | NC_003534.1 |
| Hypovirus Cryphonectria hypovirus 3 | NC_000960.1 |
| Hypovirus Cryphonectria hypovirus 4 | NC_006431.1 |
| Ichnovirus Campoletis sonorensis ichnovirus | NC_007985.1 - NC_008008.1 |
| Ichnovirus Glypta fumiferanae ichnovirus | NC_008837.1 - NC_008941.1 |
| Ichnovirus Hyposoter fugitivus ichnovirus | NC_008946.1 - NC_009003.1 |
| Ictalurivirus Ictalurid herpesvirus 1 | NC_001493.1 |
| Iflavirus Perina nuda virus | NC_003113.1 |
| Iflavirus Sacbrood virus | NC_002066.1 |
| Iltovirus Gallid herpesvirus 1 | NC_006623.1 |
| Iltovirus Psittacid herpesvirus 1 | NC_005264.1 |
| Influenzavirus B Influenza B virus | NC_002204.1 - NC_002211.1 |
| Inovirus Enterobacteria phage I2 2 | NC_001332.1 |
| Inovirus Enterobacteria phage Ike | NC_002014.1 |
| Inovirus Enterobacteria phage M13 | NC_003287.2 |
| Inovirus Pseudomonas phage Pf1 | NC_001331.1 |
| Inovirus Pseudomonas phage Pf3 | NC_001418.1 |
| Inovirus Vibrio phage KSF 1phi | NC_006294.1 |
| Inovirus Vibrio phage VGJphi | NC_004736.1 |
| Inovirus Vibrio phage VSK | NC_003327.2 |
| Inovirus Vibrio phage Vf12 | NC_005949.1 |
| Inovirus Vibrio phage Vf33 | NC_005948.1 |
| Inovirus Vibrio phage fs1 | NC_004306.1 |
| Inovirus Vibrio phage fs2 | NC_001956.1 |
| Inovirus Xanthomonas phage Cf1c | NC_001396.1 |
| Iotatorquevirus Torque teno sus virus 1 | NC_014070.1 |
| Iotatorquevirus Torque teno sus virus 2 | NC_014092.2 |
| Ipomovirus Cassava brown streak virus | NC_012698.1 |
| Ipomovirus Cucumber vein yellowing virus | NC_006941.1 |
| Isavirus Infectious salmon anemia virus | NC_006497.1 - NC_006505.1 |
| Iteravirus Bombyx mori densovirus | NC_004287.1 |
| Kappapapillomavirus Cottontail rabbit papillomavirus | NC_001541.1 |
| Kobuvirus Aichi virus | NC_001918.1 |
| Kobuvirus Bovine kobuvirus | NC_004421.1 |
| L5-like viruses Mycobacterium phage D29 | NC_001900.1 |
| L5-like viruses Mycobacterium phage L5 | NC_001335.1 |
| LUZ24-like viruses Pseudomonas phage LUZ24 | NC_010325.1 |
| LUZ24-like viruses Pseudomonas phage PaP3 | NC_004466.2 |
| Lagovirus European brown hare syndrome virus | NC_002615.1 |
| Lagovirus Rabbit hemorrhagic disease virus | NC_001543.1 |
| Lambda-like viruses Enterobacteria phage HK022 | NC_002166.1 |
| Lambda-like viruses Enterobacteria phage HK97 | NC_002167.1 |
| Lambdapapillomavirus Canine oral papillomavirus | NC_001619.1 |
| Lambdapapillomavirus Canine papillomavirus 6 | NC_013237.1 |
| Leporipoxvirus Myxoma virus | NC_001132.2 |
| Leporipoxvirus Rabbit fibroma virus | NC_001266.1 |
| Levivirus Enterobacteria phage BZ13 | NC_001426.1 |
| Levivirus Enterobacteria phage MS2 | NC_001417.2 |
| Lolavirus Lolium latent virus | NC_010434.1 |
| Luteovirus Barley yellow dwarf virus MAV | NC_003680.1 |
| Luteovirus Barley yellow dwarf virus PAS | NC_002160.2 |
| Luteovirus Barley yellow dwarf virus PAV | NC_004750.1 |
| Luteovirus Bean leafroll virus | NC_003369.1 |
| Luteovirus Rose spring dwarf associated virus | NC_010806.1 |
| Luteovirus Soybean dwarf virus | NC_003056.1 |
| Lymphocryptovirus Callitrichine herpesvirus 3 | NC_004367.1 |
| Lymphocryptovirus Human herpesvirus 4 | NC_009334.1 NC_007605.1 |
| Lymphocryptovirus Macacine herpesvirus 4 | NC_006146.1 |
| Lyssavirus Australian bat lyssavirus | NC_003243.1 |
| Lyssavirus European bat lyssavirus 1 | NC_009527.1 |
| Lyssavirus European bat lyssavirus 2 | NC_009528.1 |
| Lyssavirus Mokola virus | NC_006429.1 |
| Lyssavirus Rabies virus | NC_001542.1 |
| Macavirus Alcelaphine herpesvirus 1 | NC_002531.1 |
| Macavirus Ovine herpesvirus 2 | NC_007646.1 |
| Machlomovirus Maize chlorotic mottle virus | NC_003627.1 |
| Maculavirus Grapevine fleck virus | NC_003347.1 |
| Mamastrovirus Human astrovirus | NC_001943.1 |
| Mamastrovirus Mink astrovirus | NC_004579.1 |
| Mamastrovirus Ovine astrovirus | NC_002469.1 |
| Marafivirus Maize rayado fino virus | NC_002786.1 |
| Marafivirus Oat blue dwarf virus | NC_001793.1 |
| Marburgvirus Lake Victoria marburgvirus | NC_001608.3 |
| Mardivirus Gallid herpesvirus 2 | NC_002229.3 |
| Mardivirus Gallid herpesvirus 3 | NC_002577.1 |
| Mardivirus Meleagrid herpesvirus 1 | NC_002641.1 |
| Marnavirus Heterosigma akashiwo RNA virus | NC_005281.1 |
| Mastadenovirus Bovine adenovirus A | NC_006324.1 |
| Mastadenovirus Bovine adenovirus B | NC_001876.1 |
| Mastadenovirus Canine adenovirus | NC_001734.1 |
| Mastadenovirus Human adenovirus A | NC_001460.1 |
| Mastadenovirus Human adenovirus B | NC_011202.1 |
| Mastadenovirus Human adenovirus C | NC_001405.1 |
| Mastadenovirus Human adenovirus D | NC_010956.1 |
| Mastadenovirus Human adenovirus E | NC_003266.2 |
| Mastadenovirus Human adenovirus F | NC_001454.1 |
| Mastadenovirus Murine adenovirus A | NC_000942.1 |
| Mastadenovirus Ovine adenovirus A | NC_002513.1 |
| Mastadenovirus Porcine adenovirus A | NC_005869.1 |
| Mastadenovirus Porcine adenovirus C | NC_002702.1 |
| Mastadenovirus Tree shrew adenovirus | NC_004453.1 |
| Mastrevirus Bean yellow dwarf virus | NC_003493.2 |
| Mastrevirus Chloris striate mosaic virus | NC_001466.1 |
| Mastrevirus Digitaria streak virus | NC_001478.1 |
| Mastrevirus Eragrostis streak virus | NC_010352.1 |
| Mastrevirus Maize streak virus | NC_001346.1 |
| Mastrevirus Miscanthus streak virus | NC_003379.1 |
| Mastrevirus Panicum streak virus | NC_001647.1 |
| Mastrevirus Sugarcane streak virus | NC_003744.1 |
| Mastrevirus Urochloa streak virus | NC_010797.1 |
| Mastrevirus Wheat dwarf virus | NC_003326.1 |
| Megasphaera Megasphaera genomosp type 1 28L | NZ_ADGP01000001.1 - NZ_ADGP01000034.1 |
| Metapneumovirus Avian metapneumovirus | NC_007652.1 |
| Metapneumovirus Human metapneumovirus | NC_004148.2 |
| Microvirus Enterobacteria phage St 1 | NC_012868.1 |
| Mimoreovirus Micromonas pusilla reovirus | NC_008171.1 - NC_008181.1 |
| Mitovirus Ophiostoma mitovirus 3a | NC_004049.1 |
| Mitovirus Ophiostoma mitovirus 4 | NC_004052.1 |
| Mitovirus Ophiostoma mitovirus 5 | NC_004053.1 |
| Mitovirus Ophiostoma mitovirus 6 | NC_004054.1 |
| Morbillivirus Canine distemper virus | NC_001921.1 |
| Morbillivirus Measles virus | NC_001498.1 |
| Morbillivirus Peste des petits ruminants virus | NC_006383.2 |
| Mu-like viruses Burkholderia phage BcepMu | NC_005882.1 |
| Mu-like viruses Enterobacteria phage Mu | NC_000929.1 |
| Muromegalovirus Murid herpesvirus 1 | NC_004065.1 |
| Muromegalovirus Murid herpesvirus 2 | NC_002512.2 |
| Mus Mus musculus | NC_014326.1 |
| Mycoflexivirus Botrytis virus F | NC_002604.1 |
| Mycoreovirus Mycoreovirus 1 | NC_010743.1 - NC_010753.1 |
| Mycoreovirus Mycoreovirus 3 | NC_007524.1 - NC_007536.1 |
| N4-like viruses Enterobacteria phage N4 | NC_008720.1 |
| Nairovirus Crimean Congo hemorrhagic fever virus | NC_005300.2 - NC_005302.1 |
| Nairovirus Dugbe virus | NC_004157.1 - NC_004159.1 |
| Nanovirus Faba bean necrotic stunt virus | NC_013094.1 - NC_013101.1 |
| Nanovirus Milk vetch dwarf virus | NC_003638.1 - NC_003648.1 |
| Necrovirus Beet black scorch virus | NC_004452.3 |
| Necrovirus Leek white stripe virus | NC_001822.1 |
| Necrovirus Olive latent virus 1 | NC_001721.1 |
| Norovirus Norwalk virus | NC_001959.2 |
| Novirhabdovirus Hirame rhabdovirus | NC_005093.1 |
| Novirhabdovirus Snakehead rhabdovirus | NC_000903.1 |
| Nucleorhabdovirus Maize fine streak virus | NC_005974.1 |
| Nucleorhabdovirus Maize mosaic virus | NC_005975.1 |
| Nucleorhabdovirus Rice yellow stunt virus | NC_003746.1 |
| Okavirus Gill associated virus | NC_010306.1 |
| Oleavirus Olive latent virus 2 | NC_003671.1 - NC_003674.1 |
| Omikronpapillomavirus Phocoena spinipinnis papillomavirus | NC_003348.1 |
| Ophiovirus Citrus psorosis virus | NC_006314.1 - NC_006316.1 |
| Ophiovirus Lettuce ring necrosis virus | NC_006051.1 - NC_006054.1 |
| Orbivirus African horsesickness virus | NC_005996.1 - NC_006021.1 |
| Orbivirus Bluetongue virus | NC_006007.1 - NC_006025.1 |
| Orbivirus Palyam virus | NC_005986.1 - NC_005995.1 |
| Orbivirus Yunnan orbivirus | NC_007656.1 - NC_007665.1 |
| Orthobunyavirus Akabane virus | NC_009894.1 - NC_009896.1 |
| Orthobunyavirus Bunyamwera virus | NC_001925.1 - NC_001927.1 |
| Orthobunyavirus Oropouche virus | NC_005775.1 - NC_005777.1 |
| Orthohepadnavirus Hepatitis B virus | NC_003977.1 |
| Orthopoxvirus Camelpox virus | NC_003391.1 |
| Orthopoxvirus Cowpox virus | NC_003663.2 |
| Orthopoxvirus Ectromelia virus | NC_004105.1 |
| Orthopoxvirus Monkeypox virus | NC_003310.1 |
| Orthopoxvirus Taterapox virus | NC_008291.1 |
| Orthopoxvirus Vaccinia virus | NC_006998.1 |
| Orthopoxvirus Variola virus | NC_001611.1 |
| Orthoturdivirus Turdivirus 1 | NC_014411.1 |
| Oryzavirus Rice ragged stunt virus | NC_003749.1 - NC_003771.1 |
| Ostreavirus Ostreid herpesvirus 1 | NC_005881.1 |
| Ourmiavirus Cassava virus C | NC_013111.1 - NC_013113.1 |
| Ourmiavirus Epirus cherry virus | NC_011065.1 - NC_011067.1 |
| Ourmiavirus Ourmia melon virus | NC_011068.1 - NC_011070.1 |
| P1-like viruses Enterobacteria phage P1 | NC_005856.1 |
| P2-like viruses Aeromonas phage phiO18P | NC_009542.2 |
| P2-like viruses Enterobacteria phage P2 | NC_001895.1 |
| P2-like viruses Haemophilus phage HP1 | NC_001697.1 |
| P22-like viruses Enterobacteria phage P22 | NC_002371.2 |
| P22-like viruses Enterobacteria phage ST104 | NC_005841.1 |
| Panicovirus Panicum mosaic virus | NC_002598.1 |
| Paramecium Paramecium bursaria | NC_008603.1 |
| Parapoxvirus Bovine papular stomatitis virus | NC_005337.1 |
| Parapoxvirus Orf virus | NC_005336.1 |
| Parapoxvirus Pseudocowpox virus | NC_013804.1 |
| Paraturdivirus Turdivirus 2 | NC_014412.1 |
| Paraturdivirus Turdivirus 3 | NC_014413.1 |
| Parechovirus Human parechovirus | NC_001897.1 |
| Parechovirus Ljungan virus | NC_003976.2 |
| Partitivirus Atkinsonella hypoxylon virus | NC_003470.1 NC_003471.1 |
| Partitivirus Discula destructiva virus 1 | NC_002797.1 NC_002800.1 |
| Partitivirus Discula destructiva virus 2 | NC_003710.1 NC_003711.1 |
| Partitivirus Fusarium poae virus 1 | NC_003883.1 NC_003884.1 |
| Partitivirus Penicillium stoloniferum virus F | NC_007221.1 NC_007222.1 |
| Partitivirus Penicillium stoloniferum virus S | NC_005976.2 NC_005977.2 |
| Parvovirus H 1 parvovirus | NC_001358.1 |
| Parvovirus LuIII virus | NC_004713.1 |
| Parvovirus Minute virus of mice | NC_001510.1 |
| Parvovirus Mouse parvovirus 1 | NC_001630.1 |
| Parvovirus Porcine parvovirus | NC_001718.1 |
| Pecluvirus Indian peanut clump virus | NC_004729.1 NC_004730.1 |
| Pecluvirus Peanut clump virus | NC_003668.1 NC_003672.1 |
| Pestivirus Border disease virus | NC_003679.1 |
| Pestivirus Bovine viral diarrhea virus 1 | NC_001461.1 |
| Pestivirus Bovine viral diarrhea virus 2 | NC_002032.1 |
| Pestivirus Classical swine fever virus | NC_002657.1 |
| Phaeovirus Ectocarpus siliculosus virus 1 | NC_002687.1 |
| Phaeovirus Feldmannia species virus | NC_011183.1 |
| Phi29-like viruses Bacillus phage B103 | NC_004165.1 |
| Phi29-like viruses Bacillus phage GA 1 | NC_002649.1 |
| Phi29-like viruses Bacillus phage phi29 | NC_011048.1 |
| Phipapillomavirus Capra hircus papillomavirus type 1 | NC_008032.1 |
| Phlebovirus Rift Valley fever virus | NC_014395.1 - NC_014397.1 |
| Phlebovirus Sandfly fever Naples virus | NC_006318.1 - NC_006320.1 |
| Phlebovirus Uukuniemi virus | NC_005214.1 - NC_005221.1 |
| Phytoreovirus Rice dwarf virus | NC_003760.1 - NC_003774.1 |
| Phytoreovirus Rice gall dwarf virus | NC_009241.1 - NC_009252.1 |
| Picobirnavirus Human picobirnavirus | NC_007026.1 NC_007027.1 |
| Pipapillomavirus Mastomys coucha papillomavirus 2 | NC_008519.1 |
| Plasmavirus Acholeplasma phage L2 | NC_001447.1 |
| Plectrovirus Spiroplasma phage 1 C74 | NC_003793.1 |
| Pneumovirus Bovine respiratory syncytial virus | NC_001989.1 |
| Pneumovirus Human respiratory syncytial virus | NC_001781.1 |
| Polemovirus Poinsettia cryptic virus | NC_011543.1 |
| Polerovirus Beet chlorosis virus | NC_002766.1 |
| Polerovirus Beet mild yellowing virus | NC_003491.1 |
| Polerovirus Beet western yellows virus | NC_004756.1 |
| Polerovirus Carrot red leaf virus | NC_006265.1 |
| Polerovirus Cereal yellow dwarf virus RPS | NC_002198.2 |
| Polerovirus Cereal yellow dwarf virus RPV | NC_004751.1 |
| Polerovirus Chickpea chlorotic stunt virus | NC_008249.1 |
| Polerovirus Cucurbit aphid borne yellows virus | NC_003688.1 |
| Polerovirus Melon aphid borne yellows virus | NC_010809.1 |
| Polerovirus Potato leafroll virus | NC_001747.1 |
| Polerovirus Tobacco vein distorting virus | NC_010732.1 |
| Polerovirus Turnip yellows virus | NC_003743.1 |
| Polyomavirus African green monkey polyomavirus | NC_004763.1 |
| Polyomavirus BK polyomavirus | NC_001538.1 |
| Polyomavirus Bovine polyomavirus | NC_001442.1 |
| Polyomavirus Hamster polyomavirus | NC_001663.1 |
| Polyomavirus JC polyomavirus | NC_001699.1 |
| Polyomavirus Murine polyomavirus | NC_001515.1 |
| Polyomavirus Simian virus 12 | NC_012122.1 |
| Polyomavirus Simian virus 40 | NC_001669.1 |
| Pomovirus Beet soil borne virus | NC_003518.1 - NC_003520.1 |
| Pomovirus Beet virus Q | NC_003510.1 - NC_003512.1 |
| Pomovirus Broad bean necrosis virus | NC_004423.1 - NC_004425.1 |
| Pomovirus Potato mop top virus | NC_003723.1 - NC_003725.1 |
| Potexvirus Alternanthera mosaic virus | NC_007731.1 |
| Potexvirus Bamboo mosaic virus | NC_001642.1 |
| Potexvirus Cactus virus X | NC_002815.2 |
| Potexvirus Cassava common mosaic virus | NC_001658.1 |
| Potexvirus Clover yellow mosaic virus | NC_001753.1 |
| Potexvirus Cymbidium mosaic virus | NC_001812.1 |
| Potexvirus Foxtail mosaic virus | NC_001483.1 |
| Potexvirus Hosta virus X | NC_011544.1 |
| Potexvirus Hydrangea ringspot virus | NC_006943.1 |
| Potexvirus Lettuce virus X | NC_010832.1 |
| Potexvirus Lily virus X | NC_007192.1 |
| Potexvirus Narcissus mosaic virus | NC_001441.1 |
| Potexvirus Nerine virus X | NC_007679.1 |
| Potexvirus Papaya mosaic virus | NC_001748.1 |
| Potexvirus Pepino mosaic virus | NC_004067.1 |
| Potexvirus Phaius virus X | NC_010295.1 |
| Potexvirus Plantago asiatica mosaic virus | NC_003849.1 |
| Potexvirus Potato virus X | NC_011620.1 |
| Potexvirus Scallion virus X | NC_003400.1 |
| Potexvirus Tulip virus X | NC_004322.1 |
| Potyvirus Algerian watermelon mosaic virus | NC_010736.1 |
| Potyvirus Banana bract mosaic virus | NC_009745.1 |
| Potyvirus Bean common mosaic necrosis virus | NC_004047.1 |
| Potyvirus Bean common mosaic virus | NC_003397.1 |
| Potyvirus Bean yellow mosaic virus | NC_003492.1 |
| Potyvirus Beet mosaic virus | NC_005304.1 |
| Potyvirus Bidens mottle virus | NC_014325.1 |
| Potyvirus Canna Yellow Streak Virus | NC_013261.1 |
| Potyvirus Chilli veinal mottle virus | NC_005778.1 |
| Potyvirus Clover yellow vein virus | NC_003536.1 |
| Potyvirus Cocksfoot streak virus | NC_003742.1 |
| Potyvirus Cowpea aphid borne mosaic virus | NC_004013.1 |
| Potyvirus Daphne mosaic virus | NC_008028.1 |
| Potyvirus Dasheen mosaic virus | NC_003537.1 |
| Potyvirus Freesia mosaic virus | NC_014064.1 |
| Potyvirus Japanese yam mosaic virus | NC_000947.1 |
| Potyvirus Johnsongrass mosaic virus | NC_003606.1 |
| Potyvirus Konjac mosaic virus | NC_007913.1 |
| Potyvirus Leek yellow stripe virus | NC_004011.1 |
| Potyvirus Lettuce mosaic virus | NC_003605.1 |
| Potyvirus Lily mottle virus | NC_005288.1 |
| Potyvirus Maize dwarf mosaic virus | NC_003377.1 |
| Potyvirus Moroccan watermelon mosaic virus | NC_009995.1 |
| Potyvirus Narcissus degeneration virus | NC_008824.1 |
| Potyvirus Narcissus yellow stripe virus | NC_011541.1 |
| Potyvirus Onion yellow dwarf virus | NC_005029.1 |
| Potyvirus Papaya leaf distortion mosaic virus | NC_005028.1 |
| Potyvirus Papaya ringspot virus | NC_001785.1 |
| Potyvirus Pea seed borne mosaic virus | NC_001671.1 |
| Potyvirus Peanut mottle virus | NC_002600.1 |
| Potyvirus Pepper mottle virus | NC_001517.1 |
| Potyvirus Pepper severe mosaic virus | NC_008393.1 |
| Potyvirus Pepper veinal mottle virus | NC_011918.1 |
| Potyvirus Pepper yellow mosaic virus | NC_014327.1 |
| Potyvirus Peru tomato mosaic virus | NC_004573.1 |
| Potyvirus Plum pox virus | NC_001445.1 |
| Potyvirus Potato virus A | NC_004039.1 |
| Potyvirus Potato virus V | NC_004010.1 |
| Potyvirus Potato virus Y | NC_001616.1 |
| Potyvirus Scallion mosaic virus | NC_003399.1 |
| Potyvirus Shallot yellow stripe virus | NC_007433.1 |
| Potyvirus Sorghum mosaic virus | NC_004035.1 |
| Potyvirus Soybean mosaic virus | NC_002634.1 |
| Potyvirus Sugarcane mosaic virus | NC_003398.1 |
| Potyvirus Sunflower chlorotic mottle virus | NC_014038.1 |
| Potyvirus Telosma mosaic virus | NC_009742.1 |
| Potyvirus Tobacco etch virus | NC_001555.1 |
| Potyvirus Tobacco vein banding mosaic virus | NC_009994.1 |
| Potyvirus Turnip mosaic virus | NC_002509.2 |
| Potyvirus Watermelon mosaic virus | NC_006262.1 |
| Potyvirus Wild tomato mosaic virus | NC_009744.1 |
| Potyvirus Wisteria vein mosaic virus | NC_007216.1 |
| Potyvirus Yam mosaic virus | NC_004752.1 |
| Potyvirus Zantedeschia mild mosaic virus | NC_011560.1 |
| Ranavirus Ambystoma tigrinum virus | NC_005832.1 |
| Ranavirus Frog virus 3 | NC_005946.1 |
| Respirovirus Bovine parainfluenza virus 3 | NC_002161.1 |
| Respirovirus Sendai virus | NC_001552.1 |
| Rhadinovirus Ateline herpesvirus 3 | NC_001987.1 |
| Rhadinovirus Bovine herpesvirus 4 | NC_002665.1 |
| Rhadinovirus Human herpesvirus 8 | NC_009333.1 |
| Rhadinovirus Macacine herpesvirus 5 | NC_003401.1 |
| Rhadinovirus Murid herpesvirus 4 | NC_001826.2 |
| Roseolovirus Human herpesvirus 6 | NC_000898.1 NC_001664.2 |
| Roseolovirus Human herpesvirus 7 | NC_001716.2 |
| Rotavirus Rotavirus A | NC_011500.1 - NC_011510.1 |
| Rotavirus Rotavirus C | NC_007543.1 - NC_007574.1 |
| Rousettus Rousettus aegyptiacus | NC_008298.1 |
| Rubivirus Rubella virus | NC_001545.1 |
| Rubulavirus Human parainfluenza virus 2 | NC_003443.1 |
| Rubulavirus Mapuera virus | NC_009489.1 |
| Rubulavirus Mumps virus | NC_002200.1 |
| Rubulavirus Parainfluenza virus 5 | NC_006430.1 |
| Rubulavirus Porcine rubulavirus | NC_009640.1 |
| Rubulavirus Simian virus 41 | NC_006428.1 |
| Rymovirus Agropyron mosaic virus | NC_005903.1 |
| Rymovirus Hordeum mosaic virus | NC_005904.1 |
| Rymovirus Ryegrass mosaic virus | NC_001814.1 |
| SP6-like viruses Enterobacteria phage K1E | NC_007637.1 |
| SP6-like viruses Enterobacteria phage K1 5 | NC_008152.1 |
| SPO1-like viruses Bacillus phage SPO1 | NC_011421.1 |
| Sadwavirus Satsuma dwarf virus | NC_003785.2 NC_003786.2 |
| Salterprovirus His1 virus | NC_007914.1 |
| Salterprovirus His2 virus | NC_007918.1 |
| Sclerodarnavirus Sclerotinia sclerotiorum debilitation associated RNA virus | NC_007415.1 |
| Seadornavirus Banna virus | NC_004198.1 - NC_004221.1 |
| Seadornavirus Kadipiro virus | NC_004199.1 - NC_004216.1 |
| Seadornavirus Liao ning virus | NC_007736.1 - NC_007747.1 |
| Senecavirus Seneca valley virus | NC_011349.1 |
| Sequivirus Parsnip yellow fleck virus | NC_003628.1 |
| Siadenovirus Frog adenovirus | NC_002501.1 |
| Siadenovirus Turkey adenovirus A | NC_001958.1 |
| Sigmapapillomavirus Erethizon dorsatum papillomavirus type 1 | NC_006951.1 |
| Simplexvirus Cercopithecine herpesvirus 2 | NC_006560.1 |
| Simplexvirus Human herpesvirus 1 | NC_001806.1 |
| Simplexvirus Human herpesvirus 2 | NC_001798.1 |
| Simplexvirus Macacine herpesvirus 1 | NC_004812.1 |
| Simplexvirus Papiine herpesvirus 2 | NC_007653.1 |
| Sobemovirus Cocksfoot mild mosaic virus | NC_011108.1 |
| Sobemovirus Cocksfoot mottle virus | NC_002618.2 |
| Sobemovirus Lucerne transient streak virus | NC_001696.1 |
| Sobemovirus Rice yellow mottle virus | NC_001575.1 |
| Sobemovirus Ryegrass mottle virus | NC_003747.1 |
| Sobemovirus Sesbania mosaic virus | NC_002568.2 |
| Sobemovirus Southern bean mosaic virus | NC_004060.1 |
| Sobemovirus Southern cowpea mosaic virus | NC_001625.1 |
| Sobemovirus Turnip rosette virus | NC_004553.1 |
| Soymovirus Blueberry red ringspot virus | NC_003138.2 |
| Spiromicrovirus Spiroplasma phage 4 | NC_003438.1 |
| Spiroplasma Spiroplasma kunkelii | NC_009987.1 |
| Spumavirus African green monkey simian foamy virus | NC_010820.1 |
| Spumavirus Bovine foamy virus | NC_001831.1 |
| Spumavirus Equine foamy virus | NC_002201.1 |
| Spumavirus Feline foamy virus | NC_001871.1 |
| Spumavirus Macaque simian foamy virus | NC_010819.1 |
| Spumavirus Simian foamy virus | NC_001364.1 |
| Suipoxvirus Swinepox virus | NC_003389.1 |
| Sus Sus scrofa | NC_011280.1 |
| T1-like viruses Enterobacteria phage T1 | NC_005833.1 |
| T4-like viruses Aeromonas phage Aeh1 | NC_005260.1 |
| T5-like viruses Enterobacteria phage T5 | NC_005859.1 |
| T7-like viruses Enterobacteria phage K1F | NC_007456.1 |
| T7-like viruses Enterobacteria phage T3 | NC_003298.1 |
| T7-like viruses Enterobacteria phage T7 | NC_001604.1 |
| T7-like viruses Kluyvera phage Kvp1 | NC_011534.1 |
| T7-like viruses Pseudomonas phage gh 1 | NC_004665.1 |
| T7-like viruses Salmonella phage phiSG JL2 | NC_010807.1 |
| T7-like viruses Vibrio phage N4 | NC_013651.1 |
| T7-like viruses Vibriophage VP4 | NC_007149.1 |
| T7-like viruses Yersinia phage Berlin | NC_008694.1 |
| Taupapillomavirus Canine papillomavirus 2 | NC_006564.1 |
| Tenuivirus Rice grassy stunt virus | NC_002323.1 - NC_002328.1 |
| Tenuivirus Rice stripe virus | NC_003753.1 - NC_003776.1 |
| Teschovirus Porcine teschovirus | NC_003985.1 |
| Thetatorquevirus Torque teno canis virus | NC_014071.1 |
| Thogotovirus Thogoto virus | NC_006495.1 - NC_006508.1 |
| Tobamovirus Cucumber fruit mottle mosaic virus | NC_002633.1 |
| Tobamovirus Cucumber green mottle mosaic virus | NC_001801.1 |
| Tobamovirus Hibiscus latent Singapore virus | NC_008310.1 |
| Tobamovirus Kyuri green mottle mosaic virus | NC_003610.1 |
| Tobamovirus Obuda pepper virus | NC_003852.1 |
| Tobamovirus Odontoglossum ringspot virus | NC_001728.1 |
| Tobamovirus Paprika mild mottle virus | NC_004106.1 |
| Tobamovirus Pepper mild mottle virus | NC_003630.1 |
| Tobamovirus Rehmannia mosaic virus | NC_009041.1 |
| Tobamovirus Ribgrass mosaic virus | NC_002792.1 |
| Tobamovirus Streptocarpus flower break virus | NC_008365.1 |
| Tobamovirus Tobacco mosaic virus | NC_001367.1 |
| Tobamovirus Tomato mosaic virus | NC_002692.1 |
| Tobamovirus Youcai mosaic virus | NC_004422.1 |
| Tobravirus Pea early browning virus | NC_001368.1 NC_002036.1 |
| Tobravirus Pepper ringspot virus | NC_003669.1 NC_003670.1 |
| Tobravirus Tobacco rattle virus | NC_003805.1 NC_003811.1 |
| Tombusvirus Artichoke mottled crinkle virus | NC_001339.1 |
| Tombusvirus Carnation Italian ringspot virus | NC_003500.2 |
| Tombusvirus Cucumber Bulgarian latent virus | NC_004725.1 |
| Tombusvirus Cucumber necrosis virus | NC_001469.1 |
| Tombusvirus Cymbidium ringspot virus | NC_003532.1 |
| Tombusvirus Grapevine Algerian latent virus | NC_011535.1 |
| Tombusvirus Pear latent virus | NC_004723.1 |
| Torradovirus Tomato marchitez virus | NC_010987.1 NC_010988.1 |
| Torradovirus Tomato torrado virus | NC_009013.1 NC_009032.1 |
| Tremovirus Avian encephalomyelitis virus | NC_003990.1 |
| Trichovirus Apple chlorotic leaf spot virus | NC_001409.1 |
| Trichovirus Cherry mottle leaf virus | NC_002500.1 |
| Trichovirus Peach mosaic virus | NC_011552.1 |
| Tritimovirus Brome streak mosaic virus | NC_003501.1 |
| Tritimovirus Oat necrotic mottle virus | NC_005136.1 |
| Tursiops Tursiops truncatus | NC_011109.1 - NC_011110.1 |
| Tymovirus Anagyris vein yellowing virus | NC_011559.1 |
| Tymovirus Chayote mosaic virus | NC_002588.1 |
| Tymovirus Diascia yellow mottle virus | NC_011086.1 |
| Tymovirus Dulcamara mottle virus | NC_007609.1 |
| Tymovirus Eggplant mosaic virus | NC_001480.1 |
| Tymovirus Erysimum latent virus | NC_001977.1 |
| Tymovirus Okra mosaic virus | NC_009532.1 |
| Tymovirus Physalis mottle virus | NC_003634.1 |
| Tymovirus Plantago mottle virus | NC_011539.1 |
| Tymovirus Scrophularia mottle virus | NC_011537.1 |
| Umbravirus Carrot mottle mimic virus | NC_001726.1 |
| Umbravirus Groundnut rosette virus | NC_003603.1 |
| Umbravirus Tobacco bushy top virus | NC_004366.1 |
| Ursus Ursus maritimus | NC_010739.1 |
| VP2-like phages Vibrio phage VP2 | NC_005879.1 |
| VP2-like phages Vibrio phage VP5 | NC_005891.1 |
| Varicellovirus Bovine herpesvirus 1 | NC_001847.1 |
| Varicellovirus Bovine herpesvirus 5 | NC_005261.2 |
| Varicellovirus Cercopithecine herpesvirus 9 | NC_002686.2 |
| Varicellovirus Felid herpesvirus 1 | NC_013590.2 |
| Varicellovirus Human herpesvirus 3 | NC_001348.1 |
| Varicosavirus Lettuce big vein associated virus | NC_011558.1 NC_011568.1 |
| Vesivirus Feline calicivirus | NC_001481.2 |
| Victorivirus Chalara elegans RNA Virus 1 | NC_005883.1 |
| Victorivirus Magnaporthe oryzae virus 1 | NC_006367.1 |
| Vitivirus Grapevine virus A | NC_003604.2 |
| Vitivirus Grapevine virus B | NC_003602.1 |
| Vitivirus Grapevine virus E | NC_011106.1 |
| Waikavirus Maize chlorotic dwarf virus | NC_003626.1 |
| Waikavirus Rice tungro spherical virus | NC_001632.1 |
| Xipapillomavirus Bovine papillomavirus 3 | NC_004197.1 |
| Yatapoxvirus Tanapox virus | NC_009888.1 |
| Yatapoxvirus Yaba monkey tumor virus | NC_005179.1 |
| Zetatorquevirus Torque teno douroucouli virus | NC_014087.1 |
| Abalone shriveling syndrome associated virus | NC_011646.1 |
| Abelson murine leukemia virus | NC_001499.1 |
| Abutilon Brazil virus | NC_014138.1 NC_014139.1 |
| Acanthocystis turfacea Chlorella virus 1 | NC_008724.1 |
| Acheta domestica densovirus | NC_004290.1 |
| Acholeplasma phage MV L1 | NC_001341.1 |
| Acidianus rod shaped virus 1 | NC_009965.1 |
| Acidianus spindle shaped virus 1 | NC_013585.1 |
| Acinetobacter phage AP205 | NC_002700.2 |
| Actinomyces phage Av 1 | NC_009643.1 |
| Actinoplanes phage phiAsp2 | NC_005885.1 |
| Acute bee paralysis virus | NC_002548.1 |
| Acyrthosiphon pisum virus | NC_003780.1 |
| Adeno associated virus 7 | NC_006260.1 |
| Adeno associated virus 8 | NC_006261.1 |
| Adoxophyes orana nucleopolyhedrovirus | NC_011423.1 |
| Aedes flavivirus | NC_012932.1 |
| Aeromonas phage 25 | NC_008208.1 |
| Aeromonas phage 31 | NC_007022.1 |
| African oil palm ringspot virus | NC_012519.1 |
| Ageratum leaf curl Cameroon betasatellite | NC_012557.1 |
| Ageratum leaf curl virus | NC_006384.1 |
| Ageratum yellow vein China virus associated DNA beta | NC_007067.1 |
| Ageratum yellow vein Hualian virus | NC_010812.1 |
| Agrotis segetum granulovirus | NC_005839.2 |
| Agrotis segetum nucleopolyhedrovirus | NC_007921.1 |
| Allamanda leaf curl virus | NC_010947.1 |
| Allium virus X | NC_012211.1 |
| Allpahuayo virus | NC_010249.1 NC_010253.1 |
| Alstroemeria virus x | NC_007408.1 |
| Alternanthera yellow vein virus | NC_007211.1 |
| Alternanthera yellow vein virus satellite DNA beta | NC_009562.1 |
| Alternaria alternata dsRNA mycovirus | NC_010984.1 - NC_010991.1 |
| Amapari virus | NC_010247.1 NC_010251.1 |
| Amasya cherry disease associated chrysovirus | NC_009944.1 - NC_009947.1 |
| Amasya cherry disease associated mycovirus | NC_006440.1 NC_006441.1 |
| American plum line pattern virus | NC_003451.1 - NC_003453.1 |
| Angelonia flower break virus | NC_007733.1 |
| Anguillid herpesvirus 1 | NC_013668.1 |
| Anopheles gambiae densonucleosis virus | NC_011317.1 |
| Antheraea pernyi nucleopolyhedrovirus | NC_008035.3 |
| Anticarsia gemmatalis nucleopolyhedrovirus | NC_008520.1 |
| Apple mosaic virus | NC_003464.1 - NC_003480.1 |
| Apricot pseudo chlorotic leaf spot virus | NC_006946.1 |
| Arabis mosaic virus | NC_006056.1 NC_006057.1 |
| Arabis mosaic virus large satellite RNA | NC_003523.1 |
| Archaeal BJ1 virus | NC_008695.1 |
| Asparagus virus 2 | NC_011807.1 - NC_011809.1 |
| Asparagus virus 3 | NC_010416.1 |
| Astrovirus MLB1 | NC_011400.1 |
| Astrovirus VA1 | NC_013060.1 |
| Atlantic salmon swim bladder sarcoma virus | NC_007654.1 |
| Avian adeno associated virus ATCC VR 865 | NC_004828.1 |
| Avian carcinoma virus | NC_001402.1 |
| Avian endogenous retrovirus EAV HP | NC_005947.1 |
| Avian myelocytomatosis virus | NC_001866.1 |
| Azospirillum phage Cd | NC_010355.1 |
| Bacillus phage 0305phi8 36 | NC_009760.1 |
| Bacillus phage AP50 | NC_011523.1 |
| Bacillus phage BCJA1c | NC_006557.1 |
| Bacillus phage Cherry | NC_007457.1 |
| Bacillus phage Fah | NC_007814.1 |
| Bacillus phage GIL16c | NC_006945.1 |
| Bacillus phage Gamma | NC_007458.1 |
| Bacillus phage IEBH | NC_011167.1 |
| Bacillus phage SPP1 | NC_004166.2 |
| Bacillus phage TP21 L | NC_011645.1 |
| Bacillus phage WBeta | NC_007734.1 |
| Bacillus phage phBC6A51 | NC_004820.1 |
| Bacillus phage phBC6A52 | NC_004821.1 |
| Bacillus phage phi105 | NC_004167.1 |
| Bacillus virus 1 | NC_009737.2 |
| Bacteriophage APSE 2 | NC_011551.1 |
| Bacteroides phage B40 8 | NC_011222.1 |
| Bamboo mosaic virus satellite RNA | NC_003497.1 |
| Banana mild mosaic virus | NC_002729.1 |
| Banana streak virus | NC_007003.1 NC_008018.1 |
| Bandicoot papillomatosis carcinomatosis virus type 1 | NC_010107.1 |
| Bandicoot papillomatosis carcinomatosis virus type 2 | NC_010817.1 |
| Barfin flounder virus BF93Hok | NC_011063.1 NC_011064.1 |
| Barley dwarf virus | NC_010798.1 |
| Barley yellow dwarf virus GAV | NC_004666.1 |
| Basella rugose mosaic virus | NC_009741.1 |
| Bat adeno associated virus YNM | NC_014468.1 |
| Bat coronavirus 1A | NC_010437.1 |
| Bat coronavirus 1B | NC_010436.1 |
| Bear Canyon virus | NC_010255.1 NC_010256.1 |
| Beet ringspot virus | NC_003693.1 NC_003694.1 |
| Beet western yellows ST9 associated virus | NC_004045.1 |
| Begomovirus associated DNA II | NC_006956.1 |
| Begomovirus associated DNA III | NC_006957.1 |
| Beilong virus | NC_007803.1 |
| Bell pepper mottle tobamovirus | NC_009642.1 |
| Bettongia penicillata papillomavirus 1 | NC_014143.1 |
| Bhendi yellow vein Bhubhaneswar virus | NC_012041.1 |
| Bitter gourd leaf curl disease associated DNA beta | NC_007655.1 |
| Black raspberry necrosis virus | NC_008182.1 NC_008183.1 |
| Black raspberry virus F | NC_009890.1 |
| Blackberry chlorotic ringspot virus | NC_011553.1 - NC_011555.2 |
| Blackcurrant reversion virus | NC_003502.1 NC_003509.1 |
| Blackcurrant reversion virus satellite RNA | NC_003872.1 |
| Blainvillea yellow spot virus | NC_010837.1 NC_010838.1 |
| Botryotinia fuckeliana partitivirus 1 | NC_010349.1 - NC_010351.1 |
| Botryotinia fuckeliana totivirus 1 | NC_009224.1 |
| Bougainvillea spectabilis chlorotic vein banding virus | NC_011592.1 |
| Bovine immunodeficiency virus | NC_001413.1 |
| Bovine papillomavirus | NC_001522.1 - NC_009752.1 |
| Bovine parvovirus 2 | NC_006259.1 |
| Bovine respiratory coronavirus AH187 | NC_012948.1 |
| Brevicoryne brassicae picorna like virus | NC_009530.1 |
| Broome virus | NC_014236.1 - NC_014245.1 |
| Bundibugyo ebolavirus | NC_014373.1 |
| Burkholderia ambifaria phage BcepF1 | NC_009015.1 |
| Burkholderia phage Bcep1 | NC_005263.2 |
| Burkholderia phage Bcep176 | NC_007497.1 |
| Burkholderia phage Bcep22 | NC_005262.2 |
| Burkholderia phage Bcep43 | NC_005342.2 |
| Burkholderia phage Bcep781 | NC_004333.2 |
| Burkholderia phage BcepB1A | NC_005886.2 |
| Burkholderia phage BcepGomr | NC_009447.1 |
| Burkholderia phage BcepIL02 | NC_012743.1 |
| Burkholderia phage BcepNY3 | NC_009604.1 |
| Burkholderia phage BcepNazgul | NC_005091.2 |
| Burkholderia phage KS10 | NC_011216.1 |
| Burkholderia phage KS9 | NC_013055.1 |
| Burkholderia phage phi1026b | NC_005284.1 |
| Burkholderia phage phi52237 | NC_007145.2 |
| Burkholderia phage phiE125 | NC_003309.1 |
| Burkholderia phage phiE12 2 | NC_009236.1 |
| Burkholderia phage phiE202 | NC_009234.1 |
| Burkholderia phage phiE255 | NC_009237.1 |
| Butterbur mosaic virus | NC_013527.1 |
| Cactus mild mottle virus | NC_011803.1 |
| Calicivirus isolate TCG | NC_006875.1 |
| California sea lion anellovirus | NC_012126.1 |
| California sea lion polyomavirus 1 | NC_013796.1 |
| Canine calicivirus | NC_004542.1 |
| Caprine arthritis encephalitis virus | NC_001463.1 |
| Capsicum chlorosis virus | NC_008301.1 - NC_008303.1 |
| Cardiospermum yellow leaf curl virus satellite DNA beta | NC_010297.1 |
| Carrot red leaf luteovirus associated RNA | NC_003871.1 |
| Casphalia extranea densovirus | NC_004288.1 |
| Caviid herpesvirus 2 | NC_011587.1 |
| Cell fusing agent virus | NC_001564.1 |
| Ceratocystis polonica partitivirus | NC_010705.1 NC_010706.1 |
| Chapare virus | NC_010562.1 NC_010563.1 |
| Chayote yellow mosaic virus | NC_004618.1 |
| Cherry green ring mottle virus | NC_001946.1 |
| Cherry necrotic rusty mottle virus | NC_002468.1 |
| Chickpea chlorotic dwarf Sudan virus | NC_010289.1 |
| Chickpea chlorotic dwarf virus | NC_011058.1 |
| Chicory yellow mottle virus large satellite RNA | NC_003778.1 |
| Chicory yellow mottle virus satellite RNA | NC_003971.1 |
| Chikungunya virus | NC_004162.2 |
| Chilli leaf curl Multan alphasatellite | NC_013103.1 |
| Chiltepin yellow mosaic virus | NC_014127.1 |
| Chlamydia phage 3 | NC_008355.1 |
| Chlamydia phage 4 | NC_007461.1 |
| Choristoneura occidentalis granulovirus | NC_008168.1 |
| Chronic bee paralysis virus | NC_010711.1 NC_010712.1 |
| Chrysodeixis chalcites nucleopolyhedrovirus | NC_007151.1 |
| Circovirus like genome BBC A | NC_013020.1 |
| Circovirus like genome CB A | NC_013028.1 |
| Circovirus like genome CB B | NC_013029.1 |
| Circovirus like genome RW A | NC_013023.1 |
| Circovirus like genome RW B | NC_013024.1 |
| Circovirus like genome RW C | NC_013025.1 |
| Circovirus like genome RW D | NC_013026.1 |
| Circovirus like genome RW E | NC_013027.1 |
| Circovirus like genome SAR A | NC_013030.2 |
| Circovirus like genome SAR B | NC_013018.1 |
| Circulifer tenellus virus 1 | NC_014360.1 |
| Citrus leaf rugose virus | NC_003546.1 - NC_003548.1 |
| Citrus sudden death associated virus | NC_006950.1 |
| Citrus variegation virus | NC_009536.1 - NC_009538.1 |
| Clanis bilineata nucleopolyhedrosis virus | NC_008293.1 |
| Clavibacter phage CMP1 | NC_013698.1 |
| Clerodendron yellow mosaic virus | NC_009451.1 |
| Clerodendrum golden mosaic China virus | NC_011346.1 NC_011347.1 |
| Clerodendrum golden mosaic virus | NC_010713.1 NC_010714.1 |
| Clostridium phage 39 O | NC_011318.1 |
| Clostridium phage c st | NC_007581.1 |
| Clostridium phage phi3626 | NC_003524.1 |
| Clostridium phage phiC2 | NC_009231.1 |
| Clostridium phage phiCD27 | NC_011398.1 |
| Clostridium phage phiCTP1 | NC_014457.1 |
| Clostridium phage phi CD119 | NC_007917.1 |
| Coconut foliar decay virus | NC_001465.1 |
| Columbid circovirus | NC_002361.1 |
| Corchorus golden mosaic virus | NC_009644.1 NC_009646.1 |
| Corchorus yellow spot virus | NC_008492.1 NC_008493.1 |
| Corchorus yellow vein virus | NC_006359.1 |
| Corynebacterium phage BFK20 | NC_009799.2 |
| Corynebacterium phage P1201 | NC_009816.1 |
| Cotton leaf curl Bangalore virus | NC_007290.1 |
| Cotton leaf curl Bangalore virus associated DNA beta | NC_007219.1 |
| Cotton leaf curl Burewala alphasatellite | NC_013803.1 |
| Cotton leaf curl Burewala betasatellite | NC_013802.1 |
| Cotton leaf curl Burewala virus | NC_012137.1 |
| Cotton leaf curl Gezira alphasatellite | NC_013593.1 |
| Cotton leaf curl Gezira beta | NC_009740.1 |
| Cotton leaf curl Gezira betasatellite | NC_013637.1 |
| Cotton leaf curl Multan betasatellite | NC_009535.1 |
| Cotton leaf curl Multan virus satellite U36 1 | NC_007721.1 |
| Cotton leaf curl virus associated DNA beta | NC_003200.1 |
| Cowpea severe leaf curl associated DNA beta | NC_006952.1 |
| Crassocephalum yellow vein virus | NC_008794.1 |
| Crocodilepox virus | NC_008030.1 |
| Croton yellow vein mosaic alphasatellite | NC_013801.1 |
| Croton yellow vein mosaic betasatellite | NC_008579.1 |
| Croton yellow vein virus | NC_014473.1 |
| Crow polyomavirus | NC_007922.1 |
| Crucifer tobamovirus | NC_003355.1 |
| Cryphonectria parasitica mitovirus 1 NB631 | NC_004046.1 |
| Cucumber mottle virus | NC_008614.1 |
| Culex flavivirus | NC_008604.2 |
| Culex pipiens densovirus | NC_012685.1 |
| Cupixi virus | NC_010252.1 NC_010254.1 |
| Curvularia thermal tolerance virus | NC_010985.1 NC_010986.1 |
| Cyanophage PSS2 | NC_013021.1 |
| Cycad leaf necrosis virus | NC_011097.1 |
| Cycas necrotic stunt virus | NC_003791.1 NC_003792.2 |
| Cymbidium ringspot virus satellite RNA | NC_004009.2 |
| Deerpox virus W 1170 84 | NC_006967.1 |
| Deformed wing virus | NC_004830.2 |
| Deftia phage phiW 14 | NC_013697.1 |
| Dendrolimus punctatus densovirus | NC_006555.1 |
| Dendrolimus punctatus tetravirus | NC_005898.1 NC_005899.1 |
| Desmodium leaf distortion virus | NC_008494.1 NC_008495.1 |
| Diaporthe ambigua RNA virus 1 | NC_001278.1 |
| Diplodia scrobiculata RNA virus 1 | NC_013699.1 |
| Dracaena mottle virus | NC_008034.1 |
| Drosophila A virus | NC_012958.1 |
| Drosophila melanogaster sigma virus AP30 | NC_013135.1 |
| Drosophila melanogaster totivirus SW 2009a | NC_013499.1 |
| Duck circovirus | NC_007220.1 |
| Duck hepatitis virus AP | NC_009750.1 |
| East Asian Passiflora virus | NC_007728.1 |
| Eastern equine encephalitis virus | NC_003899.1 |
| Ectropis obliqua picorna like virus | NC_005092.1 |
| Eimeria brunetti RNA virus 1 | NC_002701.1 |
| Elm mottle virus | NC_003568.1 - NC_003570.1 |
| Emilia yellow vein virus | NC_010307.1 |
| Emilia yellow vein virus associated DNA beta | NC_012666.1 |
| Enterobacteria phage 13a | NC_011045.1 |
| Enterobacteria phage BA14 | NC_011040.1 |
| Enterobacteria phage BP 4795 | NC_004813.1 |
| Enterobacteria phage EPS7 | NC_010583.1 |
| Enterobacteria phage ES18 | NC_006949.1 |
| Enterobacteria phage EcoDS1 | NC_011042.1 |
| Enterobacteria phage Felix 01 | NC_005282.1 |
| Enterobacteria phage Fels 2 | NC_010463.1 |
| Enterobacteria phage G4 sensu lato | NC_001420.2 |
| Enterobacteria phage ID18 sensu lato | NC_007856.1 |
| Enterobacteria phage IME08 | NC_014260.1 |
| Enterobacteria phage JK06 | NC_007291.1 |
| Enterobacteria phage JS10 | NC_012741.1 |
| Enterobacteria phage JSE | NC_012740.1 |
| Enterobacteria phage Min27 | NC_010237.1 |
| Enterobacteria phage P4 | NC_001609.1 |
| Enterobacteria phage Phi1 | NC_009821.1 |
| Enterobacteria phage PsP3 | NC_005340.1 |
| Enterobacteria phage RB14 | NC_012638.1 |
| Enterobacteria phage RB16 | NC_014467.1 |
| Enterobacteria phage RB32 | NC_008515.1 |
| Enterobacteria phage RB43 | NC_007023.1 |
| Enterobacteria phage RB51 | NC_012635.1 |
| Enterobacteria phage RB69 | NC_004928.1 |
| Enterobacteria phage RTP | NC_007603.1 |
| Enterobacteria phage SSL 2009a | NC_012223.1 |
| Enterobacteria phage TLS | NC_009540.1 |
| Enterobacteria phage WA13 sensu lato | NC_007821.1 |
| Enterobacteria phage WV8 | NC_012749.1 |
| Enterobacteria phage YYZ 2008 | NC_011356.1 |
| Enterobacteria phage cdtI | NC_009514.1 |
| Enterobacteria phage phiEcoM GJ1 | NC_010106.1 |
| Enterococcus phage EFAP 1 | NC_012419.1 |
| Enterococcus phage phiEF24C | NC_009904.1 |
| Enterococcus phage phiEf11 | NC_013696.1 |
| Enterococcus phage phiFL1A | NC_013646.1 |
| Enterococcus phage phiFL2A | NC_013643.1 |
| Enterococcus phage phiFL3A | NC_013648.1 |
| Enterococcus phage phiFL4A | NC_013644.1 |
| Enzootic nasal tumour virus of goats | NC_004994.2 |
| Eragrostis curvula streak virus | NC_012664.1 |
| Erectites yellow mosaic virus | NC_009549.1 |
| Erectites yellow mosaic virus satellite DNA beta | NC_009559.1 |
| Erwinia phage phiEa21 4 | NC_011811.1 |
| Escherichia phage D108 | NC_013594.1 |
| Escherichia phage rv5 | NC_011041.1 |
| Eupatorium vein clearing virus | NC_010738.1 |
| Euphorbia mosaic virus | NC_008304.1 NC_008305.1 |
| Euphorbia yellow mosaic virus | NC_012553.1 NC_012554.2 |
| Euproctis pseudoconspersa nucleopolyhedrovirus | NC_012639.1 |
| Feline immunodeficiency virus | NC_001482.1 |
| Feline leukemia virus | NC_001940.1 |
| Fenneropenaeus chinensis hepatopancreatic densovirus | NC_014357.1 |
| Fer de lance virus | NC_005084.2 |
| Fiji disease virus | NC_007154.1 - NC_007163.1 |
| Finch circovirus | NC_008522.1 |
| Finch polyomavirus | NC_007923.1 |
| Flexal virus | NC_010757.1 NC_010759.1 |
| Fort Morgan virus | NC_013528.1 |
| Fragaria chiloensis cryptic virus | NC_009519.1 - NC_009521.1 |
| Fragaria chiloensis latent virus | NC_006566.1 - NC_006568.1 |
| Fritillary virus Y | NC_010954.1 |
| Fusarium graminearum dsRNA mycovirus 1 | NC_006937.2 |
| Fusarium graminearum dsRNA mycovirus 3 | NC_013469.1 |
| Fusarium graminearum dsRNA mycovirus 4 | NC_013470.1 NC_013471.1 |
| GB virus A | NC_001837.1 |
| GB virus C | NC_001710.1 |
| Gammapapillomavirus HPV127 | NC_014469.1 |
| Gayfeather mild mottle virus | NC_012134.1 - NC_012136.1 |
| Geobacillus phage GBSV1 | NC_008376.2 |
| Geobacillus virus E2 | NC_009552.2 |
| Getah virus | NC_006558.1 |
| Glossina pallidipes salivary gland hypertrophy virus | NC_010356.1 |
| Goose paramyxovirus SF02 | NC_005036.1 |
| Gossypium darwinii symptomless alphasatellite | NC_013013.1 |
| Gossypium darwinii symptomless virus | NC_011804.1 |
| Gossypium davidsonii symptomless alphasatellite | NC_013011.1 |
| Gossypium mustilinum symptomless alphasatellite | NC_013012.1 |
| Gossypium punctatum mild leaf curl virus | NC_011805.1 NC_012120.1 |
| Grapevine Syrah Virus 1 | NC_012484.1 |
| Grapevine chrome mosaic virus | NC_003621.1 NC_003622.1 |
| Grapevine fanleaf virus | NC_003615.1 NC_003623.1 |
| Grapevine fanleaf virus satellite RNA | NC_003203.1 |
| Grapevine leafroll associated virus 10 | NC_011702.1 |
| Grapevine rootstock stem lesion associated virus | NC_004724.1 |
| Gremmeniella abietina RNA virus L2 | NC_005965.1 |
| Gremmeniella abietina RNA virus MS2 | NC_006444.1 - NC_006446.1 |
| Gremmeniella abietina mitochondrial RNA virus S2 | NC_006264.1 |
| Gryllus bimaculatus nudivirus | NC_009240.1 |
| Guanarito virus | NC_005077.1 NC_005082.1 |
| Gull circovirus | NC_008521.1 |
| HMO Astrovirus A | NC_013443.1 |
| Haemophilus phage Aaphi23 | NC_004827.1 |
| Haemophilus phage HP2 | NC_003315.1 |
| Haloarcula hispanica pleomorphic virus 1 | NC_013758.1 |
| Haloarcula phage SH1 | NC_007217.1 |
| Halomonas phage phiHAP 1 | NC_010342.1 |
| Halorubrum phage HF2 | NC_003345.1 |
| Halorubrum pleomorphic virus 1 | NC_012558.1 |
| Halovirus HF1 | NC_004927.1 |
| Hantavirus Z10 | NC_006433.1 - NC_006437.1 |
| Helicoverpa armigera multiple nucleopolyhedrovirus | NC_011615.1 |
| Heliothis zea virus 1 | NC_004156.1 |
| Hepatitis GB virus B | NC_001655.1 |
| Highlands J virus | NC_012561.1 |
| Hippeastrum latent virus | NC_011540.1 |
| Homalodisca vitripennis reovirus | NC_012535.1 - NC_012546.1 |
| Honeysuckle yellow vein beta | NC_009449.1 |
| Honeysuckle yellow vein mosaic disease associated satellite DNA beta | NC_009571.1 |
| Human TMEV like cardiovirus | NC_010810.1 |
| Human T lymphotropic virus 4 | NC_011800.1 |
| Human bocavirus | NC_007455.1 |
| Human bocavirus 2 | NC_012042.1 |
| Human bocavirus 3 | NC_012564.1 |
| Human bocavirus 4 | NC_012729.2 |
| Human enterovirus 107 | NC_013115.1 |
| Human enterovirus 109 | NC_014336.1 |
| Human enterovirus 98 | NC_013114.1 |
| Human erythrovirus V9 | NC_004295.1 |
| Human immunodeficiency virus 1 | NC_001802.1 |
| Human immunodeficiency virus 2 | NC_001722.1 |
| Human klassevirus 1 | NC_012986.1 |
| Human papillomavirus | NC_001357.1 - NC_001583.1 |
| Human parvovirus 4 | NC_007018.1 |
| Human rhinovirus C | NC_009996.1 |
| Humulus japonicus latent virus | NC_006064.1 - NC_006066.1 |
| Hydrangea chlorotic mottle virus | NC_012869.1 |
| Hyperthermophilic Archaeal Virus 1 | NC_014322.1 |
| Hyperthermophilic Archaeal Virus 2 | NC_014321.1 |
| Hyphantria cunea nucleopolyhedrovirus | NC_007767.1 |
| Imperata yellow mottle virus | NC_011536.1 |
| Iodobacteriophage phiPLPE | NC_011142.1 |
| Ippy virus | NC_007905.1 NC_007906.1 |
| Iranian maize mosaic nucleorhabdovirus | NC_011542.1 |
| Israel acute paralysis virus of bees | NC_009025.1 |
| J virus | NC_007454.1 |
| Japanese holly fern mottle virus | NC_013133.1 NC_013134.1 |
| Jatropha leaf curl virus | NC_011268.1 |
| Jatropha yellow mosaic India virus | NC_011309.1 |
| Junin virus | NC_005080.1 NC_005081.1 |
| KI polyomavirus | NC_009238.1 |
| Kakugo virus | NC_005876.1 |
| Kamiti River virus | NC_005064.1 |
| Kashmir bee virus | NC_004807.1 |
| Kedougou virus | NC_012533.1 |
| Kelp fly virus | NC_007619.1 |
| Kenaf leaf curl virus | NC_010435.1 |
| Klebsiella phage KP15 | NC_014036.1 |
| Klebsiella phage KP32 | NC_013647.1 |
| Klebsiella phage KP34 | NC_013649.2 |
| Klebsiella phage phiKO2 | NC_005857.1 |
| Kudzu mosaic virus | NC_009645.1 NC_009647.1 |
| Lactobacillus johnsonii prophage Lj771 | NC_010179.2 |
| Lactobacillus phage A2 | NC_004112.1 |
| Lactobacillus phage KC5a | NC_007924.1 |
| Lactobacillus phage LL H | NC_009554.1 |
| Lactobacillus phage Lb338 1 | NC_012530.1 |
| Lactobacillus phage Lc Nu | NC_007501.1 |
| Lactobacillus phage Lrm1 | NC_011104.1 |
| Lactobacillus phage Lv 1 | NC_011801.1 |
| Lactobacillus phage phiJL 1 | NC_006936.1 |
| Lactococcus phage | NC_008370.1 - NC_001835.1 |
| Lactococcus phage 1706 | NC_010576.1 |
| Lactococcus phage BK5 T | NC_002796.1 |
| Lactococcus phage KSY1 | NC_009817.1 |
| Lactococcus phage P087 | NC_012663.1 |
| Lactococcus phage asccphi28 | NC_010363.1 |
| Lactococcus phage r1t | NC_004302.1 |
| Lactococcus phage ul36 | NC_004066.1 |
| Lamium leaf distortion associated virus | NC_010737.1 |
| Lassa virus | NC_004296.1 NC_004297.1 |
| Latino virus | NC_010758.1 NC_010760.1 |
| Leucania separata nuclear polyhedrosis virus | NC_008348.1 |
| Leucas zeylanica yellow vein virus satellite DNA beta | NC_013424.1 |
| Leuconostoc phage L5 | NC_009534.1 |
| Lindernia anagallis yellow vein virus | NC_009550.1 |
| Lindernia anagallis yellow vein virus satellite DNA beta | NC_009561.1 |
| Lisianthus necrosis virus | NC_007983.1 |
| Listeria phage 2389 | NC_003291.2 |
| Listeria phage A006 | NC_009815.1 |
| Listeria phage A118 | NC_003216.1 |
| Listeria phage A500 | NC_009810.1 |
| Listeria phage A511 | NC_009811.2 |
| Listeria phage B025 | NC_009812.1 |
| Listeria phage B054 | NC_009813.1 |
| Listeria phage P35 | NC_009814.1 |
| Listeria phage P40 | NC_011308.1 |
| Listonella phage phiHSIC | NC_006953.1 |
| Little cherry virus 1 | NC_001836.1 |
| Lucky bamboo bacilliform virus | NC_009568.1 |
| Ludwigia leaf distortion betasatellite | NC_010569.1 |
| Ludwigia yellow vein virus | NC_007210.2 |
| Ludwigia yellow vein virus associated DNA beta | NC_007212.1 |
| Luffa begomovirus associated DNA beta | NC_008031.1 |
| Luffa puckering and leaf distortion associated DNA beta | NC_007459.1 |
| Lujo virus | NC_012776.1 NC_012777.1 |
| Lymantria xylina MNPV | NC_013953.1 |
| Lymphocytic choriomeningitis virus | NC_004291.1 NC_004294.1 |
| Machupo virus | NC_005078.1 NC_005079.1 |
| Macrobrachium rosenbergii nodavirus | NC_005094.1 NC_005095.1 |
| Macroptilium golden mosaic virus | NC_010952.1 NC_010953.1 |
| Magnaporthe oryzae chrysovirus 1 | NC_014462.1 - NC_014465.1 |
| Magnaporthe oryzae virus 2 | NC_010246.1 |
| Maize necrotic streak virus | NC_007729.1 |
| Mal de Rio Cuarto virus | NC_008737.1 |
| Malachra yellow vein mosaic virus associated satellite DNA beta | NC_010328.1 |
| Malvastrum leaf curl Guangdong virus | NC_008316.1 |
| Malvastrum leaf curl virus | NC_007725.1 NC_007724.1 |
| Malvastrum yellow mosaic virus | NC_008559.1 |
| Malvastrum yellow mosaic virus associated DNA 1 | NC_008561.1 |
| Malvastrum yellow mosaic virus satellite DNA beta | NC_008560.1 |
| Malvastrum yellow vein Baoshan virus | NC_012665.1 |
| Malvastrum yellow vein Yunnan virus | NC_006631.1 |
| Malvastrum yellow vein virus satellite DNA beta | NC_004733.1 |
| Mannheimia phage phiMHaA1 | NC_008201.1 |
| Maracuja mosaic virus | NC_008716.1 |
| Marine RNA virus JP A | NC_009757.1 |
| Marine RNA virus JP B | NC_009758.1 |
| Marine RNA virus SOG | NC_009756.1 |
| Marine birnavirus | NC_008019.2 NC_008026.1 |
| Marseillevirus | NC_013756.1 |
| Maruca vitrata MNPV | NC_008725.1 |
| Mayaro virus | NC_003417.1 |
| Melon chlorotic mosaic virus | NC_014380.1 NC_014381.1 |
| Melon chlorotic mosaic virus associated alphasatellite | NC_014379.1 |
| Melon yellow spot virus | NC_008300.1 - NC_008307.1 |
| Menangle virus | NC_007620.1 |
| Merkel cell polyomavirus | NC_010277.1 |
| Merremia mosaic virus | NC_007965.1 NC_007966.1 |
| Mesta yellow vein mosaic Bahraich virus | NC_010818.1 |
| Mesta yellow vein mosaic virus | NC_009088.1 |
| Mesta yellow vein mosaic virus associated DNA beta | NC_009903.1 |
| Microbacterium phage Min1 | NC_009603.1 |
| Midway virus | NC_012702.1 |
| Mimosa yellow leaf curl virus | NC_009546.1 |
| Mimosa yellow leaf curl virus associated DNA 1 | NC_009564.1 |
| Mimosa yellow leaf curl virus satellite DNA beta | NC_009556.1 |
| Mint virus 1 | NC_006944.1 |
| Mint virus X | NC_006948.1 |
| Mobala virus | NC_007903.1 NC_007904.1 |
| Montana myotis leukoencephalitis virus | NC_004119.1 |
| Mopeia Lassa reassortant 29 | NC_006572.1 NC_006573.1 |
| Morganella phage MmP1 | NC_011085.2 |
| Morogoro virus | NC_013057.1 NC_013058.1 |
| Mossman virus | NC_005339.1 |
| Mouse parvovirus 2 | NC_008186.1 |
| Mouse parvovirus 3 | NC_008185.1 |
| Mouse parvovirus 5a | NC_011618.1 |
| Mulard duck circovirus | NC_005053.1 |
| Musca domestica salivary gland hypertrophy virus | NC_010671.1 |
| Muscovy duck circovirus | NC_006561.1 |
| Mycobacterium phage 244 | NC_008194.1 |
| Mycobacterium phage Adjutor | NC_010763.1 |
| Mycobacterium phage Angel | NC_012788.1 |
| Mycobacterium phage Angelica | NC_014458.1 |
| Mycobacterium phage Ardmore | NC_013936.1 |
| Mycobacterium phage BPs | NC_010762.1 |
| Mycobacterium phage Bethlehem | NC_009878.1 |
| Mycobacterium phage Boomer | NC_011054.1 |
| Mycobacterium phage Brujita | NC_011291.1 |
| Mycobacterium phage Butterscotch | NC_011286.1 |
| Mycobacterium phage Cali | NC_011271.1 |
| Mycobacterium phage Catera | NC_008207.1 |
| Mycobacterium phage Chah | NC_011284.1 |
| Mycobacterium phage Che12 | NC_008203.1 |
| Mycobacterium phage Cooper | NC_008195.1 |
| Mycobacterium phage CrimD | NC_014459.1 |
| Mycobacterium phage DD5 | NC_011022.1 |
| Mycobacterium phage ET08 | NC_013650.1 |
| Mycobacterium phage Fruitloop | NC_011288.1 |
| Mycobacterium phage Giles | NC_009993.1 |
| Mycobacterium phage Gumball | NC_011290.1 |
| Mycobacterium phage Halo | NC_008202.1 |
| Mycobacterium phage Jasper | NC_011020.1 |
| Mycobacterium phage KBG | NC_011019.1 |
| Mycobacterium phage Konstantine | NC_011292.1 |
| Mycobacterium phage Kostya | NC_011056.1 |
| Mycobacterium phage LeBron | NC_014461.1 |
| Mycobacterium phage Llij | NC_008196.1 |
| Mycobacterium phage Lockley | NC_011021.1 |
| Mycobacterium phage Myrna | NC_011273.1 |
| Mycobacterium phage Nigel | NC_011044.1 |
| Mycobacterium phage Orion | NC_008197.1 |
| Mycobacterium phage PBI1 | NC_008198.1 |
| Mycobacterium phage PG1 | NC_005259.1 |
| Mycobacterium phage PLot | NC_008200.1 |
| Mycobacterium phage PMC | NC_008205.1 |
| Mycobacterium phage Pacc40 | NC_011287.1 |
| Mycobacterium phage Peaches | NC_013694.1 |
| Mycobacterium phage Phaedrus | NC_011057.1 |
| Mycobacterium phage Phlyer | NC_012027.1 |
| Mycobacterium phage Pipefish | NC_008199.1 |
| Mycobacterium phage Porky | NC_011055.1 |
| Mycobacterium phage Predator | NC_011039.1 |
| Mycobacterium phage Pukovnik | NC_011023.1 |
| Mycobacterium phage Qyrzula | NC_008204.1 |
| Mycobacterium phage Ramsey | NC_011289.1 |
| Mycobacterium phage Rizal | NC_011272.1 |
| Mycobacterium phage ScottMcG | NC_011269.1 |
| Mycobacterium phage Solon | NC_011267.1 |
| Mycobacterium phage Spud | NC_011270.1 |
| Mycobacterium phage TM4 | NC_003387.1 |
| Mycobacterium phage Troll4 | NC_011285.1 |
| Mycobacterium phage Tweety | NC_009820.1 |
| Mycobacterium phage U2 | NC_009877.1 |
| Mycobacterium phage Wildcat | NC_008206.1 |
| Mycoplasma phage MAV1 | NC_001942.1 |
| Mycoplasma phage P1 | NC_002515.1 |
| Mycovirus FusoV | NC_003885.1 NC_003886.1 |
| Myotis polyomavirus VM 2008 | NC_011310.1 |
| Myxococcus phage Mx8 | NC_003085.1 |
| Nanovirus like particle | NC_005954.1 |
| Narcissus symptomless virus | NC_008552.1 |
| Natrialba phage PhiCh1 | NC_004084.1 |
| Nemesia ring necrosis virus | NC_011538.1 |
| Ngaingan virus | NC_013955.1 |
| Nootka lupine vein clearing virus | NC_009017.1 |
| Nora virus | NC_007919.2 |
| Nyamanini virus | NC_012703.1 |
| Oat dwarf virus | NC_010799.1 |
| Okra leaf curl Mali virus satellite DNA beta | NC_009731.1 |
| Okra leaf curl disease associated DNA 1 | NC_010620.1 |
| Okra mottle virus | NC_011181.1 NC_011182.1 |
| Okra yellow crinkle virus | NC_008377.1 |
| Okra yellow mosaic Mexico virus | NC_014066.1 NC_014067.1 |
| Olive latent virus 3 | NC_013920.1 |
| Olive mild mosaic virus | NC_006939.1 |
| Oliveros virus | NC_010248.1 NC_010250.1 |
| Operophtera brumata reovirus | NC_007559.1 - NC_007568.1 |
| Opuntia virus X | NC_006060.1 |
| Orangutan polyomavirus | NC_013439.1 |
| Orchid fleck virus | NC_009608.1 NC_009609.1 |
| Orgyia leucostigma NPV | NC_010276.1 |
| Oryctes rhinoceros virus | NC_011588.1 |
| Ostreococcus tauri virus 1 | NC_013288.1 |
| Ostreococcus virus OsV5 | NC_010191.1 |
| Ovine lentivirus | NC_001511.1 |
| Ovine papillomavirus | NC_001789.1 |
| Oyster mushroom spherical virus | NC_004560.1 |
| Panax virus Y | NC_014252.1 |
| Papaya leaf curl China virus satellite DNA beta | NC_009555.1 |
| Paralichthys olivaceus birnavirus | NC_009923.1 NC_009924.1 |
| Paramecium bursaria Chlorella virus AR158 | NC_009899.1 |
| Parana virus | NC_010756.1 NC_010761.1 |
| Parietaria mottle virus | NC_005848.1 - NC_005854.1 |
| Passiflora latent carlavirus | NC_008292.1 |
| Passionfruit severe leaf distortion virus | NC_012786.1 NC_012787.1 |
| Pasteurella phage F108 | NC_008193.1 |
| Pedilanthus leaf curl virus | NC_012118.1 |
| Pelargonium chlorotic ring pattern virus | NC_005985.1 |
| Pelargonium line pattern virus | NC_007017.1 |
| Pelargonium necrotic spot virus | NC_005285.1 |
| Pelargonium vein banding virus | NC_013262.1 |
| Penaeid shrimp infectious myonecrosis virus | NC_007915.1 |
| Penaeus merguiensis densovirus | NC_007218.1 |
| Penaeus monodon hepatopancreatic parvovirus | NC_011545.2 |
| Pennisetum mosaic virus | NC_007147.1 |
| Pepper curly top virus | NC_009518.1 |
| Pepper leaf curl Yunnan virus satellite DNA beta | NC_010619.1 |
| Pepper leaf curl virus satellite DNA beta | NC_010235.1 |
| Pepper yellow dwarf virus New Mexico | NC_011188.1 |
| Pepper yellow leaf curl Indonesia virus | NC_008283.1 NC_008284.1 |
| Peruvian horse sickness virus | NC_007748.1 - NC_007757.1 |
| Pestivirus Giraffe 1 | NC_003678.1 |
| Phage Gifsy 1 | NC_010392.1 |
| Phage Gifsy 2 | NC_010393.1 |
| Phage phiJL001 | NC_006938.1 |
| Phlebiopsis gigantea mycovirus dsRNA 1 | NC_013999.1 |
| Phlox Virus B | NC_009383.1 |
| Phlox virus S | NC_009383.1 |
| Phormidium phage Pf WMP3 | NC_009551.1 |
| Phormidium phage Pf WMP4 | NC_008367.1 |
| Phytophthora infestans RNA virus 1 | NC_013220.1 NC_013221.1 |
| Pichinde virus | NC_006439.1 NC_006447.1 |
| Pieris rapae granulovirus | NC_013797.1 |
| Pirital virus | NC_005894.1 NC_005897.1 |
| Plutella xylostella multiple nucleopolyhedrovirus | NC_008349.1 |
| Poinsettia mosaic virus | NC_002164.1 |
| Polyomavirus HPyV6 | NC_014406.1 |
| Polyomavirus HPyV7 | NC_014407.1 |
| Potato Virus P | NC_004039.1 |
| Potato apical leaf curl disease associated satellite DNA beta | NC_008605.1 |
| Potato virus T | NC_011062.1 |
| Prochlorococcus phage P SSM4 | NC_006884.2 |
| Propionibacterium phage PA6 | NC_009541.1 |
| Prune dwarf virus | NC_008037.1 - NC_008039.1 |
| Prunus necrotic ringspot virus | NC_004362.1 - NC_004364.1 |
| Pseudomonas phage 119X | NC_007807.1 |
| Pseudomonas phage 14 1 | NC_011703.1 |
| Pseudomonas phage 73 | NC_007806.1 |
| Pseudomonas phage B3 | NC_006548.1 |
| Pseudomonas phage D3 | NC_002484.1 |
| Pseudomonas phage D3112 | NC_005178.1 |
| Pseudomonas phage DMS3 | NC_008717.1 |
| Pseudomonas phage F10 | NC_007805.1 |
| Pseudomonas phage F116 | NC_006552.1 |
| Pseudomonas phage F8 | NC_007810.1 |
| Pseudomonas phage LBL3 | NC_011165.1 |
| Pseudomonas phage LIT1 | NC_013692.1 |
| Pseudomonas phage LMA2 | NC_011166.1 |
| Pseudomonas phage LUZ19 | NC_010326.1 |
| Pseudomonas phage LUZ7 | NC_013691.1 |
| Pseudomonas phage M6 | NC_007809.1 |
| Pseudomonas phage MP22 | NC_009818.1 |
| Pseudomonas phage MP29 | NC_011613.1 |
| Pseudomonas phage MP38 | NC_011611.1 |
| Pseudomonas phage PA11 | NC_007808.1 |
| Pseudomonas phage PAJU2 | NC_011373.1 |
| Pseudomonas phage PB1 | NC_011810.1 |
| Pseudomonas phage PP7 | NC_001628.1 |
| Pseudomonas phage PRR1 | NC_008294.1 |
| Pseudomonas phage PaP2 | NC_005884.1 |
| Pseudomonas phage SN | NC_011756.1 |
| Pseudomonas phage YuA | NC_010116.1 |
| Pseudomonas phage phi2954 | NC_012091.2 - NC_012093.1 |
| Pseudomonas phage phi 2 | NC_013638.1 |
| Pseudomonas phage phikF77 | NC_012418.1 |
| Pumpkin yellow mosaic Malaysia virus | NC_010946.1 |
| Pyrococcus abyssi virus 1 | NC_009597.1 |
| Quang Binh virus | NC_012671.1 |
| RD114 retrovirus | NC_009889.1 |
| Rabbit calicivirus Australia 1 MIC 07 | NC_011704.1 |
| Rabbit vesivirus | NC_008580.1 |
| Radish leaf curl virus | NC_010238.1 NC_014040.1 |
| Radish leaf curl virus betasatellite | NC_010239.1 |
| Ralstonia phage RSB1 | NC_011201.1 |
| Ralstonia phage RSL1 | NC_010811.2 |
| Ralstonia phage RSM1 | NC_008574.1 |
| Ralstonia phage RSM3 | NC_011399.1 |
| Ralstonia phage RSS1 | NC_008575.1 |
| Ralstonia phage p12J | NC_005131.2 |
| Ralstonia phage phiRSA1 | NC_009382.1 |
| Ramie mosaic virus | NC_010791.1 NC_010792.1 |
| Raphanus sativus cryptic virus 1 | NC_008190.1 NC_008191.1 |
| Raphanus sativus cryptic virus 2 | NC_010343.1 - NC_010345.1 |
| Raphanus sativus cryptic virus 3 | NC_011705.1 NC_011706.1 |
| Raspberry leaf mottle virus | NC_008585.1 |
| Raspberry ringspot virus | NC_005266.1 NC_005267.1 |
| Rattus norvegicus papillomavirus 1 EES 2009 | NC_013196.1 |
| Raven circovirus | NC_008375.1 |
| Respiratory syncytial virus | NC_001803.1 |
| Reticuloendotheliosis virus | NC_006934.1 |
| Rhizobium phage 16 3 | NC_011103.1 |
| Rhododendron virus A | NC_014481.1 |
| Rhynchosia golden mosaic Yucatan virus | NC_012481.1 NC_012482.1 |
| Rose cryptic virus 1 | NC_010346.1 - NC_010348.1 |
| Roseobacter phage SIO1 | NC_002519.1 |
| Ross River virus | NC_001544.1 |
| Rubus chlorotic mottle virus | NC_011187.1 |
| Rudbeckia flower distortion virus | NC_011920.1 |
| Sabia virus | NC_006313.1 NC_006317.1 |
| Saccharum streak virus | NC_013464.1 |
| Saffold virus | NC_009448.2 |
| Salmonella phage Fels 1 | NC_010391.1 |
| Salmonella phage SE1 | NC_011802.1 |
| Salmonella phage SETP3 | NC_009232.1 |
| Salmonella phage ST64B | NC_004313.1 |
| Salmonella phage c341 | NC_013059.1 |
| Salmonella phage epsilon34 | NC_011976.1 |
| Schlumbergera virus X | NC_011659.1 |
| Sclerophthora macrospora virus B | NC_004714.1 |
| Sclerotinia sclerotiorum hypovirulence associated DNA virus 1 | NC_013116.1 |
| Sclerotinia sclerotiorum partitivirus S | NC_013014.1 NC_013015.1 |
| Sea turtle tornovirus 1 | NC_012094.1 |
| Seal picornavirus type 1 | NC_009891.1 |
| Semliki forest virus | NC_003215.1 |
| Senecio yellow mosaic virus | NC_006995.1 |
| Shigella phage phiSboM AG3 | NC_013693.1 |
| Sida golden mottle virus | NC_014128.1 NC_014130.1 |
| Sida leaf curl virus | NC_007638.1 |
| Sida leaf curl virus associated DNA 1 | NC_007640.1 |
| Sida leaf curl virus associated DNA beta | NC_007639.1 |
| Sida leaf curl virus satellite DNA beta | NC_009557.1 |
| Sida mosaic Sinaloa virus | NC_008056.1 NC_008059.1 |
| Sida yellow vein Madurai virus | NC_009354.1 |
| Sida yellow vein Vietnam virus | NC_009547.1 |
| Sida yellow vein Vietnam virus associated DNA 1 | NC_009563.1 |
| Sida yellow vein Vietnam virus satellite DNA beta | NC_009558.1 |
| Sida yellow vein disease associated DNA 1 | NC_014065.1 |
| Sida yellow vein virus satellite DNA beta | NC_007213.1 |
| Siegesbeckia yellow vein Guangxi virus | NC_008317.1 |
| Siegesbeckia yellow vein virus | NC_008236.1 |
| Siegesbeckia yellow vein virus associated DNA beta | NC_008237.1 |
| Simian Human immunodeficiency virus | NC_001870.1 |
| Simian T cell lymphotropic virus 6 | NC_011546.1 |
| Simian agent 12 | NC_007611.1 |
| Simian enterovirus SV19 | NC_010412.1 |
| Simian enterovirus SV43 | NC_010413.1 |
| Simian enterovirus SV6 | NC_010415.1 |
| Simian immunodeficiency virus | NC_004455.1 NC_001549.1 |
| Simian picornavirus 1 | NC_004451.1 |
| Simian picornavirus 17 | NC_010411.1 |
| Simian retrovirus 4 | NC_014474.1 |
| Sindbis virus | NC_001547.1 |
| Siniperca chuatsi rhabdovirus | NC_008514.1 |
| Slow bee paralysis virus | NC_014137.1 |
| Small anellovirus | NC_007013.1 NC_007014.1 |
| Snake adenovirus | NC_009989.1 |
| Snake parvovirus 1 | NC_006148.1 |
| Sodalis phage SO 1 | NC_013600.1 |
| Sodalis phage phiSG1 | NC_007902.1 |
| Soft shelled turtle iridovirus | NC_012637.1 |
| Solenopsis invicta virus 1 | NC_006559.1 |
| Solenopsis invicta virus 2 | NC_009544.1 |
| Solenopsis invicta virus 3 | NC_012531.1 |
| Southern tomato virus | NC_011591.1 |
| Soybean chlorotic blotch virus | NC_014141.1 NC_014142.1 |
| Soybean mild mottle virus | NC_014140.1 |
| Soybean yellow mottle mosaic virus | NC_011643.1 |
| Spilanthes yellow vein virus | NC_009545.1 |
| Spinach curly top virus | NC_005860.1 |
| Spinach latent virus | NC_003808.1 - NC_003810.1 |
| Spiroplasma phage SVTS2 | NC_001270.2 |
| Spissistilus festinus virus 1 | NC_014359.1 |
| Spodoptera litura granulovirus | NC_009503.1 |
| Spodoptera litura nucleopolyhedrovirus II | NC_011616.1 |
| Sputnik virophage | NC_011132.1 |
| Squash vein yellowing virus | NC_010521.1 |
| Squirrel monkey polyomavirus | NC_009951.1 |
| Staphylococcus phage 11 | NC_004615.1 |
| Staphylococcus phage 187 | NC_007047.1 |
| Staphylococcus phage 2638A | NC_007051.1 |
| Staphylococcus phage 37 | NC_007055.1 |
| Staphylococcus phage 3A | NC_007053.1 |
| Staphylococcus phage 42E | NC_007052.1 |
| Staphylococcus phage 55 | NC_007060.1 |
| Staphylococcus phage 77 | NC_005356.1 |
| Staphylococcus phage 80alpha | NC_009526.1 |
| Staphylococcus phage CNPH82 | NC_008722.1 |
| Staphylococcus phage EW | NC_007056.1 |
| Staphylococcus phage G1 | NC_007066.1 |
| Staphylococcus phage K | NC_005880.1 |
[truncated: 7,982 more chars]
