# Supplementary figures and images for: Consistency of metagenomic assignment programs in simulated and real data
Source: BMC Bioinformatics. 2014 Mar 28;15:90. doi: 10.1186/1471-2105-15-90 (PMC3986635; doi:10.1186/1471-2105-15-90)

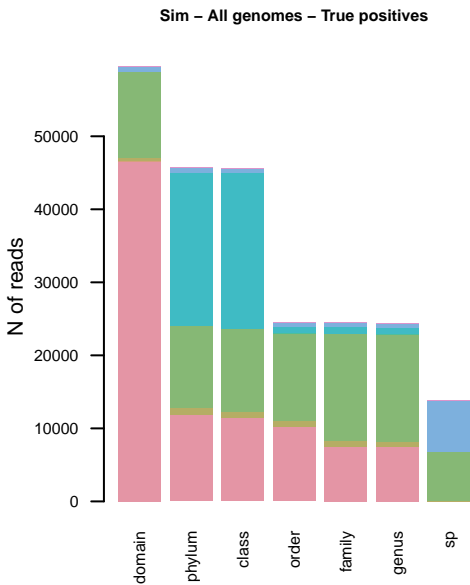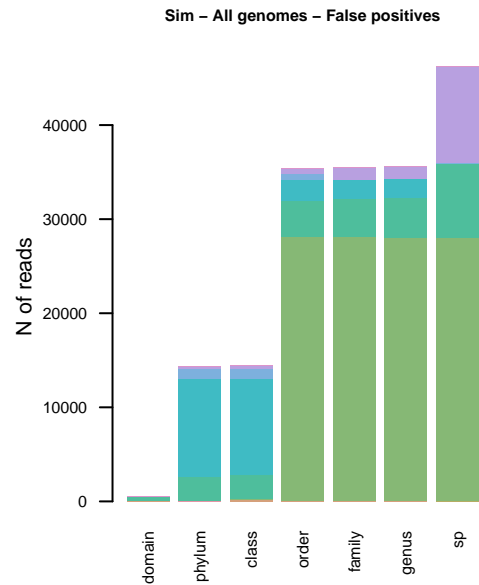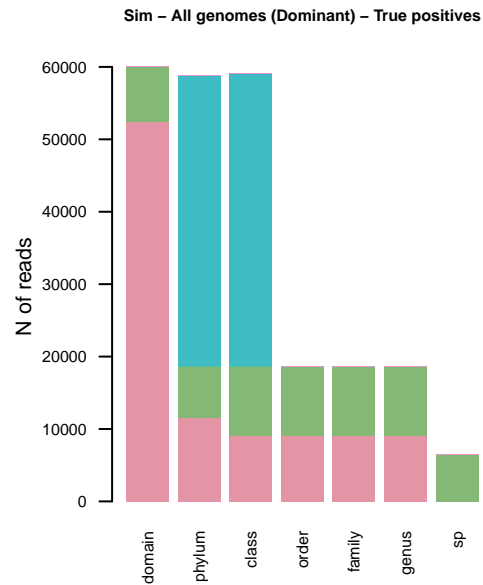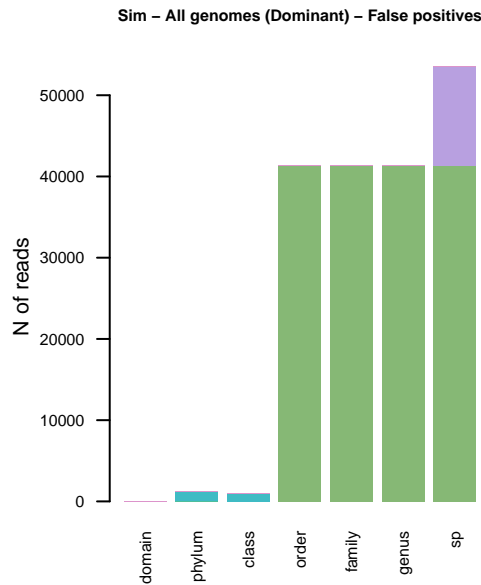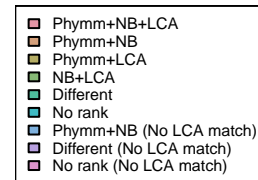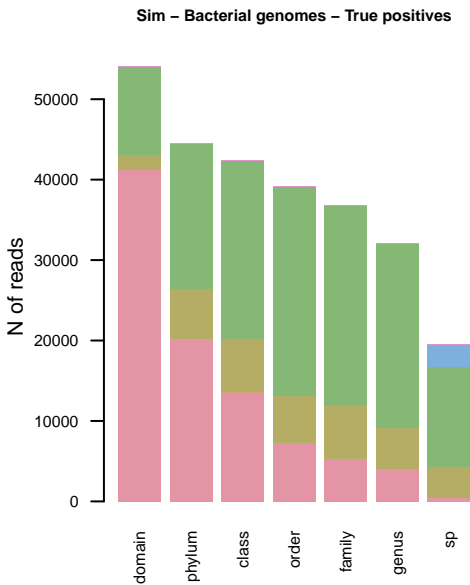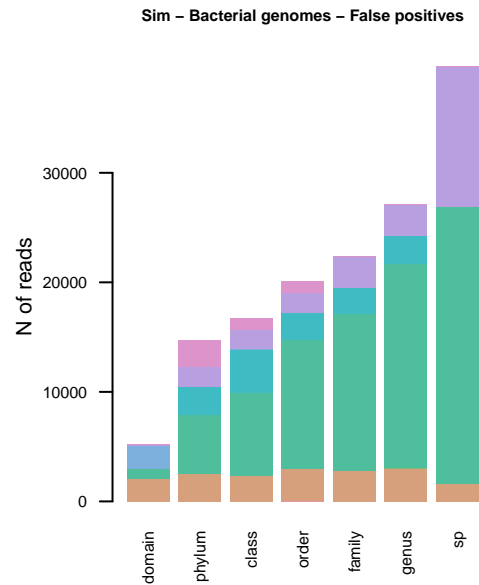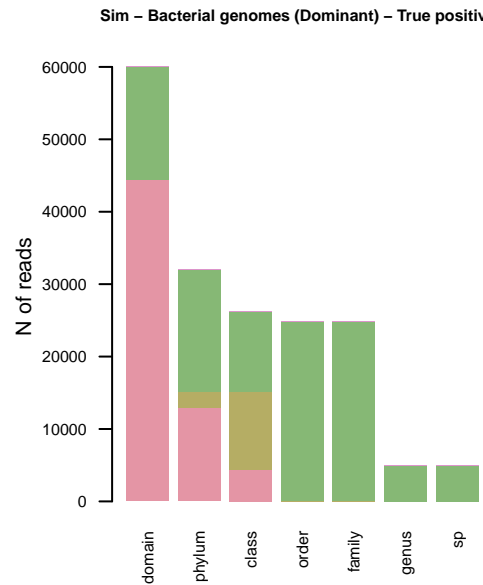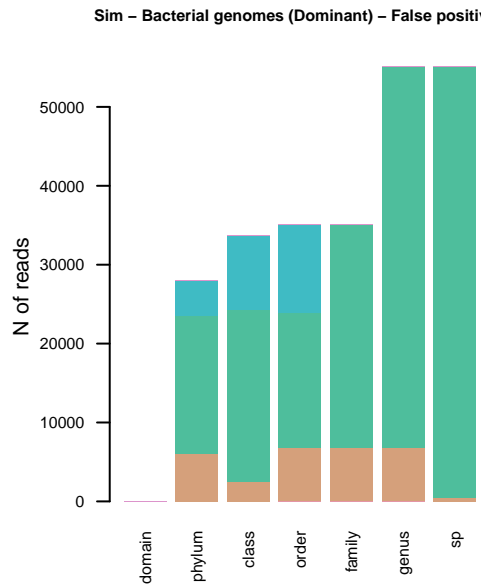

Supplement: Additional file 2: Figure S1 — Number of reads assigned in each taxonomic level in simulated data according to true positives and false positives. [file 1471-2105-15-90-S2.pdf]

**MetaSim 1**

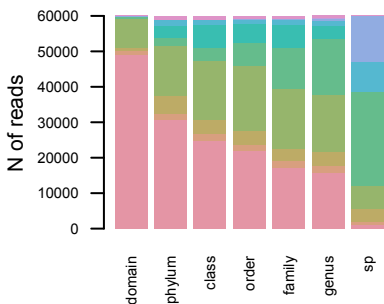

**MetaSim 2**

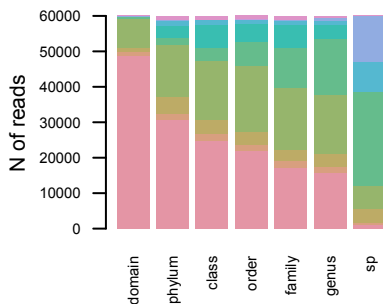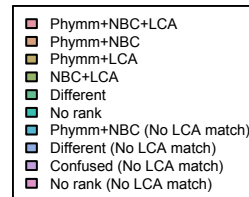

**MetaSim 3**

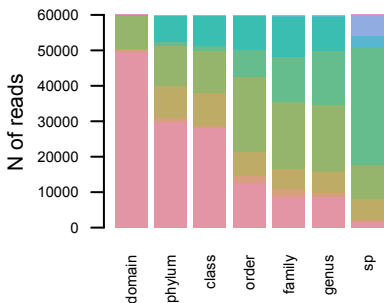

**MetaSim 4**

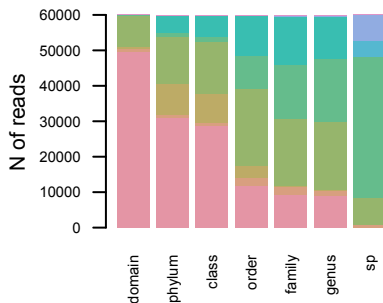

**iMESS 1**

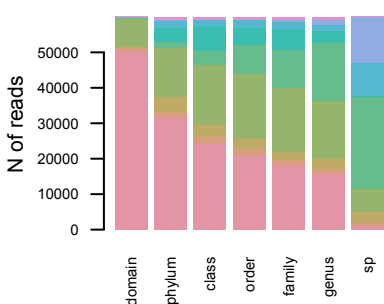

**iMESS 2**

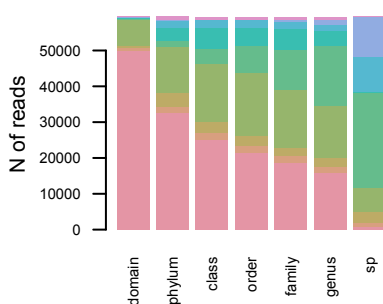

Supplement: Additional file 3: Figure S2 — Number of reads assigned in each taxonomic level in simulated data generated by Metasim. [file 1471-2105-15-90-S3.pdf]
